# APOBEC3G-Induced Hypermutation of Human Immunodeficiency Virus Type-1 Is Typically a Discrete “All or Nothing” Phenomenon

**DOI:** 10.1371/journal.pgen.1002550

**Published:** 2012-03-22

**Authors:** Andrew E. Armitage, Koen Deforche, Chih-hao Chang, Edmund Wee, Beatrice Kramer, John J. Welch, Jan Gerstoft, Lars Fugger, Andrew McMichael, Andrew Rambaut, Astrid K. N. Iversen

**Affiliations:** 1 MRC Human Immunology Unit, Weatherall Institute of Molecular Medicine, Oxford University, Oxford, United Kingdom; 2 Rega Institute for Medical Research, Katholieke Universiteit Leuven, Leuven, Belgium; 3 Department of Infectious Diseases, King's College London School of Medicine, London, United Kingdom; 4 Department of Genetics, University of Cambridge, Cambridge, United Kingdom; 5 Department of Infectious Diseases, Rigshospitalet, The National University Hospital, Copenhagen, Denmark; 6 Department of Clinical Neurology, John Radcliffe Hospital, Oxford University, Oxford, United Kingdom; 7 Institute of Evolutionary Biology, University of Edinburgh, Edinburgh, United Kingdom; 8 The Peter Medawar Building for Pathogen Research, Nuffield Department of Medicine, Oxford University, Oxford, United Kingdom; University of Arizona, United States of America

## Abstract

The rapid evolution of Human Immunodeficiency Virus (HIV-1) allows studies of ongoing host–pathogen interactions. One key selective host factor is APOBEC3G (hA3G) that can cause extensive and inactivating Guanosine-to-Adenosine (G-to-A) mutation on HIV plus-strand DNA (termed hypermutation). HIV can inhibit this innate anti-viral defense through binding of the viral protein Vif to hA3G, but binding efficiency varies and hypermutation frequencies fluctuate in patients. A pivotal question is whether hA3G-induced G-to-A mutation is always lethal to the virus or if it may occur at sub-lethal frequencies that could increase viral diversification. We show *in vitro* that limiting-levels of hA3G-activity (i.e. when only a single hA3G-unit is likely to act on HIV) produce hypermutation frequencies similar to those in patients and demonstrate *in silico* that potentially non-lethal G-to-A mutation rates are ∼10-fold lower than the lowest observed hypermutation levels *in vitro* and *in vivo*. Our results suggest that even a single incorporated hA3G-unit is likely to cause extensive and inactivating levels of HIV hypermutation and that hypermutation therefore is typically a discrete “all or nothing” phenomenon. Thus, therapeutic measures that inhibit the interaction between Vif and hA3G will likely not increase virus diversification but expand the fraction of hypermutated proviruses within the infected host.

## Author Summary

Human cells have conserved antiviral defense systems, which protect against a range of viruses. A key component of this innate, intra-cellular defense is APOBEC3G (hA3G), which can cause extensive and inactivating G-to-A mutations (termed hypermutation) in viral DNA. To circumvent this, human immunodeficiency virus type-1 (HIV-1) encodes a protein, Vif, which can bind hA3 and prevent its antiviral effects. Vif is however, not always fully efficient, and many HIV-1 infected patients harbor hypermutated sequences. A key question is whether hA3G also might generate sub-lethal levels of G-to-A mutations, which could increase viral evolution, possibly accelerating disease progression. If this were to occur, drugs and vaccine-induced CTL-responses targeting Vif might have counterproductive effects. We show through *in vitro*, *in vivo*, and *in silico* analyses that it is unlikely that hA3G-activity can enhance virus evolution. Thus, measures that inhibit the interaction between Vif and APOBEC3G are likely to only increase the fraction of hypermutated, inactivated HIV sequences in the infected host.

## Introduction

The HIV-1 population within an infected individual is characterized by extensive viral variation and continuous adaptation to its host. Such rapid evolution is the result of a combination of several factors: a large viral population, high replication and mutation rates, recombination, and various intra-host selective pressures [1]. The high mutation rate is associated with the inherent infidelity of HIV reverse transcriptase (RT) and RNA polymerase II (RNA pol II) [1] and has also been proposed to be partly caused by cellular cytidine deaminases such as hA3G, which can cause Guanosine-to-Adenosine (G-to-A) mutations on HIV plus-strand DNA [2]–[7]. Several observations appear to provide support for this hypothesis as lentiviral genomes are adenine rich [8], [9] and G-to-A is the most frequent nucleotide mutation observed during HIV-1 replication both *in vitro* [10], [11] and *in* vivo in both acute [12] and chronic infection [13].

In infected cells, hA3G can become incorporated into nascent virions as large, enzymatically inactive, ribonucleoprotein complexes termed ‘Intra-Virion A3G Complexes’ (IVAC) [14]. When a virion subsequently infects another cell, IVACs become active through the activity of viral RNaseH during reverse transcription [14] and hA3G restricts HIV replication through a combination of mutagenesis (or editing) [5], [15] and possibly non-editing activities [16]. Editing is easily recognized because it results in extensive Cytidine-to-Uridine (C-to-U) deamination of single-stranded minus-strand DNA during reverse transcription [5], [17], [18]. The mutations appear as plus-strand G-to-A changes and hA3-induced mutations are usually reported as such and termed hypermutation [19] as G-to-A transitions far exceed all other mutations. As the preferred target is TGG (encoding Tryptophan when in frame), many G-to-A mutations will produce stop-codons, TAG, resulting in viral inactivation [17], [20].

The HIV accessory protein Vif can circumvent the protective role of hA3G, and other hA3 deaminases, by targeting them for proteasomal degradation and thereby preventing their incorporation into virions [21]. However, as various frequencies of hypermutated sequences are observed in HIV DNA from infected patients, the efficiency of these Vif-hA3 interactions must vary between them [4], [22]–[24].

Two different scenarios could account for the *in vivo* variation in hypermutation frequency. First, editing could act to increase viral diversification, with possible advantages to the virus in a fluctuating fitness environment, but to do so, hA3G would have to induce mutations at a low, sub-lethal level. In such a situation, selection would act on Vif to moderate the number of hA3G molecules incorporated into virions. Alternatively, inefficient Vif-hA3G interactions could be the by-product of other hitherto undefined selective pressures and the resulting hypermutation considered a viral fitness cost, acting at the level of the viral population.

Here, we investigate the fundamental question of whether hA3G-induced G-to-A mutation is always lethal to the virus or if it may occur at sub-lethal frequencies.

## Results

### hA3G levels and mutation rates *in vitro* and *in vivo*

To examine whether limiting-levels of hA3G activity could result in sub-lethal mutation rates in HIV infections, we designed an *in vitro* hA3G titration and sequencing experiment (Table 1). Briefly, we made Vesicular Stomatitis Virus G protein (VSV-G) pseudotyped Δ*vif*-HIV(IIIB) virions, which incorporated variable amounts of editing wild-type hA3G (wt-hA3G). The total hA3G concentration was kept constant using the E259Q non-editing hA3G mutant (E259Q-hA3G) [25]. These viruses were used to infect TZM-bl cells (a HeLa cell line expressing HIV coreceptors and a *lacZ* reporter gene under the control of an HIV LTR) in a single-cycle infection assay from which DNA was extracted and provirus amplified using limiting-dilution nested-PCR.

**Table 1:** hA3G titration transfection conditions.

Transfection condition	VH17 (µg)	wt- hA3G (µg)	E259Q-hA3G (µg)	Total hA3G (µg)	pCMV4HA (µg)	VSV-G (µg)
1	3	0	1	1	–	0.15
2	3	0.01	0.99	1	–	0.15
3	3	0.033	0.967	1	–	0.15
4	3	0.1	0.9	1	–	0.15
5	3	0.33	0.67	1	–	0.15
6	3	1	0	1	–	0.15
7	3	0	0	0	1	0.15

VH17 = Δ*vif* HIV-1(IIIB) proviral construct.

wt-hA3G = wild-type editing hA3G construct.

E259Q-hA3G = E259Q non-editing mutant hA3G construct.

pCMV4HA = Empty vector.

VSV-G = Vesicular Stomatitis Virus-G envelope construct.

**Figure 1:**
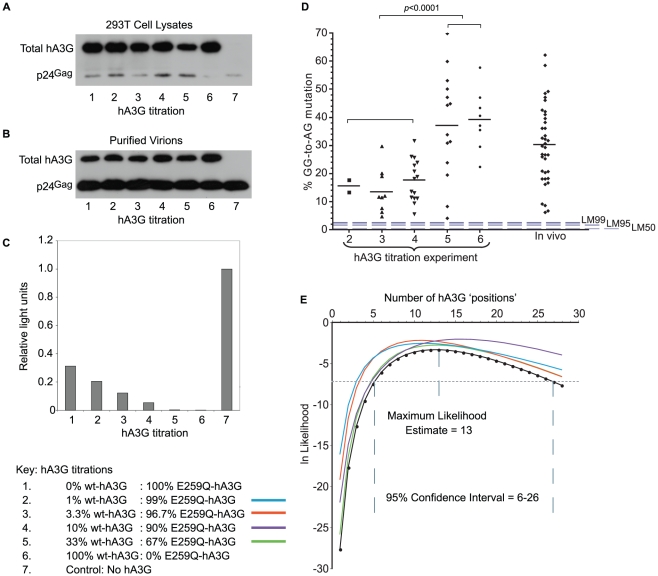
hA3G-induced mutation frequency *in vitro* and *in vivo*. A, B. VSV-G pseudotyped Vif-deficient HIV-1 virions were harvested from supernatants of 293T cells transfected with plasmids encoding Δ*vif*-HIV-1(IIIB), VSV-G envelope and variable amounts of wt- and non-editing E259Q-hA3G (Table 1). Total hA3G expression was examined using an antibody that binds to both wt- and E259Q-hA3G in immunoblots of both producer cell lysates (1A) and purified virions (1B) at each titration; the transfection efficiency of both editing and non-editing hA3G was comparable. HIV-1 p24 protein was used as a loading control. C. Relative single-cycle infectivities of viruses from each titration were quantified by infecting TZM-bl cells that express β-galactosidase under the control of an HIV-1 LTR with quantities of virus normalized by p24 ELISA. Infectivities were quantified using a β-galactosidase reporter assay. The light signal gave a read-out of β-galactosidase production, which was proportional to the infectivity of the infecting virus. Infectivities are expressed relative to that of VSV-G pseudotyped Δ*vif*-HIV-1(IIIB) generated in the absence of any hA3G (condition 7). D. Using single genome amplification, HIV-1 *env*-3′LTR sequences were obtained from DNA isolated from cells infected with viruses from titration conditions 1–6. GG-to-AG (hA3G preferred dinucleotide) mutation rates were determined in each hypermutated virus from each condition relative to the known parental virus sequence; non-hypermutated sequences are not included in this analysis; each data point represents the editing rate in one sequence; for comparison with naturally occurring editing rates, GG-to-AG mutation rates were estimated in 39 patient-derived hypermutated sequences (“in vivo”); dashed lines represent LM50/LM95/LM99 editing rates from the *in silico* simulations described in Figure 3. E. We used the proportions of sequences carrying hA3G-type editing from each titration condition (Table 1 and Table 2) to generate a Maximum Likelihood Estimate of the number of “positions” in a virion potentially occupied by wt-hA3G units; colours denote the data set omitted from the calculations in order to examine if all titrations contributed equally to the estimation.

We examined total hA3G expression in both producer cell lysates (Figure 1A) and purified virions (Figure 1B) for each titration to test that transfections of both editing and non-editing hA3G were equally efficient. Viruses with hA3G (wt- or E259Q-hA3G) displayed large reductions in infectivity compared to virus generated without hA3G, and the presence of increasing concentrations of wt-hA3G conferred relatively greater losses of infectivity, in line with previous studies (Figure 1C) [26].

We amplified and sequenced 8–20 *env*-to-3′LTR fragments (2.1 kb) from each hA3G titration. As the sequence of the parental HIV(IIIB) virus is known (Figure S1), and the infections in our experiments restricted to a single replication cycle, we could readily identify all mutations induced by hA3G using HYPERMUT (www.hiv.lanl.gov). We found that 33/87 sequences had no plus-strand G-to-A mutations while 48/87 were hypermutated carrying greater than 4% GG-to-AG mutations (Figure 1D, Table S1). In the remaining six sequences, a single G-to-A change was found in either non-hA3G (5/6) or rare (1/6) hA3G contexts (as defined in [20]), suggesting that RT/RNA pol II or PCR-related errors may have been responsible. Of the hypermutated sequences, all but one (47/48) carried stop codons, and as the sequenced region corresponds to only ∼20% of the protein-coding genome, stop-codons likely exist in the rest of the genome. Hypermutation levels in the lower three wt-hA3G titrations were significantly lower than those in the higher titrations (p<0.0001, unpaired t-test) (Figure 1D).

To evaluate whether these *in vitro* hypermutation rates were representative of those occurring *in vivo*, we estimated the mutation levels of 39 near-full length hypermutated patient-derived proviruses (www.hiv.lanl.gov). As the parental viral sequences were unknown, we made optimized reference sequences as in [20]. Briefly, reference sequences were estimated as the consensus of closely related sequences identified by NJ phylogenetic tree analysis of HIV subtype alignments in which potential hA3-type hypermutation sites were ‘repaired’ (i.e. all AG and AA sites were changed to NG and NA, respectively, if a GG or a GA was also present at the same position in the alignment). We found that the hypermutation levels observed *in vivo* were similar to those observed *in vitro* (Figure 1D). Due to the lack of original patient-derived non-hypermutated reference sequences, we were unable to distinguish whether GG-to-AG mutation levels at <5% of all GG targets in these sequences were caused by hA3G or RT/RNA pol II; however, an abundance of sequences with such low hypermutation levels would imply a bimodal distribution of mutation levels in natural infections, which would be inconsistent with the *in vitro* data.

### One editing-hA3G unit is likely to cause extensive hypermutation

For hA3G editing to contribute to viral adaptation, the induced mutations would need to occur at low, sub-lethal levels. This is most likely to happen if just a single editing hA3G-unit is incorporated into the virion. As hA3G may undergo RNA-dependent oligomerization during virion assembly, the term hA3G-unit is used here to refer to the active hA3G deaminase [27]. We cannot know for certain whether the hypermutants we observed *in vitro* did result from the incorporation of a single editing unit, but conditional on assumptions about the incorporation process, we can estimate the probability that this was so.

We examined the maximum number of hA3G units that could reside in a virion by considering the proportion of sequences carrying hypermutation at each titration to derive a maximum likelihood estimate (MLE) of the number of editing hA3G-units per virion (Figure 1E, Figure S1). As the estimate depends on hypermutation being observed, only the number of incorporated hA3G-units with editing activity is estimated.

Our analysis assumed (i) that there is a finite number of positions in a virion that can be occupied by hA3G-editing units [28]; (ii) that the efficiency of transfection, protein expression, and virion incorporation is the same for editing and non-editing hA3G (as supported by Figure 1A and [28]; (iii) that there was sufficient hA3G present in each titration for all positions to be occupied by either editing or non-editing hA3G (as supported by the 100% detection rate when 100% wild-type hA3G was present (Table S1) (iv) that hA3G editing, when it had occurred in a sampled sequence, was always successfully detected; and (v) that degradation of uracil-containing edited viral DNA by cellular uracil DNA glycosidases such as UNG2 and/or SMUG1 was insignificant [3], [29]–[31]. Under these assumptions, the probability that an observed hypermutant resulted from a single wild-type hA3G unit is approximately 1−(*k*−1)*r*/2, where *k* is the maximum possible number of hA3G units that can be incorporated into a single virion, and *r* is the proportion of hA3G present that was wild-type when the hypermutant was generated (see Materials and Methods for full details and Table 2).

**Table 2:** Transfection condition and hypermutation results.

Transfection condition			r, Proportion of wt-hA3G in transfection	h, Number of hypermutated sequences	n, Total number of sequences	% sequences hypermutated
	% wild-type hA3G	% E259Q-hA3G				
**1**	0	100	0	0	7	0.0
**2**	1	99	0.01	2	19	10.5
**3**	3.3	96.7	0.033	9	19	47.4
**4**	10	90	0.1	15	19	78.9
**5**	33	67	0.33	14	15	93.3
**6**	100	0	1	8	8	100.0
**7**	0	0	0	-	-	-

**Figure 2:**
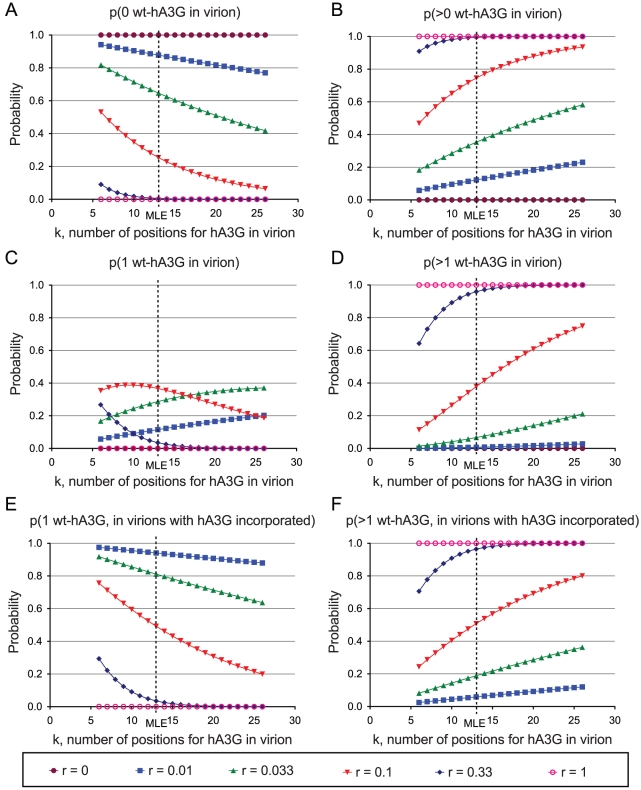
Probabilities of incorporation of editing wild-type hA3G units into progeny virions in the hA3G titration experiment. The sensitivity of the MLE analysis in Figure 1E to each individual condition was assessed by re-estimating *_k_* after removing each state in turn. The values of *r_i_*, *n_i_*, and *h_i_* are shown in Table 2. In each case, *k* refers to the hypothetical number of positions within a virion that could be occupied by wt-hA3G units that can induce hypermutation; *r* denotes the proportion of wt-hA3G in the titration experiment (the proportion of non-editing E259Q-mutant hA3G is 1−*r*). Probabilities for each of the values of *k* spanning the 95% C.I. of the maximum likelihood estimate of the number of wt-hA3G units are shown (MLE = 13; 95% C.I. = 6–26), and are based on the binomial distribution. (A–D) Probabilities of virions incorporating (A) zero wt-hA3G units, (B) at least one wt-hA3G unit, (C) exactly one wt-hA3G unit, (D) two or more wt-hA3G units, for each model are shown. (E–F) graphs depicting the probability of hypermutation having been caused by (E) a single wt-hA3G unit, or (F) more than one wt-hA3G unit.

The probability that we have observed the minimal level of hA3G-induced hypermutation therefore depends on the number of available positions, denoted *k*. Using assumptions (i)–(v) listed above, we were able to derive a maximum likelihood estimator of *k* that could be applied to the results of our titration experiments (see Materials and Methods and Figure 1E). In this way, we estimated that a virion could accommodate *_k_* = 13 editing hA3G-units (95% CI: 6–26 units) – an estimate that was robust to the removal of each titration condition in turn (Figure 1E, Figure 2). This estimate was similar to a previous biochemical estimate of 7+/−4 molecules [28].

This estimate implies that in our transfection condition 2, in which 1% of the hA3G was wild-type (*r* = 0.01), an expected 1−(13−1)0.01/2 = 94% of hypermutants are predicted to have resulted from the incorporation of a single virion. This figure rises to 97% if we take the previous biochemical estimates of *k* (*k* = 7 molecules; [28]), and remains as high as 87.5% if we take our upper confidence interval (*_k_* = 26).

**Figure 3:**
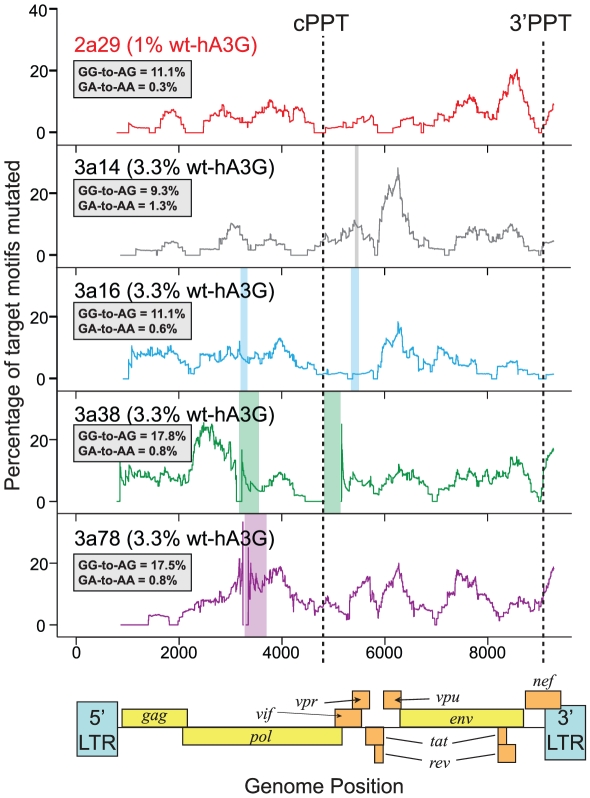
Hypermutation is induced throughout HIV-1 genomes mutated by one wt-hA3G unit. We generated near-full length sequences (*gag* to 3′LTR) of a subset of hypermutated viruses from the lower wt-hA3G titrations (1% wt-hA3G:99% E259Q-hA3G (2a29) or 3.3% wt-hA3G: 96.7% E259Q-hA3G (3a14, 3a16, 3a38, 3a78)) and determined the induced mutations by comparing the sequences with the known parental virus sequence. Hypermutation profiles were made by calculating the number of GG and GA dinucleotides mutated to AG and AA, respectively, in 400bp sliding windows to the 3′ of the base under consideration. The positions of the central polypurine tract (cPPT) and 3′PPT are indicated; the overall GG-to-AG and GA-to-AA mutation rates in each sequence are indicated in the inset boxes. Colored rectangles indicate gaps in sequences.

Based on this analysis, it follows that our lower editing hA3G titrations (with low *r* values) are highly likely to have recorded hypermutation occurring at the lowest possible level. To further assess the effects of hypermutation occuring in this way, and to ensure that the hypermutation levels were not specific to the env-3′LTR region, we analyzed several near-full length proviral sequences from these lower editing hA3G titrations (Figure 3). In each case, 9–18% of all GG-motifs were mutated to AG, with mutation occurring either side of each polypyrine tract, suggesting that single editing hA3G-units can be active throughout the genome. It has been hypothesized that editing rates are highest in the regions most distal to the polypurine tracts, which are exposed as a single-stranded DNA substrate for the longest times forming a “twin gradient” of mutational burden across the genome [17], [32], [33]. Our previous study of *in vitro* and *in vivo* hypermutated sequences demonstrated that reduced levels of editing immediately downstream of the polypurine tracts were a common feature of hA3G editing although hypermutation gradients were not always evident [20], in agreement with the single editing hA3G-unit data in Figure 3.

Transient transfections of hA3G *in vitro* have shown hA3G incorporation into IVACs, but have also demonstrated that overexpressed hA3G may become packaged external to the virion core [14]. However, as only IVAC-associated hA3G has been suggested to edit nascent viral DNA [14], our estimate (based on the proportion of edited sequences) would be expected to just reflect the number of IVAC-incorporated hA3G molecules, regardless of potential hA3G overexpression. In the case non-IVAC associated hA3G contributed to editing in this experiment, even fewer hA3G-units would likely be incorporated in natural infection, underscoring that extensive hypermutation can be induced by a single or very few hA3G units.

Together, these results suggest that even a single incorporated hA3G-unit is likely to cause extensive and inactivating levels of HIV hypermutation, and that therefore, hypermutation is typically a discrete “all or nothing” phenomenon.

### *In silico* estimation of beneficial hA3G mutation rates

**Figure 4:**
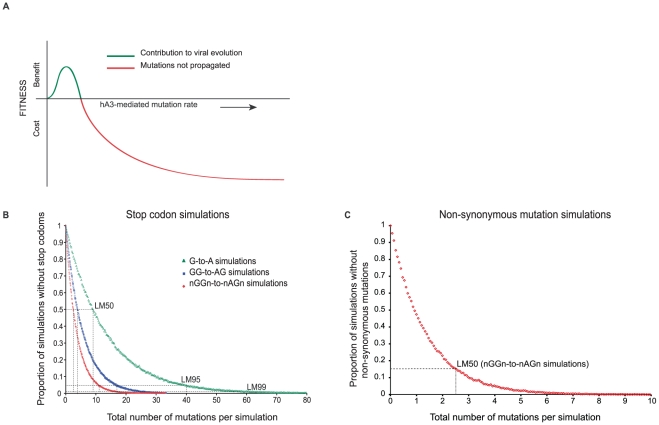
*In silico* simulation of hA3G-induced mutation in HIV(IIIB) open reading frames. A. Proposed relationship between viral fitness benefit and cost and hA3G mutation rate modified from [3]. When hA3G edits HIV-1 genomes above a particular editing rate, the mutational burden will be too high for the virus and a fitness cost is incurred, depicted in red. However, within a hypothetical window of hA3G activity, marked by the blue line, the extent of editing induced might be low enough for the virus to survive. The additional mutations may help the virus adapt faster in a fluctuating host environment and thus may be considered a viral fittness benefit. B. Each curve represents 100,000 *in silico* simulations of hA3G-induced mutation of the HIV(IIIB) open reading frames (the virus used in all *in vitro* experiments) at 100 incremental mutation rates, at which the proportion of sequences escaping in-frame stop codons was assessed. The number of mutations on the x-axis corresponds to the product of the mutation rate and the total number of available targets. Simulations using three different nucleotide targets were performed; (i) G-to-A mutation (n targets = 1362), (ii) GG-to-AG mutation (n targets = 667), and (iii) nGGn-to-nAGn mutation (n targets = 662, with 16 specific nGGn mutation rates from [20]). G-to-A simulations assumed that hA3G would recognized all Gn dinucleotide targets equally, GG-to-AG considered hA3G's preferred di-nucleotide target, while nGGn-to-nAGn simulations considered the 16 previously defined hA3G mutation rates [20] and thus more accurately mirrored the specificity with which hA3G induces mutations *in vivo*. The number of mutations necessary to induce a stop codon in 50% of viral offspring (LM50) decreased as the accuracy of the hA3G target increased from a single G to a di-nucleotide motif and lastly to a tetra-nucleotide motif. LM50 mutation rates are shown and 95% confidence intervals (CI) are smaller than the data points. C. As in B however here the proportion of simulations without non-synonomous substitutions and the associated LM50 was determined using the defined nGGn-to-nAGn mutation preferences [20].

If hA3G-induced G-to-A mutations were to increase viral diversification [2]–[7], they would have to be generated at a low, sub-lethal level (Figure 4A). To determine how low this level should be to permit neutral or potentially beneficial mutations while avoiding lethal mutations (i.e. stop codons), we determined hA3G tetranucleotide target preferences [20] and simulated editing *in silico* (Figure 4B, 4C). A previous simulation study [34] assumed, in effect, that hA3G induced a single mutation per round of replication but this is in conflict with functional studies demonstrating that hA3G moves along its single stranded DNA template while inducing multiple mutations [35]. Accordingly, we simulated here the effects of increasing hA3G-mediated mutation rates on individual viruses.

We assumed that all stop codons within HIV genes would result in non-functional virus and used the HIV(IIIB) open reading frames (Figure S1 and Figure 4) to estimate the rate at which a lethal mutation was induced in 50% of viral offspring (lethal mutation 50% - LM50) using three different nucleotide targets: G-to-A, GG-to-AG, and predefined hA3G-specific nGGn-to-nAGn tetranucleotide contexts [20].

The estimated LM50 rates depended strongly on nucleotide target specificity. Considering all G-to-A targets (assuming that hA3G recognized all Gn dinucleotide targets equally) an average of 9 targets would have to be mutated to give a 50% chance of at least one lethal mutation. However, if hA3G specificity was considered using its preferred dinucleotide GG, only 3.8 out of 667 GG targets would need to be mutated to yield a 50% chance of at least one lethal mutation. Furthermore, if specific hA3G tetranucleotide target preferences (nGGn-to-nAGn) [20] were used in the simulations, we estimate an LM50 of only 2.5 mutations, implying that the innate anti-viral hA3G protein generate stop codons very efficiently (Figure 4B). At an nGGn-to-nAGn rate of 2.5% per context (equivalent to only 11 mutations per genome), stop codons were induced in 99% of simulations (LM99).

These estimates are highly conservative as they ignore the likely harmful effects of most non-synonymous (NS, amino acid changing) mutations and possible negative effects of synonymous (S) changes on RNA secondary structure [36]–[38]. Both NS and S mutations are more frequent than stop codons (e.g. at the nGGn-to-nAGn LM50 rate, >80% of the simulations also had at least one NS mutation (Figure 4C), and about 60% had multiple). At these rates, only a few hA3G-induced mutations are needed to inactivate progeny viruses and considering the hypermutation rates observed *in vitro*, we found that the lowest hypermutation frequency detected was ∼10 fold higher than the estimated LM50 rate and over double the estimated LM99 rate (Figure 1D).

Collectively, our results suggest that even a single virion-incorporated hA3G-unit rarely, if ever, generate G-to-A mutations at sub-lethal levels but is very likely to cause extensive and inactivating levels of HIV hypermutation.

## Discussion

Here we investigate the pivotal question of whether hA3G-induced G-to-A mutation is always lethal to the virus or if it may occur at sub-lethal frequencies.

We examined whether limiting-levels of hA3G activity could result in sub-lethal mutation rates using an *in vitro* hA3G titration and sequencing experiment. The resulting *in vitro* mutation patterns and per replication cycle rates were similar to mutation levels found in *in vivo* hypermutated HIV DNA sequences implying that our experimental data reflected natural infection [20].

Second, based on the proportions of sequences carrying hypermutation in these datasets, we estimated that the maximum number of editing hA3G molecules packaged in a virion was 13 (95% CI, 6–26), which was only slightly higher than a previous biochemical estimate of 7+/−4 molecules [28]. Using our estimate, we calculated that it was highly likely that the hypermutants we observed at the lowest wt-hA3G concentrations in the titration experiments (Table 1) were caused by the incorporation of just a single hA3G-unit, and this becomes even more likely if the lower biochemical estimate is correct. As the editing observed was extensive and induced inactivating levels of G-to-A mutations, hypermutation typically seems to be an “all or nothing” phenomenon.

It has been hypothesized that a proportion of hypermutated sequences might be degraded by the cellular uracil DNA glycosylases UNG2 and/or SMUG1 and that this may contribute to the antiviral effect of hA3G [39], [40]. This hypothesis is however controversial [3] as few studies support it [41] while several have demonstrated that the absence or inhibition of UNG2 and/or SMUG1 activity neither abrogates hA3G inhibition of infection nor rescues viral cDNA accumulation in infected cells, suggesting that these enzymes are not involved in hA3G restriction of viral replication [3], [29]–[31]. Without conclusive data demonstrating UNG-mediated degradation, it is impossible to model in a realistic manner. However, we estimate that UNG-mediated degradation, if it destroyed a large proportion of the hypermutated sequences, would increase our estimation of k (the number of hA3G units in a virion). This would however not impact on our analyses of the role of hA3G in viral evolution *in vivo* as sequences that are degraded disappear and do not form part of the viral population.

Third, we simulated editing *in silico* taking viral reading frames into account, to determine how low levels of hA3G-induced G-to-A mutations should be to increase viral diversification through neutral or potentially beneficial mutations while avoiding induction of lethal mutations (i.e. stop codons). We found that due to hA3G tetranucleotide target preferences, which render it efficient at generating stop codons, only a few mutations were generally needed to inactivate progeny viruses. When we compared the estimated LM50 rate with *in vitro* hypermutation rates, we found that it was ∼10 fold less than the very lowest hypermutation frequency, suggesting that even a single hA3G-unit rarely, if ever, causes G-to-A mutations at potentially beneficial low levels.

Examining the role of hA3G in HIV evolution is an area of active research. *In vitro* studies have used reporter-genes to extrapolate the effect hA3G editing on HIV diversification [42] and the nucleoside analog RT inhibitor 2′,3′-dideoxy-3′-thia-cytidine (3TC or Lamivudine) to assess the effect of hA3G on the appearance of drug resistance mutations in lab-adapted HIV [43]. Population sequencing, which only detects polymorphisms present in >20–25% of the viral population [44]–[47], was used to identify drug-resistance mutations and as Lamivudine accumulates to different degrees in different cell lines [48]–[51] and increases intracellular dATP levels [52], which may affect RT misincorporation [13], the relevance of these studies for HIV evolution in natural infection needs further examination.

Studies of patient-derived HIV sequences either directly support our finding that hA3G is unlikely to contribute to viral diversification [13], [53] or does not contrast it [54], [55]. One report found that about 25% of rapidly diversifying sites in HIV were in sequence motifs that could be mutated by either hA3C, hA3F, hA3G or RT [54]. Another study indicated that RT misincorporation was affected by imbalances in dNTP pools, which could explain the observed bias of G-to-A mutations in HIV evolution, and found no sign of hA3F/G editing [13]. A third study of plasma virus sequences from HIV-1 infected patients that were either drug-naïve or had failed HAART demonstrated that Vif was highly polymorphic in both groups, but more so in pretreated patients [55]. One of the Vif substitutions (K22H) was further analyzed as another substitution (K22E) had previously been demonstrated to partially neutralize hA3F but not hA3G [4]. K22H was shown to partially neutralize hA3G whilst the effect on hA3F was not tested. *In vitro* culture of mutated virus in MT2 cells that express high levels of hA3F and hA3G [56] resulted in a minority of the sequences carrying sub-lethal mutations, which could be caused by either hA3F or hA3G. In contrast to hA3G, hA3C and hA3F are likely to sometimes induce sub-lethal G-to-A mutations as hA3F neutralization is dispensable for spread of HIV-1 in primary lymphocytes [56] and hA3C neutralization is not needed for viral spread in SupT1 cells, which does not express hA3F and hA3G [57]. A fourth cross-sectional study of patient-derived sequences found no evidence of an evolutionary footprint of hA3F/G [53] and studies of thousands of patient-derived sequences have found either no, or very few, hypermutated RNA sequences, suggesting that low-level hypermutation, or recombination between hypermutated and non-hypermutated viruses, very rarely occurs *in vivo* [12], [23]. Such a recombination has been found only once *in vitro* after co-transfection of 32 hypermutated and non-hypermutated proviruses and 3TC drug selection [58].

As hA3G activity has such detrimental effects on HIV, strong viral selective pressures must act to optimize Vif's interaction with hA3G. However, as variable levels of hypermutation are observed in many HIV infected patients, other selective pressures may sometimes also affect *vif* evolution. Several studies have demonstrated that CD8+ cytotoxic T-cells (CTL) can target Vif [59]–[65] and we hypothesize that these CTL responses sometimes select for Vif variants that by chance interact less efficiently with hA3G. As hypermutation frequency has been found to correlate inversely with plasma viremia in three large patient cohorts [66]–[68], but not in two smaller cohorts [69], [70], increasing hypermutation frequencies in patients through therapeutic measures is potentially beneficial.

In conclusion, our study suggests that hA3G activity is unlikely to increase HIV evolution and that hA3G-activity is highly likely to inactivate HIV-1.

## Materials and Methods

### Plasmids, cell lines, and preparation of viral stocks

pcDNA3.1 expression vectors with wild-type hA3G (wt-A3G) or non-editing E259Q mutant hA3G (E259Q-hA3G), VSV-G and the *vif*-deficient HIV-1(IIIB) (pIIIB/Δ*vif*) proviral construct have been described previously [21], [25], [71]–[73]. *Vif*-deficiency was caused by the introduction of two nonsense mutations while all other accessory genes were functional. pIIIB/Δ*vif* was furthermore modified with a G-to-A mutation at position 571 of the 5′LTR U5 region, which copies to the 3′LTR during reverse transcription, enabling discrimination of viral sequences that have passed through a replication cycle from those derived from the residual transfection cocktail. VSV-G pseudotyped Δvif-HIV-1 was produced by transfection of subconfluent monolayers of 293T cells using polyethylenimine (PEI) (Polyscience). as in [25]. The transfection efficiency of PEI is reported to be over 98% [74] and the average number of transfected plasmids per cell using similar plasmid concentrations and cell numbers is about 10^5^ plasmid molecules [75]. The pIIIB/Δvif construct, VSV-G, and varied ratios of wt-hA3G to E259Q-mutant hA3G were used (summarized in Table 1). Media were changed after 6 h and supernatants were harvested after 24 hr (hA3G titration experiment) or 48 hr (patient-derived Vif experiment); virus production was quantified by p24 Gag ELISA (Perkin Elmer), prior to storage at −80°C and use in subsequent experiments.

### Immunoblot analysis

For preparation of purified HIV-1 virion associated proteins, virus supernatant equivalent to 30 ng of p24 Gag was diluted in media, and underlain with 20% sucrose solution. Samples were centrifuged for 2 hours at 14000 rpm at 4C° and supernatants removed. Purified virions or infected 293T cells were lysed, centrifuged to remove cell debris, and prepared for loading onto SDS-PAGE gels in a 1∶1∶1 mix of 3× SDS-PAGE sample buffer (180 mM Tris, pH 6.8; 9% (w/v) SDS; 30% glycerol; bromophenol blue), DTT (in PBS, giving a final concentration of 100 mM) and lysate, and were incubated for 10 minutes at 95°C. 5–10 µl of samples were loaded into a 4% stacking gel on a 12% separating gel and run for 1 hr at 25 mA/gel at maximum voltage. Proteins were transferred from gels onto PVDF membranes (pre-soaked in methanol and running buffer (WB: 0.1% Tween20 in PBS)) at 16 V overnight; membranes were blocked in 5% milk powder in WB for at least 30 minutes, prior to incubation with primary antibody (either anti-hA3G (recognizing both wt- and E259Q-hA3G) or anti-p24^CA^ (loading control) diluted in 5% milk powder/WB) for 1 hr at room temperature. After rinsing 3 times and washing 4 times for 5′ with WB, membranes were incubated with horseradish-peroxidase conjugated secondary antibody (diluted in 5% milk powder/WB) for 40′ at room temperature, and the rinse/wash procedure was repeated. Membranes were then incubated for 1–5′ with ECL substrate before exposure to film as in [25].

### Viral infections, single-cycle infectivity, and DNA purification

TZM-bl cells (a HeLa cell line expressing HIV-1 co-receptors and a *lacZ* reporter gene under control of an HIV-1 LTR promoter) were infected with 293T cell produced VSV-G-pseudotyped Δvif-HIV-1 virions containing various ratios of wt-hA3G to E259Q-mutant hA3G (hA3G titration experiment) or Δvif-HIV-1 virions containing hA3G and patient derived Vif (patient-derived Vif experiment). After 24 hrs, supernatants were removed and cells were washed with PBS, before lysing with 200 µl lysis solution. Following transfer to microfuge tubes, debris from cell lysates was pelleted by microcentrifugation at 14,000 rpm for 10 minutes and 20 µl cell extract was then added to 100 µl Galacton-Star (reporter gene assay system for mammalian cells) substrate (Applied Biosystems Inc., CA, USA) diluted 1∶50 with reaction buffer diluent in white microplate wells. The light signal was measured every 10–15 minutes up to 2 hr after the start of the reaction on a luminometer, giving a read-out of β-galactosidase production, which is proportional to the infectivity of the infecting virus. For sequencing experiments, total DNA was extracted from infected cells using the DNeasy DNA extraction kit (Qiagen Inc, CA, USA) and digested with DpnI (New England Biolabs), a restriction endonuclease that specifically targets methylated DNA, to remove carried-over transfection mixture.

### Amplification of near-full length proviral genomes

Near-full length proviral single genomes were amplified by limiting dilution nested PCR using Advantage 2 Polymerase mix (TakaraBio/Clontech, Paris, France) and HIV-1 specific oligonucleotide primers, as described previously [20]. The product of an 8.5 kb first-round PCR from *gag*-to-3′LTR was used as a template for a second-round PCR spanning *env*-to-3′LTR (2.1 kb, 8–20 fragments per hA3G transfection condition, 87 amplicons in total)(Figure S2). For a subset of sequences, *gag*-to-*pol*, *pol*-to-*vif*, and *vif*-to-*env* fragments were amplified to derive near-full length sequences. Where possible, primers (Table S2) were designed to exclude 5′GG or 5′GA (plus-strand) or 5′CC or 5′TC (minus-strand) motifs, the preferred contexts for hA3F and hA3G activity respectively, in order to reduce the potential for bias in amplification of hypermutated viruses. Amplicons were purified using the QIAquick PCR purification kit (Qiagen Incorporated, CA, USA) and both strands were sequenced directly using Dyedeoxy Terminator sequencing (Applied Biosystems, CA, USA) on an Applied Biosystems 3730xl DNA Analyzer as previously described [20]. DNA reads were assembled and proofread using the Pregap4 and Gap4 software within the Staden package [76] (Figure S2). Sequences lacking the engineered G-to-A mutation in the 3′LTR [72] were assumed to be carried-over transfection mixture and were discarded. Sequences were screened for evidence of hA3G-mediated editing/hypermutation (defined as a mutational process in which G-to-A transitions far exceed all other mutations [19]) using the HYPERMUT software (www.hiv.lanl.gov) [77].

### MLE of the number of virion-associated hA3G-units

The proportion of sequences carrying evidence of hypermutation at each titration was used to generate a MLE of the average number of deaminating hA3G units incorporated into a progeny virion. Our analysis assumes (i) that there are a limited number of positions in a virion that can be occupied by hA3G-editing units [28]. The number of such positions, denoted *k*, is unknown, but we can use our titrations to obtain a maximum likelihood estimate of its value. Let us denote as *r_i_*, the proportion of the hA3G in transfection condition *i* that is wild-type editing (wt-hA3G), as opposed to non-editing (E259Q-hA3G); for example, from Table 1 and Table 2, in condition 5, *r*_5_ = 0.33. How likely is a virion to incorporate an editing hA3G-unit under this condition? To answer this question, we assume (ii) that the efficiency of transfection, protein expression, and virion incorporation is the same for editing and non-editing hA3G; and (iii) that there is sufficient hA3G present in each titration for all *k* slots to be occupied. Under these three assumptions, the probability that a virion incorporates one or more editing hA3G-units is simply *q_i_* = 1−(1−*r_i_*)*^k^*. If we further assume (iv) that detectable hypermutation always ensues from the incorporation of one or more editing hA3G-units, then *q_i_* is also the probability that a sequence undergoes hypermutation. As such, given a sample of *n_i_* sequences, the probability that *h_i_* of them will be hypermutants is the binomial probability:
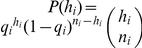
. Because *q_i_* is a function of *k* we can now write the likelihood function of *k* as 
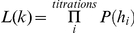
, and thereby obtain the value of *k* that is most likely to have given rise to our data, i.e., the value that maximises L(*k*). 95% confidence intervals on this estimate were obtained by assuming that twice the log likelihood ratio is χ^2^ -distributed with 1 degree of freedom, and the sensitivity of the analysis to each individual condition was assessed by jackknifing, i.e., reestimating *_k_* after removing each condition in turn. Results of the analyses are shown in Figure 1E and Figure 2, and the values of *r_i_*, *n_i_*, and *h_i_* are shown in Table 2. The approximation for the probability that an observed hypermutated sequence has arisen from the incorporation of a single hA3G-editing unit is obtained from:
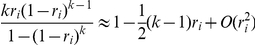



### Analyses of *in vivo* hypermutated HIV DNA

The thirty-eight near-full length HIV genomes annotated as hypermutated in the Los Alamos HIV sequence database (www.hiv.lanl.gov) at the time of this analysis and one non-annotated hypermutated sequence (EF036536) were used to estimate levels of hypermutation in HIV DNA (Table S3). EF036536 was identified by examining GenBank entries of 1725 near-full length HIV genomes. The sequences were tested by the search terms ‘stop’, ‘truncated’, ‘truncation’, ‘terminated’, ‘termination’, ‘mutated’, ‘mutation’, ‘hypermutated’, ‘hypermutation’, ‘non-functional’, and ‘nonfunctional’, and those carrying more than 4 stop codons were tested for evidence of hA3G-induced mutations as previously described [20]; analyses of sequences with fewer mutations was not possible due to noise. GG-to-AG mutation rates were estimated for each of these 39 hypermutated sequences using reference sequences generated from closely related taxa identified by NJ phylogenetic tree analyses as described previously [20]. GG-to-AG mutation rates were corrected for probable non-hA3G-mediated mutation by subtracting the mean of the GC-to-AC and GT-to-AT mutation rates in each sample from the GG-to-AG mutation rate, after adjusting for the biased nucleotide composition of the HIV genome in each case (GC and GT are seldom mutated by hA3G in single cycle *in vitro* infections [20].

### *In silico* simulations

The open reading frames of HIV-1 pIIIB, (the virus used in the *in vitro* analyses) were used in computer simulations of hA3G-induced mutation (Figure S1). Predefined nGGn-to-nAGn mutation rates and the array of defined hA3G nGGn-to-nAGn mutation preferences [20] were used to determine the probability of mutation of each nGGn context. The mutation rate required to induce at least one stop codon in open reading frames in 50% of the simulations (the Lethal Mutation 50% or LM50) was determined from 100,000 simulations of 100 incremental mutation rates in simulations of G-to-A, GG-to-AG and nGGn-to-nGAn mutations. Other thresholds such as LM95 and LM99 were also determined. The proportion of simulations without non-synonomous substitutions and the LM50 was also determined using the defined nGGn-to-nAGn mutation preferences [20].

The simulations did not account for the proposed twin gradient hypothesis for hypermutation, whereby the hA3G-induced mutational burden across individual genomes is proposed to increase from minima at the polypurine tracts (PPTs) in a plus-strand 5′-3′ direction [17], [33] as existing data are insufficient to model this effect [20], [78]. Nevertheless, since the twin gradient hypothesis predicts higher levels of mutation in the structural *pol* and *env* genes (most distal to the 3′ ends of the PPTs), we predict that at a given mutation rate, simulations modeling this effect would yield increased numbers of stop codons in these genes with respect to the simulations described; thus our estimates are conservative.

## Supporting Information

Figure S1 The HIVIIIB sequence (similar to the one used in the *in vitro* experiments) in alignment with ORFs as used in the simulations.
(PDF)

Figure S2 HIVIIIB sequence alignments from the in vitro titration experiment: 1) envLTR and 2) almost full-length provirus.
(PDF)

Table S1 Mutation summary.
(PDF)

Table S2 PCR primers.
(PDF)

Table S3 GenBank accession numbers.
(PDF)
