# Supplementary material for: APOBEC3G-Induced Hypermutation of Human Immunodeficiency Virus Type-1 Is Typically a Discrete “All or Nothing” Phenomenon
Source: PLoS Genet. 2012 Mar 22;8(3):e1002550. doi: 10.1371/journal.pgen.1002550 (PMC3310730; doi:10.1371/journal.pgen.1002550)
Supplement: Figure S1 — The HIVIIIB sequence (similar to the one used in the in vitro experiments) in alignment with ORFs as used in the simulations. (PDF) [file pgen.1002550.s001.pdf]

Supplementary figure 1.

HIVIIIB sequence from in vitro experiments in alignment with ORFs as used in the simulations.

```
>IIIB-VH12_complete_seq
--------------------------------------------------------------------------------
--------------------------------------------------------------------------------
--------------------------------------------------------------------------------
--------------------------------------------------------------------------------
--------------------------------------------------------------------------------
--------------------------------------------------------------------------------
--------------------------------------------------------------------------------
--------------------------------------------------------------------------------
--------------------------------------------------------------------------------
--------------------------------------------------------------------------------
--------------------------------------------------------------------------------
--------------------------------------------------------------------------------
--------------------------------------------------------------------------------
--------------------------------------------------------------------------------
----------------------------------------------------------------ACCCTATAGTGCAGAA
CATCCAGGGGCAAATGGTACATCAGGCCATATCACCTAGAACTTTAAATGCATGGGTAAAAGTAGTAGAAGAGAAGGCTT
TCAGCCCAGAAGTGATACCCATGTTTTCAGCATTATCAGAAGGAGCCACCCCACAAGATTTAAACACCATGCTAAACACA
GTGGGGGGACATCAAGCAGCCATGCAAATGTTAAAAGAGACCATCAATGAGGAAGCTGCAGAATGGGATAGAGTGCATCC
AGTGCATGCAGGGCCTATTGCACCAGGCCAGATGAGAGAACCAAGGGGAAGTGACATAGCAGGAACTACTAGTACCCTTC
AGGAACAAATAGGATGGATGACACATAATCCACCTATCCCAGTAGGAGAAATCTATAAAAGATGGATAATCCTGGGATTA
AATAAAATAGTAAGAATGTATAGCCCTACCAGCATTCTGGACATAAGACAAGGACCAAAGGAACCCTTTAGAGACTATGT
AGACCGATTCTATAAAACTCTAAGAGCCGAGCAAGCTTCACAAGAGGTAAAAAATTGGATGACAGAAACCTTGTTGGTCC
AAAATGCGAACCCAGATTGTAAGACTATTTTAAAAGCATTGGGACCAGGAGCGACACTAGAAGAAATGATGACAGCATGT
CAGGGAGTGGGGGGACCCGGCCATAAAGCAAGAGTTTTGGCTGAAGCAATGAGCCAAGTAACAAATCCAGCTACCATAAT
GATACAGAAAGGCAATTTTAGGAACCAAAGAAAGACTGTTAAGTGTTTCAATTGTGGCAAAGAAGGGCACATAGCCAAAA
ATTGCAGGGCCCCTAGGAAAAAGGGCTGTTGGAAATGTGGAAAGGAAGGACACCAAATGAAAGATTGTACTGAGAGACAG
GCTAATTTTTTAGGGAAGATCTGGCCTTCCCACAAGGGAAGGCCAGGGAATTTTCTTCAGAGCAGACCAGAGCCAACAGC
CCCACCAGAAGAGAGCTTCAGGTTTGGGGAAGAGACAACAACTCCCTCTCAGAAGCAGGAGCCGATAGACAAGGAACTGT
ATCCTTTAGCTTCCCTCAGATCACTCTTTGGCAGCGACCCCTCGTCACAATAAAGATAGGGGGGCAATTAAAGGAAGCTC
TATTAGATACAGGAGCAGATGATACAGTATTAGAAGAAATGAATTTGCCAGGAAGATGGAAACCAAAAATGATAGGGGGA
ATTGGAGGTTTTATCAAAGTAAGACAGTATGATCAGATACTCATAGAAATCTGCGGACATAAAGCTATAGGTACAGTATT
AGTAGGACCTACACCTGTCAACATAATTGGAAGAAATCTGTTGACTCAGATTGGCTGCACTTTAAATTTTCCCATTAGTC
CTATTGAGACTGTACCAGTAAAATTAAAGCCAGGAATGGATGGCCCAAAAGTTAAACAATGGCCATTGACAGAAGAAAAA
ATAAAAGCATTAGTAGAAATTTGTACAGAAATGGAAAAGGAAGGAAAAATTTCAAAAATTGGGCCTGAAAATCCATACAA
TACTCCAGTATTTGCCATAAAGAAAAAAGACAGTACTAAATGGAGAAAATTAGTAGATTTCAGAGAACTTAATAAGAGAA
CTCAAGATTTCTGGGAAGTTCAATTAGGAATACCACATCCTGCAGGGTTAAAACAGAAAAAATCAGTAACAGTACTGGAT
GTGGGCGATGCATATTTTTCAGTTCCCTTAGATAAAGACTTCAGGAAGTATACTGCATTTACCATACCTAGTATAAACAA
TGAGACACCAGGGATTAGATATCAGTACAATGTGCTTCCACAGGGATGGAAAGGATCACCAGCAATATTCCAGTGTAGCA
TGACAAAAATCTTAGAGCCTTTTAGAAAACAAAATCCAGACATAGTCATCTATCAATACATGGATGATTTGTATGTAGGA
TCTGACTTAGAAATAGGGCAGCATAGAACAAAAATAGAGGAACTGAGACAACATCTGTTGAGGTGGGGATTTACCACACC
AGACAAAAAACATCAGAAAGAACCTCCATTCCTTTGGATGGGTTATGAACTCCATCCTGATAAATGGACAGTACAGCCTA
TAGTGCTGCCAGAAAAGGACAGCTGGACTGTCAATGACATACAGAAATTAGTGGGAAAATTGAATTGGGCAAGTCAGATT
TATGCAGGGATTAAAGTAAGGCAATTATGTAAACTTCTTAGGGGAACCAAAGCACTAACAGAAGTAGTACCACTAACAGA
AGAAGCAGAGCTAGAACTGGCAGAAAACAGGGAGATTCTAAAAGAACCGGTACATGGAGTGTATTATGACCCATCAAAAG
ACTTAATAGCAGAAATACAGAAGCAGGGGCAAGGCCAATGGACATATCAAATTTATCAAGAGCCATTTAAAAATCTGAAA
ACAGGAAAGTATGCAAGAATGAAGGGTGCCCACACTAATGATGTGAAACAATTAACAGAGGCAGTACAAAAAATAGCCAC
AGAAAGCATAGTAATATGGGGAAAGACTCCTAAATTTAAATTACCCATACAAAAGGAAACATGGGAAGCATGGTGGACAG
AGTATTGGCAAGCCACCTGGATTCCTGAGTGGGAGTTTGTCAATACCCCTCCCTTAGTGAAGTTATGGTACCAGTTAGAG
AAAGAACCCATAATAGGAGCAGAAACTTTCTATGTAGATGGGGCAGCCAATAGGGAAACTAAATTAGGAAAAGCAGGATA
TGTAACTGACAGAGGAAGACAAAAAGTTGTCCCCCTAACGGACACAACAAATCAGAAGACTGAGTTACAAGCAATTCATC
TAGCTTTGCAGGATTCGGGATTAGAAGTAAACATAGTGACAGACTCACAATATGCATTGGGAATCATTCAAGCACAACCA
GATAAGAGTGAATCAGAGTTAGTCAGTCAAATAATAGAGCAGTTAATAAAAAAGGAAAAAGTCTACCTGGCATGGGTACC
AGCACACAAAGGAATTGGAGGAAATGAACAAGTAGATAAATTGGTCAGTGCTGGAATCAGGAAAGTACTATTTTTAGATG
GAATAGATAAGGCCCAAGAAGAACATGAGAAATATCACAGTAATTGGAGAGCAATGGCTAGTGATTTTAACCTACCACCT
GTAGTAGCAAAAGAAATAGTAGCCAGCTGTGATAAATGTCAGCTAAAAGGGGAAGCCATGCATGGACAAGTAGACTGTAG
CCCAGGAATATGGCAGCTAGATTGTACACATTTAGAAGGAAAAGTTATCTTGGTAGCAGTTCATGTAGCCAGTGGATATA
TAGAAGCAGAAGTAATTCCAGCAGAGACAGGGCAAGAAACAGCATACTTCCTCTTAAAATTAGCAGGAAGATGGCCAGTA
AAAACAGTACATACAGACAATGGCAGCAATTTCACCAGTACTACAGTTAAGGCCGCCTGTTGGTGGGCGGGGATCAAGCA
GGAATTTGGCATTCCCTACAATCCCCAAAGTCAAGGAGTAATAGAATCTATGAATAAAGAATTAAAGAAAATTATAGGAC
```

AGGTAAGAGATCAGGCTGAACATCTTAAGACAGCAGTACAAATGGCAGTATTCATCCACAATTTTAAAAGAAAAGGGGGG
ATTGGGGGGTACAGTGCAGGGGAAAGAATAGTAGACATAATAGCAACAGACATACAAACTAAAGAATTACAAAAACAAAT
TACAAAAATTCAAAATTTTCGGGTTTATTACAGGGACAGCAGAGATCCAGTTTGGAAAGGACCAGCAAAGCTCCTCTGGA
AAGGTGAAGGGGCAGTAGTAATACAAGATAATAGTGACATAAAAGTAGTGCCAAGAAGAAAAGCAAAGATCATCAGGGAT
TATGGAAAACAGATGGCAGGTGATGATTGTGTGGCAAGTAGACAGGATGAGGATTAACACATGGAAAAGATTAGTATAAT
AGCATATGTATATTTCAAGGAAAGCTAAGGACTGGTTTTATAGACATCACTATGAAAGTACTAATCCAAAAATAAGTTCA
GAAGTACACATCCCACTAGGGGATGCTAAATTAGTAATAACAACATATTGGGGTCTGCATACAGGAGAAAGAGACTGGCA
TTTGGGTCAGGGAGTCTCCATAGAATGGAGGAAAAAGAGATATAGCACACAAGTAGACCCTGACCTAGCAGACCAACTAA
TTCATCTGCACTATTTTGATTGTTTTTCAGAATCTGCTATAAGAAATACCATATTAGGACGTATAGTTAGTCCTAGGTGT
GAATATCAAGCAGGACATAACAAGGTAGGATCTCTACAGTACTTGGCACTAGCAGCATTAATAAAACCAAAACAGATAAA
GCCACCTTTGCCTAGTGTTAGGAAACTGACAGAGGACAGATGGAACAAGCCCCAGAAGACCAAGGGCCACAGAGGGAGCC
ATACAATGAATGGACACTAGAGCTTTTAGAGGAACTTAAGAGTGAAGCTGTTAGACATTTTCCTAGGATATGGCTCCATA
ACTTAGGACAACATATCTATGAAACTTACGGGGATACTTGGGCAGGAGTGGAAGCCATAATAAGAATTCTGCAACAACTG
CTGTTTATCCATTTCAGAATTGGGTGTCGACATAGCAGAATAGGCGTTACTCGACAGAGGAGAGCAAGAAATGGAGCCAG
TAGATCCTAGACTAGAGCCCTGGAAGCATCCAGGAAGTCAGCCTAAAACTGCTTGTACCAATTGCTATTGTAAAAAGTGT
TGCTTTCATTGCCAAGTTTGTTTCATAACAAAAGCCTTAGGCATCTCCTATGGCAGGAAGAAGCGGAGACAGCGACGAAG
ACCTCCTCAAGGCAGTCAGACTCATCAAGTTTCTCTATCAAAGCAGTAAGTAATACATGTAATGCAACCTATACAAATAG
CAATAGTAGCATTAGTAGTAGCAATAATAATAGCAATAGTTGTGTGGTCCATAGTAATCATAGAATATAGGAAAATATTA
AGACAAAGAAAAATAGACAGGTTAATTGATAGACTAATAGAAAGAGCAGAAGACAGTGGCAATGAGAGTGAAGGAGAAAT
ATCAGCACTTGTGGAGATGGGGGTGGAGATGGGGCACCATGCTCCTTGGGATGTTGATGATCTGTAGTGCTACAGAAAAA
TTGTGGGTCACAGTCTATTATGGGGTACCTGTGTGGAAGGAAGCAACCACCACTCTATTTTGTGCATCAGATGCTAAAGC
ATATGATACAGAGGTACATAATGTTTGGGCCACACATGCCTGTGTACCCACAGACCCCAACCCACAAGAAGTAGTATTGG
TAAATGTGACAGAAAATTTTAACATGTGGAAAAATGACATGGTAGAACAGATGCATGAGGATATAATCAGTTTATGGGAT
CAAAGCCTAAAGCCATGTGTAAAATTAACCCCACTCTGTGTTAGTTTAAAGTGCACTGATTTGAAGAATGATACTAATAC
CAATAGTAGTAGCGGGAGAATGATAATGGAGAAAGGAGAGATAAAAAACTGCTCTTTCAATATCAGCACAAGCATAAGAG
GTAAGGTGCAGAAAGAATATGCATTTTTTTATAAACTTGATATAATACCAATAGATAATGATACTACCAGCTATACGTTG
ACAAGTTGTAACACCTCAGTCATTACACAGGCCTGTCCAAAGGTATCCTTTGAGCCAATTCCCATACATTATTGTGCCCC
GGCTGGTTTTGCGATTCTAAAATGTAATAATAAGACGTTCAATGGAACAGGACCATGTACAAATGTCAGCACAGTACAAT
GTACACATGGAATTAGGCCAGTAGTATCAACTCAACTGCTGTTAAATGGCAGTCTAGCAGAAGAAGAGGTAGTAATTAGA
TCTGTCAATTTCACGGACAATGCTAAAACCATAATAGTACAGCTGAACACATCTGTAGAAATTAATTGTACAAGACCCAA
CAACAATACAAGAAAAAAAATCCGTATCCAGAGGGGACCAGGGAGAGCATTTGTTACAATAGGAAAAATAGGAAATATGA
GACAAGCACATTGTAACATTAGTAGAGCAAAATGGAATGCCACTTTAAAACAGATAGCTAGCAAATTAAGAGAACAATTT
GGAAATAATAAAACAATAATCTTTAAGCAATCCTCAGGAGGGGACCCAGAAATTGTAACGCACAGTTTTAATTGTGGAGG
GGAATTTTTCTACTGTAATTCAACACAACTGTTTAATAGTACTTGGTTTAATAGTACTTGGAGTACTGAAGGGTCAAATA
ACACTGAAGGAAGTGACACAATCACACTCCCATGCAGAATAAAACAATTTATAAACATGTGGCAGGAAGTAGGAAAAGCA
ATGTATGCCCCTCCCATCAGCGGACAAATTAGATGTTCATCAAATATTACAGGGCTGCTATTAACAAGAGATGGTGGTAA
TAACAACAATGGGTCCGAGATCTTCAGACCTGGAGGAGGAGATATGAGGGACAATTGGAGAAGTGAATTATATAAATATA
AAGTAGTAAAAATTGAACCATTAGGAGTAGCACCCACCAAGGCAAAGAGAAGAGTGGTGCAGAGAGAAAAAAGAGCAGTG
GGAATAGGAGCTTTGTTCCTTGGGTTCTTGGGAGCAGCAGGAAGCACTATGGGCGCAGCGTCAATGACGCTGACGGTACA
GGCCAGACAATTATTGTCTGGTATAGTGCAGCAGCAGAACAATTTGCTGAGGGCTATTGAGGCGCAACAGCATCTGTTGC
AACTCACAGTCTGGGGCATCAAGCAGCTCCAGGCAAGAATCCTGGCTGTGGAAAGATACCTAAAGGATCAACAGCTCCTG
GGGATTTGGGGTTGCTCTGGAAAACTCATTTGCACCACTGCTGTGCCTTGGAATGCTAGTTGGAGTAATAAATCTCTGGA
ACAGATTTGGAATCACACGACCTGGATGGAGTGGGACAGAGAAATTAACAATTACACAAGCTTAATACACTCCTTAATTG
AAGAATCGCAAAACCAGCAAGAAAAGAATGAACAAGAATTATTGGAATTAGATAAATGGGCAAGTTTGTGGAATTGGTTT
AACATAACAAATTGGCTGTGGTATATAAAATTATTCATAATGATAGTAGGAGGCTTGGTAGGTTTAAGAATAGTTTTTGC
TGTACTTTCTGTAGTGAATAGAGTTAGGCAGGGATATTCACCATTATCGTTTCAGACCCACCTCCCAATCCCGAGGGGAC
CCGACAGGCCCGAAGGAATAGAAGAAGAAGGTGGAGAGAGAGACAGAGACAGATCCATTCGATTAGTGAACGGATCCTTA
GCACTTATCTGGGACGATCTGCGGAGCCTGTGCCTCTTCAGCTACCACCGCTTGAGAGACTTACTCTTGATTGTAACGAG
GATTGTGGAACTTCTGGGACGCAGGGGGTGGGAAGCCCTCAAATATTGGTGGAATCTCCTACAATATTGGAGTCAGGAGC
TAAAGAATAGTGCTGTTAGCTTGCTCAATGCCACAGCTATAGCAGTAGCTGAGGGGACAGATAGGGTTATAGAAGTAGTA
CAAGAAGCTTATAGAGCTATTCGCCACATACCTAGAAGAATAAGACAGGGCTTGGAAAGGATTTTGCTATAAGATGGGTG
GCAAGTGGTCAAAAAGTAGTGTGGTTGGATGGCCTGCTGTAAGGGAAAGAATGAGACGAGCTGAGCCAGCAGCAGATGGG
GTGGGAGCAGCATCTCGAGACCTAGAAAAACATGGAGCAATCACAAGTAGCAACACAGCAGCTAACAATGCTGCTTGTGC
CTGGCTAGAAGCACAAGAGGAGGAGAAGGTGGGTTTTCCAGTCACACCTCAGGTACCTTTAAGACCAATGACTTACAAGG
CAGCTGTAGATCTTAGCCACTTTTTAAAAGAAAAGGGGGGACTGGAAGGGCTAATTCACTCCCAACGAAGACAAGATATC
CTTGATCTGTGGATCTACCACACACAAGGCTACTTCCCTGATTGGCAGAACTACACACCAGGACCAGGGATCAGATATCC
ACTGACCTTTGGATGGCGCTACAAGCTAGTACCAGTTGAGCCAGAGAAGTTAGAAGAAGCCAACAAAGGAGAGAACACCA
GCTTGTTACACCCTGTGAGCCTGCATGGAATGGATGACCCGGAGAGAGAAGTGTTAGAGTGGAGGTTTGACAGCCGCCTA
GCATTTCATCACGTGGCCCGAGAGCTGCATCCGGAGTACTTCAAGAACTGCTGATATCGAGCTTGCTACAAGGGACTTTC
CGCTGGGGACTTTCCAGGGAGGCGTGGCCTGGGCGGGACTGGGGAGTGGCGAGCCCTCAGATCCTGCATATAAGCAGCTG
CTTTTTGCCTGTACTGGGTCTCTCTGGTTAGACCAGATCTGAGCCTGGGAGCTCTCTGGCTAACTAGGGAACCCACTGCT
TAAGCCTCAATAAAGCTTGCCTTGAGTGCTTCAAGTAGTGTGTGCCCATCTGTTGTGTGACTCTGGTAACTAGAGATCCC
TCA

>IIIB-VH12cons_gag


```
--------------------------------------------------------------------------------
--------------------------------------------------------------------------------
--------------------------------------------------------------------------------
--------------------------------------------------------------------------------
--------------------------------------------------------------------------------
--------------------------------------------------------------------------------
--------------------------------------------------------------------------------
--------------------------------------------------------------------------------
--------------------------------------------------------------------------------
--------------------------------------------------------------------------------
--------------------------------------------------------------------------------
--------------------------------------------------------------------------------
----------------------------------------------------------------ACCCTATAGTGCAGAA
CATCCAGGGGCAAATGGTACATCAGGCCATATCACCTAGAACTTTAAATGCATGGGTAAAAGTAGTAGAAGAGAAGGCTT
TCAGCCCAGAAGTGATACCCATGTTTTCAGCATTATCAGAAGGAGCCACCCCACAAGATTTAAACACCATGCTAAACACA
GTGGGGGGACATCAAGCAGCCATGCAAATGTTAAAAGAGACCATCAATGAGGAAGCTGCAGAATGGGATAGAGTGCATCC
AGTGCATGCAGGGCCTATTGCACCAGGCCAGATGAGAGAACCAAGGGGAAGTGACATAGCAGGAACTACTAGTACCCTTC
AGGAACAAATAGGATGGATGACACATAATCCACCTATCCCAGTAGGAGAAATCTATAAAAGATGGATAATCCTGGGATTA
AATAAAATAGTAAGAATGTATAGCCCTACCAGCATTCTGGACATAAGACAAGGACCAAAGGAACCCTTTAGAGACTATGT
AGACCGATTCTATAAAACTCTAAGAGCCGAGCAAGCTTCACAAGAGGTAAAAAATTGGATGACAGAAACCTTGTTGGTCC
AAAATGCGAACCCAGATTGTAAGACTATTTTAAAAGCATTGGGACCAGGAGCGACACTAGAAGAAATGATGACAGCATGT
CAGGGAGTGGGGGGACCCGGCCATAAAGCAAGAGTTTTGGCTGAAGCAATGAGCCAAGTAACAAATCCAGCTACCATAAT
GATACAGAAAGGCAATTTTAGGAACCAAAGAAAGACTGTTAAGTGTTTCAATTGTGGCAAAGAAGGGCACATAGCCAAAA
ATTGCAGGGCCCCTAGGAAAAAGGGCTGTTGGAAATGTGGAAAGGAAGGACACCAAATGAAAGATTGTACTGAGAGACAG
GCTAATTTTTTAGGGAAGATCTGGCCTTCCCACAAGGGAAGGCCAGGGAATTTTCTTCAGAGCAGACCAGAGCCAACAGC
CCCACCAGAAGAGAGCTTCAGGTTTGGGGAAGAGACAACAACTCCCTCTCAGAAGCAGGAGCCGATAGACAAGGAACTGT
ATCCTTTAGCTTCCCTCAGATCACTCTTTGGCAGCGACCCCTCGTCACAATAA---------------------------
--------------------------------------------------------------------------------
--------------------------------------------------------------------------------
--------------------------------------------------------------------------------
--------------------------------------------------------------------------------
--------------------------------------------------------------------------------
--------------------------------------------------------------------------------
--------------------------------------------------------------------------------
--------------------------------------------------------------------------------
--------------------------------------------------------------------------------
--------------------------------------------------------------------------------
--------------------------------------------------------------------------------
--------------------------------------------------------------------------------
--------------------------------------------------------------------------------
--------------------------------------------------------------------------------
--------------------------------------------------------------------------------
--------------------------------------------------------------------------------
--------------------------------------------------------------------------------
--------------------------------------------------------------------------------
--------------------------------------------------------------------------------
--------------------------------------------------------------------------------
--------------------------------------------------------------------------------
--------------------------------------------------------------------------------
--------------------------------------------------------------------------------
--------------------------------------------------------------------------------
--------------------------------------------------------------------------------
--------------------------------------------------------------------------------
--------------------------------------------------------------------------------
--------------------------------------------------------------------------------
--------------------------------------------------------------------------------
--------------------------------------------------------------------------------
--------------------------------------------------------------------------------
--------------------------------------------------------------------------------
--------------------------------------------------------------------------------
--------------------------------------------------------------------------------
--------------------------------------------------------------------------------
--------------------------------------------------------------------------------
--------------------------------------------------------------------------------
--------------------------------------------------------------------------------
--------------------------------------------------------------------------------
```


>IIIB-VH12cons_pol

-----TTTTTTAGGGAAGATCTGGCCTTCCCACAAGGGAAGGCCAGGGAATTTCTTCAGAGCAGACCAGAGCCAACAGC
CCCACCAGAAGAGAGCTTCAGGTTTGGGGAAGAGACAACAACTCCCTCTCAGAAGCAGGAGCCGATAGACAAGGAACTGT
ATCCTTTAGCTTCCCTCAGATCACTCTTTGGCAGCGACCCCTCGTCACAATAAAGATAGGGGGGCAATTAAAGGAAGCTC
TATTAGATACAGGAGCAGATGATACAGTATTAGAAGAAATGAATTTGCCAGGAAGATGGAAACCAAAAATGATAGGGGGA
ATTGGAGGTTTTATCAAAGTAAGACAGTATGATCAGATACTCATAGAAATCTGCGGACATAAAGCTATAGGTACAGTATT
AGTAGGACCTACACCTGTCAACATAATTGGAAGAAATCTGTTGACTCAGATTGGCTGCACTTTAAATTTTCCCATTAGTC
CTATTGAGACTGTACCAGTAAAATTAAAGCCAGGAATGGATGGCCCAAAAGTTAAACAATGGCCATTGACAGAAGAAAAA
ATAAAAGCATTAGTAGAAATTTGTACAGAAATGGAAAAGGAAGGAAAAATTTCAAAAATTGGGCCTGAAAATCCATACAA
TACTCCAGTATTTGCCATAAAGAAAAAAGACAGTACTAAATGGAGAAAATTAGTAGATTTCAGAGAACTTAATAAGAGAA
CTCAAGATTTCTGGGAAGTTCAATTAGGAATACCACATCCTGCAGGGTTAAAACAGAAAAAATCAGTAACAGTACTGGAT
GTGGGCGATGCATATTTTTCAGTTCCCTTAGATAAAGACTTCAGGAAGTATACTGCATTTACCATACCTAGTATAAACAA
TGAGACACCAGGGATTAGATATCAGTACAATGTGCTTCCACAGGGATGGAAAGGATCACCAGCAATATTCCAGTGTAGCA
TGACAAAAATCTTAGAGCCTTTTAGAAAACAAAATCCAGACATAGTCATCTATCAATACATGGATGATTTGTATGTAGGA
TCTGACTTAGAAATAGGGCAGCATAGAACAAAAATAGAGGAACTGAGACAACATCTGTTGAGGTGGGGATTTACCACACC
AGACAAAAAACATCAGAAAGAACCTCCATTCCTTTGGATGGGTTATGAACTCCATCCTGATAAATGGACAGTACAGCCTA
TAGTGCTGCCAGAAAAGGACAGCTGGACTGTCAATGACATACAGAAATTAGTGGGAAAATTGAATTGGGCAAGTCAGATT
TATGCAGGGATTAAAGTAAGGCAATTATGTAAACTTCTTAGGGGAACCAAAGCACTAACAGAAGTAGTACCACTAACAGA
AGAAGCAGAGCTAGAACTGGCAGAAAACAGGGAGATTCTAAAAGAACCGGTACATGGAGTGTATTATGACCCATCAAAAG
ACTTAATAGCAGAAATACAGAAGCAGGGGCAAGGCCAATGGACATATCAAATTTATCAAGAGCCATTTAAAAATCTGAAA
ACAGGAAAGTATGCAAGAATGAAGGGTGCCCACACTAATGATGTGAAACAATTAACAGAGGCAGTACAAAAAATAGCCAC
AGAAAGCATAGTAATATGGGGAAAGACTCCTAAATTTAAATTACCCATACAAAAGGAAACATGGGAAGCATGGTGGACAG
AGTATTGGCAAGCCACCTGGATTCCTGAGTGGGAGTTTGTCAATACCCCTCCCTTAGTGAAGTTATGGTACCAGTTAGAG
AAAGAACCCATAATAGGAGCAGAAACTTTCTATGTAGATGGGGCAGCCAATAGGGAAACTAAATTAGGAAAAGCAGGATA
TGTAACTGACAGAGGAAGACAAAAAGTTGTCCCCCTAACGGACACAACAAATCAGAAGACTGAGTTACAAGCAATTCATC
TAGCTTTGCAGGATTCGGGATTAGAAGTAAACATAGTGACAGACTCACAATATGCATTGGGAATCATTCAAGCACAACCA
GATAAGAGTGAATCAGAGTTAGTCAGTCAAATAATAGAGCAGTTAATAAAAAAGGAAAAAGTCTACCTGGCATGGGTACC
AGCACACAAAGGAATTGGAGGAAATGAACAAGTAGATAAATTGGTCAGTGCTGGAATCAGGAAAGTACTATTTTTAGATG
GAATAGATAAGGCCCAAGAAGAACATGAGAAATATCACAGTAATTGGAGAGCAATGGCTAGTGATTTTAACCTACCACCT
GTAGTAGCAAAAGAAATAGTAGCCAGCTGTGATAAATGTCAGCTAAAAGGGGAAGCCATGCATGGACAAGTAGACTGTAG
CCCAGGAATATGGCAGCTAGATTGTACACATTTAGAAGGAAAAGTTATCTTGGTAGCAGTTCATGTAGCCAGTGGATATA
TAGAAGCAGAAGTAATTCCAGCAGAGACAGGGCAAGAAACAGCATACTTCCTCTTAAAATTAGCAGGAAGATGGCCAGTA
AAAACAGTACATACAGACAATGGCAGCAATTTCACCAGTACTACAGTTAAGGCCGCCTGTTGGTGGGCGGGGATCAAGCA
GGAATTTGGCATTCCCTACAATCCCCAAAGTCAAGGAGTAATAGAATCTATGAATAAAGAATTAAAGAAAATTATAGGAC
AGGTAAGAGATCAGGCTGAACATCTTAAGACAGCAGTACAAATGGCAGTATTCATCCACAATTTTAAAAGAAAAGGGGGG
ATTGGGGGGTACAGTGCAGGGGAAAGAATAGTAGACATAATAGCAACAGACATACAAACTAAAGAATTACAAAAACAAAT
TACAAAAATTCAAAATTTTCGGGTTTATTACAGGGACAGCAGAGATCCAGTTTGGAAAGGACCAGCAAAGCTCCTCTGGA
AAGGTGAAGGGGCAGTAGTAATACAAGATAATAGTGACATAAAAGTAGTGCCAAGAAGAAAAGCAAAGATCATCAGGGAT
TATGGAAAACAGATGGCAGGTGATGATTGTGTGGCAAGTAGACAGGATGAGGATTAA-----------------------
----


```
--------------------------------------------------------------------------------
--------------------------------------------------------------------------------
--------------------------------------------------------------------------------
--------------------------------------------------------------------------------
--------------------------------------------------------------------------------
--------------------------------------------------------------------------------
--------------------------------------------------------------------------------
--------------------------------------------------------------------------------
--------------------------------------------------------------------------------
--------------------------------------------------------------------------------
--------------------------------------------------------------------------------
--------------------------------------------------------------------------------
--------------------------------------------------------------------------------
--------------------------------------------------------------------------------
--------------------------------------------------------------------------------
--------------------------------------------------------------------------------
--------------------------------------------------------------------------------
--------------------------------------------------------------------------------
--------------------------------------------------------------------------------
--------------------------------------------------------------------------------
--------------------------------------------------------------------------------
--------------------------------------------------------------------------------
--------------------------------------------------------------------------------
--------------------------------------------------------------------------------
--------------------------------------------------------------------------------
--------------------------------------------------------------------------------
--------------------------------------------------------------------------------
--------------------------------------------------------------------------------
--------------------------------------------------------------------------------
--------------------------------------------------------------------------------
--------------------------------------------------------------------------------
--------------------------------------------------------------------------------
--------------------------------------------------------------------------------
--------------------------------------------------------------------------------
--------------------------------------------------------------------------------
--------------------------------------------------------------------------------
--------------------------------------------------------------------------------
--------------------------------------------------------------------------------
--------------------------------------------------------------------------------
--------------------------------------------------------------------------------
--------------------------------------------------------------------------------
--------------------------------------------------------------------------------
--------------------------------------------------------------------------------
--------------------------------------------------------------------------------
---
>IIIB-VH12cons_vif
--------------------------------------------------------------------------------
--------------------------------------------------------------------------------
--------------------------------------------------------------------------------
--------------------------------------------------------------------------------
--------------------------------------------------------------------------------
--------------------------------------------------------------------------------
--------------------------------------------------------------------------------
--------------------------------------------------------------------------------
--------------------------------------------------------------------------------
--------------------------------------------------------------------------------
--------------------------------------------------------------------------------
--------------------------------------------------------------------------------
--------------------------------------------------------------------------------
--------------------------------------------------------------------------------
--------------------------------------------------------------------------------
--------------------------------------------------------------------------------
--------------------------------------------------------------------------------
--------------------------------------------------------------------------------
--------------------------------------------------------------------------------
--------------------------------------------------------------------------------
```

```
--------------------------------------------------------------------------------
--------------------------------------------------------------------------------
--------------------------------------------------------------------------------
--------------------------------------------------------------------------------
--------------------------------------------------------------------------------
--------------------------------------------------------------------------------
--------------------------------------------------------------------------------
--------------------------------------------------------------------------------
--------------------------------------------------------------------------------
--------------------------------------------------------------------------------
--------------------------------------------------------------------------------
--------------------------------------------------------------------------------
--------------------------------------------------------------------------------
--------------------------------------------------------------------------------
--------------------------------------------------------------------------------
--------------------------------------------------------------------------------
--------------------------------------------------------------------------------
--------------------------------------------------------------------------------
--------------------------------------------------------------------------------
--------------------------------------------------------------------------------
--------------------------------------------------------------------------------
--------------------------------------------------------------------------------
--------------------------------------------------------------------------------
--------------------------------------------------------------------------------
--------------------------------------------------------------------------------
--------------------------------------------------------------------------------
--------------------------------------------------------------------------------
--------------------------------------------------------------------------------
--------------------------------------------------------------------------------
--------------------------------------------------------------------------------
--------------------------------------------------------------------------------
--------------------------------------------------------------------------------
--------------------------------------------------------------------------------
--------------------------------------------------------------------------------
--------------------------------------------------------------------------------
--------------------------------------------------------------------------------
--------------------------------------------------------------------------------
--------------------------------------------------------------------------------
--------------------------------------------------------------------------------
--------------------------------------------------------------------------------
--------------------------------------------------------------------------------
--------------------------------------------------------------------------------
--------------------------------------------------------------------------------
-ATGGAAAACAGATGGCAGGTGATGATTGTGTGGCAAGTAGACAGGATGAGGATTAACACATGGAAAAGATTAGTATAAT
AGCATATGTATATTTCAAGGAAAGCTAAGGACTGGTTTTATAGACATCACTATGAAAGTACTAATCCAAAAATAAGTTCA
GAAGTACACATCCCACTAGGGGATGCTAAATTAGTAATAACAACATATTGGGGTCTGCATACAGGAGAAAGAGACTGGCA
TTTGGGTCAGGGAGTCTCCATAGAATGGAGGAAAAAGAGATATAGCACACAAGTAGACCCTGACCTAGCAGACCAACTAA
TTCATCTGCACTATTTTGATTGTTTTTCAGAATCTGCTATAAGAAATACCATATTAGGACGTATAGTTAGTCCTAGGTGT
GAATATCAAGCAGGACATAACAAGGTAGGATCTCTACAGTACTTGGCACTAGCAGCATTAATAAAACCAAAACAGATAAA
GCCACCTTTGCCTAGTGTTAGGAAACTGACAGAGGACAGATGGAACAAGCCCCAGAAGACCAAGGGCCACAGAGGGAGCC
ATACAATGAATGGACACTAG------------------------------------------------------------
--------------------------------------------------------------------------------
--------------------------------------------------------------------------------
--------------------------------------------------------------------------------
--------------------------------------------------------------------------------
--------------------------------------------------------------------------------
--------------------------------------------------------------------------------
--------------------------------------------------------------------------------
--------------------------------------------------------------------------------
--------------------------------------------------------------------------------
--------------------------------------------------------------------------------
--------------------------------------------------------------------------------
--------------------------------------------------------------------------------
--------------------------------------------------------------------------------
--------------------------------------------------------------------------------
--------------------------------------------------------------------------------
```

```
--------------------------------------------------------------------------------
--------------------------------------------------------------------------------
--------------------------------------------------------------------------------
--------------------------------------------------------------------------------
--------------------------------------------------------------------------------
--------------------------------------------------------------------------------
--------------------------------------------------------------------------------
--------------------------------------------------------------------------------
--------------------------------------------------------------------------------
--------------------------------------------------------------------------------
--------------------------------------------------------------------------------
--------------------------------------------------------------------------------
--------------------------------------------------------------------------------
--------------------------------------------------------------------------------
--------------------------------------------------------------------------------
--------------------------------------------------------------------------------
--------------------------------------------------------------------------------
--------------------------------------------------------------------------------
--------------------------------------------------------------------------------
--------------------------------------------------------------------------------
--------------------------------------------------------------------------------
--------------------------------------------------------------------------------
--------------------------------------------------------------------------------
--------------------------------------------------------------------------------
--------------------------------------------------------------------------------
--------------------------------------------------------------------------------
--------------------------------------------------------------------------------
--------------------------------------------------------------------------------
--------------------------------------------------------------------------------
--------------------------------------------------------------------------------
--------------------------------------------------------------------------------
--------------------------------------------------------------------------------
--------------------------------------------------------------------------------
--------------------------------------------------------------------------------
--------------------------------------------------------------------------------
---
>IIIB-VH12cons_vpr
--------------------------------------------------------------------------------
--------------------------------------------------------------------------------
--------------------------------------------------------------------------------
--------------------------------------------------------------------------------
--------------------------------------------------------------------------------
--------------------------------------------------------------------------------
--------------------------------------------------------------------------------
--------------------------------------------------------------------------------
--------------------------------------------------------------------------------
--------------------------------------------------------------------------------
--------------------------------------------------------------------------------
--------------------------------------------------------------------------------
--------------------------------------------------------------------------------
--------------------------------------------------------------------------------
--------------------------------------------------------------------------------
--------------------------------------------------------------------------------
--------------------------------------------------------------------------------
--------------------------------------------------------------------------------
--------------------------------------------------------------------------------
--------------------------------------------------------------------------------
--------------------------------------------------------------------------------
--------------------------------------------------------------------------------
--------------------------------------------------------------------------------
--------------------------------------------------------------------------------
--------------------------------------------------------------------------------
--------------------------------------------------------------------------------
--------------------------------------------------------------------------------
--------------------------------------------------------------------------------
--------------------------------------------------------------------------------
```


---------------------------------------ATGGAACAAGCCCCAGAAGACCAAGGGCCACAGAGGGAGCC
ATACAATGAATGGACACTAGAGCTTTTAGAGGAACTTAAGAGTGAAGCTGTTAGACATTTTCCTAGGATATGGCTCCATA
ACTTAGGACAACATATCTATGAAACTTACGGGGATACTTGGGCAGGAGTGGAAGCCATAATAAGAATTCTGCAACAACTG
CTGTTTATCCATTTCAGAATTGGGTGTCGACATAGCAGAATAGGCGTTACTCGACAGAGGAGAGCAAGAAATGGAGCCAG
TAGATCCTAG--


```
--------------------------------------------------------------------------------
--------------------------------------------------------------------------------
--------------------------------------------------------------------------------
--------------------------------------------------------------------------------
--------------------------------------------------------------------------------
--------------------------------------------------------------------------------
--------------------------------------------------------------------------------
--------------------------------------------------------------------------------
--------------------------------------------------------------------------------
--------------------------------------------------------------------------------
--------------------------------------------------------------------------------
--------------------------------------------------------------------------------
--------------------------------------------------------------------------------
--------------------------------------------------------------------------------
--------------------------------------------------------------------------------
--------------------------------------------------------------------------------
--------------------------------------------------------------------------------
--------------------------------------------------------------------------------
--------------------------------------------------------------------------------
--------------------------------------------------------------------------------
--------------------------------------------------------------------------------
--------------------------------------------------------------------------------
--------------------------------------------------------------------------------
--------------------------------------------------------------------------------
--------------------------------------------------------------------------------
--------------------------------------------------------------------------------
---
>IIIB-VH12cons_Tatexon1
--------------------------------------------------------------------------------
--------------------------------------------------------------------------------
--------------------------------------------------------------------------------
--------------------------------------------------------------------------------
--------------------------------------------------------------------------------
--------------------------------------------------------------------------------
--------------------------------------------------------------------------------
--------------------------------------------------------------------------------
--------------------------------------------------------------------------------
--------------------------------------------------------------------------------
--------------------------------------------------------------------------------
--------------------------------------------------------------------------------
--------------------------------------------------------------------------------
--------------------------------------------------------------------------------
--------------------------------------------------------------------------------
--------------------------------------------------------------------------------
--------------------------------------------------------------------------------
--------------------------------------------------------------------------------
--------------------------------------------------------------------------------
--------------------------------------------------------------------------------
--------------------------------------------------------------------------------
--------------------------------------------------------------------------------
--------------------------------------------------------------------------------
--------------------------------------------------------------------------------
--------------------------------------------------------------------------------
--------------------------------------------------------------------------------
--------------------------------------------------------------------------------
--------------------------------------------------------------------------------
--------------------------------------------------------------------------------
--------------------------------------------------------------------------------
--------------------------------------------------------------------------------
--------------------------------------------------------------------------------
--------------------------------------------------------------------------------
--------------------------------------------------------------------------------
--------------------------------------------------------------------------------
--------------------------------------------------------------------------------
--------------------------------------------------------------------------------
--------------------------------------------------------------------------------
```


--ATGGAGCCAG
TAGATCCTAGACTAGAGCCCTGGAAGCATCCAGGAAGTCAGCCTAAAACTGCTTGTACCAATTGCTATTGTAAAAAGTGT
TGCTTTCATTGCCAAGTTTGTTTCATAACAAAAGCCTTAGGCATCTCCTATGGCAGGAAGAAGCGGAGACAGCGACGAAG
ACCTCCTCAAGGCAGTCAGACTCATCAAGTTTCTCTATCAAAGCA-----------------------------------
----


```
--------------------------------------------------------------------------------
--------------------------------------------------------------------------------
--------------------------------------------------------------------------------
--------------------------------------------------------------------------------
--------------------------------------------------------------------------------
--------------------------------------------------------------------------------
--------------------------------------------------------------------------------
--------------------------------------------------------------------------------
--------------------------------------------------------------------------------
--------------------------------------------------------------------------------
--------------------------------------------------------------------------------
--------------------------------------------------------------------------------
--------------------------------------------------------------------------------
--------------------------------------------------------------------------------
--------------------------------------------------------------------------------
--------------------------------------------------------------------------------
--------------------------------------------------------------------------------
---
>IIIB-VH12cons_Revexon1
--------------------------------------------------------------------------------
--------------------------------------------------------------------------------
--------------------------------------------------------------------------------
--------------------------------------------------------------------------------
--------------------------------------------------------------------------------
--------------------------------------------------------------------------------
--------------------------------------------------------------------------------
--------------------------------------------------------------------------------
--------------------------------------------------------------------------------
--------------------------------------------------------------------------------
--------------------------------------------------------------------------------
--------------------------------------------------------------------------------
--------------------------------------------------------------------------------
--------------------------------------------------------------------------------
--------------------------------------------------------------------------------
--------------------------------------------------------------------------------
--------------------------------------------------------------------------------
--------------------------------------------------------------------------------
--------------------------------------------------------------------------------
--------------------------------------------------------------------------------
--------------------------------------------------------------------------------
--------------------------------------------------------------------------------
--------------------------------------------------------------------------------
--------------------------------------------------------------------------------
--------------------------------------------------------------------------------
--------------------------------------------------------------------------------
--------------------------------------------------------------------------------
--------------------------------------------------------------------------------
--------------------------------------------------------------------------------
--------------------------------------------------------------------------------
--------------------------------------------------------------------------------
--------------------------------------------------------------------------------
--------------------------------------------------------------------------------
--------------------------------------------------------------------------------
--------------------------------------------------------------------------------
--------------------------------------------------------------------------------
--------------------------------------------------------------------------------
--------------------------------------------------------------------------------
--------------------------------------------------------------------------------
--------------------------------------------------------------------------------
--------------------------------------------------------------------------------
--------------------------------------------------------------------------------
--------------------------------------------------------------------------------
--------------------------------------------------------------------------------
--------------------------------------------------------------------------------
--------------------------------------------------------------------------------
--------------------------------------------------------------------------------
```


---ATGGCAGGAAGAAGCGGAGACAGCGACGAAG
ACCTCCTCAAGGCAGTCAGACTCATCAAGTTTCTCTATCAAAGCA-----------------------------------
----

>IIIB-VH12cons_vpu

---ATGCAACCTATACAAATAG
CAATAGTAGCATTAGTAGTAGCAATAATAATAGCAATAGTTGTGTGGTCCATAGTAATCATAGAATATAGGAAAATATTA
AGACAAAGAAAAATAGACAGGTTAATTGATAGACTAATAGAAAGAGCAGAAGACAGTGGCAATGAGAGTGAAGGAGAAAT
ATCAGCACTTGTGGAGATGGGGGTGGAGATGGGGCACCATGCTCCTTGGGATGTTGATGATCTGTAG-------------
----


```
>IIIB-VH12cons_env
--------------------------------------------------------------------------------
--------------------------------------------------------------------------------
--------------------------------------------------------------------------------
--------------------------------------------------------------------------------
--------------------------------------------------------------------------------
--------------------------------------------------------------------------------
--------------------------------------------------------------------------------
--------------------------------------------------------------------------------
--------------------------------------------------------------------------------
--------------------------------------------------------------------------------
--------------------------------------------------------------------------------
--------------------------------------------------------------------------------
--------------------------------------------------------------------------------
--------------------------------------------------------------------------------
--------------------------------------------------------------------------------
--------------------------------------------------------------------------------
--------------------------------------------------------------------------------
--------------------------------------------------------------------------------
--------------------------------------------------------------------------------
--------------------------------------------------------------------------------
--------------------------------------------------------------------------------
--------------------------------------------------------------------------------
--------------------------------------------------------------------------------
--------------------------------------------------------------------------------
--------------------------------------------------------------------------------
--------------------------------------------------------------------------------
--------------------------------------------------------------------------------
--------------------------------------------------------------------------------
--------------------------------------------------------------------------------
--------------------------------------------------------------------------------
--------------------------------------------------------------------------------
--------------------------------------------------------------------------------
--------------------------------------------------------------------------------
--------------------------------------------------------------------------------
--------------------------------------------------------------------------------
--------------------------------------------------------------------------------
--------------------------------------------------------------------------------
--------------------------------------------------------------------------------
--------------------------------------------------------------------------------
--------------------------------------------------------------------------------
--------------------------------------------------------------------------------
--------------------------------------------------------------------------------
--------------------------------------------------------------------------------
--------------------------------------------------------------------------------
--------------------------------------------------------------------------------
--------------------------------------------------------------------------------
--------------------------------------------------------------------------------
--------------------------------------------------------------------------------
--------------------------------------------------------------------------------
--------------------------------------------------------------------------------
--------------------------------------------------------------------------------
--------------------------------------------------------------------------------
--------------------------------------------------------------------------------
--------------------------------------------------------------------------------
--------------------------------------------------------------------------------
--------------------------------------------------------------------------------
--------------------------------------------------------------------------------
--------------------------------------------------------------------------------
--------------------------------------------------------------------------------
--------------------------------------------------------------------------------
--------------------------------------------------------------------------------
--------------------------------------------------------------------------------
--------------------------------------------------------------------------------
--------------------------------------------------------------------------------
--------------------------------------------------------------------------------
```


---ATGAGAGTGAAGGAGAAAT
ATCAGCACTTGTGGAGATGGGGGTGGAGATGGGGCACCATGCTCCTTGGGATGTTGATGATCTGTAGTGCTACAGAAAAA
TTGTGGGTCACAGTCTATTATGGGGTACCTGTGTGGAAGGAAGCAACCACCACTCTATTTTGTGCATCAGATGCTAAAGC
ATATGATACAGAGGTACATAATGTTTGGGCCACACATGCCTGTGTACCCACAGACCCCAACCCACAAGAAGTAGTATTGG
TAAATGTGACAGAAAATTTTAACATGTGGAAAAATGACATGGTAGAACAGATGCATGAGGATATAATCAGTTTATGGGAT
CAAAGCCTAAAGCCATGTGTAAAATTAACCCCACTCTGTGTTAGTTTAAAGTGCACTGATTTGAAGAATGATACTAATAC
CAATAGTAGTAGCGGGAGAATGATAATGGAGAAAGGAGAGATAAAAAACTGCTCTTTCAATATCAGCACAAGCATAAGAG
GTAAGGTGCAGAAAGAATATGCATTTTTTTATAAACTTGATATAATACCAATAGATAATGATACTACCAGCTATACGTTG
ACAAGTTGTAACACCTCAGTCATTACACAGGCCTGTCCAAAGGTATCCTTTGAGCCAATTCCCATACATTATTGTGCCCC
GGCTGGTTTTGCGATTCTAAAATGTAATAATAAGACGTTCAATGGAACAGGACCATGTACAAATGTCAGCACAGTACAAT
GTACACATGGAATTAGGCCAGTAGTATCAACTCAACTGCTGTTAAATGGCAGTCTAGCAGAAGAAGAGGTAGTAATTAGA
TCTGTCAATTTCACGGACAATGCTAAAACCATAATAGTACAGCTGAACACATCTGTAGAAATTAATTGTACAAGACCCAA
CAACAATACAAGAAAAAAAATCCGTATCCAGAGGGGACCAGGGAGAGCATTTGTTACAATAGGAAAAATAGGAAATATGA
GACAAGCACATTGTAACATTAGTAGAGCAAAATGGAATGCCACTTTAAAACAGATAGCTAGCAAATTAAGAGAACAATTT
GGAAATAATAAAACAATAATCTTTAAGCAATCCTCAGGAGGGGACCCAGAAATTGTAACGCACAGTTTTAATTGTGGAGG
GGAATTTTTCTACTGTAATTCAACACAACTGTTTAATAGTACTTGGTTTAATAGTACTTGGAGTACTGAAGGGTCAAATA
ACACTGAAGGAAGTGACACAATCACACTCCCATGCAGAATAAAACAATTTATAAACATGTGGCAGGAAGTAGGAAAAGCA
ATGTATGCCCCTCCCATCAGCGGACAAATTAGATGTTCATCAAATATTACAGGGCTGCTATTAACAAGAGATGGTGGTAA
TAACAACAATGGGTCCGAGATCTTCAGACCTGGAGGAGGAGATATGAGGGACAATTGGAGAAGTGAATTATATAAATATA
AAGTAGTAAAAATTGAACCATTAGGAGTAGCACCCACCAAGGCAAAGAGAAGAGTGGTGCAGAGAGAAAAAAGAGCAGTG
GGAATAGGAGCTTTGTTCCTTGGGTTCTTGGGAGCAGCAGGAAGCACTATGGGCGCAGCGTCAATGACGCTGACGGTACA
GGCCAGACAATTATTGTCTGGTATAGTGCAGCAGCAGAACAATTTGCTGAGGGCTATTGAGGCGCAACAGCATCTGTTGC
AACTCACAGTCTGGGGCATCAAGCAGCTCCAGGCAAGAATCCTGGCTGTGGAAAGATACCTAAAGGATCAACAGCTCCTG
GGGATTTGGGGTTGCTCTGGAAAACTCATTTGCACCACTGCTGTGCCTTGGAATGCTAGTTGGAGTAATAAATCTCTGGA
ACAGATTTGGAATCACACGACCTGGATGGAGTGGGACAGAGAAATTAACAATTACACAAGCTTAATACACTCCTTAATTG
AAGAATCGCAAAACCAGCAAGAAAAGAATGAACAAGAATTATTGGAATTAGATAAATGGGCAAGTTTGTGGAATTGGTTT
AACATAACAAATTGGCTGTGGTATATAAAATTATTCATAATGATAGTAGGAGGCTTGGTAGGTTTAAGAATAGTTTTTGC
TGTACTTTCTGTAGTGAATAGAGTTAGGCAGGGATATTCACCATTATCGTTTCAGACCCACCTCCCAATCCCGAGGGGAC
CCGACAGGCCCGAAGGAATAGAAGAAGAAGGTGGAGAGAGAGACAGAGACAGATCCATTCGATTAGTGAACGGATCCTTA
GCACTTATCTGGGACGATCTGCGGAGCCTGTGCCTCTTCAGCTACCACCGCTTGAGAGACTTACTCTTGATTGTAACGAG
GATTGTGGAACTTCTGGGACGCAGGGGGTGGGAAGCCCTCAAATATTGGTGGAATCTCCTACAATATTGGAGTCAGGAGC
TAAAGAATAGTGCTGTTAGCTTGCTCAATGCCACAGCTATAGCAGTAGCTGAGGGGACAGATAGGGTTATAGAAGTAGTA
CAAGAAGCTTATAGAGCTATTCGCCACATACCTAGAAGAATAAGACAGGGCTTGGAAAGGATTTGCTATAA--------
----

>IIIB-VH12cons_TatExon2

---ACCCACCTCCCAATCCCGAGGGGAC
CCGACAGGCCCGAAGGAATAGAAGAAGAAGGTGGAGAGAGAGACAGAGACAGATCCATTCGATTAG--------------
----

>IIIB-VH12cons_RevExon2

---ACCCACCTCCCAATCCCGAGGGGAC
CCGACAGGCCCGAAGGAATAGAAGAAGAAGGTGGAGAGAGAGACAGAGACAGATCCATTCGATTAGTGAACGGATCCTTA
GCACTTATCTGGGACGATCTGCGGAGCCTGTGCCTCTTCAGCTACCACCGCTTGAGAGACTTACTCTTGATTGTAACGAG
GATTGTGGAACTTCTGGGACGCAGGGGGTGGGAAGCCCTCAAATATTGGTGGAATCTCCTACAATATTGGAGTCAGGAGC
TAAAGAATAG--

>IIIB-VH12cons_nef

--TGGGTG
GCAAGTGGTCAAAAAGTAGTGTGGTTGGATGGCCTGCTGTAAGGGAAAGAATGAGACGAGCTGAGCCAGCAGCAGATGGG
GTGGGAGCAGCATCTCGAGACCTAGAAAAACATGGAGCAATCACAAGTAGCAACACAGCAGCTAACAATGCTGCTTGTGC
CTGGCTAGAAGCACAAGAGGAGGAGAAGGTGGGTTTTCCAGTCACACCTCAGGTACCTTTAAGACCAATGACTTACAAGG
CAGCTGTAGATCTTAGCCACTTTTTAAAAGAAAAGGGGGGACTGGAAGGGCTAATTCACTCCCAACGAAGACAAGATATC
CTTGATCTGTGGATCTACCACACACAAGGCTACTTCCCTGATTGGCAGAACTACACACCAGGACCAGGGATCAGATATCC
ACTGACCTTTGGATGGCGCTACAAGCTAGTACCAGTTGAGCCAGAGAAGTTAGAAGAAGCCAACAAAGGAGAGAACACCA
GCTTGTTACACCCTGTGAGCCTGCATGGAATGGATGACCCGGAGAGAGAAGTGTTAGAGTGGAGGTTTGACAGCCGCCTA
GCATTTCATCACGTGGCCCGAGAGCTGCATCCGGAGTACTTCAAGAACTGCTGA--------------------------
----

