# Supplementary material for: APOBEC3G-Induced Hypermutation of Human Immunodeficiency Virus Type-1 Is Typically a Discrete “All or Nothing” Phenomenon
Source: PLoS Genet. 2012 Mar 22;8(3):e1002550. doi: 10.1371/journal.pgen.1002550 (PMC3310730; doi:10.1371/journal.pgen.1002550)
Supplement: Figure S2 — HIVIIIB sequence alignments from the in vitro titration experiment: 1) envLTR and 2) almost full-length provirus. (PDF) [file pgen.1002550.s002.pdf]

Supplementary Figure 2.

1.Sequence alignments from the in vitro titration experiment: envLTR [3GtitnALL_EnvLTR_seqs.fasta]

```
>consensus
--------------------------------------------------------------------------------
--------------------------------------------------------------------------------
--------------------------------------------------------------------------------
--------------------------------------------------------------------------------
--------------------------------------------------------------------------------
--------------------------------------------------------------------------------
--------------------------------------------------------------------------------
--------------------------------------------------------------------------------
--------------------------------------------------------------------------------
--------------------------------------------------------------------------------
--------------------------------------------------------------------------------
--------------------------------------------------------------------------------
--------------------------------------------------------------------------------
--------------------------------------------------------------------------------
--------------------------------------------------------------------------------
--------------------------------------------------------------------------------
--------------------------------------------------------------------------------
--------------------------------------------------------------------------------
--------------------------------------------------------------------------------
--------------------------------------------------------------------------------
--------------------------------------------------------------------------------
--------------------------------------------------------------------------------
--------------------------------------------------------------------------------
--------------------------------------------------------------------------------
--------------------------------------------------------------------------------
--------------------------------------------------------------------------------
--------------------------------------------------------------------------------
--------------------------------------------------------------------------------
--------------------------------------------------------------------------------
--------------------------------------------------------------------------------
--------------------------------------------------------------------------------
--------------------------------------------------------------------------------
--------------------------------------------------------------------------------
--------------------------------------------------------------------------------
--------------------------------------------------------------------------------
--------------------------------------------------------------------------------
--------------------------------------------------------------------------------
--------------------------------------------------------------------------------
--------------------------------------------------------------------------------
--------------------------------------------------------------------------------
--------------------------------------------------------------------------------
--------------------------------------------------------------------------------
--------------------------------------------------------------------------------
--------------------------------------------------------------------------------
--------------------------------------------------------------------------------
--------------------------------------------------------------------------------
--------------------------------------------------------------------------------
--------------------------------------------------------------------------------
--------------------------------------------------------------------------------
--------------------------------------------------------------------------------
--------------------------------------------------------------------------------
--------------------------------------------------------------------------------
--------------------------------------------------------------------------------
--------------------------------------------------------------------------------
--------------------------------------------------------------------------------
--------------------------------------------------------------------------------
--------------------------------------------------------------------------------
--------------------------------------------------------------------------------
--------------------------------------------------------------------------------
--------------------------------------------------------------------------------
--------------------------------------------------------------------------------
--------------------------------------------------------------------------------
--------------------------------------------------------------------------------
--------------------------------------------------------------------------------
--------------------------------------------------------------------------------
--------------------------------------------------------------------------------
--------------------------------------------------------------------------------
--------------------------------------------------------------------------------
--------------------------------------------------------------------------------
--------------------------------------------------------------------------------
--------------------------------------------------------------------------------
--------------------------------------------------------------------------------
--------------------------------------------------------------------------------
--------------------------------------------------------------------------------
--------------------------------------------------------------------------------
--------------------------------------------------------------------------------
--------------------------------------------------------------------------------
--------------------------------------------------------------------------------
--------------------------------------------------------------------------------
--------------------------------------------------------------------------------
--------------------------------------------------------------------------------
--------------------------------------------------------------------------------
--------------------------------------------------------------------------------
--------------------------------------------------------------------------------
--------------------------------------------------------------------------------
--------------------------------------------------------------------------------
--------------------------------------------------------------------------------
--------------------------------------------------------------------------------
```

```
--------------------------------------------------------------------------------
--------------------------------------------------------------------------------
--------------------------------------------------------------------------------
--------------------------------------------------------------------------------
--------------------------------------------------------------------------------
------------------------------------------AAAGCAATGTATGCCCCTCCCATCAGCGGACAAATTAG
ATGTTCATCAAATATTACAGGGCTGCTATTAACAAGAGATGGTGGTAATAACAACAATGGGTCCGAGATCTTCAGACCTG
GAGGAGGAGATATGAGGGACAATTGGAGAAGTGAATTATATAAATATAAAGTAGTAAAAATTGAACCATTAGGAGTAGCA
CCCACCAAGGCAAAGAGAAGAGTGGTGCAGAGAGAAAAAAGAGCAGTGGGAATAGGAGCTTTGTTCCTTGGGTTCTTGGG
AGCAGCAGGAAGCACTATGGGCGCAGCGTCAATGACGCTGACGGTACAGGCCAGACAATTATTGTCTGGTATAGTGCAGC
AGCAGAACAATTTGCTGAGGGCTATTGAGGCGCAACAGCATCTGTTGCAACTCACAGTCTGGGGCATCAAGCAGCTCCAG
GCAAGAATCCTGGCTGTGGAAAGATACCTAAAGGATCAACAGCTCCTGGGGATTTGGGGTTGCTCTGGAAAACTCATTTG
CACCACTGCTGTGCCTTGGAATGCTAGTTGGAGTAATAAATCTCTGGAACAGATTTGGAATCACACGACCTGGATGGAGT
GGGACAGAGAAATTAACAATTACACAAGCTTAATACACTCCTTAATTGAAGAATCGCAAAACCAGCAAGAAAAGAATGAA
CAAGAATTATTGGAATTAGATAAATGGGCAAGTTTGTGGAATTGGTTTAACATAACAAATTGGCTGTGGTATATAAAATT
ATTCATAATGATAGTAGGAGGCTTGGTAGGTTTAAGAATAGTTTTTGCTGTACTTTCTGTAGTGAATAGAGTTAGGCAGG
GATATTCACCATTATCGTTTCAGACCCACCTCCCAATCCCGAGGGGACCCGACAGGCCCGAAGGAATAGAAGAAGAAGGT
GGAGAGAGAGACAGAGACAGATCCATTCGATTAGTGAACGGATCCTTAGCACTTATCTGGGACGATCTGCGGAGCCTGTG
CCTCTTCAGCTACCACCGCTTGAGAGACTTACTCTTGATTGTAACGAGGATTGTGGAACTTCTGGGACGCAGGGGGTGGG
AAGCCCTCAAATATTGGTGGAATCTCCTACAATATTGGAGTCAGGAGCTAAAGAATAGTGCTGTTAGCTTGCTCAATGCC
ACAGCTATAGCAGTAGCTGAGGGGACAGATAGGGTTATAGAAGTAGTACAAGAAGCTTATAGAGCTATTCGCCACATACC
TAGAAGAATAAGACAGGGCTTGGAAAGGATTTTGCTATAAGATGGGTGGCAAGTGGTCAAAAAGTAGTGTGGTTGGATGG
CCTGCTGTAAGGGAAAGAATGAGACGAGCTGAGCCAGCAGCAGATGGGGTGGGAGCAGCATCTCGAGACCTAGAAAAACA
TGGAGCAATCACAAGTAGCAACACAGCAGCTAACAATGCTGCTTGTGCCTGGCTAGAAGCACAAGAGGAGGAGAAGGTGG
GTTTTCCAGTCACACCTCAGGTACCTTTAAGACCAATGACTTACAAGGCAGCTGTAGATCTTAGCCACTTTTTAAAAGAA
AAGGGGGGACTGGAAGGGCTAATTCACTCCCAACGAAGACAAGATATCCTTGATCTGTGGATCTACCACACACAAGGCTA
CTTCCCTGATTGGCAGAACTACACACCAGGACCAGGGATCAGATATCCACTGACCTTTGGATGGCGCTACAAGCTAGTAC
CAGTTGAGCCAGAGAAGTTAGAAGAAGCCAACAAAGGAGAGAACACCAGCTTGTTACACCCTGTGAGCCTGCATGGAATG
GATGACCCGGAGAGAGAAGTGTTAGAGTGGAGGTTTGACAGCCGCCTAGCATTTCATCACGTGGCCCGAGAGCTGCATCC
GGAGTACTTCAAGAACTGCTGATATCGAGCTTGCTACAAGGGACTTTCCGCTGGGGACTTTCCAGGGAGGCGTGGCCTGG
GCGGGACTGGGGAGTGGCGAGCCCTCAGATCCTGCATATAAGCAGCTGCTTTTTGCCTGTACTGGGTCTCTCTGGTTAGA
CCAGATCTGAGCCTGGGAGCTCTCTGGCTAACTAGGGAACCCACTGCTTAAGCCTCAATAAAGCTTGCCTTGAGTGCTTC
AAGTAGTGTGTGCCCATCTGTTGTGTGACTCTGGTAACTAGAGATCCCTCA
>1-10envLTR
--------------------------------------------------------------------------------
--------------------------------------------------------------------------------
--------------------------------------------------------------------------------
--------------------------------------------------------------------------------
--------------------------------------------------------------------------------
--------------------------------------------------------------------------------
--------------------------------------------------------------------------------
--------------------------------------------------------------------------------
--------------------------------------------------------------------------------
--------------------------------------------------------------------------------
--------------------------------------------------------------------------------
--------------------------------------------------------------------------------
--------------------------------------------------------------------------------
--------------------------------------------------------------------------------
--------------------------------------------------------------------------------
--------------------------------------------------------------------------------
--------------------------------------------------------------------------------
--------------------------------------------------------------------------------
--------------------------------------------------------------------------------
--------------------------------------------------------------------------------
--------------------------------------------------------------------------------
--------------------------------------------------------------------------------
--------------------------------------------------------------------------------
--------------------------------------------------------------------------------
--------------------------------------------------------------------------------
--------------------------------------------------------------------------------
--------------------------------------------------------------------------------
--------------------------------------------------------------------------------
--------------------------------------------------------------------------------
--------------------------------------------------------------------------------
--------------------------------------------------------------------------------
--------------------------------------------------------------------------------
--------------------------------------------------------------------------------
--------------------------------------------------------------------------------
--------------------------------------------------------------------------------
--------------------------------------------------------------------------------
--------------------------------------------------------------------------------
--------------------------------------------------------------------------------
--------------------------------------------------------------------------------
--------------------------------------------------------------------------------
--------------------------------------------------------------------------------
--------------------------------------------------------------------------------
--------------------------------------------------------------------------------
--------------------------------------------------------------------------------
--------------------------------------------------------------------------------
--------------------------------------------------------------------------------
--------------------------------------------------------------------------------
--------------------------------------------------------------------------------
--------------------------------------------------------------------------------
--------------------------------------------------------------------------------
--------------------------------------------------------------------------------
--------------------------------------------------------------------------------
--------------------------------------------------------------------------------
--------------------------------------------------------------------------------
--------------------------------------------------------------------------------
--------------------------------------------------------------------------------
--------------------------------------------------------------------------------
--------------------------------------------------------------------------------
--------------------------------------------------------------------------------
--------------------------------------------------------------------------------
```


-AGGAGGAGATATGAGGGACAATTGGAGAAGTGAATTATATAAATATAAAGTAGTAAAAATTGAACCATTAGGAGTAGCA
CCCACCAAGGCAAAGAGAAGAGTGGTGCANAGAGAAAAAAGAGCAGTGGGAATAGGAGCTTTGTTCCTTGGGTTCTTGGG
AGCAGCAGGAAGCACTATGGGCGCAGCGTCAATGACGTTGACGGTACAGGCCAGACAATTATTGTCTGGTATAGTGCAGC
AGCAGAACAATTTGCTGAGGGCTATTGAGGCGCAACAGCATCTGTTGCAACTCACAGTCTGGGGCATCAAGCAGCTCCAG
GCAAGAATCCTGGCTGTGGAAAGATACCTAAAGGATCAACAGCTCCTGGGGATTTGGGGTTGCTCTGGAAAACTCATTTG
CACCACTGCTGTGCCTTGGAATGCTAGTTGGAGTAATAAATCTCTGGAACAGATTTGGAATCACACGACCTGGATGGAGT
GGGACAGAGAAATTAACAATTACACAAGCTTAATACACTCCTTAATTGAAGAATCGCAAAACCAGCAAGAAAAGAATGAA
CAAGAATTATTGGAATTAGATAAATGGGCAAGTTTGTGGAATTGGTTTAACATAACAAATTGGCTGTGGTATATAAAATT
ATTCATAATGATAGTAGGAGGCTTGGTAGGTTTAAGAATAGTTTTTGCTGTACTTTCTGTAGTGAATAGAGTTAGGCAGG
GATATTCACCATTATCGTTTCAGACCCACCTCCCAATCCCGAGGGGACCCGACAGGCCCGAAGGAATAGAAGAAGAAGGT
GGAGAGAGAGACAGAGACAGATCCATTCGATTAGTGAACGGATCCTTAGCACTTATCTGGGACGATCTGCGGAGCCTGTG
CCTCTTCAGCTACCACCGCTTGAGAGACTTACTCTTGATTGTAACGAGGATTGTGGAACTTCTGGGACGCAGGGGGTGGG
AAGCCCTCAAATATTGGTGGAATCTCCTACAATATTGGAGTCAGGAGCTAAAGAATAGTGCTGTTAGCTTGCTCAATGCC
ACAGCTATAGCAGTAGCTGAGGGGACAGATAGGGTTATAGAAGTAGTACAAGAAGCTTATAGAGCTATTCGCCACATACC
TAGAAGAATAAGACAGGGCTTGGAAAGGATTTTGCTATAAGATGGGTGGCAAGTGGTCAAAAAGTAGTGTGGTTGGATGG
CCTGCTGTAAGGGAAAGAATGAGACGAGCTGAGCCAGCAGCAGATGGGGTGGGAGCAGCATCTCGAGACCTAGAAAAACA
TGGAGCAATCACAAGTAGCAACACAGCAGCTAACAATGCTGCTTGTGCCTGGCTAGAAGCACAAGAGGAGGAGAAGGTGG
GTTTTCCAGTCACACCTCAGGTACCTTTAAGACCAATGACTTACAAGGCAGCTGTAGATCTTAGCCACTTTTTAAAAGAA
AAGGGGGGACTGGAAGGGCTAATTCACTCCCAACGAAGACAAGATATCCTTGATCTGTGGATCTACCACACACAAGGCTA
CTTCCCTGATTGGCAGAACTACACACCAGGACCAGGGATCAGATATCCACTGACCTTTGGATGGCGCTACAAGCTAGTAC
CAGTTGAGCCAGAGAAGTTAGAAGAAGCCAACAAAGGAGAGAACACCAGCTTGTTACACCCTGTGAGCCTGCATGGAATG
GATGACCCGGAGAGAGAAGTGTTAGAGTGGAGGTTTGACAGCCGCCTAGCATTTCATCACGTGGCCCGAGAGCTGCATCC
GGAGTACTTCAAGAACTGCTGATATCGAGCTTGCTACAAGGGACTTTCCGCTGGGGACTTTCCAGGGAGGCGTGGCCTGG
GCGGGACTGGGGAGTGGCGAGCCCTCAGATCCTGCATATAAGCAGCTGCTTTTTGCCTGTACTGGGTCTCTCTGGTTAGA
CCAGATCTGAGCCTGGGAGCTCTCTGGCTAACTAGGGAACCCACTGCTTAAGCCTCAATAAAGCTTGCCTTGAGTGCTTC
AAGTAGTGTGTGCCCATCTGTTGTGTGACTCTGGTAACTAGAGATCCCTCA
>1-11envLTR

-------------------GGGCTGCTATTAACAAGAGATGGTGGTAATAACAACAATGGGTCCGAGATCTTCAGACCTG
GAGGAGGAGATATGAGGGACAATTGGAGAAGTGAATTATATAAATATAAAGTAGTAAAAATTGAACCATTAGGAGTAGCA
CCCACCAAGGCAAAGAGAAGAGTGGTGCAGAGAGAAAAAAGAGCAGTGGGAATAGGAGCTTTGTTCCTTGGGTTCTTGGG
AGCAGCAGGAAGCACTATGGGCGCAGCGTCAATGACGCTGACGGTACAGGCCAGACAATTATTGTCTGGTATAGTGCAGC
AGCAGAACAATTTGCTGAGGGCTATTGAGGCGCAACAGCATCTGTTGCAACTCACAGTCTGGGGCATCAAGCAGCTCCAG
GCAAGAATCCTGGCTGTGGAAAGATACCTAAAGGATCAACAGCTCCTGGGGATTTGGGGTTGCTCTGGAAAACTCATTTG
CACCACTGCTGTGCCTTGGAATGCTAGTTGGAGTAATAAATCTCTGGAACAGATTTGGAATCACACGACCTGGATGGAGT
GGGACAGAGAAATTAACAATTACACAAGCTTAATACACTCCTTAATTGAAGAATCGCAAAACCAGCAAGAAAAGAATGAA
CAAGAATTATTGGAATTAGATAAATGGGCAAGTTTGTGGAATTGGTTTAACATAACAAATTGGCTGTGGTATATAAAATT
ATTCATAATGATAGTAGGAGGCTTGGTAGGTTTAAGAATAGTTTTTGCTGTACTTTCTGTAGTGAATAGAGTTAGGCAGG
GATATTCACCATTATCGTTTCAGACCCACCTCCCAATCCCGAGGGGACCCGACAGGCCCGAAGGAATAGAAGAAGAAGGT
GGAGAGAGAGACAGAGACAGATCCATTCGATTAGTGAACGGATCCTTAGCACTTATCTGGGACGATCTGCGGAGCCTGTG
CCTCTTCAGCTACCACCGCTTGAGAGACTTACTCTTGATTGTAACGAGGATTGTGGAACTTCTGGGACGCAGGGGGTGGG
AAGCCCTCAAATATTGGTGGAATCTCCTACAATATTGGAGTCAGGAGCTAAAGAATAGTGCTGTTAGCTTGCTCAATGCC
ACAGCTATAGCAGTAGCTGAGGGGACAGATAGGGTTATAGAAGTAGTACAAGAAGCTTATAGAGCTATTCGCCACATACC
TAGAAGAATAAGACAGGGCTTGGAAAGGATTTTGCTATAAGATGGGTGGCAAGTGGTCAAAAAGTAGTGTGGTTGGATGG
CCTGCTGTAAGGGAAAGAATGAGACGAGCTGAGCCAGCAGCAGATGGGGTGGGAGCAGCATCTCGAGACCTAGAAAAACA
TGGAGCAATCACAAGTAGCAACACAGCAGCTAACAATGCTGCTTGTGCCTGGCTAGAAGCACAAGAGGAGGAGAAGGTGG
GTTTTCCAGTCACACCTCAGGTACCTTTAAGACCAATGACTTACAAGGCAGCTGTAGATCTTAGCCACTTTTTAAAAGAA
AAGGGGGGACTGGAAGGGCTAATTCACTCCCAACGAAGACAAGATATCCTTGATCTGTGGATCTACCACACACAAGGCTA
CTTCCCTGATTGGCAGAACTACACACCAGGACCAGGGATCAGATATCCACTGACCTTTGGATGGCGCTACAAGCTAGTAC
CAGTTGAGCCAGAGAAGTTAGAAGAAGCCAACAAAGGAGAGAACACCAGCTTGTTACACCCTGTGAGCCTGCATGGAATG
GATGACCCGGAGAGAGAAGTGTTAGAGTGGAGGTTTGACAGCCGCCTAGCATTTCATCACGTGGCCCGAGAGCTGCATCC
GGAGTACTTCAAGAACTGCTGATATCGAGCTTGCTACAAGGGACTTTCCGCTGGGGACTTTCCAGGGAGGCGTGGCCTGG
GCGGGACTGGGGAGTGGCGAGCCCTCAGATCCTGCATATAAGCAGCTGCTTTTTGCCTGTACTGGGTCTCTCTGGTTAGA
CCAGATCTGAGCCTGGGAGCTCTCTGGCTAACTAGGGAACCCACTGCTTAAGCCTCAATAAAGCTTGCCTTGAGTGCTTC
AAGTAGTGTGTGCCCATCTGTTGTGTGACTCTGGTAACTAGAGATCCCTCA
>1-13envLTR


```
--------------------------------------------------------------------------------
--------------------------------------------------------------------------------
--------------------------------------------------------------------------------
--------------------------------------------------------------------------------
--------------------------------------------------------------------------------
--------------------------------------------------------------------------------
--------------------------------------------------------------------------------
--------------------------------------------------------------------------------
--------------------------------------------------------------------------------
--------------------------------------------------------------------------------
--------------------------------------------------------------------------------
--------------------------------------------------------------------------------
--------------------------------------------------------------------------------
--------------------------------------------------------------------------------
--------------------------------------------------------------------------------
--------------------------------------------------------------------------------
--------------------------------------------------------------------------------
--------------------------------------------------------------------------------
--------------------------------------------------------------------------------
--------------------------------------------------------------------------------
--------------------------------------------------------------------------------
--------------------------------------------------------------------------------
--------------------------------------------------------------------------------
--------------------------------------------------------------------------------
--------------------------------------------------------------------------------
--------------------------------------------------------------------------------
--------------------------------------------------------------------------------
--------------------------------------------------------------------------------
--------------------------------------------------------------------------------
--------------------------------------------------------------------------------
--------------------------------------------------------------------------------
--------------------------------------------------------------------------------
--------------------------------------------------------------------------------
--------------------------------------------------------------------------------
--------------------------------------------------------------------------------
--------------------------------------------------------------------------------
--------------------------------------------------------------------------------
--------------------------------------------------------------------------------
--------------------------------------------------------------------------------
--------------------------------------------------------------------------------
--------------------------------------------------------------------------------
--------------------------------------------------------------------------------
--------------------------------------------------------------------------------
--------------------------------------------------------------------------------
--------------------------------------------------------------------------------
--------------------------------------------------------------------------------
--------------------------------------------------------------------------------
--------------------------------------------------------------------------------
--------------------------------------------------------------------------------
--------------------------------------------------------------------------------
--------------------------------------------------------------------------------
--------------------------------------------------------------------------------
--------------------------------------------------------------------------------
--------------------------------------------------------------------------------
--------------------------------------------------------------------------------
--------------------------------------------------------------------------------
--------------------------------------------------------------------------------
--------------------------------------------------------------------------------
--------------------------------------------------------------------------------
--------------------------------------------------------------------------------
--------------------------------------------------------------------------------
--------------------------------------------------------------------------------
--------------------------------------------------------------------------------
--------------------------------------------------------------------------------
--------------------------------------------------------------------------------
--------------------------------------------------------------------------------
--------------------------------------------------------------------------------
--------------------------------------------------------------------------------
--------------------------------------------------------------------------------
--------------------------------------------------------------------------------
--------------------------------------------------------------------------------
--------------------------------------------------------------------------------
--------------------------------------------------------------------------------
--------------------------------------------------------------------------------
--------------------------------------------------------------------------------
--------------------------------------------------------------------------------
--------------------------------------------------------------------------------
--------------------------------------------------------------------------------
--------------------------------------------------------------------------------
--------------------------------------------------------------------------------
--------------------------------------------------------------------------------
--------------------------------------------------------------------------------
--------------------------------------------------------------------------------
--------------------------------------------------------------------------------
--------------------------------------------------------------------------------
--------------------------------------------------------------------------------
--------------------------------------------------------------------------------
--------------------------------------------------------------------------------
------------------------------------------AAAGCAATGTATGCCCCTCCCATCAGCGGACAAATTAG
ATGTTCATCAAATATTACAGGGCTGCTATTAACAAGAGATGGTGGTAATAACAACAATGGGTCCGAGATCTTCAGACCTG
GAGGAGGAGATATGAGGGACAATTG-------------------------------------------------------
----CCAAGGCAAAGAGAAGAGTGGTGCAGAGAGAAAAAAGAGCAGTGGGAATAGGAGCTTTGTTCCTTGGGTTCTTGGG
AGCAGCAGGAAGCACTATGGGCGCAGCGTCAATGACGCTGACGGTACAGGCCAGACAATTATTGTCTGGTATAGTGCAGC
```

AGCAGAACAATTTGCTGAGGGCTATTGAGGCGCAACAGCATCTGTTGCAACTCACAGTCTGGGGCATCAAGCAGCTCCAG
GCAAGAATCCTGGCTGTGGAAAGATACCTAAAGGATCAACAGCTCCTGGGGATTTGGGGTTGCTCTGGAAAACTCATTTG
CACCACTGCTGTGCCTTGGAATGCTAGTTGGAGTAATAAATCTCTGGAACAGATTTGGAATCACACGACCTGGATGGAGT
GGGACAGAGAAATTAACAATTACACAAGCTTAATACACTCCTTAATTGAAGAATCGCAAAACCAGCAAGAAAAGAATGAA
CAAGAATTATTGGAATTAGATAAATGGGCAAGTTTGTGGAATTGGTTTAACATAACAAATTGGCTGTGGTATATAAAATT
ATTCATAATGATAGTAGGAGGCTTGGTAGGTTTAAGAATAGTTTTTGCTGTACTTTCTGTAGTGAATAGAGTTAGGCAGG
GATATTCACCATTATCGTTTCAGACCCACCTCCCAATCCCGAGGGGACCCGACAGGCCCGAAGGAATAGAAGAAGAAGGT
GGAGAGAGAGACAGAGACAGATCCATTCGATTAGTGAACGGATCCTTAGCACTTATCTGGGACGATCTGCGGAGCCTGTG
CCTCTTCAGCTACCACCGCTTGAGAGACTTACTCTTGATTGTAACGAGGATTGTGGAACTTCTGGGACGCAGGGGGTGGG
AAGCCCTCAAATATTGGTGGAATCTCCTACAATATTGGAGTCAGGAGCTAAAGAATAGTGCTGTTAGCTTGCTCAATGCC
ACAGCTATAGCAGTAGCTGAGGGGACAGATAGGGTTATAGAAGTAGTACAAGAAGCTTATAGAGCTATTCGCCACATACC
TAGAAGAATAAGACAGGGCTTGGAAAGGATTTTGCTATAAGATGGGTGGCAAGTGGTCAAAAAGTAGTGTGGTTGGATGG
CCTGCTGTAAGGGAAAGAATGAGACGAGCTGAGCCAGCAGCAGATGGGGTGGGAGCAGCATCTCGAGACCTAGAAAAACA
TGGAGCAATCACAAGTAGCAACACAGCAGCTAACAATGCTGCTTGTGCCTGGCTAGAAGCACAAGAGGAGGAGAAGGTGG
GTTTTCCAGTCACACCTCAGGTACCTTTAAGACCAATGACTTACAAGGCAGCTGTAGATCTTAGCCACTTTTTAAAAGAA
AAGGGGGGACTGGAAGGGCTAATTCACTCCCAACGAAGACAAGATATCCTTGATCTGTGGATCTACCACACACAAGGCTA
CTTCCCTGATTGGCAGAACTACACACCAGGACCAGGGATCAGATATCCACTGACCTTTGGATGGCGCTACAAGCTAGTAC
CAGTTGAGCCAGAGAAGTTAGAAGAAGCCAACAAAGGAGAGAACACCAGCTTGTTACACCCTGTGAGCCTGCATGGAATG
GATGACCCGGAGAGAGAAGTGTTAGAGTGGAGGTTTGACAGCCGCCTAGCATTTCATCACGTGGCCCGAGAGCTGCATCC
GGAGTACTTCAAGAACTGCTGATATCGAGCTTGCTACAAGGGACTTTCCGCTGGGGACTTTCCAGGGAGGCGTGGCCTGG
GCGGGACTGGGGAGTGGCGAGCCCTCAGATCCTGCATATAAGCAGCTGCTTTTTGCCTGTACTGGGTCTCTCTGGTTAGA
CCAGATCTGAGCCTGGGAGCTCTCTGGCTAACTAGGGAACCCACTGCTTAAGCCTCAATAAAGCTTGCCTTGAGTGCTTC
AAGTAGTGTGTGCCCATCTGTTGTGTGACTCTGGTAACTAGAGATCCCTCA

>1-49envLTR


```
--------------------------------------------------------------------------------
--------------------------------------------------------------------------------
--------------------------------------------------------------------------------
--------------------------------------------------------------------------------
--------------------------------------------------------------------------------
--------------------------------------------------------------------------------
--------------------------------------------------------------------------------
--------------------------------------------------------------------------------
--------------------------------------------------------------------------------
--------------------------------------------------------------------------------
--------------------------------------------------------------------------------
--------------------------------------------------------------------------------
--------------------------------------------------------------------------------
--------------------------------------------------------------------------------
--------------------------------------------------------------------------------
--------------------------------------------------------------------------------
--------------------------------------------------------------------------------
--------------------------------------------------------------------------------
--------------------------------------------------------------------------------
--------------------------------------------------------------------------------
--------------------------------------------------------------------------------
--------------------------------------------------------------------------------
--------------------------------------------------------------------------------
--------------------------------------------------------------------------------
----------------------CTGCTATTAACAAGAGATGGTGGTAATAACAACAATGGGTCCGAGATCTTCAGACCTG
GAGGAGGAGATATGAGGGACAATTGGAGAAGTGAATTATATAAATATAAAGTAGTAAAAATTGAACCATTAGGAGTAGCA
CCCACCAAGGCAAAGAGAAGAGTGGTGCAGAGAGAAAAAAGAGCAGTGGGAATAGGAGCTTTGTTCCTTGGGTTCTTGGG
AGCAGCAGGAAGCACTATGGGCGCAGCGTCAATGACGCTGACGGTACAGGCCAGACAATTATTGTCTGGTATAGTGCAGC
AGCAGAACAATTTGCTGAGGGCTATTGAGGCGCAACAGCATCTGTTGCAACTCACAGTCTGGGGCATCAAGCAGCTCCAG
GCAAGAATCCTGGCTGTGGAAAGATACCTAAAGGATCAACAGCTCCTGGGGATTTGGGGTTGCTCTGGAAAACTCATTTG
CACCACTGCTGTGCCTTGGAATGCTAGTTGGAGTAATAAATCTCTGGAACAGATTTGGAATCACACGACCTGGATGGAGT
GGGACAGAGAAATTAACAATTACACAAGCTTAATACACTCCTTAATTGAAGAATCGCAAAACCAGCAAGAAAAGAATGAA
CAAGAATTATTGGAATTAGATAAATGGGCAAGTTTGTGGAATTGGTTTAACATAACAAATTGGCTGTGGTATATAAAATT
ATTCATAATGATAGTAGGAGGCTTGGTAGGTTTAAGAATAGTTTTTGCTGTACTTTCTGTAGTGAATAGAGTTAGGCAGG
GATATTCACCATTATCGTTTCAGACCCACCTCCCAATCCCGAGGGGACCCGACAGGCCCGAAGGAATAGAAGAAGAAGGT
GGAGAGAGAGACAGAGACAGATCCATTCGATTAGTGAACGGATCCTTAGCACTTATCTGGGACGATCTGCGGAGCCTGTG
CCTCTTCAGCTACCACCGCTTGAGAGACTTACTCTTGATTGTAACGAGGATTGTGGAACTTCTGGGACGCAGGGGGTGGG
AAGCCCTCAAATATTGGTGGAATCTCCTACAATATTGGAGTCAGGAGCTAAAGAATAGTGCTGTTAGCTTGCTCAATGCC
ACAGCTATAGCAGTAGCTGAGGGGACAGATAGGGTTATAGAAGTAGTACAAGAAGCTTATAGAGCTATTCGCCACATACC
TAGAAGAATAAGACAGGGCTTGGAAAGGATTTTGCTATAAGATGGGTGGCAAGTGGTCAAAAAGTAGTGTGGTTGGATGG
CCTGCTGTAAGGGAAAGAATGAGACGAGCTGAGCCAGCAGCAGATGGGGTGGGAGCAGCATCTCGAGACCTAGAAAAACA
TGGAGCAATCACAAGTAGCAACACAGCAGCTAACAATGCTGCTTGTGCCTGGCTAGAAGCACAAGAGGAGGAGAAGGTGG
GTTTTCCAGTCACACCTCAGGTACCTTTAAGACCAATGACTTACAAGGCAGCTGTAGATCTTAGCCACTTTTTAAAAGAA
AAGGGGGGACTGGAAGGGCTAATTCACTCCCAACGAAGACAAGATATCCTTGATCTGTGGATCTACCACACACAAGGCTA
CTTCCCTGATTGGCAGAACTACACACCAGGACCAGGGATCAGATATCCACTGACCTTTGGATGGCGCTACAAGCTAGTAC
CAGTTGAGCCAGAGAAGTTAGAAGAAGCCAACAAAGGAGAGAACACCAGCTTGTTACACCCTGTGAGCCTGCATGGAATG
GATGACCCGGAGAGAGAAGTGTTAGAGTGGAGGTTTGACAGCCGCCTAGCATTTCATCACGTGGCCCGAGAGCTGCATCC
GGAGTACTTCAAGAACTGCTGATATCGAGCTTGCTACAAGGGACTTTCCGCTGGGGACTTTCCAGGGAGGCGTGGCCTGG
GCGGGACTGGGGAGTGGCGAGCCCTCAGATCCTGCATATAAGCAGCTGCTTTTTGCCTGTACTGGGTCTCTCTGGTTAGA
CCAGATCTGAGCCTGGGAGCTCTCTGGCTAACTAGGGAACCCACTGCTTAAGCCTCAATAAAGCTTGCCTTGAGTGCTTC
AAGTAGTGTGTGCCCATCTGTTGTGTGACTCTGGTAACTAGAGATCCCTCA
>1-52envLTR
--------------------------------------------------------------------------------
--------------------------------------------------------------------------------
--------------------------------------------------------------------------------
--------------------------------------------------------------------------------
--------------------------------------------------------------------------------
--------------------------------------------------------------------------------
--------------------------------------------------------------------------------
--------------------------------------------------------------------------------
--------------------------------------------------------------------------------
--------------------------------------------------------------------------------
--------------------------------------------------------------------------------
--------------------------------------------------------------------------------
--------------------------------------------------------------------------------
--------------------------------------------------------------------------------
--------------------------------------------------------------------------------
--------------------------------------------------------------------------------
--------------------------------------------------------------------------------
--------------------------------------------------------------------------------
--------------------------------------------------------------------------------
--------------------------------------------------------------------------------
--------------------------------------------------------------------------------
--------------------------------------------------------------------------------
--------------------------------------------------------------------------------
--------------------------------------------------------------------------------
--------------------------------------------------------------------------------
--------------------------------------------------------------------------------
--------------------------------------------------------------------------------
--------------------------------------------------------------------------------
--------------------------------------------------------------------------------
--------------------------------------------------------------------------------
--------------------------------------------------------------------------------
--------------------------------------------------------------------------------
--------------------------------------------------------------------------------
--------------------------------------------------------------------------------
--------------------------------------------------------------------------------
--------------------------------------------------------------------------------
--------------------------------------------------------------------------------
--------------------------------------------------------------------------------
--------------------------------------------------------------------------------
--------------------------------------------------------------------------------
--------------------------------------------------------------------------------
--------------------------------------------------------------------------------
```


--GACCTG
GAGGAGGAGATATGAGGGACAATTGGAGAAGTGAATTATATAAATATAAAGTAGTAAAAATTGAACCATTAGGAGTAGCA
CCCACCAAGGCAAAGAGAAGAGTGGTGCAGAGAGAAAAAAGAGCAGTGGGAATAGGAGCTTTGTTCCTTGGGTTCTTGGG
AGCAGCAGGAAGCACTATGGGCGCAGCGTCAATGACGCTGACGGTACAGGCCAGACAATTATTGTCTGGTATAGTGCAGC
AGCAGAACAATTTGCTGAGGGCTATTGAGGCGCAACAGCATCTGTTGCAACTCACAGTCTGGGGCATCAAGCAGCTCCAG
GCAAGAATCCTGGCTGTGGAAAGATACCTAAAGGATCAACAGCTCCTGGGGATTTGGGGTTGCTCTGGAAAACTCATTTG
CACCACTGCTGTGCCTTGGAATGCTAGTTGGAGTAATAAATCTCTGGAACAGATTTGGAATCACACGACCTGGATGGAGT
GGGACAGAGAAATTAACAATTACACAAGCTTAATACACTCCTTAATTGAAGAATCGCAAAACCAGCAAGAAAAGAATGAA
CAAGAATTATTGGAATTAGATAAATGGGCAAGTTTGTGGAATTGGTTTAACATAACAAATTGGCTGTGGTATATAAAATT
ATTCATAATGATAGTAGGAGGCTTGGTAGGTTTAAGAATAGTTTTTGCTGTACTTTCTGTAGTGAATAGAGTTAGGCAGG
GATATTCACCATTATCGTTTCAGACCCACCTCCCAATCCCGAGGGGACCCGACAGGCCCGAAGGAATAGAAGAAGAAGGT
GGAGAGAGAGACAGAGACAGATCCATTCGATTAGTGAACGGATCCTTAGCACTTATCTGGGACGATCTGCGGAGCCTGTG
CCTCTTCAGCTACCACCGCTTGAGAGACTTACTCTTGATTGTAACGAGGATTGTGGAACTTCTGGGACGCAGGGGGTGGG
AAGCCCTCAAATATTGGTGGAATCTCCTACAATATTGGAGTCAGGAGCTAAAGAATAGTGCTGTTAGCTTGCTCAATGCC
ACAGCTATAGCAGTAGCTGAGGGGACAGATAGGGTTATAGAAGTAGTACAAGAAGCTTATAGAGCTATTCGCCACATACC
TAGAAGAATAAGACAGGGCTTGGAAAGGATTTTGCTATAAGATGGGTGGCAAGTGGTCAAAAAGTAGTGTGGTTGGATGG
CCTGCTGTAAGGGAAAGAATGAGACGAGCTGAGCCAGCAGCAGATGGGGTGGGAGCAGCATCTCGAGACCTAGAAAAACA
TGGAGCAATCACAAGTAGCAACACAGCAGCTAACAATGCTGCTTGTGCCTGGCTAGAAGCACAAGAGGAGGAGAAGGTGG
GTTTTCCAGTCACACCTCAGGTACCTTTAAGACCAATGACTTACAAGGCAGCTGTAGATCTTAGCCACTTTTTAAAAGAA
AAGGGGGGACTGGAAGGGCTAATTCACTCCCAACGAAGACAAGATATCCTTGATCTGTGGATCTACCACACACAAGGCTA
CTTCCCTGATTGGCAGAACTACACACCAGGACCAGGGATCAGATATCCACTGACCTTTGGATGGCGCTACAAGCTAGTAC
CAGTTGAGCCAGAGAAGTTAGAAGAAGCCAACAAAGGAGAGAACACCAGCTTGTTACACCCTGTGAGCCTGCATGGAATG
GATGACCCGGAGAGAGAAGTGTTAGAGTGGAGGTTTGACAGCCGCCTAGCATTTCATCACGTGGCCCGAGAGCTGCATCC
GGAGTACTTCAAGAACTGCTGATATCGAGCTTGCTACAAGGGACTTTCCGCTGGGGACTTTCCAGGGAGGCGTGGCCTGG
GCGGGACTGGGGAGTGGCGAGCCCTCAGATCCTGCATATAAGCAGCTGCTTTTTGCCTGTACTGGGTCTCTCTGGTTAGA
CCAGATCTGAGCCTGGGAGCTCTCTGGCTAACTAGGGAACCCACTGCTTAAGCCTCAATAAAGCTTGCCTTGAGTGCTTC
AAGTAGTGTGTGCCCATCTGTTGTGTGACTCTGGTAACTAGAGATCCCTCA
>1-57envLTR


```
--------------------------------------------------------------------------------
--------------------------------------------------------------------------------
--------------------------------------------------------------------------------
--------------------------------------------------------------------------------
--------------------------------------------------------------------------------
--------------------------------------------------------------------------------
--------------------------------------------------------------------------------
--------------------------------------------------------------------------------
--------------------------------------------------------------------------------
--------------------------------------------------------------------------------
--------------------------------------------------------------------------------
--------------------------------------------------------------------------------
--------------------------------------------------------------------------------
--------------------------------------------------------------------------------
--------------------------------------------------------------------------------
--------------------------------------------------------------------------------
--------------------------------------------------------------------------------
--------------------------------------------------------------------------------
--------------------------------------------------------------------------------
--------------------------------------------------------------------------------
--------------------------------------------------------------------------------
--------------------------------------------------------------------------------
--------------------------------------------------------------------------------
--------------------------------------------------------------------------------
--------------------------------------------------------------------------------
--------------------------------------------------------------------------------
--------------------------------------------------------------------------------
--------------------------------------------------------------------------------
--------------------------------------------------------------------------------
--------------------------------------------------------------------------------
--------------------------------------------------------------------------------
--------------------------------------------------------------------------------
--------------------------------------------------------------------------------
--------------------------------------------------------------------------------
--------------------------------------------------------------------------------
--------------------------------------------------------------------------------
--------------------------------------------------------------------------------
--------------------------------------------------------------------------------
--------------------------------------------------------------------------------
--------------------------------------------------------------------------------
--------------------------------------------------------------------------------
--------------------------------------------------------------------------------
--------------------------------------------------------------------------------
--------------------------------------------------------------------------------
--------------------------------------------------------------------------------
--------------------------------------------------------------------------------
--------------------------------------------------------------------------------
--------------------------------------------------------------------------------
--------------------------------------------------------------------------------
--------------------------------------------------------------------------------
--------------------------------------------------------------------------------
--------------------------------------------------------------------------------
--------------------------------------------------------------------------------
--------------------------------------------------------------------------------
--------------------------------------------------------------------------------
--------------------------------------------------------------------------------
--------------------------------------------------------------------------------
--------------------------------------------------------------------------------
--------------------------------------------------------------------------------
--------------------------------------------------------------------------------
--------------------------------------------------------------------------------
--------------------------------------------------------------------------------
--------------------------------------------------------------------------------
--------------------------------------------------------------------------------
--------------------------------------------------------------------------------
--------------------------------------------------------------------------------
--------------------------------------------------------------------------------
--------------------------------------------------------------------------------
--------------------------------------------------------------------------------
--------------------------------------------------------------------------------
--------------------------------------------------------------------------------
--------------------------------------------------------------------------------
--------------------------------------------------------------------------------
--------------------------------------------------------------------------------
--------------------------------------------------------------------------------
--------------------------------------------------------------------------------
--------------------------------------------------------------------------------
--------------------------------------------------------------------------------
--------------------------------------------------------------------------------
--------------------------------------------------------------------------------
--------------------------------------------------------------------------------
-------------GAGGGACAATTGGAGAAGTGAATTATATAAATATAAAGTAATAAAAATTGAACCATTAGGAGTAGCA
CCCACCAAGGCAAAGAGAAGAGTGGTGCAGAGAGAAAAAAGAGCAGTGGGAATAGGAGCTTTGTTCCTTGGGTTCTTGGG
AGCAGCAGGAAGCACTATGGGCGCAGCGTCAATGACGCTGACGGTACAGGCCAGACAATTATTGTCTGGTATAGTGCAGC
AGCAGAACAATTTGCTGAGGGCTATTGAGGCGCAACAGCATCTGTTGCAACTCACAGTCTGGGGCATCAAGCAGCTCCAG
GCAAGAATCCTGGCTGTGGAAAGATACCTAAAGGATCAACAGCTCCTGGGGATTTGGGGTTGCTCTGGAAAACTCATTTG
CACCACTGCTGTGCCTTGGAATGCTAGTTGGAGTAATAAATCTCTGGAACAGATTTGGAATCACACGACCTGGATGGAGT
GGGACAGAGAAATTAACAATTACACAAGCTTAATACACTCCTTAATTGAAGAATCGCAAAACCAGCAAGAAAAGAATGAA
CAAGAATTATTGGAATTAGATAAATGGGCAAGTTTGTGGAATTGGTTTAACATAACAAATTGGCTGTGGTATATAAAATT
ATTCATAATGATAGTAGGAGGCTTGGTAGGTTTAAGAATAGTTTTTGCTGTACTTTCTGTAGTGAATAGAGTTAGGCAGG
GATATTCACCATTATCGTTTCAGACCCACCTCCCAATCCCGAGGGGACCCGACAGGCCCGAAGGAATAGAAGAAGAAGGT
GGAGAGAGAGACAGAGACAGATCCATTCGATTAGTGAACGGATCCTTAGCACTTATCTGGGACGATCTGCGGAGCCTGTG
CCTCTTCAGCTACCACCGCTTGAGAGACTTACTCTTGATTGTAACGAGGATTGTGGAACTTCTGGGACGCAGGGGGTGGG
AAGCCCTCAAATATTGGTGGAATCTCCTACAATATTGGAGTCAGGAGCTAAAGAATAGTGCTGTTAGCTTGCTCAATGCC
```

ACAGCTATAGCAGTAGCTGAGGGGACAGATAGGGTTATAGAAGTAGTACAAGAAGCTTATAGAGCTATTCGCCACATACC
TAGAAGAATAAGACAGGGCTTGGAAAGGATTTTGCTATAAGATGGGTGGCAAGTGGTCAAAAAGTAGTGTGGTTGGATGG
CCTGCTGTAAGGGAAAGAATGAGACGAGCTGAGCCAGCAGCAGATGGGGTGGGAGCAGCATCTCGAGACCTAGAAAAACA
TGGAGCAATCACAAGTAGCAACACAGCAGCTAACAATGCTGCTTGTGCCTGGCTAGAAGCACAAGAGGAGGAGAAGGTGG
GTTTTCCAGTCACACCTCAGGTACCTTTAAGACCAATGACTTACAAGGCAGCTGTAGATCTTAGCCACTTTTTAAAAGAA
AAGGGGGGACTGGAAGGGCTAATTCACTCCCAACGAAGACAAGATATCCTTGATCTGTGGATCTACCACACACAAGGCTA
CTTCCCTGATTGGCAGAACTACACACCAGGACCAGGGATCAGATATCCACTGACCTTTGGATGGCGCTACAAGCTAGTAC
CAGTTGAGCCAGAGAAGTTAGAAGAAGCCAACAAAGGAGAGAACACCAGCTTGTTACACCCTGTGAGCCTGCATGGAATG
GATGACCCGGAGAGAGAAGTGTTAGAGTGGAGGTTTGACAGCCGCCTAGCATTTCATCACGTGGCCCGAGAGCTGCATCC
GGAGTACTTCAAGAACTGCTGATATCGAGCTTGCTACAAGGGACTTTCCGCTGGGGACTTTCCAGGGAGGCGTGGCCTGG
GCGGGACTGGGGAGTGGCGAGCCCTCAGATCCTGCATATAAGCAGCTGCTTTTTGCCTGTACTGGGTCTCTCTGGTTAGA
CCAGATCTGAGCCTGGGAGCTCTCTGGCTAACTAGGGAACCCACTGCTTAAGCCTCAATAAAGCTTGCCTTGAGTGCTTC
AAGTAGTGTGTGCCCATCTGTTGTGTGACTCTGGTAACTAGAGATCCCTCA

>1-60envLTR

-------------------GGGCTGCTATTAACAAGAGATGGTGGTAATAACAACAATGGGTCCGAGATCTTCAGACCTG
GAGGAGGAGATATGAGGGACAATTGGAGAAGTGAATTATATAAATATAAAGTAGTAAAAATTGAACCATTAGGAGTAGCA
CCCACCAAGGCAAAGAGAAGAGTGGTGCAGAGAGAAAAAAGAGCAGTGGGAATAGGAGCTTTGTTCCTTGGGTTCTTGGG
AGCAGCAGGAAGCACTATGGGCGCAGCGTCAATGACGCTGACGGTACAGGCCAGACAATTATTGTCTGGTATAGTGCAGC
AGCAGAACAATTTGCTGAGGGCTATTGAGGCGCAACAGCATCTGTTGCAACTCACAGTCTGGGGCATCAAGCAGCTCCAG
GCAAGAATCCTGGCTGTGGAAAGATACCTAAAGGATCAACAGCTCCTGGGGATTTGGGGTTGCTCTGGAAAACTCATTTG
CACCACTGCTGTGCCTTGGAATGCTAGTTGGAGTAATAAATCTCTGGAACAGATTTGGAATCACACGACCTGGATGGAGT
GGGACAGAGAAATTAACAATTACACAAGCTTAATACACTCCTTAATTGAAGAATCGCAAAACCAGCAAGAAAAGAATGAA
CAAGAATTATTGGAATTAGATAAATGGGCAAGTTTGTGGAATTGGTTTAACATAACAAATTGGCTGTGGTATATAAAATT
ATTCATAATGATAGTAGGAGGCTTGGTAGGTTTAAGAATAGTTTTTGCTGTACTTTCTGTAGTGAATAGAGTTAGGCAGG
GATATTCACCATTATCGTTTCAGACCCACCTCCCAATCCCGAGGGGACCCGACAGGCCCGAAGGAATAGAAGAAGAAGGT
GGAGAGAGAGACAGAGACAGATCCATTCGATTAGTGAACGGATCCTTAGCACTTATCTGGGACGATCTGCGGAGCCTGTG
CCTCTTCAGCTACCACCGCTTGAGAGACTTACTCTTGATTGTAACGAGGATTGTGGAACTTCTGGGACGCAGGGGGTGGG
AAGCCCTCAAATATTGGTGGAATCTCCTACAATATTGGAGTCAGGAGCTAAAGAATAGTGCTGTTAGCTTGCTCAATGCC
ACAGCTATAGCAGTAGCTGAGGGGACAGATAGGGTTATAGAAGTAGTACAAGAAGCTTATAGAGCTATTCGCCACATACC
TAGAAGAATAAGACAGGGCTTGGAAAGGATTTTGCTATAAGATGGGTGGCAAGTGGTCAAAAAGTAGTGTGGTTGGATGG
CCTGCTGTAAGGGAAAGAATGAGACGAGCTGAGCCAGCAGCAGATGGGGTGGGAGCAGCATCTCGAGACCTAGAAAAACA
TGGAGCAATCACAAGTAGCAACACAGCAGCTAACAATGCTGCTTGTGCCTGGCTAGAAGCACAAGAGGAGGAGAAGGTGG
GTTTTCCAGTCACACCTCAGGTACCTTTAAGACCAATGACTTACAAGGCAGCTGTAGATCTTAGCCACTTTTTAAAAGAA
AAGGGGGGACTGGAAGGGCTAATTCACTCCCAACGAAGACAAGATATCCTTGATCTGTGGATCTACCACACACAAGGCTA
CTTCCCTGATTGGCAGAACTACACACCAGGACCAGGGATCAGATATCCACTGACCTTTGGATGGCGCTACAAGCTAGTAC
CAGTTGAGCCAGAGAAGTTAGAAGAAGCCAACAAAGGAGAGAACACCAGCTTGTTACACCCTGTGAGCCTGCATGGAATG
GATGACCCGGAGAGAGAAGTGTTAGAGTGGAGGTTTGACAGCCGCCTAGCATTTCATCACGTGGCCCGAGAGCTGCATCC
GGAGTACTTCAAGAACTGCTGATATCGAGCTTGCTACAAGGGACTTTCCGCTGGGGACTTTCCAGGGAGGCGTGGCCTGG
GCGGGACTGGGGAGTGGCGAGCCCTCAGATCCTGCATATAAGCAGCTGCTTTTTGCCTGTACTGGGTCTCTCTGGTTAGA
CCAGATCTGAGCCTGGGAGCTCTCTGGCTAACTAGGGAACCCACTGCTTAAGCCTCAATAAAGCTTGCCTTGAGTGCTTC
AAGTAGTGTGTGCCCATCTGTTGTGTGACTCTGGTAACTAGAGATCCCTCA

>1-63envLTR

---ACCTG
GAGGAGGAGATATGAGGGACAATTGGAGAAGTGAATTATATAAATATAAAGTAGTAAAAATTGAACCATTAGGAGTAGCA
CCCACCAAGGCAAAGAGAAGAGTGGTGCAGAGAGAAAAAAGAGCAGTGGGAATAGGAGCTTTGTTCCTTGGGTTCTTGGG
AGCAGCAGGAAGCACTATGGGCGCAGCGTCAATGACGCTGACGGTACAGGCCAGACAATTATTGTCTGGTATAGTGCAGC
AGCAGAACAATTTGCTGAGGGCTATTGAGGCGCAACAGCATCTGTTGCAACTCACAGTCTGGGGCATCAAGCAGCTCCAG
GCAAGAATCCTGGCTGTGGAAAGATACCTAAAGGATCAACAGCTCCTGGGGATTTGGGGTTGCTCTGGAAAACTCATTTG
CACCACTGCTGTGCCTTGGAATGCTAGTTGGAGTAATAAATCTCTGGAACAGATTTGGAATCACACGACCTGGATGGAGT
GGGACAGAGAAATTAACAATTACACAAGCTTAATACACTCCTTAATTGAAGAATCGCAAAACCAGCAAGAAAAGAATGAA
CAAGAATTATTGGAATTAGATAAATGGGCAAGTTTGTGGAATTGGTTTAACATAACAAATTGGCTGTGGTATATAAAATT
ATTCATAATGATAGTAGGAGGCTTGGTAGGTTTAAGAATAGTTTTTGCTGTACTTTCTGTAGTGAATAGAGTTAGGCAGG
GATATTCACCATTATCGTTTCAGACCCACCTCCCAATCCCGAGGGGACCCGACAGGCCCGAAGGAATAGAAGAAGAAGGT
GGAGAGAGAGACAGAGACAGATCCATTCGATTAGTGAACGGATCCTTAGCACTTATCTGGGACGATCTGCGGAGCCTGTG
CCTCTTCAGCTACCACCGCTTGAGAGACTTACTCTTGATTGTAACGAGGATTGTGGAACTTCTGGGACGCAGGGGGTGGG
AAGCCCTCAAATATTGGTGGAATCTCCTACAATATTGGAGTCAGGAGCTAAAGAATAGTGCTGTTAGCTTGCTCAATGCC
ACAGCTATAGCAGTAGCTGAGGGGACAGATAGGGTTATAGAAGTAGTACAAGAAGCTTATAGAGCTATTCGCCACATACC
TAGAAGAATAAGACAGGGCTTGGAAAGGATTTTGCTATAAGATGGGTGGCAAGTGGTCAAAAAGTAGTGTGGTTGGATGG
CCTGCTGTAAGGGAAAGAATGAGACGAGCTGAGCCAGCAGCAGATGGGGTGGGAGCAGCATCTCGAGACCTAGAAAAACA
TGGAGCAATCACAAGTAGCAACACAGCAGCTAACAATGCTGCTTGTGCCTGGCTAGAAGCACAAGAGGAGGAGAAGGTGG
GTTTTCCAGTCACACCTCAGGTACCTTTAAGACCAATGACTTACAAGGCAGCTGTAGATCTTAGCCACTTTTTAAAAGAA
AAGGGGGGACTGGAAGGGCTAATTCACTCCCAACGAAGACAAGATATCCTTGATCTGTGGATCTACCACACACAAGGCTA
CTTCCCTGATTGGCAGAACTACACACCAGGACCAGGGATCAGATATCCACTGACCTTTGGATGGCGCTACAAGCTAGTAC
CAGTTGAGCCAGAGAAGTTAGAAGAAGCCAACAAAGGAGAGAACACCAGCTTGTTACACCCTGTGAGCCTGCATGGAATG
GATGACCCGGAGAGAGAAGTGTTAGAGTGGAGGTTTGACAGCCGCCTAGCATTTCATCACGTGGCCCGAGAGCTGCATCC
GGAGTACTTCAAGAACTGCTGATATCGAGCTTGCTACAAGGGACTTTCCGCTGGGGACTTTCCAGGGAGGCGTGGCCTGG
GCGGGACTGGGGAGTGGCGAGCCCTCAGATCCTGCATATAAGCAGCTGCTTTTTGCCTGTACTGGGTCTCTCTGGTTAGA
CCAGATCTGAGCCTGGGAGCTCTCTGGCTAACTAGGGAACCCACTGCTTAAGCCTCAATAAAGCTTGCCTTGAGTGCTTC
AAGTAGTGTGTGCCCATCTGTTGTGTGACTCTGGTAACTAGAGATCCCTCA
>2-1envLTR

-----------------------------TAACAAGAGATGGTGGTAATAACAACAATGGGTCCGAGATCTTCAGACCTG
GAGGAGGAGATATGAGGGACAATTGGAGAAGTGAATTATATAAATATAAAGTAGTAAAAATTGAACCATTAGGAGTAGCA
CCCACCAAGGCAAAGAGAAGAGTGGTGCAGAGAGAAAAAAGAGCAGTGGGAATAGGAGCTTTGTTCCTTGGGTTCTTGGG
AGCAGCAGGAAGCACTATGGGCGCAGCGTCAATGACGCTGACGGTACAGGCCAGACAATTATTGTCTGGTATAGTGCAGC
AGCAGAACAATTTGCTGAGGGCTATTGAGGCGCAACAGCATCTGTTGCAACTCACAGTCTGGGGCATCAAGCAGCTCCAG
GCAAGAATCCTGGCTGTGAAAAGATACCTAAAGGATCAACAGCTCCTGGGGATTTGGGGTTGCTCTGGAAAACTCATTTG
CACCACTGCTGTGCCTTGGAATGCTAGTTGGAGTAATAAATCTCTGGAACAGATTTGGAATCACACGACCTGGATGGAGT
GGGACAGAGAAATTAACAATTACACAAGCTTAATACACTCCTTAATTGAAGAATCGCAAAACCAGCAAGAAAAGAATGAA
CAAGAATTATTGGAATTAGATAAATGGGCAAGTTTGTGGAATTGGTTTAACATAACAAATTGGCTGTGGTATATAAAATT
ATTCATAATGATAGTAGGAGGCTTGGTAGGTTTAAGAATAGTTTTTGCTGTACTTTCTGTAGTGAATAGAGTTAGGCAGG
GATATTCACCATTATCGTTTCAGACCCACCTCCCAATCCCGAGGGGACCCGACAGGCCCGAAGGAATAGAAGAAGAAGGT
GGAGAGAGAGACAGAGACAGATCCATTCGATTAGTGAACGGATCCTTAGCACTTATCTGGGACGATCTGCGGAGCCTGTG
CCTCTTCAGCTACCACCGCTTGAGAGACTTACTCTTGATTGTAACGAGGATTGTGGAACTTCTGGGACGCAGGGGGTGGG
AAGCCCTCAAATATTGGTGGAATCTCCTACAATATTGGAGTCAGGAGCTAAAGAATAGTGCTGTTAGCTTGCTCAATGCC
ACAGCTATAGCAGTAGCTGAGGGGACAGATAGGGTTATAGAAGTAGTACAAGAAGCTTATAGAGCTATTCGCCACATACC
TAGAAGAATAAGACAGGGCTTGGAAAGGATTTTGCTATAAGATGGGTGGCAAGTGGTCAAAAAGTAGTGTGGTTGGATGG
CCTGCTGTAAGGGAAAGAATGAGACGAGCTGAGCCAGCAGCAGATGGGGTGGGAGCAGCATCTCGAGACCTAGAAAAACA
TGGAGCAATCACAAGTAGCAACACAGCAGCTAACAATGCTGCTTGTGCCTGGCTAGAAGCACAAGAGGAGGAGAAGGTGG
GTTTTCCAGTCACACCTCAGGTACCTTTAAGACCAATGACTTACAAGGCAGCTGTAGATCTTAGCCACTTTTTAAAAGAA
AAGGGGGGACTGGAAGGGCTAATTCACTCCCAACGAAGACAAGATATCCTTGATCTGTGGATCTACCACACACAAGGCTA
CTTCCCTGATTGGCAGAACTACACACCAGGACCAGGGATCAGATATCCACTGACCTTTGGATGGCGCTACAAGCTAGTAC
CAGTTGAGCCAGAGAAGTTAGAAGAAGCCAACAAAGGAGAGAACACCAGCTTGTTACACCCTGTGAGCCTGCATGGAATG
GATGACCCGGAGAGAGAAGTGTTAGAGTGGAGGTTTGACAGCCGCCTAGCATTTCATCACGTGGCCCGAGAGCTGCATCC
GGAGTACTTCAAGAACTGCTGATATCGAGCTTGCTACAAGGGACTTTCCGCTGGGGACTTTCCAGGGAGGCGTGGCCTGG

```
GCGGGACTGGGGAGTGGCGAGCCCTCAGATCCTGCATATAAGCAGCTGCTTTTTGCCTGTACTGGGTCTCTCTGGTTAGA
CCAGATCTGAGCCTGGGAGCTCTCTGGCTAACTAGGGAACCCACTGCTTAAGCCTCAATAAAGCTTGCCTTGAGTGCTTC
AAGTAGTGTGTGCCCATCTGTTGTGTGACTCTGGTAACTAGAGATCCCTCA
>2-2envLTR
--------------------------------------------------------------------------------
--------------------------------------------------------------------------------
--------------------------------------------------------------------------------
--------------------------------------------------------------------------------
--------------------------------------------------------------------------------
--------------------------------------------------------------------------------
--------------------------------------------------------------------------------
--------------------------------------------------------------------------------
--------------------------------------------------------------------------------
--------------------------------------------------------------------------------
--------------------------------------------------------------------------------
--------------------------------------------------------------------------------
--------------------------------------------------------------------------------
--------------------------------------------------------------------------------
--------------------------------------------------------------------------------
--------------------------------------------------------------------------------
--------------------------------------------------------------------------------
--------------------------------------------------------------------------------
--------------------------------------------------------------------------------
--------------------------------------------------------------------------------
--------------------------------------------------------------------------------
--------------------------------------------------------------------------------
--------------------------------------------------------------------------------
--------------------------------------------------------------------------------
--------------------------------------------------------------------------------
--------------------------------------------------------------------------------
--------------------------------------------------------------------------------
--------------------------------------------------------------------------------
--------------------------------------------------------------------------------
--------------------------------------------------------------------------------
--------------------------------------------------------------------------------
--------------------------------------------------------------------------------
--------------------------------------------------------------------------------
--------------------------------------------------------------------------------
--------------------------------------------------------------------------------
--------------------------------------------------------------------------------
--------------------------------------------------------------------------------
--------------------------------------------------------------------------------
--------------------------------------------------------------------------------
--------------------------------------------------------------------------------
--------------------------------------------------------------------------------
--------------------------------------------------------------------------------
--------------------------------------------------------------------------------
--------------------------------------------------------------------------------
--------------------------------------------------------------------------------
--------------------------------------------------------------------------------
--------------------------------------------------------------------------------
--------------------------------------------------------------------------------
--------------------------------------------------------------------------------
--------------------------------------------------------------------------------
--------------------------------------------------------------------------------
--------------------------------------------------------------------------------
--------------------------------------------------------------------------------
--------------------------------------------------------------------------------
--------------------------------------------------------------------------------
--------------------------------------------------------------------------------
--------------------------------------------------------------------------------
--------------------------------------------------------------------------------
--------------------------------------------------------------------------------
--------------------------------------------------------------------------------
--------------------------------------------------------------------------------
--------------------------------------------------------------------------------
--------------------------------------------------------------------------------
--------------------------------------------------------------------------------
--------------------------------------------------------------------------------
--------------------------------------------------------------------------------
--------------------------------------------------------------------------------
--------------------------------------------------------------------------------
--------------------------------------------------------------------------------
--------------------------------------------------------------------------------
--------------------------------------------------------------------------------
--------------------------------------------------------------------------------
--------------------------------------------------------------------------------
--------------------------------------------------------------------------------
--------------------------------------------------------------------------------
--------------------------------------------------------------------------------
--------------------------------------------------------------------------------
--------------------------------------------------------------------------------
--------------------------------------------------------------------------------
--------------------------------------------------------------------------------
--------------------------------------------------------------------------------
--------------------------------------------------------------------------------
--------------------------------------------------------------------------------
--------------------------------------------------------------------------------
--------------------------------------------------------------------------------
--------------------------------------------------------------------------------
--------------------------------------------------------------------------------
--------------------------------------------------------------------------------
--------------------------------------------------------------------------------
--------------------------------------------------------------------------------
--------------------------------------------------------------------------------
```

```
--------------------------------------------------------------------------------
--------------------------------------------------------------------------------
--------------------------------------------------------------------------------
--------------------------------------------------------------------------------
------------------------------------AGATGGTGGTAATAACAACAATGGGTCCGAGATCTTCAGACCTG
GAGGAGGAGATATGAGGGACAATTGGAGAAGTGAATTATATAAATATAAAGTAGTAAAAATTGAACCATTAGGAGTAGCA
CCCACCAAGGCAAAGAGAAGAGTGGTGCAGAGAGAAAAAAGAGCAGTGGGAATAGGAGCTTTGTTCCTTGGGTTCTTGGG
AGCAGCAGGAAGCACTATGGGCGCAGCGTCAATGACGCTGACGGTACAGGCCAGACAATTATTGTCTGGTATAGTGCAGC
AGCAGAACAATTTGCTGAGGGCTATTGAGGCGCAACAGCATCTGTTGCAACTCACAGTCTGGGGCATCAAGCAGCTCCAG
GCAAGAATCCTGGCTGTGGAAAGATACCTAAAGGATCAACAGCTCCTGGGGATTTGGGGTTGCTCTGGAAAACTCATTTG
CACCACTGCTGTGCCTTGGAATGCTAGTTGGAGTAATAAATCTCTGGAACAGATTTGGAATCACACGACCTGGATGGAGT
GGGACAGAGAAATTAACAATTACACAAGCTTAATACACTCCTTAATTGAAGAATCGCAAAACCAGCAAGAAAAGAATGAA
CAAGAATTATTGGAATTAGATAAATGGGCAAGTTTGTGGAATTGGTTTAACATAACAAATTGGCTGTGGTATATAAAATT
ATTCATAATGATAGTAGGAGGCTTGGTAGGTTTAAGAATAGTTTTTGCTGTACTTTCTGTAGTGAATAGAGTTAGGCAGG
GATATTCACCATTATCGTTTCAGACCCACCTCCCAATCCCGAGGGGACCCGACAGGCCCGAAGGAATAGAAGAAGAAGGT
GGAGAGAGAGACAGAGACAGATCCATTCGATTAGTGAACGGATCCTTAGCACTTATCTGGGACGATCTGCGGAGCCTGTG
CCTCTTCAGCTACCACCGCTTGAGAGACTTACTCTTGATTGTAACGAGGATTGTGGAACTTCTGGGACGCAGGGGGTGGG
AAGCCCTCAAATATTGGTGGAATCTCCTACAATATTGGAGTCAGGAGCTAAAGAATAGTGCTGTTAGCTTGCTCAATGCC
ACAGCTATAGCAGTAGCTGAGGGGACAGATAGGGTTATAGAAGTAGTACAAGAAGCTTATAGAGCTATTCGCCACATACC
TAGAAGAATAAGACAGGGCTTGGAAAGGATTTTGCTATAAGATGGGTGGCAAGTGGTCAAAAAGTAGTGTGGTTGGATGG
CCTGCTGTAAGGGAAAGAATGAGACGAGCTGAGCCAGCAGCAGATGGGGTGGGAGCAGCATCTCGAGACCTAGAAAAACA
TGGAGCAATCACAAGTAGCAACACAGCAGCTAACAATGCTGCTTGTGCCTGGCTAGAAGCACAAGAGGAGGAGAAGGTGG
GTTTTCCAGTCACACCTCAGGTACCTTTAAGACCAATGACTTACAAGGCAGCTGTAGATCTTAGCCACTTTTTAAAAGAA
AAGGGGGGACTGGAAGGGCTAATTCACTCCCAACGAAGACAAGATATCCTTGATCTGTGGATCTACCACACACAAGGCTA
CTTCCCTGATTGGCAGAACTACACACCAGGACCAGGGATCAGATATCCACTGACCTTTGGATGGCGCTACAAGCTAGTAC
CAGTTGAGCCAGAGAAGTTAGAAGAAGCCAACAAAGGAGAGAACACCAGCTTGTTACACCCTGTGAGCCTGCATGGAATG
GATGACCCGGAGAGAGAAGTGTTAGAGTGGAGGTTTGACAGCCGCCTAGCATTTCATCACGTGGCCCGAGAGCTGCATCC
GGAGTACTTCAAGAACTGCTGATATCGAGCTTGCTACAAGGGACTTTCCGCTGGGGACTTTCCAGGGAGGCGTGGCCTGG
GCGGGACTGGGGAGTGGCGAGCCCTCAGATCCTGCATATAAGCAGCTGCTTTTTGCCTGTACTGGGTCTCTCTGGTTAGA
CCAGATCTGAGCCTGGGAGCTCTCTGGCTAACTAGGGAACCCACTGCTTAAGCCTCAATAAAGCTTGCCTTGAGTGCTTC
AAGTAGTGTGTGCCCATCTGTTGTGTGACTCTGGTAACTAGAGATCCCTCA
>2-3envLTR
--------------------------------------------------------------------------------
--------------------------------------------------------------------------------
--------------------------------------------------------------------------------
--------------------------------------------------------------------------------
--------------------------------------------------------------------------------
--------------------------------------------------------------------------------
--------------------------------------------------------------------------------
--------------------------------------------------------------------------------
--------------------------------------------------------------------------------
--------------------------------------------------------------------------------
--------------------------------------------------------------------------------
--------------------------------------------------------------------------------
--------------------------------------------------------------------------------
--------------------------------------------------------------------------------
--------------------------------------------------------------------------------
--------------------------------------------------------------------------------
--------------------------------------------------------------------------------
--------------------------------------------------------------------------------
--------------------------------------------------------------------------------
--------------------------------------------------------------------------------
--------------------------------------------------------------------------------
--------------------------------------------------------------------------------
--------------------------------------------------------------------------------
--------------------------------------------------------------------------------
--------------------------------------------------------------------------------
--------------------------------------------------------------------------------
--------------------------------------------------------------------------------
--------------------------------------------------------------------------------
--------------------------------------------------------------------------------
--------------------------------------------------------------------------------
--------------------------------------------------------------------------------
--------------------------------------------------------------------------------
--------------------------------------------------------------------------------
--------------------------------------------------------------------------------
--------------------------------------------------------------------------------
--------------------------------------------------------------------------------
--------------------------------------------------------------------------------
--------------------------------------------------------------------------------
--------------------------------------------------------------------------------
--------------------------------------------------------------------------------
--------------------------------------------------------------------------------
--------------------------------------------------------------------------------
--------------------------------------------------------------------------------
--------------------------------------------------------------------------------
--------------------------------------------------------------------------------
--------------------------------------------------------------------------------
--------------------------------------------------------------------------------
--------------------------------------------------------------------------------
--------------------------------------------------------------------------------
--------------------------------------------------------------------------------
--------------------------------------------------------------------------------
--------------------------------------------------------------------------------
--------------------------------------------------------------------------------
--------------------------------------------------------------------------------
--------------------------------------------------------------------------------
--------------------------------------------------------------------------------
--------------------------------------------------------------------------------
--------------------------------------------------------------------------------
--------------------------------------------------------------------------------
--------------------------------------------------------------------------------
--------------------------------------------------------------------------------
--------------------------------------------------------------------------------
```

```
--------------------------------------------------------------------------------
--------------------------------------------------------------------------------
--------------------------------------------------------------------------------
--------------------------------------------------------------------------------
--------------------------------------------------------------------------------
--------------------------------------------------------------------------------
--------------------------------------------------------------------------------
--------------------------------------------------------------------------------
--------------------------------------------------------------------------------
--------------------------------------------------------------------------------
--------------------------------------------------------------------------------
--------------------------------------------------------------------------------
--------------------------------------------------------------------------------
--------------------------------------------------------------------------------
--------------------------------------------------------------------------------
--------------------------------------------------------------------------------
--------------------------------------------------------------------------------
--------------------------------------------------------------------------------
--------------------------------------------------------------------------------
--------------------------------------------------------------------------------
--------------------------------------------------------------------------------
--------------------------------------------------------------------------------
--------------------------------------------------------------------------------
--------------------------------------------------------------------------------
--------------------------------------------------------------------------------
--------------------------------------------------------------------------------
--------------------------------------------------------------------------------
--------------------------------------------------------------------------------
--------------------------------------------------------------------------------
--------------------------------------------------------------------------------
--------------------------------------------------------------------------------
--------------------------------------------------------------------------------
------------------------------AACAAGAGATGGTGGTAATAACAACAATAGGTCCGAGATCTTCAGACCTG
GAAGAGGAGATATGAAGGACAATTGGAGAAGTGAATTATATAAATATAAAGTAGTAAAAATTGAACCATTAGGAGTAGCA
CCCACCAAGGCAAAGAGAAGAGTGGTGCAGAGAGAAAAAAGAGCAGTAGGAATAGGAGCTTTGTTCCTTAGGTTCTTAGG
AGCAGCAGGAAGCACTATGGGCGCAGCGTCAATGACGCTGACGGTACAGGCCAGACAATTATTGTCTGGTATAGTGCAGC
AGCAGAACAATTTGCTGAGGGCTATTAAGGCGCAACAGCATCTGTTGCAACTCACAGTCTAGGGCATCAAGCAGCTCCAG
GCAAGAATCCTGGCTGTGGAAAGATACCTAAAGGATCAACAGCTCCTGGGGATTTGGGGTTGCTCTAGAAAACTCATTTG
CACCACTGCTGTGCCTTGGAATGCTAGTTGGAGTAATAAATCTCTGGAACAGATTTAGAATCACACGACCTGGATGAAGT
AGGACAGAGAAATTAACAATTACACAAGCTTAATACACTCCTTAATTGAAGAATCGCAAAACCAGCAAGAAAAGAATGAA
CAAGAATTATTAGAATTAGATAAATGGGCAAGTTTGTGGAATTGGTTTAACATAACAAATTGGCTGTGGTATATAAAATT
ATTCATAATGATAGTAGGAGGCTTGGTAAGTTTAAGAATAGTTTTTGCTGTACTTTCTGTAGTGAATAGAGTTAGGCAAA
GATATTCACCATTATCGTTTCAGACCCACCTCCCAATCCCGAGGGGACCCGACAGGCCCGAAGGAATAGAAGAAGAAGGT
AGAGAGAGAGACAGAGACAGATCCATTCGATTAGTGAACGGATCCTTAGCACTTATCTAGGACGATCTGCGGAGCCTGTG
CCTCTTCAGCTACCACCGCTTGAGAGACTTACTCTTGATTGTAACGAGGATTGTAGAACTTCTAGGACGCAGGGGGTAGG
AAGCCCTCAAATATTGGTAGAATCTCCTACAATATTGGAGTCAGGAGCTAAAGAATAGTGCTGTTAGCTTGCTCAATGCC
ACAGCTATAGCAGTAGCTGAGAGGACAGATAGGGTTATAGAAGTAGTACAAGAAGCTTATAGAGCTATTCGCCACATACC
TAGAAGAATAAGACAGGGCTTGGAAAGGATTTTGCTATAAGATGGGTGGCAAGTGGTCAAAAAGTAGTGTGGTTGGATGG
CCTGCTGTAAGGGAAAGAATGAGACGAGCTGAGCCAGCAGCAGATGGGGTGGGAGCAGCATCTCGAGACCTAGAAAAACA
TGGAGCAATCACAAGTAGCAACACAGCAGCTAACAATGCTGCTTGTGCCTGGCTAGAAGCCCAAGAGGAGGAGAAGGTGG
GTTTTCCAGTCACACCTCAGGTACCTTTAAGACCAATGACTTACAAGGCAGCTGTAGATCTTAGCCACTTTTTAAAAGAA
AAGGGGGGACTGGAAGGGCTAATTCACTCCCAACGAAGACAAGATATCCTTGATCTGTGGATCTACCACACACAAGGCTA
CTTCCCTGATTGGCAGAACTACACACCAGGACCAGGGATCAGATATCCACTGACCTTTGGATGGCGCTACAAGCTAGTAC
CAGTTGAGCCAGAGAAGTTAGAAGAAGCCAACAAAGGAGAGAACACCAGCTTGTTACACCCTGTGAGCCTGCATGGAATG
GATGACCCGGAGAGAGAAGTGTTAGAGTGGAGGTTTGACAGCCGCCTAGCATTTCATCACGTGGCCCGAGAGCTGCATCC
GGAGTACTTCAAGAACTGCTGATATCGAGCTTGCTACAAGGGACTTTCCGCTAGGGACTTTCCAGGGAGGCGTGGCCTGG
GCAGGACTGGGGAGTGGCGAGCCCTCAGATCCTGCATATAAGCAGCTGCTTTTTGCCTGTACTGGGTCTCTCTGGTTAGA
CCAGATCTGAGCCTGGGAGCTCTCTGGCTAACTAGGGAACCCACTGCTTAAGCCTCAATAAAGCTTGCCTTGAGTGCTTC
                AAGTAGTGTGTGCCCATCTGTTGTGTGACTCTGGTAACTAGAGATCCCTCA
                                   >2-6envLTR
--------------------------------------------------------------------------------
--------------------------------------------------------------------------------
--------------------------------------------------------------------------------
--------------------------------------------------------------------------------
--------------------------------------------------------------------------------
--------------------------------------------------------------------------------
--------------------------------------------------------------------------------
--------------------------------------------------------------------------------
--------------------------------------------------------------------------------
--------------------------------------------------------------------------------
--------------------------------------------------------------------------------
--------------------------------------------------------------------------------
--------------------------------------------------------------------------------
--------------------------------------------------------------------------------
--------------------------------------------------------------------------------
--------------------------------------------------------------------------------
--------------------------------------------------------------------------------
--------------------------------------------------------------------------------
--------------------------------------------------------------------------------
--------------------------------------------------------------------------------
--------------------------------------------------------------------------------
--------------------------------------------------------------------------------
--------------------------------------------------------------------------------
--------------------------------------------------------------------------------
--------------------------------------------------------------------------------
--------------------------------------------------------------------------------
--------------------------------------------------------------------------------
--------------------------------------------------------------------------------
--------------------------------------------------------------------------------
--------------------------------------------------------------------------------
--------------------------------------------------------------------------------
--------------------------------------------------------------------------------
--------------------------------------------------------------------------------
--------------------------------------------------------------------------------
```

```
--------------------------------------------------------------------------------
--------------------------------------------------------------------------------
--------------------------------------------------------------------------------
--------------------------------------------------------------------------------
--------------------------------------------------------------------------------
--------------------------------------------------------------------------------
--------------------------------------------------------------------------------
--------------------------------------------------------------------------------
--------------------------------------------------------------------------------
--------------------------------------------------------------------------------
--------------------------------------------------------------------------------
--------------------------------------------------------------------------------
--------------------------------------------------------------------------------
--------------------------------------------------------------------------------
--------------------------------------------------------------------------------
--------------------------------------------------------------------------------
--------------------------------------------------------------------------------
--------------------------------------------------------------------------------
--------------------------------------------------------------------------------
--------------------------------------------------------------------------------
--------------------------------------------------------------------------------
--------------------------------------------------------------------------------
--------------------------------------------------------------------------------
--------------------------------------------------------------------------------
--------------------------------------------------------------------------------
--------------------------------------------------------------------------------
--------------------------------------------------------------------------------
--------------------------------------------------------------------------------
--------------------------------------------------------------------------------
--------------------------------------------------------------------------------
--------------------------------------------------------------------------------
--------------------------------------------------------------------------------
--------------------------------------------------------------------------------
--------------------------------------------------------------------------------
--------------------------------------------------------------------------------
--------------------------------------------------------------------------------
--------------------------------------------------------------------------------
--------------------------------------------------------------------------------
--------------------------------------------------------------------------------
--------------------------------------------------------------------------------
--------------------------------------------------------------------------------
--------------------------------------------------------------------------------
--------------------------------------------------------------------------------
--------------------------------------------------------------------------------
--------------------------------------------------------------------------------
--------------------------------------------------------------------------------
--------------------------------------------------------------------------------
--------------------------------------------------------------------------------
--------------------------------------------------------------------------------
--------------------------------------------------------------------------------
--------------------------------------------------------------------------------
--------------------------------------------------------------------------------
--------------------------------------------------------------------------------
--------------------------------------------------------------------------------
--------------------------------------------------------------------------------
--------------------------------------------------------------------------------
--------------------------------------------------------------------------------
--------------------------------------------------------------------------------
--------------------------------------------------------------------------------
--------------------------------------------------------------------------------
------------------------------------AGATGGTGGTAATAACAACAATGGGTCCGAGATCTTCAGACCTG
GAGGAGGAGATATGAGGGACAATTGGAGAAGTGAATTATATAAATATAAAGTAGTAAAAATTGAACCATTAGGAGTAGCA
CCCACCAAGGCAAAGAGAAGAGTGGTGCAGAGAGAAAAAAGAGCAGTGGGAATAGGAGCTTTGTTCCTTGGGTTCTTGGG
AGCAGCAGGAAGCACTATGGGCGCAGCGTCAATGACGCTGACGGTACAGGCCAGACAATTATTGTCTGGTATAGTGCAGC
AGCAGAACAATTTGCTGAGGGCTATTGAGGCGCAACAGCATCTGTTGCAACTCACAGTCTGGGGCATCAAGCAGCTCCAG
GCAAGAATCCTGGCTGTGGAAAGATACCTAAAGGATCAACAGCTCCTGGGGATTTGGGGTTGCTCTGGAAAACTCATTTG
CACCACTGCTGTGCCTTGGAATGCTAGTTGGAGTAATAAATCTCTGGAACAGATTTGGAATCACACGACCTGGATGGAGT
GGGACAGAGAAATTAACAATTACACAAGCTTAATACACTCCTTAATTGAAGAATCGCAAAACCAGCAAGAAAAGAATGAA
CAAGAATTATTGGAATTAGATAAATGGGCAAGTTTGTGGAATTGGTTTAACATAACAAATTGGCTGTGGTATATAAAATT
ATTCATAATGATAGTAGGAGGCTTGGTAGGTTTAAGAATAGTTTTTGCTGTACTTTCTGTAGTGAATAGAGTTAGGCAGG
GATATTCACCATTATCGTTTCAGACCCACCTCCCAATCCCGAGGGGACCCGACAGGCCCGAAGGAATAGAAGAAGAAGGT
GGAGAGAGAGACAGAGACAGATCCATTCGATTAGTGAACGGATCCTTAGCACTTATCTGGGACGATCTGCGGAGCCTGTG
CCTCTTCAGCTACCACCGCTTGAGAGACTTACTCTTGATTGTAACGAGGATTGTGGAACTTCTGGGACGCAGGGGGTGGG
AAGCCCTCAAATATTGGTGGAATCTCCTACANTATTGGAGTCAGGAGCTAAAGAATAGTGCTGTTAGCTTGCTCAATGCC
ACAGCTATAGCAGTAGCTGAGGGGACAGATAGGGTTATAGAAGTAGTACAAGAAGCTTATAGAGCTATTCGCCACATACC
TAGAAGAATAAGACAGGGCTTGGAAAGGATTTTGCTATAAGATGGGTGGCAAGTGGTCAAAAAGTAGTGTGGTTGGATGG
CCTGCTGTAAGGGAAAGAATGAGACGAGCTGAGCCAGCAGCAGATGGGGTGGGAGCAGCATCTCGAGACCTAGAAAAACA
TGGAGCAATCACAAGTAGCAACACAGCAGCTAACAATGCTGCTTGTGCCTGGCTAGAAGCACAAGAGGAGGAGAAGGTGG
GTTTTCCAGTCACACCTCAGGTACCTTTAAGACCAATGACTTACAAGGCAGCTGTAGATCTTAGCCACTTTTTAAAAGAA
AAGGGGGGACTGGAAGGGCTAATTCACTCCCAACGAAGACAAGATATCCTTGATCTGTGGATCTACCACACACAAGGCTA
CTTCCCTGATTGGCAGAACTACACACCAGGACCAGGGATCAGATATCCACTGACCTTTGGATGGCGCTACAAGCTAGTAC
CAGTTGAGCCAGAGAAGTTAGAAGAAGCCAACAAAGGAGAGAACACCAGCTTGTTACACCCTGTGAGCCTGCATGGAATG
GATGACCCGGAGAGAGAAGTGTTAGAGTGGAGGTTTGACAGCCGCCTAGCATTTCATCACGTGGCCCGAGAGCTGCATCC
GGAGTACTTCAAGAACTGCTGATATCGAGCTTGCTACAAGGGACTTTCCGCTGGGGACTTTCCAGGGAGGCGTGGCCTGG
GCGGGACTGGGGAGTGGCGAGCCCTCAGATCCTGCATATAAGCAGCTGCTTTTTGCCTGTACTGGGTCTCTCTGGTTAGA
CCAGATCTGAGCCTGGGAGCTCTCTGGCTAACTAGGGAACCCACTGCTTAAGCCTCAATAAAGCTTGCCTTGAGTGCTTC
               AAGTAGTGTGTGCCCATCTGTCGTGTGACTCTGGTAACTAGAGATCCCTCA
                                   >2-17envLTR
--------------------------------------------------------------------------------
--------------------------------------------------------------------------------
--------------------------------------------------------------------------------
--------------------------------------------------------------------------------
--------------------------------------------------------------------------------
--------------------------------------------------------------------------------
```

```
--------------------------------------------------------------------------------
--------------------------------------------------------------------------------
--------------------------------------------------------------------------------
--------------------------------------------------------------------------------
--------------------------------------------------------------------------------
--------------------------------------------------------------------------------
--------------------------------------------------------------------------------
--------------------------------------------------------------------------------
--------------------------------------------------------------------------------
--------------------------------------------------------------------------------
--------------------------------------------------------------------------------
--------------------------------------------------------------------------------
--------------------------------------------------------------------------------
--------------------------------------------------------------------------------
--------------------------------------------------------------------------------
--------------------------------------------------------------------------------
--------------------------------------------------------------------------------
--------------------------------------------------------------------------------
--------------------------------------------------------------------------------
--------------------------------------------------------------------------------
--------------------------------------------------------------------------------
--------------------------------------------------------------------------------
--------------------------------------------------------------------------------
--------------------------------------------------------------------------------
--------------------------------------------------------------------------------
--------------------------------------------------------------------------------
--------------------------------------------------------------------------------
--------------------------------------------------------------------------------
--------------------------------------------------------------------------------
--------------------------------------------------------------------------------
--------------------------------------------------------------------------------
--------------------------------------------------------------------------------
--------------------------------------------------------------------------------
--------------------------------------------------------------------------------
--------------------------------------------------------------------------------
--------------------------------------------------------------------------------
--------------------------------------------------------------------------------
--------------------------------------------------------------------------------
--------------------------------------------------------------------------------
--------------------------------------------------------------------------------
--------------------------------------------------------------------------------
--------------------------------------------------------------------------------
--------------------------------------------------------------------------------
--------------------------------------------------------------------------------
--------------------------------------------------------------------------------
--------------------------------------------------------------------------------
--------------------------------------------------------------------------------
--------------------------------------------------------------------------------
--------------------------------------------------------------------------------
--------------------------------------------------------------------------------
--------------------------------------------------------------------------------
--------------------------------------------------------------------------------
--------------------------------------------------------------------------------
--------------------------------------------------------------------------------
--------------------------------------------------------------------------------
--------------------------------------------------------------------------------
--------------------------------------------------------------------------------
--------------------------------------------------------------------------------
--------------------------------------------------------------------------------
--------------------------------------------------------------------------------
--------------------------------------------------------------------------------
--------------------------------------------------------------------------------
--------------------------------------------------------------------------------
--------------------------------------------------------------------------------
--------------------------------------------------------------------------------
--------------------------------------------------------------------------------
--------------------------------------------------------------------------------
--------------------------------------------------------------------------------
--------------------------------------------------------------------------------
--------------------------------------------------------------------------------
--------------------------------------------------------------------------------
--------------------------------------------------------------------------------
--------------------------------------------------------------------------------
--------------------------------------------------------------------------------
--------------------------------------------------------------------------------
--------------------------------------------------------------------------------
--------------------------------------------------------------------------------
--------------------------------------------------------------------------------
--------------------------------------------------------------------------------
--------------------------------------------------------------------------------
--------------------------------------------------------------------------------
--------------------------------------------------------------------------------
--------------------------------------------------------------------------------
--------------------------------------------------------------------------------
--------------------------------------------------------------------------------
--------------------------------------------------------------------------------
--------------------------------------------------------------------------------
--------------------------------------------------------------------------------
--------------TTACAGGGCTGCTATTAACAAGAGATGGTGGTAATAACAACAATGGGTCCGAGATCTTCAGACCTG
GAGGAGGAGATATGAGGGACAATTGGAGAAGTGAATTATATAAATATAAAGTAGTAAAAATTGAACCATTAGGAGTAGCA
CCCACCAAGGCAAAGAGAAGAGTGGTGCAGAGAGAAAAAAGAGCAGTGGGAATAGGAGCTTTGTTCCTTGGGTTCTTGGG
AGCAGCAGGAAGCACTATGGGCGCAGCGTCAATGACGCTGACGGTACAGGCCAGACAATTATTGTCTGGTATAGTGCAGC
AGCAGAACAATTTGCTGAGGGCTATTGAGGCGCAACAGCATCTGTTGCAACTCACAGTCTGGGGCATCAAGCAGCTCCAG
GCAAGAATCCTGGCTGTGGAAAGATACCTAAAGGATCAACAGCTCCTGGGGATTTGGGGTTGCTCTGGAAAACTCATTTG
```

```
CACCACTGCTGTGCCTTGGAATGCTAGTTGGAGTAATAAATCTCTGGAACAGATTTGGAATCACACGACCTGGATGGAGT
GGGACAGAGAAATTAACAATTACACAAGCTTAATACACTCCTTAATTGAAGAATCGCAAAACCAGCAAGAAAAGAATGAA
CAAGAATTATTGGAATTAGATAAATGGGCAAGTTTGTGGAATTGGTTTAACATAACAAATTGGCTGTGGTATATAAAATT
ATTCATAATGATAGTAGGAGGCTTGGTAGGTTTAAGAATAGTTTTTGCTGTACTTTCTGTAGTGAATAGAGTTAGGCAGG
GATATTCACCATTATCGTTTCAGACCCACCTCCCAATCCCGAGGGGACCCGACAGGCCCGAAGGAATAGAAGAAGAAGGT
GGAGAGAGAGACAGAGACAGATCCATTCGATTAGTGAACGGATCCTTAGCACTTATCTGGGACGATCTGCGGAGCCTGTG
CCTCTTCAGCTACCACCGCTTGAGAGACTTACTCTTGATTGTAACGAGGATTGTGGAACTTCTGGGACGCAGGGGGTGGG
AAGCCCTCAAATATTGGTGGAATCTCCTACAATATTGGAGTCAGGAGCTAAAGAATAGTGCTGTTAGCTTGCTCAATGCC
ACAGCTATAGCAGTAGCTGAGGGGACAGATAGGGTTATAGAAGTAGTACAAGAAGCTTATAGAGCTATTCGCCACATACC
TAGAAGAATAAGACAGGGCTTGGAAAGGATTTTGCTATAAGATGGGTGGCAAGTGGTCAAAAAGTAGTGTGGTTGGATGG
CCTGCTGTAAGGGAAAGAATGAGACGAGCTGAGCCAGCAGCAGATGGGGTGGGAGCAGCATCTCGAGACCTAGAAAAACA
TGGAGCAATCACAAGTAGCAACACAGCAGCTAACAATGCTGCTTGTGCCTGGCTAGAAGCACAAGAGGAGGAGAAGGTGG
GTTTTCCAGTCACACCTCAGGTACCTTTAAGACCAATGACTTACAAGGCAGCTGTAGATCTTAGCCACTTTTTAAAAGAA
AAGGGGGGACTGGAAGGGCTAATTCACTCCCAACGAAGACAAGATATCCTTGATCTGTGGATCTACCACACACAAGGCTA
CTTCCCTGATTGGCAGAACTACACACCAGGACCAGGGATCAGATATCCACTGACCTTTGGATGGCGCTACAAGCTAGTAC
CAGTTGAGCCAGAGAAGTTAGAAGAAGCCAACAAAGGAGAGAACACCAGCTTGTTACACCCTGTGAGCCTGCATGGAATG
GATGACCCGGAGAGAGAAGTGTTAGAGTGGAGGTTTGACAGCCGCCTAGCATTTCATCACGTGGCCCGAGAGCTGCATCC
GGAGTACTTCAAGAACTGCTGATATCGAGCTTGCTACAAGGGACTTTCCGCTGGGGACTTTCCAGGGAGGCGTGGCCTGG
GCGGGACTGGGGAGTGGCGAGCCCTCAGATCCTGCATATAAGCAGCTGCTTTTTGCCTGTACTGGGTCTCTCTGGTTAGA
CCAGATCTGAGCCTGGGAGCTCTCTGGCTAACTAGGGAACCCACTGCTTAAGCCTCAATAAAGCTTGCCTTGAGTGCTTC
AAGTAGTGTGTGCCCATCTGTTGTGTGACTCTGGTAACTAGAGATCCCTCA
>2-23envLTR
--------------------------------------------------------------------------------
--------------------------------------------------------------------------------
--------------------------------------------------------------------------------
--------------------------------------------------------------------------------
--------------------------------------------------------------------------------
--------------------------------------------------------------------------------
--------------------------------------------------------------------------------
--------------------------------------------------------------------------------
--------------------------------------------------------------------------------
--------------------------------------------------------------------------------
--------------------------------------------------------------------------------
--------------------------------------------------------------------------------
--------------------------------------------------------------------------------
--------------------------------------------------------------------------------
--------------------------------------------------------------------------------
--------------------------------------------------------------------------------
--------------------------------------------------------------------------------
--------------------------------------------------------------------------------
--------------------------------------------------------------------------------
--------------------------------------------------------------------------------
--------------------------------------------------------------------------------
--------------------------------------------------------------------------------
--------------------------------------------------------------------------------
--------------------------------------------------------------------------------
--------------------------------------------------------------------------------
--------------------------------------------------------------------------------
--------------------------------------------------------------------------------
--------------------------------------------------------------------------------
--------------------------------------------------------------------------------
--------------------------------------------------------------------------------
--------------------------------------------------------------------------------
--------------------------------------------------------------------------------
--------------------------------------------------------------------------------
--------------------------------------------------------------------------------
--------------------------------------------------------------------------------
--------------------------------------------------------------------------------
--------------------------------------------------------------------------------
--------------------------------------------------------------------------------
--------------------------------------------------------------------------------
--------------------------------------------------------------------------------
--------------------------------------------------------------------------------
--------------------------------------------------------------------------------
--------------------------------------------------------------------------------
--------------------------------------------------------------------------------
--------------------------------------------------------------------------------
--------------------------------------------------------------------------------
--------------------------------------------------------------------------------
--------------------------------------------------------------------------------
--------------------------------------------------------------------------------
--------------------------------------------------------------------------------
--------------------------------------------------------------------------------
--------------------------------------------------------------------------------
--------------------------------------------------------------------------------
--------------------------------------------------------------------------------
--------------------------------------------------------------------------------
--------------------------------------------------------------------------------
--------------------------------------------------------------------------------
--------------------------------------------------------------------------------
--------------------------------------------------------------------------------
--------------------------------------------------------------------------------
--------------------------------------------------------------------------------
--------------------------------------------------------------------------------
--------------------------------------------------------------------------------
--------------------------------------------------------------------------------
--------------------------------------------------------------------------------
--------------------------------------------------------------------------------
--------------------------------------------------------------------------------
--------------------------------------------------------------------------------
--------------------------------------------------------------------------------
--------------------------------------------------------------------------------
--------------------------------------------------------------------------------
--------------------------------------------------------------------------------
--------------------------------------------------------------------------------
```


--GGTGGTAATAACAACAATGGGTCCGAGATCTTCAGACCTG
GAGGAGGAGATATGAGGGACAATTGGAGAAGTGAATTATATAAATATAAAGTAGTAAAAATTGAACCATTAGGAGTAGCA
CCCACCAAGGCAAAGAGAAGAGTGGTGCAGAGAGAAAAAAGAGCAGTGGGAATAGGAGCTTTGTTCCTTGGGTTCTTGGG
AGCAGCAGGAAGCACTATGGGCGCAGCGTCAATGACGCTGACGGTACAGGCCAGACAATTATTGTCTGGTATAGTGCAGC
AGCAGAACAATTTGCTGAGGGCTATTGAGGCGCAACAGCATCTGTTGCAACTCACAGTCTGGGGCATCAAGCAGCTCCAG
GCAAGAATCCTGGCTGTGGAAAGATACCTAAAGGATCAACAGCTCCTGGGGATTTGGGGTTGCTCTGGAAAACTCATTTG
CACCACTGCTGTGCCTTGGAATGCTAGTTGGAGTAATAAATCTCTGGAACAGATTTGGAATCACACGACCTGGATGGAGT
GGGACAGAGAAATTAACAATTACACAAGCTTAATACACTCCTTAATTGAAGAATCGCAAAACCAGCAAGAAAAGAATGAA
CAAGAATTATTGGAATTAGATAAATGGGCAAGTTTGTGGAATTGGTTTAACATAACAAATTGGCTGTGGTATATAAAATT
ATTCATAATGATAGTAGGAGGCTTGGTAGGTTTAAGAATAGTTTTTGCTGTACTTTCTGTAGTGAATAGAGTTAGGCAGG
GATATTCACCATTATCGTTTCAGACCCACCTCCCAATCCCGAGGGGACCCGACAGGCCCGAAGGAATAGAAGAAGAAGGT
GGAGAGAGAGACAGAGACAGATCCATTCGATTAGTGAACGGATCCTTAGCACTTATCTGGGACGATCTGCGGAGCCTGTG
CCTCTTCAGCTACCACCGCTTGAGAGACTTACTCTTGATTGTAACGAGGATTGTGGAACTTCTGGGACGCAGGGGGTGGG
AAGCCCTCAAATATTGGTGGAATCTCCTACAATATTGGAGTCAGGAGCTAAAGAATAGTGCTGTTAGCTTGCTCAATGCC
ACAGCTATAGCAGTAGCTGAGGGGACAGATAGGGTTATAGAAGTAGTACAAGAAGCTTATAGAGCTATTCGCCACATACC
TAGAAGAATAAGACAGGGCTTGGAAAGGATTTTGCTATAAGATGGGTGGCAAGTGGTCAAAAAGTAGTGTGGTTGGATGG
CCTGCTGTAAGGGAAAGAATGAGACGAGCTGAGCCAGCAGCAGATGGGGTGGGAGCAGCATCTCGAGACCTAGAAAAACA
TGGAGCAATCACAAGTAGCAACACAGCAGCTAACAATGCTGCTTGTGCCTGGCTAGAAGCACAAGAGGAGGAGAAGGTGG
GTTTTCCAGTCACACCTCAGGTACCTTTAAGACCAATGACTTACAAGGCAGCTGTAGATCTTAGCCACTTTTTAAAAGAA
AAGGGGGGACTGGAAGGGCTAATTCACTCCCAACGAAGACAAGATATCCTTGATCTGTGGATCTACCACACACAAGGCTA
CTTCCCTGATTGGCAGAACTACACACCAGGACCAGGGATCAGATATCCACTGACCTTTGGATGGCGCTACAAGCTAGTAC
CAGTTGAGCCAGAGAAGTTAGAAGAAGCCAACAAAGGAGAGAACACCAGCTTGTTACACCCTGTGAGCCTGCATGGAATG
GATGACCCGGAGAGAGAAGTGTTAGAGTGGAGGTTTGACAGCCGCCTAGCATTTCATCACGTGGCCCGAGAGCTGCATCC
GGAGTACTTCAAGAACTGCTGATATCGAGCTTGCTACAAGGGACTTTCCGCTGGGGACTTTCCAGGGAGGCGTGGCCTGG
GCGGGACTGGGGAGTGGCGAGCCCTCAGATCCTGCATATAAGCAGCTGCTTTTTGCCTGTACTGGGTCTCTCTGGTTAGA
CCAGATCTGAGCCTGGGAGCTCTCTGGCTAACTAGGGAACCCACTGCTTAAGCCTCAATAAAGCTTGCCTTGAGTGCTTC
AAGTAGTGTGTGCCCATCTGTTGTGTGACTCTGGTAACTAGAGATCCCTCA

>2-29envLTR

--AAAGCAATGTATGCCCCTCCCATCAGCGGACAAATTAG
ATGTTCATCAAATATTACAGGGCTGCTATTAACAAGAGATGGTGGTAATAACAACAATGGGTCCGAGATCTTCAGACCTG
GAGGAGGAGATATGAAGGACAATTGGAGAAGTGAATTATATAAATATAAAGTAGTAAAAATTGAACCATTAGGAGTAGCA
CCCACCAAGGCAAAGAGAAGAGTAGTGCAGAGAGAAAAAAGAGCAGTAGGAATAGGAGCTTTGTTCCTTAGGTTCTTAGG
AGCAGCAGGAAGCACTATAGGCGCAGCGTCAATGACGCTGACGGTACAGGCCAGACAATTATTGTCTGGTATAGTGCAGC
AGCAGAACAATTTGCTGAGGGCTATTGAGGCGCAACAGCATCTGTTGCAACTCACAGTCTGAGGCATCAAGCAGCTCCAG
GCAAGAATCCTGGCTGTGGAAAGATACCTAAAGGATCAACAGCTCCTGGGGATTTGAGGTTGCTCTGGAAAACTCATTTG
CACCACTGCTGTGCCTTGGAATGCTAGTTGGAGTAATAAATCTCTGGAACAGATTTGGAATCACACGACCTGGATGGAGT
AGGACAGAGAAATTAACAATTACACAAGCTTAATACACTCCTTAATTGAAGAATCGCAAAACCAGCAAGAAAAGAATGAA
CAAGAATTATTAGAATTAGATAAATGGGCAAGTTTGTGGAATTGGTTTAACATAACAAATTGGCTGTGGTATATAAAATT
ATTCATAATGATAGTAGGAGGCTTGGTAGGTTTAAGAATAGTTTTTGCTGTACTTTCTGTAGTGAATAGAGTTAGGCAAG
GATATTCACCATTATCGTTTCAGACCCACCTCCCAATCCCGAGGGGACCCGACAGGCCCGAAGGAATAGAAGAAGAAGGT
GGAGAGAGAGACAGAGACAGATCCATTCGATTAGTGAACGGATCCTTAGCACTTATCTAGGACGATCTGCGGAGCCTGTG
CCTCTTCAGCTACCACCGCTTGAGAGACTTACTCCTGATTGTAACGAGGATTGTGGAACTTCTAAGACGCAGGGGGTGAG
AAGCCCTCAAATATTGGTAAAATCTCCTACAATATTAGAGTCAGGAGCTAAAGAATAGTGCTGTTAGCTTGCTCAATGCC
ACAGCTATAGCAGTAGCTGAGAGGACAGATAAGGTTATAGAAGTAGTACAAGAAGCTTATAGAGCTATTCGCCACATACC
TAGAAGAATAAGACAGGGCTTAGAAAGGATTTTGCTATAAGATAAGTGGCAAGTGGTCAAAAAGTAGTGTGGTTAGATGG
CCTGCTGTAAGGGAAAGAATGAGACGAGCTGAGCCAGCAGCAGATGGGGTGGGAGCAGCATCTCAAGACCTAGAAAAACA
TAGAGCAATCACAAGTAGCAACACAGCAGCTAACAATGCTGCTTGTGCCTGGCTAGAAGCACAAGAGGAGGAGAAGGTAG
GTTTTCCAGTCACACCTCAGGTACCTTTAAGACCAATGACTTACAAGGCAGCTGTAGATCTTAGCCACTTTTTAAAAGAA
AAGGGGGGACTGGAAGGGCTAATTCACTCCCAACGAAGACAAGATATCCTTGATCTGTGGATCTACCACACACAAGGCTA
CTTCCCTGATTGGCAGAACTACACACCAGGACCAGGGATCAGATATCCACTGACCTTTGGATGGCGCTACAAGCTAGTAC
CAGTTGAGCCAGAGAAGTTAGAAGAAGCCAACAAAGGAGAGAACACCAGCTTGTTACACCCTGTGAGCCTGCATGGAATG
GATGACCCGGAGAGAGAAGTGTTAGAGTGGAGGTTTGACAGCCGCCTAGCATTTCATCACGTGGCCCGAGAGCTGCATCC
GGAGTACTTCAAGAACTGCTGATATCGAGCTTGCTACAAGGGACTTTCCGCTGAGGACTTTCCAGGGAGGCGTGGCCTGG
GCAGGACTGGGGAGTGGCGAGCCCTCAGATCCTGCATATAAGCAGCTGCTTTTTGCCTGTACTAGGTCTCTCTGGTTAGA
CCAGATCTGAGCCTAGGAGCTCTCTGGCTAACTAAGGAACCCACTGCTTAAGCCTCAATAAAGCTTGCCTTGAGTGCTTC
AAGTAGTGTGTGCCCATCTGTTGTGTGACTCTAGTAACTAGAGATCCCTCA
>2-32envLTR


```
--------------------------------------------------------------------------------
--------------------------------------------------------------------------------
--------------------------------------------------------------------------------
--------------------------------------------------------------------------------
--------------------------------------------------------------------------------
--------------------------------------------------------------------------------
--------------------------------------------------------------------------------
--------------------------------------------------------------------------------
--------------------------------------------------------------------------------
--------------------------------------------------------------------------------
--------------------------------------------------------------------------------
--------------------------------------------------------------------------------
--------------------------------------------------------------------------------
--------------------------------------------------------------------------------
--------------------------------------------------------------------------------
--------------------------------------------------------------------------------
--------------------------------------------------------------------------------
--------------------------------------------------------------------------------
--------------------------------------------------------------------------------
--------------------------------------------------------------------------------
--------------------------------------------------------------------------------
--------------------------------------------------------------------------------
--------------------------------------------------------------------------------
--------------------------------------------------------------------------------
--------------------------------------------------------------------------------
--------------------------------------------------------------------------------
--------------------------------------------------------------------------------
--------------------------------------------------------------------------------
--------------------------------------------------------------------------------
--------------------------------------------------------------------------------
--------------------------------------------------------------------------------
--------------------------------------------------------------------------------
--------------------------------------------------------------------------------
--------------------------------------------------------------------------------
--------------------------------------------------------------------------------
--------------------------------------------------------------------------------
--------------------------------------------------------------------------------
--------------------------------------------------------------------------------
--------------------------------------------------------------------------------
--------------------------------------------------------------------------------
--------------------------------------------------------------------------------
--------------------------------------------------------------------------------
--------------------------------------------------------------------------------
--------------------------------------------------------------------------------
--------------------------------------------------------------------------------
--------------------------------------------------------------------------------
--------------------------------------------------------------------------------
--------------------------------------------------------------------------------
--------------------------------------------------------------------------------
--------------------------------------------------------------------------------
--------------------------------------------------------------------------------
--------------------------------------------------------------------------------
--------------------------------------------------------------------------------
--------------------------------------------------------------------------------
--------------------------------------------------------------------------------
--------------------------------------------------------------------------------
--------------------------------------------------------------------------------
--------------------------------------------------------------------------------
--------------------------------------------------------------------------------
--------------------------------------------------------------------------------
--------------------------------------------------------------------------------
--------------------------------------------------------------------------------
--------------------------------------------------------------------------------
--------------------------------------------------------------------------------
--------------------------------------------------------------------------------
--------------------------------------------------------------------------------
--------------------------------------------------------------------------------
--------------------------------------------------------------------------------
--------------------------------------------------------------------------------
--------------------------------------------------------------------------------
--------------------------------------------------------------------------------
--------------------------------------------------------------------------------
--------------------------------------------------------------------------------
--------------------------------------------------------------------------------
--------------------------------------------------------------------------------
--------------------------------------------------------------------------------
--------------------------------------------------------------------------------
------------------------------------------AAAGCAATGTATGCCCCTCCCATCAGCGGACAAATTAG
ATGTTCATCAAATATTACAGGGCTGCTATTAACAAGAGATGGTGGTAATAACAACAATGGGTCCGAGATCTTCAGACCTG
GAGGAGGAGATATGAGGGACAATTGGAGAAGTGAATTATATAAATATAAAGTAGTAAAAATTGAACCATTAGGAGTAGCA
CCCACCAAGGCAAAGAGAAGAGTGGTGCAGAGAGAAAAAAGAGCAGTGGGAATAGGAGCTTTGTTCCTTGGGTTCTTGGG
AGCAGCAGGAAGCACTATGGGCGCAGCGTCAATGACGCTGACGGTACAGGCCAGACAATTATTGTCTGGTATAGTGCAGC
AGCAGAACAATTTGCTGAGGGCTATTGAGGCGCAACAGCATCTGTTGCAACTCACAGTCTGGGGCATCAAGCAGCTCCAG
GCAAGAATCCTGGCTGTGGAAAGATACCTAAAGGATCAACAGCTCCTGGGGATTTGGGGTTGCTCTGGAAAACTCATTTG
CACCACTGCTGTGCCTTGGAATGCTAGTTGGAGTAATAAATCTCTGGAACAGATTTGGAATCACACGACCTGGATGGAGT
GGGACAGAGAAATTAACAATTACACAAGCTTAATACACTCCTTAATTGAAGAATCGCAAAACCAGCAAGAAAAGAATGAA
CAAGAATTATTGGAATTAGATAAATGGGCAAGTTTGTGGAATTGGTTTAACATAACAAATTGGCTGTGGTATATAAAATT
ATTCATAATGATAGTAGGAGGCTTGGTAGGTTTAAGAATAGTTTTTGCTGTACTTTCTGTAGTGAATAGAGTTAGGCAGG
GATATTCACCATTATCGTTTCAGACCCACCTCCCAATCCCGAGGGGACCCGACAGGCCCGAAGGAATAGAAGAAGAAGGT
GGAGAGAGAGACAGAGACAGATCCATTCGATTAGTGAACGGATCCTTAGCACTTATCTGGGACGATCTGCGGAGCCTGTG
CCTCTTCAGCTACCACCGCTTGAGAGACTTACTCTTGATTGTAACGAGGATTGTGGNACTTCTGGGACGCAGGGGGTGGG
AAGCCCTCAAATATTGGTGGAATCTCCTACAATATTGGAGTCAGGAGCTAAAGAATAGTGCTGTTAGCTTGCTCAATGCC
ACAGCTATAGCAGTAGCTGAGGGGACAGATAGGGTTATAGAAGTAGTACAAGAAGCTTATAGAGCTATTCGCCACATACC
TAGAAGAATAAGACAGGGCTTGGAAAGGATTTTGCTATAAGATGGGTGGCAAGTGGTCAAAAAGTAGTGTGGTTGGATGG
```

```
CCTGCTGTAAGGGAAAGAATGAGACGAGCTGAGCCAGCAGCAGATGGGGTGGGAGCAGCATCTCGAGACCTAGAAAAACA
TGGAGCAATCACAAGTAGCAACACAGCAGCTAACAATGCTGCTTGTGCCTGGCTAGAAGCACAAGAGGAGGAGAAGGTGG
GTTTTCCAGTCACACCTCAGGTACCTTTAAGACCAATGACTTACAAGGCAGCTGTAGATCTTAGCCACTTTTTAAAAGAA
AAGGGGGGACTGGAAGGGCTAATTCACTCCCAACGAAGACAAGATATCCTTGATCTGTGGATCTACCACACACAAGGCTA
CTTCCCTGATTGGCAGAACTACACACCAGGACCAGGGATCAGATATCCACTGACCTTTGGATGGCGCTACAAGCTAGTAC
CAGTTGAGCCAGAGAAGTTAGAAGAAGCCAACAAAGGAGAGAACACCAGCTTGTTACACCCTGTGAGCCTGCATGGAATG
GATGACCCGGAGAGAGAAGTGTTAGAGTGGAGGTTTGACAGCCGCCTAGCATTTCATCACGTGGCCCGAGAGCTGCATCC
GGAGTACTTCAAGAACTGCTGATATCGAGCTTGCTACAAGGGACTTTCCGCTGGGGACTTTCCAGGGAGGCGTGGCCTGG
GCGGGACTGGGGAGTGGCGAGCCCTCAGATCCTGCATATAAGCAGCTGCTTTTTGCCTGTACTGGGTCTCTCTGGTTAGA
CCAGATCTGAGCCTGGGAGCTCTCTGGCTAACTAGGGAACCCACTGCTTAAGCCTCAATAAAGCTTGCCTTGAGTGCTTC
AAGTAGTGTGTGCCCATCTGTTGTGTGACTCTGGTAACTAGAGATCCCTCA
>2-40envLTR
--------------------------------------------------------------------------------
--------------------------------------------------------------------------------
--------------------------------------------------------------------------------
--------------------------------------------------------------------------------
--------------------------------------------------------------------------------
--------------------------------------------------------------------------------
--------------------------------------------------------------------------------
--------------------------------------------------------------------------------
--------------------------------------------------------------------------------
--------------------------------------------------------------------------------
--------------------------------------------------------------------------------
--------------------------------------------------------------------------------
--------------------------------------------------------------------------------
--------------------------------------------------------------------------------
--------------------------------------------------------------------------------
--------------------------------------------------------------------------------
--------------------------------------------------------------------------------
--------------------------------------------------------------------------------
--------------------------------------------------------------------------------
--------------------------------------------------------------------------------
--------------------------------------------------------------------------------
--------------------------------------------------------------------------------
--------------------------------------------------------------------------------
--------------------------------------------------------------------------------
--------------------------------------------------------------------------------
--------------------------------------------------------------------------------
--------------------------------------------------------------------------------
--------------------------------------------------------------------------------
--------------------------------------------------------------------------------
--------------------------------------------------------------------------------
--------------------------------------------------------------------------------
--------------------------------------------------------------------------------
--------------------------------------------------------------------------------
--------------------------------------------------------------------------------
--------------------------------------------------------------------------------
--------------------------------------------------------------------------------
--------------------------------------------------------------------------------
--------------------------------------------------------------------------------
--------------------------------------------------------------------------------
--------------------------------------------------------------------------------
--------------------------------------------------------------------------------
--------------------------------------------------------------------------------
--------------------------------------------------------------------------------
--------------------------------------------------------------------------------
--------------------------------------------------------------------------------
--------------------------------------------------------------------------------
--------------------------------------------------------------------------------
--------------------------------------------------------------------------------
--------------------------------------------------------------------------------
--------------------------------------------------------------------------------
--------------------------------------------------------------------------------
--------------------------------------------------------------------------------
--------------------------------------------------------------------------------
--------------------------------------------------------------------------------
--------------------------------------------------------------------------------
--------------------------------------------------------------------------------
--------------------------------------------------------------------------------
--------------------------------------------------------------------------------
--------------------------------------------------------------------------------
--------------------------------------------------------------------------------
--------------------------------------------------------------------------------
--------------------------------------------------------------------------------
--------------------------------------------------------------------------------
--------------------------------------------------------------------------------
--------------------------------------------------------------------------------
--------------------------------------------------------------------------------
--------------------------------------------------------------------------------
--------------------------------------------------------------------------------
--------------------------------------------------------------------------------
--------------------------------------------------------------------------------
--------------------------------------------------------------------------------
--------------------------------------------------------------------------------
--------------------------------------------------------------------------------
--------------------------------------------------------------------------------
--------------------------------------------------------------------------------
--------------------------------------------------------------------------------
--------------------------------------------------------------------------------
--------------------------------------------------------------------------------
--------------------------------------------------------------------------------
--------------------------------------------------------------------------------
--------------------------------------------------------------------------------
--------------------------------------------------------------------------------
--------------------------------------------------------------------------------
```

```
--------------------------------------------------------------------------------
--------------------------------------------------------------------------------
--------------------------------------------------------------------------------
--------------------------------------------------------------------------------
--------------------------------------------------------------------------------
--------------------------------------------------------------------------------
--------------------------------------------------------------------------------
--------------------------------------------------------------------------------
--------------------------------------------------------------------------------
--------------------------------------------------------------------------------
--------------------------------------------------------------------------------
------------------------------------------AAAGCAATGTATGCCCCTCCCATCAGCGGACAAATTAG
ATGTTCATCAAATATTACAGGGCTGCTATTAACAAGAGATGGTGGTAATAACAACAATGGGTCCGAGATCTTCAGACCTG
GAGGAGGAGATATGAGGGACAATTGGAGAAGTGAATTATATAAATATAAAGTANTAAAAATTGAACCATTAGGAGTAGCA
CCCACCAAGGCAAAGAGAAGAGTGGTGCAGAGAGAAAAAAGAGCAGTGGGAATAGGAGCTTTGTTCCTTGGGTTCTTGGG
AGCAGCAGGAAGCACTATGGGCGCAGCGTCAATGACGCTGACGGTACAGGCCAGACAATTATTGTCTGGTATAGTGCAGC
AGCAGAACAATTTGCTGAGGGCTATTGAGGCGCAACAGCATCTGTTGCAACTCACAGTCTGGGGCATCAAGCAGCTCCAG
GCAAGAATCCTGGCTGTGGAAAGATACCTAAAGGATCAACAGCTCCTGGGGATTTGGGGTTGCTCTGGAAAACTCATTTG
CACCACTGCTGTGCCTTGGAATGCTAGTTGGAGTAATAAATCTCTGGAACAGATTTGGAATCACACGACCTGGATGGAGT
GGGACAGAGAAATTANCAATTACACAAGCTTAATACACTCCTTAATTGAAGAATCGCAAAACCAGCAAGAAAAGAATGAA
CAAGAATTATTGGAATTAGATAAATGGGCAAGTTTGTGGAATTGGTTTAACATAACAAATTGGCTGTGGTATATAAAATT
ATTCATAATGATAGTAGGAGGCTTGGTAGGTTTAAGAATAGTTTTTGCTGTACTTTCTGTAGTGAATAGAGTTAGGCAGG
GATATTCACCATTATCGTTTCAGACCCACCTCCCAATCCCGAGGGGACCCGACAGGNCCGAAGGAATAGAAGAAGAAGGT
GGAGAGAGAGACAGAGACAGATCCATTCGATTAGTGAACGGATCCTTAGCACTTATCTGGGACGATCTGCGGAGCCTGTG
CCTCTTCAGCTACCACCGCTTGAGAGACTTACTCTTGATTGTAACGAGGATTGTGGAACTTCTGGGACGCAGGGGGTGGG
AAGCCCTCAAATATTGGTGGAATCTCCTACAATATTGGAGTCAGGAGCTAAAGAATAGTGCTGTTAGCTTGCTCAATGCC
ACAGCTATAGCAGTAGCTGAGGGGACAGATAGGGTTATAGAAGTAGTACAAGAAGCTTATAGAGCTATTCGCCACATACC
TAGAAGAATAAGACAGGGCTTGGAAAGGATTTTGCTATAAGATGGGTGGCAAGTGGTCAAAAAGTAGTGTGGTTGGATGG
CCTGCTGTAAGGGAAAGAATGAGACGAGCTGAGCCAGCAGCAGATGGGGTGGGAGCAGCATCTCGAGACCTAGAAAAACA
TGGAGCAATCACAAGTAGCAACACAGCAGCTAACAATGCTGCTTGTGCCTGGCTAGAAGCACAAGAGGAGGAGAAGGTGG
GTTTTCCAGTCACACCTCAGGTACCTTTAAGACCAATGACTTACAAGGCAGCTGTANATCTTAGCCACTTTTTAAAAGAA
AAGGGGGGACTGGAAGGGCTAATTCACTCCCAACGAAGACAAGATATCCTTGATCTGTGGATCTACCACACACAAGGCTA
CTTCCCTGATTGGCAGAACTACACACCAGGACCAGGGATCAGATATCCACTGACCTTTGGATGGCGCTACAAGCTAGTAC
CAGTTGAGCCAGAGAAGTTAGAAGAAGCCAACAAAGGAGAGAACACCAGCTTGTTACACCCTGTGAGCCTGCATGGAATG
GATGACCCGGAGAGAGAAGTGTTAGAGTGGAGGTTTGACAGCCGCCTAGCATTTCATCACGTGGCCCGAGAGCTGCATCC
GGAGTACTTCAAGAACTGCTGATATCGAGCTTGCTACAAGGGACTTTCCGCTGGGGACTTTCCAGGGAGGCGTGGCCTGG
GCGGGACTGGGGAGTGGCGAGCCCTCAGATCCTGCATATAAGCAGCTGCTTTTTGCCTGTACTGGGTCTCTCTGGTTAGA
CCAGATCTGAGCCTGGGAGCTCTCTGGCTAACTAGGGAACCCACTGCTTAAGCCTCAATAAAGCTTGCCTTGAGTGCTTC
AAGTAGTGTGTGCCCATCTGTTGTGTGACTCTGGTAACTAGAGATCCCTCA
>2-41envLTR
--------------------------------------------------------------------------------
--------------------------------------------------------------------------------
--------------------------------------------------------------------------------
--------------------------------------------------------------------------------
--------------------------------------------------------------------------------
--------------------------------------------------------------------------------
--------------------------------------------------------------------------------
--------------------------------------------------------------------------------
--------------------------------------------------------------------------------
--------------------------------------------------------------------------------
--------------------------------------------------------------------------------
--------------------------------------------------------------------------------
--------------------------------------------------------------------------------
--------------------------------------------------------------------------------
--------------------------------------------------------------------------------
--------------------------------------------------------------------------------
--------------------------------------------------------------------------------
--------------------------------------------------------------------------------
--------------------------------------------------------------------------------
--------------------------------------------------------------------------------
--------------------------------------------------------------------------------
--------------------------------------------------------------------------------
--------------------------------------------------------------------------------
--------------------------------------------------------------------------------
--------------------------------------------------------------------------------
--------------------------------------------------------------------------------
--------------------------------------------------------------------------------
--------------------------------------------------------------------------------
--------------------------------------------------------------------------------
--------------------------------------------------------------------------------
--------------------------------------------------------------------------------
--------------------------------------------------------------------------------
--------------------------------------------------------------------------------
--------------------------------------------------------------------------------
--------------------------------------------------------------------------------
--------------------------------------------------------------------------------
--------------------------------------------------------------------------------
--------------------------------------------------------------------------------
--------------------------------------------------------------------------------
--------------------------------------------------------------------------------
--------------------------------------------------------------------------------
--------------------------------------------------------------------------------
--------------------------------------------------------------------------------
--------------------------------------------------------------------------------
--------------------------------------------------------------------------------
--------------------------------------------------------------------------------
--------------------------------------------------------------------------------
--------------------------------------------------------------------------------
--------------------------------------------------------------------------------
--------------------------------------------------------------------------------
--------------------------------------------------------------------------------
--------------------------------------------------------------------------------
--------------------------------------------------------------------------------
--------------------------------------------------------------------------------
```

```
--------------------------------------------------------------------------------
--------------------------------------------------------------------------------
--------------------------------------------------------------------------------
--------------------------------------------------------------------------------
--------------------------------------------------------------------------------
--------------------------------------------------------------------------------
--------------------------------------------------------------------------------
--------------------------------------------------------------------------------
--------------------------------------------------------------------------------
--------------------------------------------------------------------------------
--------------------------------------------------------------------------------
--------------------------------------------------------------------------------
--------------------------------------------------------------------------------
--------------------------------------------------------------------------------
--------------------------------------------------------------------------------
--------------------------------------------------------------------------------
--------------------------------------------------------------------------------
--------------------------------------------------------------------------------
--------------------------------------------------------------------------------
--------------------------------------------------------------------------------
--------------------------------------------------------------------------------
--------------------------------------------------------------------------------
--------------------------------------------------------------------------------
--------------------------------------------------------------------------------
--------------------------------------------------------------------------------
--------------------------------------------------------------------------------
--------------------------------------------------------------------------------
--------------------------------------------------------------------------------
--------------------------------------------------------------------------------
--------------------------------------------------------------------------------
--------------------------------------------------------------------------------
--------------------------------------------------------------------------------
--------------------------------------------------------------------------------
--------------------------------------------------------------------------------
--------------------------------------------------------------------------------
--------------------------------------------------------------------------------
--------------------------------------------------------------------------------
--------------------------------------------------------------------------------
--------------------------------------------------------------------------------
--------------------------------------------------------------------------------
------------------------GCTATTAACAAGAGATGGTGGTAATAACAACAATGGGTCCGAGATCTTCAGACCTG
GAGGAGGAGATATGAGGGACAATTGGAGAAGTGAATTATATAAATATAAAGTAGTAAAAATTGAACCATTAGGAGTAGCA
CCCACCAAGGCAAAGAGAAGAGTGGTGCAGAGAGAAAAAAGAGCAGTGGGAATAGGAGCTTTGTTCCTTGGGTTCTTGGG
AGCAGCAGGAAGCACTATGGGCGCAGCGTCAATGACGCTGACGGTACAGGCCAGACAATTATTGTCTGGTATAGTGCAGC
AGCAGAACAATTTGCTGAGGGCTATTGAGGCGCAACAGCATCTGTTGCAACTCACAGTCTGGGGCATCAAGCAGCTCCAG
GCAAGAATCCTGGCTGTGGAAAGATACCTAAAGGATCAACAGCTCCTGGGGATTTGGGGTTGCTCTGGAAAACTCATTTG
CACCACTGCTGTGCCTTGGAATGCTAGTTGGAGTAATAAATCTCTGGAACAGATTTGGAATCACACGACCTGGATGGAGT
GGGACAGAGAAATTAACAATTACACAAGCTTAATACACTCCTTAATTGAAGAATCGCAAAACCAGCAAGAAAAGAATGAA
CAAGAATTATTGGAATTAGATAAATGGGCAAGTTTGTGGAATTGGTTTAACATAACAAATTGGCTGTGGTATATAAAATT
ATTCATAATGATAGTAGGAGGCTTGGTAGGTTTAAGAATAGTTTTTGCTGTACTTTCTGTAGTGAATAGAGTTAGGCAGG
GATATTCACCATTATCGTTTCAGACCCACCTCCCAATCCCGAGGGGACCCGACAGGCCCGAAGGAATAGAAGAAGAAGGT
GGAGAGAGAGACAGAGACAGATCCATTCGATTAGTGAACGGATCCTTAGCACTTATCTGGGACGATCTGCGGAGCCTGTG
CCTCTTCAGCTACCACCGCTTGAGAGACTTACTCTTGATTGTAACGAGGATTGTGGAACTTCTGGGACGCAGGGGGTGGG
AAGCCCTCAAATATTGGTGGAATTTCCTACAATATTGGAGTCAGGAGCTAAAGAATAGTGCTGTTAGCTTGCTCAATGCC
ACAGCTATAGCAGTAGCTGAGGGGACAGATAGGGTTATAGAAGTAGTACAAGAAGCTTATAGAGCTATTCGCCACATACC
TAGAAGAATAAGACAGGGCTTGGAAAGGATTTTGCTATAAGATGGGTGGCAAGTGGTCAAAAAGTAGTGTGGTTGGATGG
CCTGCTGTAAGGGAAAGAATGAGACGAGCTGAGCCAGCAGCAGATGGGGTGGGAGCAGCATCTCGAGACCTAGAAAAACA
TGGAGCAATCACAAGTAGCAACACAGCAGCTAACAATGCTGCTTGTGCCTGGCTAGAAGCACAAGAGGAGGAGAAGGTGG
GTTTTCCAGTCACACCTCAGGTACCTTTAAGACCAATGACTTACAAGGCAGCTGTAGATCTTAGCCACTTTTTAAAAGAA
AAGGGGGGACTGGAAGGGCTAATTCACTCCCAACGAAGACAAGATATCCTTGATCTGTGGATCTACCACACACAAGGCTA
CTTCCCTGATTGGCAGAACTACACACCAGGACCAGGGATCAGATATCCACTGACCTTTGGATGGCGCTACAAGCTAGTAC
CAGTTGAGCCAGAGAAGTTAGAAGAAGCCAACAAAGGAGAGAACACCAGCTTGTTACACCCTGTGAGCCTGCATGGAATG
GATGACCCGGAGAGAGAAGTGTTAGAGTGGAGGTTTGACAGCCGCCTAGCATTTCATCACGTGGCCCGAGAGCTGCATCC
GGAGTACTTCAAGAACTGCTGATATCGAGCTTGCTACAAGGGACTTTCCGCTGGGGACTTTCCAGGGAGGCGTGGCCTGG
GCGGGACTGGGGAGTGGCGAGCCCTCAGATCCTGCATATAAGCAGCTGCTTTTTGCCTGTACTGGGTCTCTCTGGTTAGA
CCAGATCTGAGCCTGGGAGCTCTCTGGCTAACTAGGGAACCCACTGCTTAAGCCTCAATAAAGCTTGCCTTGAGTGCTTC
               AAGTAGTGTGTGCCCATCTGTTGTGTGACTCTGGTAACTAGAGATCCCTCA
                                   >2-45envLTR
--------------------------------------------------------------------------------
--------------------------------------------------------------------------------
--------------------------------------------------------------------------------
--------------------------------------------------------------------------------
--------------------------------------------------------------------------------
--------------------------------------------------------------------------------
--------------------------------------------------------------------------------
--------------------------------------------------------------------------------
--------------------------------------------------------------------------------
--------------------------------------------------------------------------------
--------------------------------------------------------------------------------
--------------------------------------------------------------------------------
--------------------------------------------------------------------------------
--------------------------------------------------------------------------------
--------------------------------------------------------------------------------
--------------------------------------------------------------------------------
--------------------------------------------------------------------------------
--------------------------------------------------------------------------------
--------------------------------------------------------------------------------
--------------------------------------------------------------------------------
--------------------------------------------------------------------------------
--------------------------------------------------------------------------------
--------------------------------------------------------------------------------
--------------------------------------------------------------------------------
--------------------------------------------------------------------------------
--------------------------------------------------------------------------------
```

```
--------------------------------------------------------------------------------
--------------------------------------------------------------------------------
--------------------------------------------------------------------------------
--------------------------------------------------------------------------------
--------------------------------------------------------------------------------
--------------------------------------------------------------------------------
--------------------------------------------------------------------------------
--------------------------------------------------------------------------------
--------------------------------------------------------------------------------
--------------------------------------------------------------------------------
--------------------------------------------------------------------------------
--------------------------------------------------------------------------------
--------------------------------------------------------------------------------
--------------------------------------------------------------------------------
--------------------------------------------------------------------------------
--------------------------------------------------------------------------------
--------------------------------------------------------------------------------
--------------------------------------------------------------------------------
--------------------------------------------------------------------------------
--------------------------------------------------------------------------------
--------------------------------------------------------------------------------
--------------------------------------------------------------------------------
--------------------------------------------------------------------------------
--------------------------------------------------------------------------------
--------------------------------------------------------------------------------
--------------------------------------------------------------------------------
--------------------------------------------------------------------------------
--------------------------------------------------------------------------------
--------------------------------------------------------------------------------
--------------------------------------------------------------------------------
--------------------------------------------------------------------------------
--------------------------------------------------------------------------------
--------------------------------------------------------------------------------
--------------------------------------------------------------------------------
--------------------------------------------------------------------------------
--------------------------------------------------------------------------------
--------------------------------------------------------------------------------
--------------------------------------------------------------------------------
--------------------------------------------------------------------------------
--------------------------------------------------------------------------------
--------------------------------------------------------------------------------
--------------------------------------------------------------------------------
--------------------------------------------------------------------------------
--------------------------------------------------------------------------------
--------------------------------------------------------------------------------
--------------------------------------------------------------------------------
--------------------------------------------------------------------------------
--------------------------------------------------------------------------------
--------------------------------------------------------------------------------
--------------------------------------------------------------------------------
--------------------------------------------------------------------------------
--------------------------------------------------------------------------------
--------------------------------------------------------------------------------
--------------------------------------------------------------------------------
--------------------------------------------------------------------------------
--------------------------------------------------------------------------------
--------------------------------------------------------------------------------
--------------------------------------------------------------------------------
--------------------------------------------------------------------------------
--------------------------------------------------------------------------------
--------------------------------------------------------------------------------
--------------------------------------------------------------------------------
--------------------------------------------------------------------------------
--------------------------------------------------------------------------------
--------------------------------------------------------------------------------
--------------------------------------------------------------------------------
--------------------------------------------------------------------------------
--------------------------------------------------------------------------------
-----------------------TGCTATTAACAAGAGATGGTGGTAATAACAACAATGGGTCCGAGATCTTCANACCTG
GAGGAGGAGATATGAGGGACAATTGGAGAAGTGAATTATATAAATATAAAGTAGTAAAAATTGAACCATTAGGAGTAGCA
CCCACCAAGGCAAAGAGAAGAGTGGTGCAGAGAGAAAAAAGAGCAGTGGGAATAGGAGCTTTGTTCCTTGGGTTCTTGGG
AGCAGCAGGAAGCACTATGGGCGCAGCGTCAATGACGCTGACGGTACAGGCCAGACAATTATTGTCTGGTATAGTGCAGC
AGCAGAACAATTTGCTGAGGGCTATTGAGGCGCAACAGCATCTGTTGCAACTCACAGTCTGGGGCATCAAGCAGCTCCAG
GCAAGAATCCTGGCTGTGGAAAGATACCTAAAGGATCAACAGCTCCTGGGGATTTGGGGTTGCTCTGGAAAACTCATTTG
CACCACTGCTGTGCCTTGGAATGCTAGTTGGAGTAATAAATCTCTGGAACAGATTTGGAATCACACGACCTGGATGGAGT
GGGACAGAGAAATTAACAATTACACAAGCTTAATACACTCCTTAATTGAAGAATCGCAAAACCAGCAAGAAAAGAATGAA
CAAGAATTATTGGAATTAGATAAATGGGCAAGTTTGTGGAATTGGTTTAACATAACAAATTGGCTGTGGTATATAAAATT
ATTCATAATGATAGTAGGAGGCTTGGTAGGTTTAAGAATAGTTTTTGCTGTACTTTCTGTAGTGAATAGAGTTAGGCAGG
GATATTCACCATTATCGTTTCAGACCCACCTCCCAATCCCGAGGGGACCCGACAGGCCCGAAGGAATAGAAGAAGAAGGT
GGAGAGAGAGACAGAGACAGATCCATTCGATTAGTGAACGGATCCTTAGCACTTATCTGGGACGATCTGCGGAGCCTGTG
CCTCTTCAGCTACCACCGCTTGAGAGACTTACTCTTGATTGTAACGAGGATTGTGGAACTTCTGGGACGCAGGGGGTGGG
AAGCCCTCAAATATTGGTGGAATCTCCTACAATATTGGAGTCAGGAGCTAAAGAATAGTGCTGTTAGCTTGCTCAATGCC
ACAGCTATAGCAGTAGCTGAGGGGACAGATAGGGTTATAGAAGTAGTACAAGAAGCTTATAGAGCTATTCGCCACATACC
TAGAAGAATAAGACAGGGCTTGGAAAGGATTTTGCTATAAGATGGGTGGCAAGTGGTCAAAAAGTAGTGTGGTTGGATGG
CCTGCTGTAAGGGAAAGAATGAGACGAGCTGAGCCAGCAGCAGATGGGGTGGGAGCAGCATCTCGAGACCTAGAAAAACA
TGGAGCAATCACAAGTAGCAACACAGCAGCTAACAATGCTGCTTGTGCCTGGCTAGAAGCACAAGAGGAGGAGAAGGTGG
GTTTTCCAGTCACACCTCAGGTACCTTTAAGACCAATGACTTACAAGGCAGCTGTAGATCTTAGCCACTTTTTAAAAGAA
AAGGGGGGACTGGAAGGGCTAATTCACTCCCAACGAAGACAAGATATCCTTGATCTGTGGATCTACCACACACAAGGCTA
CTTCCCTGATTGGCAGAACTACACACCAGGACCAGGGATCAGATATCCACTGACCTTTGGATGGCGCTACAAGCTAGTAC
CAGNTGAGCCAGAGAAGTTAGAAGAAGCCAACAAAGGAGAGAACACCAGCTTGTTACACCCTGTGAGCCTGCATGGAATG
GATGACCCGGAGAGAGAAGTGTTAGAGTGGAGGTTTGACAGCCGCCTAGCATTTCATCACGTGGCCCGAGAGCTGCATCC
GGAGTACTTCAAGAACTGCTGATATCGAGCTTGCTACAAGGGACTTTCCGCTGGGGACTTTCCAGGGAGGCGTGGCCTGG
GCGGGACTGGGGAGTGGCGAGCCCTCAGATCCTGCATATAAGCAGCTGCTTTTTGCCTGTACTGGGTCTCTCTGGTTAGA
CCAGATCTGAGCCTGGGAGCTCTCTGGCTAACTAGGGAACCCACTGCTTAAGCCTCAATAAAGCTTGCCTTGAGTGCTTC
```

```
AAGTAGTGTGTGCCCATCTGTTGTGTGACTCTGGTAACTAGAGATCCCTCA
>2-46envLTR
--------------------------------------------------------------------------------
--------------------------------------------------------------------------------
--------------------------------------------------------------------------------
--------------------------------------------------------------------------------
--------------------------------------------------------------------------------
--------------------------------------------------------------------------------
--------------------------------------------------------------------------------
--------------------------------------------------------------------------------
--------------------------------------------------------------------------------
--------------------------------------------------------------------------------
--------------------------------------------------------------------------------
--------------------------------------------------------------------------------
--------------------------------------------------------------------------------
--------------------------------------------------------------------------------
--------------------------------------------------------------------------------
--------------------------------------------------------------------------------
--------------------------------------------------------------------------------
--------------------------------------------------------------------------------
--------------------------------------------------------------------------------
--------------------------------------------------------------------------------
--------------------------------------------------------------------------------
--------------------------------------------------------------------------------
--------------------------------------------------------------------------------
--------------------------------------------------------------------------------
--------------------------------------------------------------------------------
--------------------------------------------------------------------------------
--------------------------------------------------------------------------------
--------------------------------------------------------------------------------
--------------------------------------------------------------------------------
--------------------------------------------------------------------------------
--------------------------------------------------------------------------------
--------------------------------------------------------------------------------
--------------------------------------------------------------------------------
--------------------------------------------------------------------------------
--------------------------------------------------------------------------------
--------------------------------------------------------------------------------
--------------------------------------------------------------------------------
--------------------------------------------------------------------------------
--------------------------------------------------------------------------------
--------------------------------------------------------------------------------
--------------------------------------------------------------------------------
--------------------------------------------------------------------------------
--------------------------------------------------------------------------------
--------------------------------------------------------------------------------
--------------------------------------------------------------------------------
--------------------------------------------------------------------------------
--------------------------------------------------------------------------------
--------------------------------------------------------------------------------
--------------------------------------------------------------------------------
--------------------------------------------------------------------------------
--------------------------------------------------------------------------------
--------------------------------------------------------------------------------
--------------------------------------------------------------------------------
--------------------------------------------------------------------------------
--------------------------------------------------------------------------------
--------------------------------------------------------------------------------
--------------------------------------------------------------------------------
--------------------------------------------------------------------------------
--------------------------------------------------------------------------------
--------------------------------------------------------------------------------
--------------------------------------------------------------------------------
--------------------------------------------------------------------------------
--------------------------------------------------------------------------------
--------------------------------------------------------------------------------
--------------------------------------------------------------------------------
--------------------------------------------------------------------------------
--------------------------------------------------------------------------------
--------------------------------------------------------------------------------
--------------------------------------------------------------------------------
--------------------------------------------------------------------------------
--------------------------------------------------------------------------------
--------------------------------------------------------------------------------
--------------------------------------------------------------------------------
--------------------------------------------------------------------------------
--------------------------------------------------------------------------------
--------------------------------------------------------------------------------
--------------------------------------------------------------------------------
--------------------------------------------------------------------------------
--------------------------------------------------------------------------------
--------------------------------------------------------------------------------
--------------------------------------------------------------------------------
--------------------------------------------------------------------------------
--------------------------------------------------------------------------------
--------------------------------------------------------------------------------
--------------------------------------------------------------------------------
--------------------------------------------------------------------------------
--------------------------------------------------------------------------------
--------------------------------------------------------------------------------
--------------------------------------------------------------------------------
--------------------------------------------------------------------------------
--------------------------------------------------------------------------------
```


----------------------CTGCTATTAACAAGAGATGGTGGTAATAACAACAATGGGTCCGAGATCTTCAGACCTG
GAGGAGGAGATATGAGGGACAATTGGAGAAGTGAATTATATAAATATAAAGTAGTAAAAATTGAACCATTAGGAGTAGCA
CCCACCAAGGCAAAGAGAAGAGTGGTGCAGAGAGAAAAAAGAGCAGTGGGAATAGGAGCTTTGTTCCTTGGGTTCTTGGG
AGCAGCAGGAAGCACTATGGGCGCAGCGTCAATGACGCTGACGGTACAGGCCAGACAATTATTGTCTGGTATAGTGCAGC
AGCAGAACAATTTGCTGAGGGCTATTGAGGCGCAACAGCATCTGTTGCAACTCACAGTCTGGGGCATCAAGCAGCTCCAG
GCAAGAATCCTGGCTGTGGAAAGATACCTAAAGGATCAACAGCTCCTGGGGATTTGGGGTTGCTCTGGAAAACTCATTTG
CACCACTGCTGTGCCTTGGAATGCTAGTTGGAGTAATAAATCTCTGGAACAGATTTGGAATCACACGACCTGGATGGAGT
GGGACAGAGAAATTAACAATTACACAAGCTTAATACACTCCTTAATTGAAGAATCGCAAAACCAGCAAGAAAAGAATGAA
CAAGAATTATTGGAATTAGATAAATGGGCAAGTTTGTGGAATTGGTTTAACATAACAAATTGGCTGTGGTATATAAAATT
ATTCATAATGATAGTAGGAGGCTTGGTAGGTTTAAGAATAGTTTTTGCTGTACTTTCTGTAGTGAATAGAGTTAGGCAGG
GATATTCACCATTATCGTTTCAGACCCACCTCCCAATCCCGAGGGGACCCGACAGGCCCGAAGGAATAGAAGAAGAAGGT
GGAGAGAGAGACAGAGACAGATCCATTCGATTAGTGAACGGATCCTTAGCACTTATCTGGGACGATCTGCGGAGCCTGTG
CCTCTTCAGCTACCACCGCTTGAGAGACTTACTCTTGATTGTAACGAGGATTGTGGAACTTCTGGGACGCAGGGGGTGGG
AAGCCCTCAAATATTGGTGGAATCTCCTACAATATTGGAGTCAGGAGCTAAAGAATAGTGCTGTTAGCTTGCTCAATGCC
ACAGCTATAGCAGTAGCTGAGGGGACAGATAGGGTTATAGAAGTAGTACAAGAAGCTTATAGAGCTATTCGCCACATACC
TAGAAGAATAAGACAGGGCTTGGAAAGGATTTTGCTATAAGATGGGTGGCAAGTGGTCAAAAAGTAGTGTGGTTGGATGG
CCTGCTGTAAGGGAAAGAATGAGACGAGCTGAGCCAGCAGCAGATGGGGTGGGAGCAGCATCTCGAGACCTAGAAAAACA
TGGAGCAATCACAAGTAGCAACACAGCAGCTAACAATGCTGCTTGTGCCTGGCTAGAAGCACAAGAGGAGGAGAAGGTGG
GTTTTCCAGTCACACCTCAGGTACCTTTAAGACCAATGACTTACAAGGCAGCTGTAGATCTTAGCCACTTTTTAAAAGAA
AAGGGGGGACTGGAAGGGCTAATTCACTCCCAACGAAGACAAGATATCCTTGATCTGTGGATCTACCACACACAAGGCTA
CTTCCCTGATTGGCAGAACTACACACCAGGACCAGGGATCAGATATCCACTGACCTTTGGATGGCGCTACAAGCTAGTAC
CAGTTGAGCCAGAGAAGTTAGAAGAAGCCAACAAAGGAGAGAACACCAGCTTGTTACACCCTGTGAGCCTGCATGGAATG
GATGACCCGGAGAGAGAAGTGTTAGAGTGGAGGTTTGACAGCCGCCTAGCATTTCATCACGTGGCCCGAGAGCTGCATCC
GGAGTACTTCAAGAACTGCTGATATCGAGCTTGCTACAAGGGACTTTCCGCTGGGGACTTTCCAGGGAGGCGTGGCCTGG
GCGGGACTGGGGAGTGGCGAGCCCTCAGATCCTGCATATAAGCAGCTGCTTTTTGCCTGTACTGGGTCTCTCTGGTTAGA
CCAGATCTGAGCCTGGGAGCTCTCTGGCTAACTAGGGAACCCACTGCTTAAGCCTCAATAAAGCTTGCCTTGAGTGCTTC
AAGTAGTGTGTGCCCATCTGTTGTGTGACTCTGGTAACTAGAGATCCCTCA
>2-47envLTR

--AAAGCAATGTATGCCCCTCCCATCAGCGGACAAATNAG
ATNTTCATCAAATNCTACAGGGNTGCTATTAACAAGAGATGGTGGTAATAACAACAATGGGTCCGAGATNNTCAGACCTG
GAGGAGGAGATATGAGGGACAATTGGAGAAGTGAATTATATAAATATAAAGTAGTAAAAATTGAACCNTTAGGAGTAGCA
CCCACCAAGGCAAAGAGAAGAGTGGTGCAGAGAGAAAAAAGAGCAGTGGGAATAGGAGCTTTGTTCCTTGGGTTCTTGGG
AGCAGCAGGAAGCACTATGGGCGCAGCGTCAATGACGCTGACGGTACAGGCCAGACAATTATTGTCCGGTATAGTGCAGC
AGCAGAACAATTTGCTGAGGGCTATTGAGGCGCAACAGCATCTGTTGCAACTCACAGTCTGGGGCATCAAGCAGCTCCAG
GCAAGAATCCTGGCTGTGGAAAGATACCTAAAGGATCAACAGCTCCTGGGGATTTGGGGTTGCTCTGGAAAACTCATTTG
CACCACTGCTGTGCCTTGGAATGCTAGTTGGAGTAATAAATCTCTGGAACAGATTTGGAATCACACGACCTGGATGGAGT
GGGACAGAGAAATTAACAATTACACAAGCTTAATACACTCCTTAATTGAAGAATCGCAAAACCAGCAAGAAAAGAATGAA
CAAGAATTATTGGAATTAGATAAATGGGCAAGTTTGTGGAATTGGTTTAACATAACAAATTGGCTGTGGTATATAAAATT
ATTCATAATGATAGTAGGAGGCTTGGTAGGTTTAAGAATAGTNTNTGCTGTACTTTCTGTAGTGAATAGAGTTAGGCAGG
GATATTCACCATTATCGTTTCAGACCCACCTCCCAATCCCGAGGGGACCCGACAGGCCCGAAGGAATAGAAGAAGAAGGT
GGAGAGAGAGACAGAGACAGATCCATTCGATTAGTGAACGGATCCTTAGCACTTATCTGGGACGATCTGCGGAGCCTGTG
CCTCTTCAGCTACCACCGCTTGAGAGACTTACTCTTGATTGTAACGAGGATTGTGGAACTTCTGGGACGCAGGGGGTGGG
AAGCCCTCAAATATTGGTGGAATCTCCTACAATATTGGAGTCAGGAGCTAAAGAATAGTGCTGTTAGCTTGCTCAATGCC
ACAGCTATAGCAGTAGCTGAGGGGACAGATAGGGTTATAGAAGTAGTACAAGAAGCTTATAGAGCTATTCGCCACATACC
TAGAAGAATAAGACAGGGCTTGGAAAGGATTTTGCTATAAGATGGGTGGCAAGTGGTCAAAAAGTAGTGTGGTTGGATGG
CCTGCTGTAAGGGAAAGAATGAGACGAGCTGAGCCAGCAGCAGATGGGGTGGGAGCAGCATCTCGAGACCTAGAAAAACA
TGGAGCAATCACAAGTAGCAACACAGCAGCTAACAATGCTGCTTGTGCCTGGCTAGAAGCACAAGAGGAGGAGAAGGTGG
GTTTTCCAGTCACACCTCAGGTACCTTTAAGACCAATGACTTACAAGGCAGCTGTAGATCTTAGCCACTTTTTAAAAGAA
AAGGGGGGACTGGAAGGGCTAATTCACTCCCAACGAAGACAAGATATCCTTGATCTGTGGATCTACCACACACAAGGCTA
CTTCCCTGATTGGCAGAACTACACACCAGGACCAGGGATCAGATATCCACTGACCTTTGGATGGCGCTACAAGCTAGTAC
CAGTTGAGCCAGAGAAGTTAGAAGAAGCCAACAAAGGAGAGAACACCAGCTTGTTACACCCTGTGAGCCTGCATGGAATG
GATGACCCGGAGAGAGAAGTGTTAGAGTGGAGGTTTGACAGCCGCCTAGCATTTCATCACGTGGCCCGAGAGCTGCATCC
GGAGTACTTCAAGAACTGCTGATATCGAGCTTGCTACAAGGGACTTTCCGCTGGGGACTTTCCAGGGAGGCGTGGCCTGG
GCGGGACTGGGGAGTGGCGAGCCCTCAGATCCTGCATATAAGCAGCTGCTTTTTGCCTGTACTGGGTCTCTCTGGTTAGA
CCAGATCTGAGCCTGGGAGCTCTCTGGCTAACTAGGGAACCCACTGCTTAAGCCTCAATAAAGCTTGCCTTGAGTGCTTC
AAGTAGTGTGTGCCCATCTGTTGTGTGACTCTGGTAACTAGAGATCCCTCA
>2-49envLTR

----------------GGACAATTGGAGAAGTGAATTATATAAATATAAAGTAGTAAAAATTGAACAATTAGGAGTAGCA
CCCACCAAGGCAAAGAGAAGAGTGGTGCAGAGAGAAAAAAGAGCAGTGGGAATAGGAGCTTTGTTCCTTGGGTTCTTGGG
AGCAGCAGGAAGCACTATGGGCGCAGCGTCAATGACGCTGACGGTACAGGCCAGACAATTATTGTCTGGTATAGTGCAGC
AGCAGAACAATTTGCTGAGGGCTATTGAGGCGCAACAGCATCTGTTGCAACTCACAGTCTGGGGCATCAAGCAGCTCCAG
GCAAGAATCCTGGCTGTGGAAAGATACCTAAAGGATCAACAGCTCCTGGGGATTTGGGGTTGCTCTGGAAAACTCATTTG
CACCACTGCTGTGCCTTGGAATGCTAGTTGGAGTAATAAATCTCTGGAACAGATTTGGAATCACACGACCTGGATGGAGT
GGGACAGAGAAATTAACAATTACACAAGCTTAATACACTCCTTAATTGAAGAATCGCAAAACCAGCAAGAAAAGAATGAA
CAAGAATTATTGGAATTAGATAAATGGGCAAGTTTGTGGAATTGGTTTAACATAACAAATTGGCTGTGGTATATAAAATT
ATTCATAATGATAGTAGGAGGCTTGGTAGGTTTAAGAATAGTTTTTGCTGTACTTTCTGTAGTGAATAGAGTTAGGCAGG
GATATTCACCATTATCGTTTCAGACCCACCTCCCAATCCCGAGGGGACCCGACAGGCCCGAAGGAATAGAAGAAGAAGGT
GGAGAGAGAGACAGAGACAGATCCATTCGATTAGTGAACGGATCCTTAGCACTTATCTGGGACGATCTGCGGAGCCTGTG
CCTCTTCAGCTACCACCGCTTGAGAGACTTACTCTTGATTGTAACGAGGATTGTGGAACTTCTGGGACGCAGGGGGTGGG
AAGCCCTCAAATATTGGTGGAATCTCCTACAATATTGGAGTCAGGAGCTAAAGAATAGTGCTGTTAGCTTGCTCAATGCC
ACAGCTATAGCAGTAGCTGAGGGGACAGATAGGGTTATAGAAGTAGTACAAGAAGCTTATAGAGCTATTCGCCACATACC
TAGAAGAATAAGACAGGGCTTGGAAAGGATTTTGCTATAAGATGGGTGGCAAGTGGTCAAAAAGTAGTGTGGTTGGATGG
CCTGCTGTAAGGGAAAGAATGAGACGAGCTGAGCCAGCAGCAGATGGGGTGGGAGCAGCATCTCGAGACCTAGAAAAACA
TGGAGCAATCACAAGTAGCAACACAGCAGCTAACAATGCTGCTTGTGCCTGGCTAGAAGCACAAGAGGAGGAGAAGGTGG
GTTTTCCAGTCACACCTCAGGTACCTTTAAGACCAATGACTTACAAGGCAGCTGTAGATCTTAGCCACTTTTTAAAAGAA
AAGGGGGGACTGGAAGGGCTAATTCACTCCCAACGAAGACAAGATATCCTTGATCTGTGGATCTACCACACACAAGGCTA
CTTCCCTGATTGGCAGAACTACACACCAGGACCAGGGATCAGATATCCACTGACCTTTGGATGGCGCTACAAGCTAGTAC
CAGTTGAGCCAGAGAAGTTAGAAGAAGCCAACAAAGGAGAGAACACCAGCTTGTTACACCCTGTGAGCCTGCATGGAATG
GATGACCCGGAGAGAGAAGTGTTAGAGTGGAGGTTTGACAGCCGCCTAGCATTTCATCACGTGGCCCGAGAGCTGCATCC
GGAGTACTTCAAGAACTGCTGATATCGAGCTTGCTACAAGGGACTTTCCGCTGGGGACTTTCCAGGGAGGCGTGGCCTGG
GCGGGACTGGGGAGTGGCGAGCCCTCAGATCCTGCATATAAGCAGCTGCTTTTTGCCTGTACTGGGTCTCTCTGGTTAGA
CCAGATCTGAGCCTGGGAGCTCTCTGGCTAACTAGGGAACCCACTGCTTAAGCCTCAATAAAGCTTGCCTTGAGTGCTTC
AAGTAGTGTGTGCCCATCTGTTGTGTGACTCTGGTAACTAGAGATCCCTCA
>2-50envLTR


```
--------------------------------------------------------------------------------
--------------------------------------------------------------------------------
--------------------------------------------------------------------------------
--------------------------------------------------------------------------------
--------------------------------------------------------------------------------
--------------------------------------------------------------------------------
--------------------------------------------------------------------------------
--------------------------------------------------------------------------------
--------------------------------------------------------------------------------
--------------------------------------------------------------------------------
--------------------------------------------------------------------------------
--------------------------------------------------------------------------------
--------------------------------------------------------------------------------
--------------------------------------------------------------------------------
--------------------------------------------------------------------------------
--------------------------------------------------------------------------------
--------------------------------------------------------------------------------
--------------------------------------------------------------------------------
--------------------------------------------------------------------------------
--------------------------------------------------------------------------------
--------------------------------------------------------------------------------
--------------------------------------------------------------------------------
--------------------------------------------------------------------------------
--------------------------------------------------------------------------------
--------------------------------------------------------------------------------
--------------------------------------------------------------------------------
--------------------------------------------------------------------------------
--------------------------------------------------------------------------------
--------------------------------------------------------------------------------
--------------------------------------------------------------------------------
--------------------------------------------------------------------------------
--------------------------------------------------------------------------------
--------------------------------------------------------------------------------
--------------------------------------------------------------------------------
--------------------------------------------------------------------------------
--------------------------------------------------------------------------------
--------------------------------------------------------------------------------
--------------------------------------------------------------------------------
--------------------------------------------------------------------------------
--------------------------------------------------------------------------------
--------------------------------------------------------------------------------
--------------------------------------------------------------------------------
--------------------------------------------------------------------------------
--------------------------------------------------------------------------------
--------------------------------------------------------------------------------
--------------------------------------------------------------------------------
--------------------------------------------------------------------------------
--------------------------------------------------------------------------------
--------------------------------------------------------------------------------
--------------------------------------------------------------------------------
--------------------------------------------------------------------------------
--------------------------------------------------------------------------------
--------------------------------------------------------------------------------
--------------------------------------------------------------------------------
--------------------------------------------------------------------------------
--------------------------------------------------------------------------------
--------------------------------------------------------------------------------
--------------------------------------------------------------------------------
--------------------------------------------------------------------------------
--------------------------------------------------------------------------------
--------------------------------------------------------------------------------
--------------------------------------------------------------------------------
--------------------------------------------------------------------------------
--------------------------------------------------------------------------------
--------------------------------------------------------------------------------
--------------------------------------------------------------------------------
--------------------------------------------------------------------------------
--------------------------------------------------------------------------------
--------------------------------------------------------------------------------
--------------------------------------------------------------------------------
--------------------------------------------------------------------------------
--------------------------------------------------------------------------------
--------------------------------------------------------------------------------
--------------------------------------------------------------------------------
--------------------------------------------------------------------------------
--------------------------------------------------------------------------------
--------------------------------------------------------------------------------
--------------------------------------------------------------------------------
--------------------------------------------------------------------------------
--------------------------------------------------------------------------------
--------------------------------------------------------------------------------
--------------------------------------------------------------------------------
--------------------------------------------------------------------------------
--------------------------------------------------------------------------------
--------------------------------------------------------------------------------
--------------------------------------------------------------------------------
--------------------------------------------------------------------------------
--------------------------------------------------------------------------------
--GGAGGAGANATGAGGGACAATTGGAGAAGTGAATTATATAAATATAAAGTAGTAAAAATTGAACNATTAGGAGTAGCA
CCCACCAAGGCAAAGAGAAGAGTGGTGCAGAGAGAAAAAAGAGCAGTGGGAATAGGAGCTTTGTTCCTTGGGTTCTTGGG
AGCAGCAGGAAGCACTATGGGCGCAGCGTCAATGACGCTGACGGTACAGGCCAGACAATTATTGTCTGGTATAGTGCAGC
AGCAGAACAATTTGCTGAGGGCTATTGAGGCGCAACAGCATCTGTTGCAACTCACAGTCTGGGGCATCAAGCAGCTCCAG
GCAAGAATCCTGGCTGTGGAAAGATACCTAAAGGATCAACAGCTCCTGGGGATTTGGGGTTGCTCTGGAAAACTCATTTG
CACCACTGCTGTGCCTTGGAATGCTAGTTGGAGTAATAAATCTCTGGAACAGATTTGGAATCACACGACCTGGATGGAGT
GGGACAGAGAAATTAACAATTACACAAGCTTAATACACTCCTTAATTGAAGAATCGCAAAACCAGCAAGAAAAGAATGAA
```

```
CAAGAATTATTGGAATTAGATAAATGGGCAAGTTTGTGGAATTGGTTTAACATAACAAATTGGCTGTGGTATATAAAATT
ATTCATAATGATAGTAGGAGGCTTGGTAGGTTTAAGAATAGTTTTTGCTGTACTTTCTGTAGTGAATAGAGTTAGGCAGG
GATATTCACCATTATCGTTTCAGACCCACCTCCCAATCCCGAGGGGACCCGACAGGCCCGAAGGAATAGAAGAAGAAGGT
GGAGAGAGAGACAGAGACAGATCCATTCGATTAGTGAACGGATCCTTAGCACTTATCTGGGACGATCTGCGGAGCCTGTG
CCTCTTCAGCTACCACCGCTTGAGAGACTTACTCTTGATTGTAACGAGGATTGTGGAACTTCTGGGACGCAGGGGGTGGG
AAGCCCTCAAATATTGGTGGAATCTCCTACAATATTGGAGTCAGGAGCTAAAGAATAGTGCTGTTAGCTTGCTCAATGCC
ACAGCTATAGCAGTAGCTGAGGGGACAGATAGGGTTATAGAAGTAGTACAAGAAGCTTATAGAGCTATTCGCCACATACC
TAGAAGAATAAGACAGGGCTTGGAAAGGATTTTGCTATAAGATGGGTGGCAAGTGGTCAAAAAGTAGTGTGGTTGGATGG
CCTGCTGTAAGGGAAAGAATGAGACGAGCTGAGCCAGCAGCAGATGGGGTGGGAGCAGCATCTCGAGACCTAGAAAAACA
TGGAGCAATCACAAGTAGCAACACAGCAGCTAACAATGCTGCTTGTGCCTGGCTAGAAGCACAAGAGGAGGAGAAGGTGG
GTTTTCCAGTCACACCTCAGGTACCTTTAAGACCAATGACTTACAAGGCAGCTGTAGATCTTAGCCACTTTTTAAAAGAA
AAGGGGGGACTGGAAGGGCTAATTCACTCCCAACGAAGACAAGATATCCTTGATCTGTGGATCTACCACACACAAGGCTA
CTTCCCTGATTGGCAGAACTACACACCAGGACCAGGGATCAGATATCCACTGACCTTTGGATGGCGCTACAAGCTAGTAC
CAGTTGAGCCAGAGAAGTTAGAAGAAGCCAACAAAGGAGAGAACACCAGCTTGTTACACCCTGTGAGCCTGCATGGAATG
GATGACCCGGAGAGAGAAGTGTTAGAGTGGAGGTTTGACAGCCGCCTAGCATTTCATCACGTGGCCCGAGAGCTGCATCC
GGAGTACTTCAAGAACTGCTGATATCGAGCTTGCTACAAGGGACTTTCCGCTGGGGACTTTCCAGGGAGGCGTGGCCTGG
GCGGGACTGGGGAGTGGCGAGCCCTCAGATCCTGCATATAAGCAGCTGCTTTTTGCCTGTACTGGGTCTCTCTGGTTAGA
CCAGATCTGAGCCTGGGAGCTCTCTGGCTAACTAGGGAACCCACTGCTTAAGCCTCAATAAAGCTTGCCTTGAGTGCTTC
AAGTAGTGTGTGCCCATCTGTTGTGTGACTCTGGTAACTAGAGATCCCTCA
```

>2-62envLTR

```
--------------------------------------------------------------------------------
--------------------------------------------------------------------------------
--------------------------------------------------------------------------------
--------------------------------------------------------------------------------
--------------------------------------------------------------------------------
--------------------------------------------------------------------------------
--------------------------------------------------------------------------------
--------------------------------------------------------------------------------
--------------------------------------------------------------------------------
--------------------------------------------------------------------------------
--------------------------------------------------------------------------------
--------------------------------------------------------------------------------
--------------------------------------------------------------------------------
--------------------------------------------------------------------------------
--------------------------------------------------------------------------------
--------------------------------------------------------------------------------
--------------------------------------------------------------------------------
--------------------------------------------------------------------------------
--------------------------------------------------------------------------------
--------------------------------------------------------------------------------
--------------------------------------------------------------------------------
--------------------------------------------------------------------------------
--------------------------------------------------------------------------------
--------------------------------------------------------------------------------
--------------------------------------------------------------------------------
--------------------------------------------------------------------------------
--------------------------------------------------------------------------------
--------------------------------------------------------------------------------
--------------------------------------------------------------------------------
--------------------------------------------------------------------------------
--------------------------------------------------------------------------------
--------------------------------------------------------------------------------
--------------------------------------------------------------------------------
--------------------------------------------------------------------------------
--------------------------------------------------------------------------------
--------------------------------------------------------------------------------
--------------------------------------------------------------------------------
--------------------------------------------------------------------------------
--------------------------------------------------------------------------------
--------------------------------------------------------------------------------
--------------------------------------------------------------------------------
--------------------------------------------------------------------------------
--------------------------------------------------------------------------------
--------------------------------------------------------------------------------
--------------------------------------------------------------------------------
--------------------------------------------------------------------------------
--------------------------------------------------------------------------------
--------------------------------------------------------------------------------
--------------------------------------------------------------------------------
--------------------------------------------------------------------------------
--------------------------------------------------------------------------------
--------------------------------------------------------------------------------
--------------------------------------------------------------------------------
--------------------------------------------------------------------------------
--------------------------------------------------------------------------------
--------------------------------------------------------------------------------
--------------------------------------------------------------------------------
--------------------------------------------------------------------------------
--------------------------------------------------------------------------------
--------------------------------------------------------------------------------
--------------------------------------------------------------------------------
--------------------------------------------------------------------------------
--------------------------------------------------------------------------------
--------------------------------------------------------------------------------
--------------------------------------------------------------------------------
--------------------------------------------------------------------------------
--------------------------------------------------------------------------------
--------------------------------------------------------------------------------
--------------------------------------------------------------------------------
--------------------------------------------------------------------------------
--------------------------------------------------------------------------------
--------------------------------------------------------------------------------
--------------------------------------------------------------------------------
--------------------------------------------------------------------------------
```


----------------------------------AGAGATGGTGGTAATAACAACAATGGGTCCGAGATCTTCAGACCTG
GAGGAGGAGATATGAGGGACAATTGGAGAAGTGAATTATATAAATATAAAGTAGTAAAAATTGAACCATTAGGAGTAGCA
CCCACCAAGGCAAAGAGAAGAGTGGTGCAGAGAGAAAAAAGAGCAGTGGGAATAGGAGCTTTGTTCCTTGGGTTCTTGGG
AGCAGCAGGAAGCACTATGGGCGCAGCGTCAATGACGCTGACGGTACAGGCCAGACAATTATTGTCTGGTATAGTGCAGC
AGCAGAACAATTTGCTGAGGGCTATTGAGGCGCAACAGCATCTGTTGCAACTCACAGTCTGGGGCATCAAGCAGCTCCAG
GCAAGAATCCTGGCTGTGGAAAGATACCTAAAGGATCAACAGCTCCTGGGGATTTGGGGTTGCTCTGGAAAACTCATTTG
CACCACTGCTGTGCCTTGGAATGCTAGTTGGAGTAATAAATCTCTGGAACAGATTTGGAATCACACGACCTGGATGGAGT
GGGACAGAGAAATTAACAATTACACAAGCTTAATACACTCCTTAATTGAAGAATCGCAAAACCAGCAAGAAAAGAATGAA
CAAGAATTATTGGAATTAGATAAATGGGCAAGTTTGTGGAATTGGTTTAACATAACAAATTGGCTGTGGTATATAAAATT
ATTCATAATGATAGTAGGAGGCTTGGTAGGTTTAAGAATAGTTTTTGCTGTACTTTCTGTAGTGAATAGAGTTAGGCAGG
GATATTCACCATTATCGTTTCAGACCCACCTCCCAATCCCGAGGGGACCCGACAGGCCCGAAGGAATAGAAGAAGAAGGT
GGAGAGAGAGACAGAGACAGATCCATTCGATTAGTGAACGGATCCTTAGCACTTATCTGGGACGATCTGCGGAGCCTGTG
CCTCTTCAGCTACCACCGCTTGAGAGACTTACTCTTGATTGTAACGAGGATTGTGGAACTTCTGGGACGCAGGGGGTGGG
AAGCCCTCAAATATTGGTGGAATCTCCTACAATATTGGAGTCAGGAGCTAAAGAATAGTGCTGTTAGCTTGCTCAATGCC
ACAGCTATAGCAGTAGCTGAGGGGACAGATAGGGTTATAGAAGTAGTACAAGAAGCTTATAGAGCTATTCGCCACATACC
TAGAAGAATAAGACAGGGCTTGGAAAGGATTTTGCTATAAGATGAGTGGCAAGTGGTCAAAAAGTAGTGTGGTTGGATGG
CCTGCTGTAAGGGAAAGAATGAGACGAGCTGAGCCAGCAGCAGATGGGGTGGGAGCAGCATCTCGAGACCTAGAAAAACA
TGGAGCAATCACAAGTAGCAACACAGCAGCTAACAATGCTGCTTGTGCCTGGCTAGAAGCACAAGAGGAGGAGAAGGTGG
GTTTTCCAGTCACACCTCAGGTACCTTTAAGACCAATGACTTACAAGGCAGCTGTAGATCTTAGCCACTTTTTAAAAGAA
AAGGGGGGACTGGAAGGGCTAATTCACTCCCAACGAAGACAAGATATCCTTGATCTGTGGATCTACCACACACAAGGCTA
CTTCCCTGATTGGCAGAACTACACACCAGGACCAGGGATCAGATATCCACTGACCTTTGGATGGCGCTACAAGCTAGTAC
CAGTTGAGCCAGAGAAGTTAGAAGAAGCCAACAAAGGAGAGAACACCAGCTTGTTACACCCTGTGAGCCTGCATGGAATG
GATGACCCGGAGAGAGAAGTGTTAGAGTGGAGGTTTGACAGCCGCCTAGCATTTCATCACGTGGCCCGAGAGCTGCATCC
GGAGTACTTCAAGAACTGCTGATATCGAGCTTGCTACAAGGGACTTTCCGCTGGGGACTTTCCAGGGAGGCGTGGCCTGG
GCGGGACTGGGGAGTGGCGAGCCCTCAGATCCTGCATATAAGCAGCTGCTTTTTGCCTGTACTGGGTCTCTCTGGTTAGA
CCAGATCTGAGCCTGGGAGCTCTCTGGCTAACTAGGGAACCCACTGCTTAAGCCTCAATAAAGCTTGCCTTGAGTGCTTC
AAGTAGTGTGTGCCCATCTGTTGTGTGACTCTGGTAACTAGAGATCCCTCA

>2-69envLTR


```
--------------------------------------------------------------------------------
--------------------------------------------------------------------------------
--------------------------------------------------------------------------------
--------------------------------------------------------------------------------
--------------------------------------------------------------------------------
--------------------------------------------------------------------------------
--------------------------------------------------------------------------------
--------------------------------------------------------------------------------
--------------------------------------------------------------------------------
--------------------------------------------------------------------------------
--------------------------------------------------------------------------------
--------------------------------------------------------------------------------
--------------------------------------------------------------------------------
--------------------------------------------------------------------------------
--------------------------------------------------------------------------------
--------------------------------------------------------------------------------
--------------------------------------------------------------------------------
--------------------------------------------------------------------------------
--------------------------------------------------------------------------------
--------------------------------------------------------------------------------
--------------------------------------------------------------------------------
--------------------------------------------------------------------------------
--------------------------------------------------------------------------------
--------------------------------------------------------------------------------
--------------------------------------------------------------------------------
--------------------------------------------------------------------------------
--------------------------------------------------------------------------------
--------------------------------------------------------------------------------
--------------------------------------------------------------------------------
--------------------------------------------------------------------------------
--------------------------------------------------------------------------------
--------------------------------------------------------------------------------
--------------------------------------------------------------------------------
--------------------------------------------------------------------------------
--------------------------------------------------------------------------------
--------------------------------------------------------------------------------
--------------------------------------------------------------------------------
--------------------------------------------------------------------------------
--------------------------------------------------------------------------------
--------------------------------------------------------------------------------
--------------------------------------------------------------------------------
--------------------------------------------------------------------------------
--------------------------------------------------------------------------------
--------------------------------------------------------------------------------
--------------------------------------------------------------------------------
--------------------------------------------------------------------------------
--------------------------------------------------------------------------------
--------------------------------------------------------------------------------
--------------------------------------------------------------------------------
--------------------------------------------------------------------------------
------------------------------------------GCAGTGGGAATAGGAGCTTTGTTCCTTGGGTTCTTGGG
AGCAGCAGGAAGCACTATGGGCGCAGCGTCAATGACGCTGACGGTACAGGCCAGACAATTATTGTCTGGTATAGTGCAGC
AGCAGAACAATTTGCTGAGGGCTATTGAGGCGCAACAGCATCTGTTGCAACTCACAGTCTGGGGCATCAAGCAGCTCCAG
GCAAGAATCCTGGCTGTGGAAAGATACCTAAAGGATCAACAGCTCCTGGGGATTTGGGGTTGCTCTGGAAAACTCATTTG
CACCACTGCTGTGCCTTGGAATGCTAGTTGGAGTAATAAATCTCTGGAACAGATTTGGAATCACACGACCTGGATGGAGT
GGGACAGAGAAATTAACAATTACACAAGCTTAATACACTCCTTAATTGAAGAATCGCAAAACCAGCAAGAAAAGAATGAA
CAAGAATTATTGGAATTAGATAAATGGGCAAGTTTGTGGAATTGGTTTAACATAACAAATTGGCTGTGGTATATAAAATT
ATTCATAATGATAGTAGGAGGCTTGGTAGGTTTAAGAATAGTTTTTGCTGTACTTTCTGTAGTGAATAGAGTTAGGCAGG
GATATTCACCATTATCGTTTCAGACCCACCTCCCAATCCCGAGGGGACCCGACAGGCCCGAAGGAATAGAAGAAGAAGGT
GGAGAGAGAGACAGAGACAGATCCATTCGATTAGTGAACGGATCCTTAGCACTTATCTGGGACGATCTGCGGAGCCTGTG
CCTCTTCAGCTACCACCGCTTGAGAGACTTACTCTTGATTGTAACGAGGATTGTGGAACTTCTGGGACGCAGGGGGTGGG
AAGCCCTCAAATATTGGTGGAATCTCCTACAATATTGGAGTCAGGAGCTAAAGAATAGTGCTGTTAGCTTGCTCAATGCC
ACAGCTATAGCAGTAGCTGAGGGGACAGATAGGGTTATAGAAGTAGTACAAGAAGCTTATAGAGCTATTCGCCACATACC
TAGAAGAATAAGACAGGGCTTGGAAAGGATTTTGCTATAAGATGGGTGGCAAGTGGTCAAAAAGTAGTGTGGTTGGATGG
CCTGCTGTAAGGGAAAGAATGAGACGAGCTGAGCCAGCAGCAGATGGGGTGGGAGCAGCATCTCGAGACCTAGAAAAACA
TGGAGCAATCACAAGTAGCAACACAGCAGCTAACAATGCTGCTTGTGCCTGGCTAGAAGCACAAGAGGAGGAGAAGGTGG
GTTTTCCAGTCACACCTCAGGTACCTTTAAGACCAATGACTTACAAGGCAGCTGTAGATCTTAGCCACTTTTTAAAAGAA
AAGGGGGGACTGGAAGGGCTAATTCACTCCCAACGAAGACAAGATATCCTTGATCTGTGGATCTACCACACACAAGGCTA
CTTCCCTGATTGGCAGAACTACACACCAGGACCAGGGATCAGATATCCACTGACCTTTGGATGGCGCTACAAGCTAGTAC
CAGTTGAGCCAGAGAAGTTAGAAGAAGCCAACAAAGGAGAGAACACCAGCTTGTTACACCCTGTGAGCCTGCATGGAATG
GATGACCCGGAGAGAGAAGTGTTAGAGTGGAGGTTTGACAGCCGCCTAGCATTTCATCACGTGGCCCGAGAGCTGCATCC
GGAGTACTTCAAGAACTGCTGATATCGAGCTTGCTACAAGGGACTTTCCGCTGGGGACTTTCCAGGGAGGCGTGGCCTGG
GCGGGACTGGGGAGTGGCGAGCCCTCAGATCCTGCATATAAGCAGCTACTTTTTGCCTGTACTGGGTCTCTCTGGTTAGA
CCAGATCTGAGCCTGGGAGCTCTCTGGCTAACTAGGGAACCCACTGCTTAAGCCTCAATAAAGCTTGCCTTGAGTGCTTC
AAGTAGTGTGTGCCCATCTGTTGTGTGACTCTGGTAACTAGAGATCCCTCA
>2-72envLTR
--------------------------------------------------------------------------------
--------------------------------------------------------------------------------
--------------------------------------------------------------------------------
--------------------------------------------------------------------------------
--------------------------------------------------------------------------------
--------------------------------------------------------------------------------
--------------------------------------------------------------------------------
--------------------------------------------------------------------------------
--------------------------------------------------------------------------------
--------------------------------------------------------------------------------
--------------------------------------------------------------------------------
--------------------------------------------------------------------------------
--------------------------------------------------------------------------------
--------------------------------------------------------------------------------
--------------------------------------------------------------------------------
--------------------------------------------------------------------------------
--------------------------------------------------------------------------------
--------------------------------------------------------------------------------
```

```
--------------------------------------------------------------------------------
--------------------------------------------------------------------------------
--------------------------------------------------------------------------------
--------------------------------------------------------------------------------
--------------------------------------------------------------------------------
--------------------------------------------------------------------------------
--------------------------------------------------------------------------------
--------------------------------------------------------------------------------
--------------------------------------------------------------------------------
--------------------------------------------------------------------------------
--------------------------------------------------------------------------------
--------------------------------------------------------------------------------
--------------------------------------------------------------------------------
--------------------------------------------------------------------------------
--------------------------------------------------------------------------------
--------------------------------------------------------------------------------
--------------------------------------------------------------------------------
--------------------------------------------------------------------------------
--------------------------------------------------------------------------------
--------------------------------------------------------------------------------
--------------------------------------------------------------------------------
--------------------------------------------------------------------------------
--------------------------------------------------------------------------------
--------------------------------------------------------------------------------
--------------------------------------------------------------------------------
--------------------------------------------------------------------------------
--------------------------------------------------------------------------------
--------------------------------------------------------------------------------
--------------------------------------------------------------------------------
--------------------------------------------------------------------------------
--------------------------------------------------------------------------------
--------------------------------------------------------------------------------
--------------------------------------------------------------------------------
--------------------------------------------------------------------------------
--------------------------------------------------------------------------------
--------------------------------------------------------------------------------
--------------------------------------------------------------------------------
--------------------------------------------------------------------------------
--------------------------------------------------------------------------------
--------------------------------------------------------------------------------
--------------------------------------------------------------------------------
--------------------------------------------------------------------------------
--------------------------------------------------------------------------------
--------------------------------------------------------------------------------
--------------------------------------------------------------------------------
--------------------------------------------------------------------------------
--------------------------------------------------------------------------------
--------------------------------------------------------------------------------
--------------------------------------------------------------------------------
--------------------------------------------------------------------------------
--------------------------------------------------------------------------------
--------------------------------------------------------------------------------
--------------------------------------------------------------------------------
--------------------------------------------------------------------------------
--------------------------------------------------------------------------------
--------------------------------------------------------------------------------
--------------------------------------------------------------------------------
--------------------------------------------------------------------------------
--------------------------------------------------------------------------------
--------------------------------------------------------------------------------
--------------------------------------------------------------------------------
--------------------------------------------------------------------------------
--------------------------------------------------------------------------------
--------------------------------------------------------------------------------
--------------------------------------------------------------------------------
--------------------------------------------------------------------------------
--------------------------------------------------------------------------------
--------------------------------------------------------------------------------
--------------------------------------------------------------------------------
--------------------------------------------------------------------------------
--------------------------------------------------------------------------------
--------------------------------------------------------------------------------
--------------------------------------------------------------------------------
--------------------------------------------------------------------------------
--------------------------------------------------------------------------------
--------------------------------------------------------------------------------
--------------------------------------------------------------------------------
-----------------------------------GAGATGGTGGTAATAACAACAATGGGTCCGAGATCTTCAGACCTG
GAGGAGGAGATATGAGGGACAATTGGAGAAGTGAATTATATAAATATAAAGTAGTAAAAATTGAACCATTAGGAGTAGCA
CCCACCAAGGCAAAGAGAAGAGTGGTGCAGAGAGAAAAAAGAGCAGTGGGAATAGGAGCTTTGTTCCTTGGGTTCTTGGG
AGCAGCAGGAAGCACTATGGGCGCAGCGTCAATGACGCTGACGGTACAGGCCAGACAATTATTGTCTGGTATAGTGCAGC
AGCAGAACAATTTGCTGAGGGCTATTGAGGCGCAACAGCATCTGTTGCAACTCACAGTCTGGGGCATCAAGCAGCTCCAG
GCAAGAATCCTGGCTGTGGAAAGATACCTAAAGGATCAACAGCTCCTGGGGATTTGGGGTTGCTCTGGAAAACTCATTTG
CACCACTGCTGTGCCTTGGAATGCTAGTTGGAGTAATAAATCTCTGGAACAGATTTGGAATCACACGACCTGGATGGAGT
GGGACAGAGAAATTAACAATTACACAAGCTTAATACACTCCTTAATTGAAGAATCGCAAAACCAGCAAGAAAAGAATGAA
CAAGAATTATTGGAATTAGATAAATGGGCAAGTTTGTGGAATTGGTTTAACATAACAAATTGGCTGTGGTATATAAAATT
ATTCATAATGATAGTAGGAGGCTTGGTAGGTTTAAGAATAGTTTTTGCTGTACTTTCTGTAGTGAATAGAGTTAGGCAGG
GATATTCACCATTATCGTTTCAGACCCACCTCCCAATCCCGAGGGGACCCGACAGGCCCGAAGGAATAGAAGAAGAAGGT
GGAGAGAGAGACAGAGACAGATCCATTCGATTAGTGAACGGATCCTTAGCACTTATCTGGGACGATCTGCGGAGCCTGTG
CCTCTTCAGCTACCACCGCTTGAGAGACTTACTCTTGATTGTAACGAGGATTGTGGAACTTCTGGGACGCAGGGGGTGGG
AAGCCCTCAAATATTGGTGGAATCTCCTACAATATTGGAGTCAGGAGCTAAAGAATAGTGCTGTTAGCTTGCTCAATGCC
ACAGCTATAGCAGTAGCTGAGGGGACAGATAGGGTTATAGAAGTAGTACAAGAAGCTTATAGAGCTATTCGCCACATACC
TAGAAGAATAAGACAGGGCTTGGAAAGGATTTTGCTATAAGATGGGTGGCAAGTGGTCAAAAAGTAGTGTGGTTGGATGG
CCTGCTGTAAGGGAAAGAATGAGACGAGCTGAGCCAGCAGCAGATGGGGTGGGAGCAGCATCTCGAGACCTAGAAAAACA
TGGAGCAATCACAAGTAGCAACACAGCAGCTAACAATGCTGCTTGTGCCTGGCTAGAAGCACAAGAGGAGGAGAAGGTGG
```

GTTTTCCAGTCACACCTCAGGTACCTTTAAGACCAATGACTTACAAGGCAGCTGTAGATCTTAGCCACTTTTTAAAAGAA
AAGGGGGGACTGGAAGGGCTAATTCACTCCCAACGAAGACAAGATATCCTTGATCTGTGGATCTACCACACACAAGGCTA
CTTCCCTGATTGGCAGAACTACACACCAGGACCAGGGATCAGATNTCCACTGACCTTTGGATGGCGCTACAAGCTAGTAC
CAGTTGAGCCAGAGAAGTTAGAAGAAGCCAACAAAGGAGAGAACACCAGCTTGTTACACCCTGTGAGCCTGCATGGAATG
GATGACCCGGAGAGAGAAGTGTTAGAGTGGAGGTTTGACAGCCGCCTAGCATTTCATCACGTGGCCCGAGAGCTGCATCC
GGAGTACTTCAAGAACTGCTGATATCGAGCTTGCTACAAGGGACTTTCCGCTGGGGACTTTCCAGGGAGGCGTGGCCTGG
GCGGGACTGGGGAGTGGCGAGCCCTCAGATCCTGCATATAAGCAGCTGCTTTTTGCCTGTACTGGGTCTCTCTGGTTAGA
CCAGATCTGAGCCTGGGAGCTCTCTGGCTAACTAGGGAACCCACTGCTTAAGCCTCAATAAAGCTTGCCTTGAGTGCTTC
AAGTAGTGTGTGCCCATCTGTTGTGTGACTCTGGTAACTAGAGATCCCTCA
>2-76envLTR

--GGTGGTAATAACAACAATGGGTCCGAGATCTTCAGACCTG
GAGGAGGAGATATGAGGGACAATTGGAGAAGTGAATTATATAAATATAAAGTAGTAAAAATTGAACCATTAGGAGTAGCA
CCCACCAAGGCAAAGAGAAGAGTGGTGCAGAGAGAAAAAAGAGCAGTGGGAATAGGAGCTTTGTTCCTTGGGTTCTTGGG
AGCAGCAGGAAGCACTATGGGCGCAGCGTCAATGACGCTGACGGTACAGGCCAGACAATTATTGTCTGGTATAGTGCAGC
AGCAGAACAATTTGCTGAGGGCTATTGAGGCGCAACAGCATCTGTTGCAACTCACAGTCTGGGGCATCAAGCAGCTCCAG
GCAAGAATCCTGGCTGTGGAAAGATACCTAAAGGATCAACAGCTCCTGGGGATTTGGGGTTGCTCTGGAAAACTCATTTG
CACCACTGCTGTGCCTTGGAATGCTAGTTGGAGTAATAAATCTCTGGAACAGATTTGGAATCACACGACCTGGATGGAGT
GGGACAGAGAAATTAACAATTACACAAGCTTAATACACTCCTTAATTGAAGAATCGCAAAACCAGCAAGAAAAGAATGAA
CAAGAATTATTGGAATTAGATAAATGGGCAAGTTTGTGGAATTGGTTTAACATAACAAATTGGCTGTGGTATATAAAATT
ATTCATAATGATAGTAGGAGGCTTGGTAGGTTTAAGAATAGTTTTTGCTGTACTTTCTGTAGTGAATAGAGTTAGGCAGG
GATATTCACCATTATCGTTTCAGACCCACCTCCCAATCCCGAGGGGACCCGACAGGCCCGAAGGAATAGAAGAAGAAGGT
GGAGAGAGAGACAGAGACAGATCCATTCGATTAGTGAACGGATCCTTAGCACTTATCTGGGACGATCTGCGGAGCCTGTG
CCTCTTCAGCTACCACCGCTTGAGAGACTTACTCTTGATTGTAACGAGGATTGTGGAACTTCTGGGACGCAGGGGGTGGG
AAGCCCTCAAATATTGGTGGAATCTCCTACAATATTGGAGTCAGGAGCTAAAGAATAGTGCTGTTAGCTTGCTCAACGCC
ACAGCTATAGCAGTAGCTGAGGGGACAGATAGGGTTATAGAAGTAGTACAAGAAGCTTATAGAGCTATTCGCCACATACC
TAGAAGAATAAGACAGGGCTTGGAAAGGATTTTGCTATAAGATGGGTGGCAAGTGGTCAAAAAGTAGTGTGGTTGGATGG
CCTGCTGTAAGGGAAAGAATGAGACGAGCTGAGCCAGCAGCAGATGGGGTGGGAGCAGCATCTCGAGACCTAGAAAAACA
TGGAGCAATCACAAGTAGCAACACAGCAGCTAACAATGCTGCTTGTGCCTGGCTAGAAGCACAAGAGGAGGAGAAGGTGG
GTTTTCCAGTCACACCTCAGGTACCTTTAAGACCAATGACTTACAAGGCAGCTGTAGATCTTAGCCACTTTTTAAAAGAA
AAGGGGGGACTGGAAGGGCTAATTCACTCCCAACGAAGACAAGATATCCTCGATCTGTGGATCTACCACACACAAGGCTA
CTTCCCTGATTGGCAGAACTACACACCAGGACCAGGGATCAGATATCCACTGACCTTTGGATGGCGCTACAAGCTAGTAC
CAGTTGAGCCAGAGAAGTTAGAAGAAGCCAACAAAGGAGAGAACACCAGCTTGTTACACCCTGTGAGCCTGCATGGAATG
GATGACCCGGAGAGAGAAGTGTTAGAGTGGAGGTTTGACAGCCGCCTAGCATTTCATCACGTGGCCCGAGAGCTGCATCC
GGAGTACTTCAAGAACTGCTGATATCGAGCTTGCTACAAGGGACTTTCCGCTGGGGACTTTCCAGGGAGGCGTGGCCTGG
GCGGGACTGGGGAGTGGCGAGCCCTCAGATCCTGCATATAAGCAGCTGCTTTTTGCCTGTACTGGGTCTCTCTGGTTAGA
CCAGATCTGAGCCTGGGAGCTCTCTGGCTAACTAGGGAACCCACTGCTTAAGCCTCAATAAAGCTTGCCTTGAGTGCTTC
AAGTAGTGTGTGCCCATCTGTTGTGTGACTCTGGTAACTAGAGATCCCTCA
>2-84envLTR


```
--------------------------------------------------------------------------------
--------------------------------------------------------------------------------
--------------------------------------------------------------------------------
--------------------------------------------------------------------------------
--------------------------------------------------------------------------------
--------------------------------------------------------------------------------
--------------------------------------------------------------------------------
--------------------------------------------------------------------------------
--------------------------------------------------------------------------------
--------------------------------------------------------------------------------
--------------------------------------------------------------------------------
--------------------------------------------------------------------------------
--------------------------------------------------------------------------------
--------------------------------------------------------------------------------
--------------------------------------------------------------------------------
--------------------------------------------------------------------------------
--------------------------------------------------------------------------------
--------------------------------------------------------------------------------
--------------------------------------------------------------------------------
--------------------------------------------------------------------------------
--------------------------------------------------------------------------------
--------------------------------------------------------------------------------
--------------------------------------------------------------------------------
--------------------------------------------------------------------------------
--------------------------------------------------------------------------------
--------------------------------------------------------------------------------
--------------------------------------------------------------------------------
--------------------------------------------------------------------------------
--------------------------------------------------------------------------------
--------------------------------------------------------------------------------
--------------------------------------------------------------------------------
--------------------------------------------------------------------------------
--------------------------------------------------------------------------------
--------------------------------------------------------------------------------
--------------------------------------------------------------------------------
--------------------------------------------------------------------------------
--------------------------------------------------------------------------------
--------------------------------------------------------------------------------
-----------------------------------------------ATAACAACAATGGGTCCGAGATCTTCANACCTG
GAGGAGGAGATATGAGGGACAATTGGAGAAGTGAATTATATAAATATAAAGTAGTAAAAATTGAACCATTAGGAGTAGCA
CCCACCAAGGCAAAGAGAAGAGTGGTGCAGAGAGAAAAAAGAGCAGTGGGAATAGGAGCTTTGTTCCTTGGGTTCTTGGG
AGCAGCAGGAAGCACTATGGGCGCAGCGTCAATGACGCTGACGGTACAGGCCAGACAATTATTGTCTGGTATAGTGCAGC
AGCAGAACAATTTGCTGAGGGCTATTGAGGCGCAACAGCATCTGTTGCAACTCACAGTCTGGGGCATCAAGCAGCTCCAG
GCAAGAATCCTGGCTGTGGAAAGATACCTAAAGGATCAACAGCTCCTGGGGATTTGGGGTTGCTCTGGAAAACTCATTTG
CACCACTGCTGTGCCTTGGAATGCTAGTTGGAGTAATAAATCTCTGGAACAGATTTGGAATCACACGACCTGGATGGAGT
GGGACAGAGAAATTAACAATTACACAAGCTTAATACACTCCTTAATTGAAGAATCGCAAAACCAGCAAGAAAAGAATGAA
CAAGAATTATTGGAATTAGATAAATGGGCAAGTTTGTGGAATTGGTTTAACATAACAAATTGGCTGTGGTATATAAAATT
ATTCATAATGATAGTAGGAGGCTTGGTAGGTTTAAGAATAGTTTTTGCTGTACTTTCTGTAGTGAATAGAGTTAGGCAGG
GATATTCACCATTATCGTTTCAGACCCACCTCCCAATCCCGAGGGGACCCGACAGGCCCGAAGGAATAGAAGAAGAAGGT
GGAGAGAGAGACAGAGACAGATCCATTCGATTAGTGAACGGATCCTTAGCACTTATCTGGGACGATCTGCGGAGCCTGTG
CCTCTTCAGCTACCACCGCTTGAGAGACTTACTCTTGATTGTAACGAGGATTGTGGAACTTCTGGGACGCAGGGGGTGGG
AAGCCCTCAAATATTGGTGGAATCTCCTACAATATTGGAGTCAGGAGCTAAAGAATAGTGCTGTTAGCTTGCTCAATGCC
ACAGCTATAGCAGTAGCTGAGGGGACAGATAGGGTTATAGAAGTAGTACAAGAAGCTTATAGAGCTATTCGCCACATACC
TAGAAGAATAAGACAGGGCTTGGAAAGGATTTTGCTATAAGATGGGTGGCAAGTGGTCAAAAAGTAGTGTGGTTGGATGG
CCTGCTGTAAGGGAAAGAATGAGACGAGCTGAGCCAGCAGCAGATGGGGTGGGAGCAGCATCTCGAGACCTAGAAAAACA
TGGAGCAATCACAAGTAGCAACACAGCAGCTAACAATGCTGCTTGTGCCTGGCTAGAAGCACAAGAGGAGGAGAAGGTGG
GTTTTCCAGTCACACCTCAGGTACCTTTAAGACCAATGACTTACAAGGCAGCTGTAGATCTTAGCCACTTTTTAAAAGAA
AAGGGGGGACTGGAAGGGCTAATTCACTCCCAACGAAGACAAGATATCCTTGATCTGTGGATCTACCACACACAAGGCTA
CTTCCCTGATTGGCAGAACTACACACCAGGACCAGGGATCAGATATCCACTGACCTTTGGATGGCGCTACAAGCTAGTAC
CAGTTGAGCCAGAGAAGTTAGAAGAAGCCAACAAAGGAGAGAACACCAGCTTGTTACACCCTGTGAGCCTGCATGGAATG
GATGACCCGGAGAGAGAAGTGTTAGAGTGGAGGTTTGACAGCCGCCTAGCATTTCATCACGTGGCCCGAGAGCTGCATCC
GGAGTACTTCAAGAACTGCTGATATCGAGCTTGCTACAAGGGACTTTCCGCTGGGGACTTTCCAGGGAGGCGTGGCCTGG
GCGGGACTGGGGAGTGGCGAGCCCTCAGATCCTGCATATAAGCAGCTGCTTTTTGCCTGTACTGGGTCTCTCTGGTTAGA
CCAGATCTGAGCCTGGGAGCTCTCTGGCTAACTAGGGAACCCACTGCTTAAGCCTCAATAAAGCTTGCCTTGAGTGCTTC
AAGTAGTGTGTGCCCATCTGTTGTGTGACTCTGGTAACTAGAGATCCCTCA
>3-2envLTR
--------------------------------------------------------------------------------
--------------------------------------------------------------------------------
--------------------------------------------------------------------------------
--------------------------------------------------------------------------------
--------------------------------------------------------------------------------
--------------------------------------------------------------------------------
--------------------------------------------------------------------------------
--------------------------------------------------------------------------------
--------------------------------------------------------------------------------
--------------------------------------------------------------------------------
--------------------------------------------------------------------------------
--------------------------------------------------------------------------------
--------------------------------------------------------------------------------
--------------------------------------------------------------------------------
--------------------------------------------------------------------------------
--------------------------------------------------------------------------------
--------------------------------------------------------------------------------
--------------------------------------------------------------------------------
--------------------------------------------------------------------------------
--------------------------------------------------------------------------------
--------------------------------------------------------------------------------
--------------------------------------------------------------------------------
--------------------------------------------------------------------------------
--------------------------------------------------------------------------------
--------------------------------------------------------------------------------
--------------------------------------------------------------------------------
--------------------------------------------------------------------------------
--------------------------------------------------------------------------------
```


--------------------------------CAAGAGATGGTGGTAATAACAACAATGGGTCCGAGATCTTCAGACCTG
GAGGAGGAGATATGAAGGACAATTGAAGAAGTGAATTATATAAATATAAAGTAGTAAAAATTGAACCATTAGAAGTAGCA
CCCACCAAGGCAAAGAGAAGAGTGGTGCAGAGAGAAAAAAGAGCAGTAGGAATAGGAGCTTTGTTCCTTAGGTTCTTAGG
AGCAGCAGGAAGCACTATAGGCGCAGCGTCAATGACGCTGACGGTACAGGCCAGACAATTATTGTCTGGTATAGTGCAGC
AGCAGAACAATTTGCTGAGGGCTATTGAGGCGCAACAGCATCTGTTGCAACTCACAGTCTAGGGCATCAAGCAGCTCCAG
GCAAGAATCCTGGCTGTAGAAAGATACCTAAAGGATCAACAGCTCCTAAAGATTTAGGGTTGCTCTGGAAAACTCATTTG
CACCACTGCTGTGCCTTGGAATGCTAGTTGGAGTAATAAATCTCTGGAACAGATTTAGAATCACACGACCTGGATGGAGT
AGGACAGAGAAATTAACAATTACACAAGCTTAATACACTCCTTAATTGAAGAATCGCAAAACCAGCAAGAAAAGAATAAA
CAAGAATTATTAGAATTAGATAAATAGGCAAGTTTGTAGAATTAGTTTAACATAACAAATTGGCTGTGGTATATAAAATT
ATTCATAATGATAGTAGGAGGCTTGGTAAGTTTAAGAATAGTTTTTGCTGTACTTTCTGTAGTGAATAGAGTTAGGCAGG
GATATTCACCATTATCGTTTCAGACCCACCTCCCAATCCCGAGGGGACCCGACAGGCCCGAAGGAATAGAAGAAGAAGGT
AGAGAGAGAGACAGAGACAGATCCATTCGATTAGTGAACGGATCCTTAGCACTTATCTAGGACGATCTGCGGAGCCTGTG
CCTCTTCAGCTACCACCGCTTAAGAGACTTACTCTTGATTGTAACGAGGATTGTAGAACTTCTAGGACGCAGGGGGTGGG
AAGCCCTCAAATATTGGTAGAATCTCCTACAATATTAGAGTCAGGAGCTAAAAAATAGTGCTGTTAGCTTGCTCAATGCC
ACAGCTATAGCAGTAGCTGAAAGGACAGATAAGGTTATAGAAGTAGTACAAGAAGCTTATAGAGCTATTCGCCACATACC
TAGAAGAATAAGACAGGGCTTAGAAAAGATTTTGCTATAAGATAGGTGGCAAGTGGTCAAAAAGTAGTGTGGTTGGATGG
CCTGCTGTAAAGGAAAGAATGAGACGAGCTGAGCCAGCAGCAGATGAGGTAGGAGCAGCATCTCGAGACCTAGAAAAACA
TAGAGCAATCACAAGTAGCAACACAGCAGCTAACAATGCTGCTTGTGCCTGGCTAGAAGCACAAGAGGAAGAGAAAGTAA
GTTTTCCAGTCACACCTCAGGTACCTTTAAGACCAATGACTTACAAGGCAGCTGTAGATCTTAGCCACTTTTTAAAAGAA
AAGGGGGGACTGGAAGGGCTAATTCACTCCCAACGAAGACAAGATATCCTTGATCTGTGGATCTACCACACACAAGGCTA
CTTCCCTGATTGGCAGAACTACACACCAGGACCAGGGATCAGATATCCACTGACCTTTAGATGGCGCTACAAGCTAGTAC
CAGTTGAGCCAGAGAAGTTAGAAGAAGCCAACAAAGGAGAGAACACCAGCTTGTTACACCCTGTGAGCCTGCATAGAATG
GATGACCCGGAGAGAGAAGTGTTAGAGTGGAAGTTTGACAGCCGCCTAGCATTTCATCACGTGGCCCGAGAGCTGCATCC
GGAGTACTTCAAGAACTGCTGATATCGAGCTTGCTACAAAGGACTTTCCGCTGAGGACTTTCCAGGGAGGCGTGGCCTAG
GCAGGACTAAGAAGTGGCGAGCCCTCAGATCCTGCATATAAGCAGCTGCTTTTTGCCTGTACTAGGTCTCTCTGGTTAGA
CCAGATCTGAGCCTAAGAGCTCTCTGGCTAACTAAGGAACCCACTGCTTAAGCCTCAATAAAGCTTGCCTTAAGTGCTTC
AAGTAGTGTGTGCCCATCTGTTGTGTGACTCTGGTAACTAGAGATCCCTCA
>3-3envLTR

----------------------CTGCTATTAACAAGAGATGGTGGTAATAACAACAATGGGTCCGAGATCTTCAGACCTG
GAGGAGGAGATATGAGGGACAATTGGAGAAGTGAATTATATAAATATAAAGTAGTAAAAATTGAACCATTAAGAGTAGCA
CCCACCAAGGCAAAGAGAAGAGTGGTGCAGAGAGAAAAAAGAGCAGTAGGAATAGGAGCTTTGTTCCTTAGGTTCTTAGG
AGCAGCAGGAAGCACTATAGGCGCAGCGTCAATGACGCTGACGGTACAGGCCAGACAATTATTGTCTGGTATAGTGCAGC
AGCAGAACAATTTGCTGAGGGCTATTGAGGCGCAACAGCATCTGTTGCAACTCACAGTCTGGGGCATCAAGCAGCTCCAG
GCAAGAATCCTGGCTGTGGAAAGATACCTAAAGGATCAACAGCTCCTGGGGATTTGGGGTTGCTCTGGAAAACTCATTTG
CACCACTGCTGTGCCTTGGAATGCTAGTTGGAGTAATAAATCTCTGGAACAGATTTAGAATCACACGACCTGGATGGAGT
AGGACAGAGAAATTAACAATTACACAAGCTTAATACACTCCTTAATTGAAGAATCGCAAAACCAGCAAGAAAAGAATGAA
CAAGAATTATTGGAATTAGATAAATGGGCAAGTTTGTGGAATTGGTTTAACATAACAAATTGGCTGTGGTATATAAAATT
ATTCATAATGATAGTAGGAGGCTTGGTAGGTTTAAGAATAGTTTTTGCTGTACTTTCTGTAGTGAATAGAGTTAGGCAGG
GATATTCACCATTATCGTTTCAGACCCACCTCCCAATCCCGAGGGGACCCGACAGGCCCGAAGGAATAGAAGAAGAAGGT
GGAGAGAGAGACAGAGACAGATCCATTCGATTAGTGAACGGATCCTTAGCACTTATCTGGGACGATCTGCGGAGCCTGTG
CCTCTTCAGCTACCACCGCTTGAGAGACTTACTCTTGATTGTAACGAGGATTGTGGAACTTCTAGGACGCAGGGGGTGGG
AAGCCCTCAAATATTGGTGGAATCTCCTACAATATTGGAGTCAGGAGCTAAAGAATAGTGCTGTTAGCTTGCTCAATGCC
ACAGCTATAGCAGTAGCTGAGAGGACAGATAGGGTTATAGAAGTAGTACAAGAAGCTTATAGAGCTATTCGCCACATACC
TAGAAGAATAAGACAAGGCTTGGAAAGGATTTTGCTATAAGATGGGTGGCAAGTGGTCAAAAAGTAGTGTGGTTGGATGG
CCTGCTGTAAGGGAAAGAATGAGACGAGCTGAGCCAGCAGCAGATAGGGTGGGAGCAGCATCTCGAGACCTAGAAAAACA
TGGAGCAATCACAAGTAGCAACACAGCAGCTAACAATGCTGCTTGTGCCTGGCTAGAAGCACAAGAGGAAGAGAAGGTAG
GTTTTCCAGTCACACCTCAGGTACCTTTAAGACCAATGACTTACAAGGCAGCTGTAGATCTTAGCCACTTTTTAAAAGAA
AAGGGGGGACTGGAAGGGCTAATTCACTCCCAACGAAGACAAGATATCCTTGATCTGTGGATCTACCACACACAAGGCTA
CTTCCCTGATTGGCAGAACTACACACCAGGACCAGGGATCAGATATCCACTGACCTTTGGATGGCGCTACAAGCTAGTAC
CAGTTGAGCCAGAGAAGTTAGAAGAAGCCAACAAAGGAGAGAACACCAGCTTGTTACACCCNGTGAGCCTGCATGGAATG
GATGACCCGGAGAGAGAAGTGTTAGAGTGGAGGTTTGACAGCCGCCTAGCATTTCATCACGTGGCCCGAGAGCTGCATCC
GGAGTACTTCAAGAACTGCTGATATCGAGCTTGCTACAAGGGACTTTCCGCTGGGGACTTTCCAGGGAGGCGTGGCCTGG
GCGGGACTGGGGAGTGGCGAGCCCTCAGATCCTGCATATAAGCAGCTGCTTTTTGCCTGTACTGGGTCTCTCTGGTTAGA
CCAGATCTGAGCCTGGGAGCTCTCTGGCTAACTAGGGAACCCACTGCTTAAGCCTCAATAAAGCTTGCCTTGAGTGCTTC
AAGTAGTGTGTGCCCATCTGTTGTGTGACTCTGGTAACTAGAGATCCCTCA
>3-11envLTR


```
--------------------------------------------------------------------------------
--------------------------------------------------------------------------------
--------------------------------------------------------------------------------
--------------------------------------------------------------------------------
--------------------------------------------------------------------------------
--------------------------------------------------------------------------------
--------------------------------------------------------------------------------
--------------------------------------------------------------------------------
--------------------------------------------------------------------------------
--------------------------------------------------------------------------------
--------------------------------------------------------------------------------
--------------------------------------------------------------------------------
--------------------------------------------------------------------------------
--------------------------------------------------------------------------------
--------------------------------------------------------------------------------
--------------------------------------------------------------------------------
--------------------------------------------------------------------------------
--------------------------------------------------------------------------------
--------------------------------------------------------------------------------
--------------------------------------------------------------------------------
--------------------------------------------------------------------------------
--------------------------------------------------------------------------------
--------------------------------------------------------------------------------
--------------------------------------------------------------------------------
--------------------------------------------------------------------------------
--------------------------------------------------------------------------------
--------------------------------------------------------------------------------
--------------------------------------------------------------------------------
-------------------------------------------------------------------------------G
GAGGAGGAGATATGAGGGACAATTGGAGAAGTGAATTATATAAATATAAAGTAGTAAAAATTGAACCATTAGGAGTAGCA
CCCACCAAGGCAAAGAGAAGAGTGGTGCAGAGAGAAAAAAGAGCAGTGGGAATAGGAGCTTTGTTCCTTGGGTTCTTGGG
AGCAGCAGGAAGCACTATGGGCGCAGCGTCAATGACGCTGACGGTACAGGCCAGACAATTATTGTCTGGTATAGTGCAGC
AGCAGAACAATTTGCTGAGGGCTATTGAGGCGCAACAGCATCTGTTGCAACTCACAGTCTGGGGCATCAAGCAGCTCCAG
GCAAGAATCCTGGCTGTGGAAAGATACCTAAAGGATCAACAGCTCCTGGGGATTTGGGGTTGCTCTGGAAAACTCATTTG
CACCACTGCTGTGCCTTGGAATGCTAGTTGGAGTAATAAATCTCTGGAACAGATTTGGAATCACACGACCTGGATGGAGT
GGGACAGAGAAATTAACAATTACACAAGCTTAATACACTCCTTAATTGAAGAATCGCAAAACCAGCAAGAAAAGAATGAA
CAAGAATTATTGGAATTAGATAAATGGGCAAGTTTGTGGAATTGGTTTAACATAACAAATTGGCTGTGGTATATAAAATT
ATTCATAATGATAGTAGGAGGCTTGGTAGGTTTAAGAATAGTTTTTGCTGTACTTTCTGTAGTGAATAGAGTTAGGCAGG
GATATTCACCATTATCGTTTCAGACCCACCTCCCAATCCCGAGGGGACCCGACAGGCCCGAAGGAATAGAAGAAGAAGGT
GGAGAGAGAGACAGAGACAGATCCATTCGATTAGTGAACGGATCCTTAGCACTTATCTGGGACGATCTGCGGAGCCTGTG
CCTCTTCAGCTACCACCGCTTGAGAGACTTACTCTTGATTGTAACGAGGATTGTGGAACTTCTGGGACGCAGGGGGTGGG
AAGCCCTCAAATATTGGTGGAATCTCCTACAATATTGGAGTCAGGAGCTAAAGAATAGTGCTGTTAGCTTGCTCAATGCC
ACAGCTATAGCAGTAGCTGAGGGGACAGATAGGGTTATAGAAGTAGTACAAGAAGCTTATAGAGCTATTCGCCACATACC
TAGAAGAATAAGACAGGGCTTGGAAAGGATTTTGCTATAAGATGGGTGGCAAGTGGTCAAAAAGTAGTGTGGTTGGATGG
CCTGCTGTAAGGGAAAGAATGAGACGAGCTGAGCCAGCAGCAGATGGGGTGGGAGCAGCATCTCGAGACCTAGAAAAACA
TGGAGCAATCACAAGTAGCAACACAGCAGCTAACAATGCTGCTTGTGCCTGGCTAGAAGCACAAGAGGAGGAGAAGGTGG
GTTTTCCAGTCACACCTCAGGTACCTTTAAGACCAATGACTTACAAGGCAGCTGTAGATCTTAGCCACTTTTTAAAAGAA
AAGGGGGGACTGGAAGGGCTAATTCACTCCCAACGAAGACAAGATATCCTTGATCTGNGGATCTACCACACACAAGGCTA
CTTCCCTGATTGGCAGAACTACACACCAGGACCAGGGATCAGATATCCACTGACCTTTGGATGGCGCTACAAGCTAGTAC
CAGTTGAGCCAGAGAAGTTAGAAGAAGCCAACAAAGGAGAGAACACCAGCTTGTTACACCCTGTGAGCCTGCATGGAATG
GATGACCCGGAGAGAGAAGTGTTAGAGTGGAGGTTTGACAGCCGCCTAGCATTTCATCACGTGGCCCGAGAGCTGCATCC
GGAGTACTTCAAGAACTGCTGATATCGAGCTTGCTACAAGGGACTTTCCGCTGGGGACTTTCCAGGGAGGCGTGGCCTGG
GCGGGACTGGGGAGTGGCGAGCCCTCAGATCCTGCATATAAGCAGCTGCTTTTTGCCTGTACTGGGTCTCTCTGGTTAGA
CCAGATCTGAGCCTGGGAGCTCTCTGGCTAACTAGGGAACCCACTGCTTAAGCCTCAATAAAGCTTGCCTTGAGTGCTTC
AAGTAGTGTGTGCCCATCTGTTGTGTGACTCTGGTAACTAGAGATCCCTCA
>3-14envLTR
--------------------------------------------------------------------------------
--------------------------------------------------------------------------------
--------------------------------------------------------------------------------
--------------------------------------------------------------------------------
--------------------------------------------------------------------------------
--------------------------------------------------------------------------------
--------------------------------------------------------------------------------
--------------------------------------------------------------------------------
--------------------------------------------------------------------------------
--------------------------------------------------------------------------------
--------------------------------------------------------------------------------
--------------------------------------------------------------------------------
--------------------------------------------------------------------------------
--------------------------------------------------------------------------------
--------------------------------------------------------------------------------
--------------------------------------------------------------------------------
--------------------------------------------------------------------------------
--------------------------------------------------------------------------------
--------------------------------------------------------------------------------
--------------------------------------------------------------------------------
--------------------------------------------------------------------------------
--------------------------------------------------------------------------------
--------------------------------------------------------------------------------
--------------------------------------------------------------------------------
--------------------------------------------------------------------------------
--------------------------------------------------------------------------------
--------------------------------------------------------------------------------
--------------------------------------------------------------------------------
--------------------------------------------------------------------------------
--------------------------------------------------------------------------------
--------------------------------------------------------------------------------
--------------------------------------------------------------------------------
--------------------------------------------------------------------------------
--------------------------------------------------------------------------------
--------------------------------------------------------------------------------
--------------------------------------------------------------------------------
--------------------------------------------------------------------------------
--------------------------------------------------------------------------------
```

```
--------------------------------------------------------------------------------
--------------------------------------------------------------------------------
--------------------------------------------------------------------------------
--------------------------------------------------------------------------------
--------------------------------------------------------------------------------
--------------------------------------------------------------------------------
--------------------------------------------------------------------------------
--------------------------------------------------------------------------------
--------------------------------------------------------------------------------
--------------------------------------------------------------------------------
--------------------------------------------------------------------------------
--------------------------------------------------------------------------------
--------------------------------------------------------------------------------
--------------------------------------------------------------------------------
--------------------------------------------------------------------------------
--------------------------------------------------------------------------------
--------------------------------------------------------------------------------
--------------------------------------------------------------------------------
--------------------------------------------------------------------------------
--------------------------------------------------------------------------------
--------------------------------------------------------------------------------
--------------------------------------------------------------------------------
--------------------------------------------------------------------------------
--------------------------------------------------------------------------------
--------------------------------------------------------------------------------
--------------------------------------------------------------------------------
--------------------------------------------------------------------------------
--------------------------------------------------------------------------------
--------------------------------------------------------------------------------
--------------------------------------------------------------------------------
--------------------------------------------------------------------------------
--------------------------------------------------------------------------------
--------------------------------------------------------------------------------
--------------------------------------------------------------------------------
--------------------------------------------------------------------------------
--------------------------------------------------------------------------------
--------------------------------------------------------------------------------
--------------------------------------------------------------------------------
--------------------------------------------------------------------------------
--------------------------------------------------------------------------------
--------------------------------------------------------------------------------
--------------------------------------------------------------------------------
--------------------------------------------------------------------------------
--------------------------------------------------------------------------------
--------------------------------------------------------------------------------
--------------------------------------------------------------------------------
--------------------------------------------------------------------------------
--------------------------------------------------------------------------------
--------------------------------------------------------------------------------
--------------------------------------------------------------------------------
--------------------------------------------------------------------------------
--------------------------------------------------------------------------------
--------------------------------------------------------------------------------
--------------------------------------------------------------------------------
--------------------------------------------------------------------------------
--------------------------------------------------------------------------------
-----------------------------------------------------------------------TCAGACCTG
GAGGAGGAGATATGAAGGACAATTGGAGAAGTGAATTATATAAATATAAAGTAGTAAAAATTGAACCATTAGGAGTAGCA
CCCACCAAGGCAAAGAGAAGAGTGGTGCAGAGAGAAAAAAGAGCAGTAGGAATAGGAGCTTTGTTCCTTAGGTTCTTAGG
AGCAGCAGGAAGCACTATGGGCGCAGCGTCAATGACGCTGACGGTACAGGCCAGACAATTATTGTCTGGTATAGTGCAGC
AGCAGAACAATTTGCTGAGGGCTATTGAGGCGCAACAGCATCTGTTGCAACTCACAGTCTGGGGCATCAAGCAGCTCCAG
GCAAGAATCCTGGCTGTAGAAAGATACCTAAAGGATCAACAGCTCCTGGGGATTTGGGGTTGCTCTAGAAAACTCATTTG
CACCACTGCTGTGCCTTGCAATGCTAGTTGGAGTAATAAATCTCTGGAACAGATTTGGAATCACACGACCTGGATGGAGT
AGGACAGAGAAATTAACAATTACACAAGCTTAATACACTCCTTAATTGAAGAATCGCAAAACCAGCAAGAAAAGAATGAA
CAAGAATTATTAGAATTAGATAAATGGGCAAGTTTGTGGAATTAGTTTAACATAACAAATTGGCTGTGGTATATAAAATT
ATTCATAATGATAGTAGGAGGCTTGGTAGGTTTAAGAATAGTTTTTGCTGTACTTTCTGTAGTGAATAGAGTTAGGCAGG
GATATTCACCATTATCGTTTCAGACCCACCTCCCAATCCCGAGGGGACCCGACAGGCCCGAAGGAATAGAAGAAGAAGGT
GGAGAGAGAGACAGAGACAGATCCATTCGATTAGTGAACGGATCCTTAGCACTTATCTAGGACGATCTGCGGAGCCTGTG
CCTCTTCAGCTACCACCGCTTGAGAGACTTACTCTTGATTGTAACGAGGATTGTAGAACTTCTGGGACGCAGAGGGTGGG
AAGCCCTCAAATATTGGTAGAATCTCCTACAATATTGGAGTCAGGAGCTAAAGAATAGTGCTGTTAGCTTGCTCAATGCC
ACAGCTATAGCAGTAGCTGAGAGGACAGATAAGGTTATAGAAGTAGTACAAGAAGCTTATAGAGCTATTCGCCACATACC
TAGAAGAATAAGACAGGGCTTGGAAAGGATTTTGCTATAAGATAGGTGGCAAGTGGTCAAAAAGTAGTGTGGTTGGATGG
CCTGCTGTAAGGGAAAGAATGAGACGAGCTGAGCCAGCAGCAGATGGGGTGGGAGCAGCATCTCGAGACCTAGAAAAACA
TGGAGCAATCACAAGTAGCAACACAGCAGCTAACAATGCTGCTTGTGCCTGGCTAGAAGCACAAGAGGAGGAGAAGGTAG
GTTTTCCAGTCACACCTCAGGTACCTTTAAGACCAATGACTTACAAGGCAGCTGTAGATCTTAGCCACTTTTTAAAAGAA
AAGGGGGGACTGGAAGGGCTAATTCACTCCCAACGAAGACAAGATATCCTTGATCTGTGGATCTACCACACACAAGGCTA
CTTCCCTGATTGGCAGAACTACACACCAGGACCAGGGATCAGATATCCACTGACCTTTGGATGGCGCTACAAGCTAGTAC
CAGTTGAGCCAGAGAAGTTAGAAGAAGCCAACAAAGGAGAGAACACCAGCTTGTTACACCCTGTGAGCCTGCATGGAATG
GATGACCCGGAGAGAGAAGTGTTAGAGTGGAGGTTTGACAGCCGCCTAGCATTTCATCACGTGGCCCGAGAGCTGCATCC
GGAGTACTTCAAGAACTGCTGATATCGAGCTTGCTACAAAGGACTTTCCGCTGGGGACTTTCCAAGGAGGCGTGGCCTGG
GCGGGACTGAGGAGTGGCGAGCCCTCAGATCCTGCATATAAGCAGCTGCTTTTTGCCTGTACTGGGTCTCTCTGGTTAGA
CCAGATCTGAGCCTGGGAGCTCTCTGGCTAACTAGGGAACCCACTGCTTAAGCCTCAATAAAGCTTGCCTTGAGTGCTTC
AAGTAGTGTGTGCCCATCTGTTGTGTGACTCTGGTAACTAGAGATCCCTCA
>3-16envLTR
--------------------------------------------------------------------------------
--------------------------------------------------------------------------------
--------------------------------------------------------------------------------
--------------------------------------------------------------------------------
--------------------------------------------------------------------------------
--------------------------------------------------------------------------------
--------------------------------------------------------------------------------
--------------------------------------------------------------------------------
--------------------------------------------------------------------------------
--------------------------------------------------------------------------------
```

```
--------------------------------------------------------------------------------
--------------------------------------------------------------------------------
--------------------------------------------------------------------------------
--------------------------------------------------------------------------------
--------------------------------------------------------------------------------
--------------------------------------------------------------------------------
--------------------------------------------------------------------------------
--------------------------------------------------------------------------------
--------------------------------------------------------------------------------
--------------------------------------------------------------------------------
--------------------------------------------------------------------------------
--------------------------------------------------------------------------------
--------------------------------------------------------------------------------
--------------------------------------------------------------------------------
--------------------------------------------------------------------------------
--------------------------------------------------------------------------------
--------------------------------------------------------------------------------
--------------------------------------------------------------------------------
--------------------------------------------------------------------------------
--------------------------------------------------------------------------------
--------------------------------------------------------------------------------
--------------------------------------------------------------------------------
--------------------------------------------------------------------------------
--------------------------------------------------------------------------------
--------------------------------------------------------------------------------
--------------------------------------------------------------------------------
--------------------------------------------------------------------------------
--------------------------------------------------------------------------------
--------------------------------------------------------------------------------
--------------------------------------------------------------------------------
--------------------------------------------------------------------------------
--------------------------------------------------------------------------------
--------------------------------------------------------------------------------
--------------------------------------------------------------------------------
--------------------------------------------------------------------------------
--------------------------------------------------------------------------------
--------------------------------------------------------------------------------
--------------------------------------------------------------------------------
--------------------------------------------------------------------------------
--------------------------------------------------------------------------------
--------------------------------------------------------------------------------
--------------------------------------------------------------------------------
--------------------------------------------------------------------------------
--------------------------------------------------------------------------------
--------------------------------------------------------------------------------
--------------------------------------------------------------------------------
--------------------------------------------------------------------------------
--------------------------------------------------------------------------------
--------------------------------------------------------------------------------
--------------------------------------------------------------------------------
--------------------------------------------------------------------------------
--------------------------------------------------------------------------------
--------------------------------------------------------------------------------
--------------------------------------------------------------------------------
--------------------------------------------------------------------------------
--------------------------------------------------------------------------------
--------------------------------------------------------------------------------
--------------------------------------------------------------------------------
--------------------------------------------------------------------------------
--------------------------------------------------------------------------------
--------------------------------------------------------------------------------
--------------------------------------------------------------------------------
--------------------------------------------------------------------------------
--------------------------------------------------------------------------------
--------------------------------------------------------------------------------
--------------------------------------------------------------------------------
--------------------------------------------------------------------------------
--------------------------------------------------------------------------------
--------------------------------------------------------------------------------
--------------------------------------------------------------------------------
--------------------------------------------------------------------------------
--------------------------------------------------------------------------------
--------------------------------------------------------------------------------
--------------------------------------------------------------------------------
--------------------------------------------------------------------------------
--------------------------------------------------------------------------------
--------------------------------------------------------------------------------
--------------------------------------------------------------------------------
--------------------------------------------------------------------------------
--------------------------------------------------------------------------------
--------------------------------------------------------------------------------
--------------------------------------------------------------------------------
--------------------------------------------------------------------------------
--------------------------------------------------------------------------------
--------------------------------------------------------------------------------
------------------ACAATTGGAGAAGTGAATTATATAAATATAAAGTAGTAAAAATTGAACCATTAGGAGTAGCA
CCCACCAAGGCAAAGAGAAGAGTGGTGCAGAGAGAAAAAAGAGCAGTAGGAATAGGAGCTTTGTTCCTTAGGTTCTTAGG
AGCAGCAGGAAGCACTATGGGCGCAGCGTCAATGACGCTGACGGTACAGGCCAGACAATTATTGTCTGGTATAGTGCAGC
AGCAGAACAATTTGCTGAGGGCTATTGAGGCGCAACAGCATCTGTTGCAACTCACAGTCTGGGGCATCAAGCAGCTCCAG
GCAAGAATCCTGGCTGTGGAAAGATACCTAAAGGATCAACAGCTCCTGGGGATTTGGGGTTGCTCTGGAAAACTCATTTG
CACCACTGCTGTGCCTTGGAATGCTAGTTGGAGTAATAAATCTCTAGAACAGATTTGGAATCACACGACCTGGATGGAGT
GGGACAGAGAAATTAACAATTACACAAGCTTAATACACTCCTTAATTGAAGAATCGCAAAACCAGCAAGAAAAGAATGAA
CAAGAATTATTGGAATTAGATAAATGGGCAAGTTTGTGGAATTGGTTTAACATAACAAATTGGCTGTGGTATATAAAATT
ATTCATAATGATAGTAGGAGGCTTGGTAGGTTTAAGAATAGTTTTTGCTGTACTTTCTGTAGTGAATAGAGTTAGGCAGG
```

```
GATATTCACCATTATCGTTTCAGACCCACCTCCCAATCCCGAGGGGACCCGACAGGCCCGAAGGAATAGAAGAAGAAGGT
GGAGAGAGAGACAGAGACAGATCCATTCGATTAGTGAACGGATCCTTAGCACTTATCTGGGACGATCTGCGGAGCCTGTG
CCTCTTCAGCTACCACCGCTTGAGAGACTTACTCTTGATTGTAACGAGGATTGTGGAACTTCTAGGACGCAGGAGGTAGG
AAGCCCTCAAATATTGGTGGAATCTCCTACAATATTGGAGTCAGGAGCTAAAGAATAGTGCTGTTAGCTTGCTCAATGCC
ACAGCTATAGCAGTAGCTGAGGGGACAGATAGGGTTATAGAAGTAGTACAAGAAGCTTATAGAGCTATTCGCCACATACC
TAGAAGAATAAGACAGGGCTTAGAAAGGATTTTGCTATAAGATGGGTGGCAAGTGGTCAAAAAGTAGTGTGGTTGGATGG
CCTGCTGTAAGGGAAAGAATGAGACGAGCTGAGCCAGCAGCAGATGGGGTGGGAGCAGCATCTCGAGACCTAGAAAAACA
TGGAGCAATCACAAGTAGCAACACAGCAGCTAACAATGCTGCTTGTGCCTGGCTAGAAGCACAAGAGGAGGAGAAGGTAG
GTTTTCCAGTCACACCTCAGGTACCTTTAAGACCAATGACTTACAAGGCAGCTGTAGATCTTAGCCACTTTTTAAAAGAA
AAGGGGGGACTGGAAGGGCTAATTCACTCCCAACGAAGACAAGATATCCTTGATCTGTGGATCTACCACACACAAGGCTA
CTTCCCTGATTGGCAGAACTACACACCAGGACCAGGGATCAGATATCCACTGACCTTTGGATGGCGCTACAAGCTAGTAC
CAGTTGAGCCAGAGAAGTTAGAAGAAGCCAACAAAGGAGAGAACACCAGCTTGTTACACCCTGTGAGCCTGCATGGAATG
GATGACCCGGAGAGAGAAGTGTTAGAGTGGAGGTTTGACAGCCGCCTAGCATTTCATCACGTGGCCCGAGAGCTGCATCC
GGAGTACTTCAAGAACTGCTGATATCGAGCTTGCTACAAGGGACTTTCCGCTGGGGACTTTCCAGGGAGGCGTGGCCTGG
GCGGGACTGGGGAGTGGCGAGCCCTCAGATCCTGCATATAAGCAGCTGCTTTTTGCCTGTACTAGGTCTCTCTGGTTAGA
CCAGATCTGAGCCTGGGAGCTCTCTGGCTAACTAGGGAACCCACTGCTTAAGCCTCAATAAAGCTTGCCTTGAGTGCTTC
AAGTAGTGTGTGCCCATCTGTTGTGTGACTCTGGTAACTAGAGATCCCTCA
>3-17envLTR
--------------------------------------------------------------------------------
--------------------------------------------------------------------------------
--------------------------------------------------------------------------------
--------------------------------------------------------------------------------
--------------------------------------------------------------------------------
--------------------------------------------------------------------------------
--------------------------------------------------------------------------------
--------------------------------------------------------------------------------
--------------------------------------------------------------------------------
--------------------------------------------------------------------------------
--------------------------------------------------------------------------------
--------------------------------------------------------------------------------
--------------------------------------------------------------------------------
--------------------------------------------------------------------------------
--------------------------------------------------------------------------------
--------------------------------------------------------------------------------
--------------------------------------------------------------------------------
--------------------------------------------------------------------------------
--------------------------------------------------------------------------------
--------------------------------------------------------------------------------
--------------------------------------------------------------------------------
--------------------------------------------------------------------------------
--------------------------------------------------------------------------------
--------------------------------------------------------------------------------
--------------------------------------------------------------------------------
--------------------------------------------------------------------------------
--------------------------------------------------------------------------------
--------------------------------------------------------------------------------
--------------------------------------------------------------------------------
--------------------------------------------------------------------------------
--------------------------------------------------------------------------------
--------------------------------------------------------------------------------
--------------------------------------------------------------------------------
--------------------------------------------------------------------------------
--------------------------------------------------------------------------------
--------------------------------------------------------------------------------
--------------------------------------------------------------------------------
--------------------------------------------------------------------------------
--------------------------------------------------------------------------------
--------------------------------------------------------------------------------
--------------------------------------------------------------------------------
--------------------------------------------------------------------------------
--------------------------------------------------------------------------------
--------------------------------------------------------------------------------
--------------------------------------------------------------------------------
--------------------------------------------------------------------------------
--------------------------------------------------------------------------------
--------------------------------------------------------------------------------
--------------------------------------------------------------------------------
--------------------------------------------------------------------------------
--------------------------------------------------------------------------------
--------------------------------------------------------------------------------
--------------------------------------------------------------------------------
--------------------------------------------------------------------------------
--------------------------------------------------------------------------------
--------------------------------------------------------------------------------
--------------------------------------------------------------------------------
--------------------------------------------------------------------------------
--------------------------------------------------------------------------------
--------------------------------------------------------------------------------
--------------------------------------------------------------------------------
--------------------------------------------------------------------------------
--------------------------------------------------------------------------------
--------------------------------------------------------------------------------
--------------------------------------------------------------------------------
--------------------------------------------------------------------------------
--------------------------------------------------------------------------------
--------------------------------------------------------------------------------
--------------------------------------------------------------------------------
--------------------------------------------------------------------------------
--------------------------------------------------------------------------------
--------------------------------------------------------------------------------
--------------------------------------------------------------------------------
--------------------------------------------------------------------------------
--------------------------------------------------------------------------------
--------------------------------------------------------------------------------
```


--------------------------GCANAGAGAAAAAAGAGCAGTGGGAATAGGAGCTTTGTTCCTTGGGTTCTTGGG
AGCAGCAGGAAGCACTATGGGCGCAGCGTCAATGACGCTGACGGTACAGGCCANACAATTATTGTCTGGTATAGTGCAGC
AGCAGAACAATTTGCTGAGGGCTATTGAGGCGCAACAGCATCTGTTGCAACTCACAGTCTGGGGCATCAAGCAGCTCCAG
GCAAGAATCCTGGCTGTGGAAAGATACCTAAAGGATCAACAGCTCCTGGGGATTTGGGGTTGCTCTGGAAAACTCATTTG
CACCACTGCTGTGCCTTGGAATGCTAGTTGGAGTAATAAATCTCTGGAACAGATTTGGAATCACACGACCTGGATGGAGT
GGGACAGAGAAATTAACAATTACACAAGCTTAATACACTCCTTAATTGAAGAATCGCAAAACCAGCAAGAAAAGAATGAA
CAAGAATTATTGGAATTAGATAAATGGGCAAGTTTGTGGAATTGGTTTAACATAACAAATTGGCTGTGGTATATAAAATT
ATTCATAATGATAGTAGGAGGCTTGGTAGGTTTAAGAATAGTTTTTGCTGTACTTTCTGTAGTGAATAGAGTTAGGCAGG
GATATTCACCATTATCGTTTCAGACCCACCTCCCAATCCCGAGGGGACCCGACAGGCCCGAAGGAATAGAAGAAGAAGGT
GGAGAGAGAGACAGAGACAGATCCATTCGATTAGTGAACGGATCCTTAGCACTTATCTGGGACGATCTGCGGAGCCTGTG
CCTCTTCAGCTACCACCGCTTGAGAGACTTACTCTTGATTGTAACGAGGATTGTGGAACTTCTGGGACGCAGGGGGTGGG
AAGCCCTCAAATATTGGTGGAATCTCCTACAATATTGGAGTCAGGAGCTAAAGAATAGTGCTGTTAGCTTGCTCAATGCC
ACAGCTATAGCAGTAGCTGAGGGGACAGATAGGGTTATAGAAGTAGTACAAGAAGCTTATNGAGCTATTCGCCACATACC
TAGAAGAATAAGACAGGGCTTGGAAAGGATTTTGCTATAAGATGGGTGGCAAGTGGTCAAAAAGTAGTGTGGTTGGATGG
CCTGCTGTAAGGGAAAGAATGAGACGAGCTGAGCCAGCAGCAGATGGGGTGGGAGCAGCATCTCGAGACCTAGAAAAACA
TGGAGCAATCACAAGTAGCAACACAGCAGCTAACAATGCTGCTTGTGCCTGGCTAGAAGCACAAGAGGAGGAGAAGGTGG
GTTTTCCAGTCACACCTCAGGTACCTTTAAGACCAATGACTTACAAGGCAGCTGTAGATCTTAGCCACTTTTTAAAAGAA
AAGGGGGGACTGGAAGGGCTAATTCACTCCCAACGAAGACAAGATATCCTTGATCTGTGGATCTACCACACACAAGGCTA
CTTCCCTGATTGGCAGAACTACACACCAGGACCAGGGATCAGATATCCACTGACCTTTGGATGGCGCTACAAGCTAGTAC
CAGTTGAGCCAGAGAAGTTAGAAGAAGCCAACAAAGGAGAGAACACCAGCTTGTTACACCCTGTGAGCCTGCATGGAATG
GATGACCCGGAGAGAGAAGTGTTAGAGTGGAGGTTTGACAGCCGCCTAGCATTTCATCACGTGGCCCGAGAGCTGCATCC
GGAGTACTTCAAGAACTGCTGATATCGAGCTTGCTACAAGGGACTTTCCGCTGGGGACTTTCCAGGGAGGCGTGGCCTGG
GCGGGACTGGGGAGTGGCGAGCCCTCAGATCCTGCATATAAGCAGCTGCTTTTTGCCTGTACTGGGTCTCTCTGGTTAGA
CCAGATCTGAGCCTGGGAGCTCTCTGGCTAACTAGGGAACCCACTGCTTAAGCCTCAATAAAGCTTGCCTTGAGTGCTTC
AAGTAGTGTGTGCCCATCTGTTGTGTGACTCTGGTAACTAGAGATCCCTCA

>3-18envLTR

--------------------------GCAGAGAGAAAAAAGAGCAGTGGGAATAGGAGCTTTGTTCCTTGGGTTCTTGGG
AGCAGCAGGAAGCACTATGGGCGCAGCGTCAATGACGCTGACGGTACAGGCCAGACAATTATTGTCTGGTATAGTGCAGC
AGCAGAACAATTTGCTGAGGGCTATTGAGGCGCAACAGCATCTGTTGCAACTCACAGTCTGGGGCATCAAGCAGCTCCAG
GCAAGAATCCTGGCTGTGGAAAGATACCTAAAGGATCAACAGCTCCTGGGGATTTGGGGTTGCTCTGGAAAACTCATTTG
CACCACTGCTGTGCCTTGGAATGCTAGTTGGAGTAATAAATCTCTGGAACAGATTTGGAATCACACGACCTGGATGGAGT
GGGACAGAGAAATTAACAATTACACAAGCTTAATACACTCCTTAATTGAAGAATCGCAAAACCAGCAAGAAAAGAATGAA
CAAGAATTATTGGAATTAGATAAATGGGCAAGTTTGTGGAATTGGTTTAACATAACAAATTGGCTGTGGTATATAAAATT
ATTCATAATGATAGTAGGAGGCTTGGTAGGTTTAAGAATAGTTTTTGCTGTACTTTCTGTAGTGAATAGAGTTAGGCAGG
GATATTCACCATTATCGTTTCAGACCCACCTCCCAATCCCGAGGGGACCCGACAGGCCCGAAGGAATAGAAGAAGAAGGT
GGAGAGAGAGACAGAGACAGATCCATTCGATTAGTGAACGGATCCTTAGCACTTATCTGGGACGATCTGCGGAGCCTGTG
CCTCTTCAGCTACCACCGCTTGAGAGACTTACTCTTGATTGTAACGAGGATTGTGGAACTTCTGGGACGCAGGGGGTGGG
AAGCCCTCAAATATTGGTGGAATCTCCTACAATATTGGAGTCAGGAGCTAAAGAATAGTGCTGTTAGCTTGCTCAATGCC
ACAGCTATAGCAGTAGCTGAGGGGACAGATAGGGTTATAGAAGTAGTACAAGAAGCTTATAGAGCTATTCGCCACATACC
TAGAAGAATAAGACAGGGCTTGGAAAGGATTTTGCTATAAGATGGGTGGCAAGTGGTCAAAAAGTAGTGTGGTTGGATGG
CCTGCTGTAAGGGAAAGAATGAGACGAGCTGAGCCAGCAGCAGATGGGGTGGGAGCAGCATCTCGAGACCTAGAAAAACA
TGGAGCAATCACAAGTAGCAACACAGCAGCTAACAATGCTGCTTGTGCCTGGCTAGAAGCACAAGAGGAGGAGAAGGTGG
GTTTTCCAGTCACACCTCAGGTACCTTTAAGACCAATGACTTACAAGGCAGCTGTAGATCTTAGCCACTTTTTAAAAGAA
AAGGGGGGACTGGAAGGGCTAATTCACTCCCAACGAAGACAAGATATCCTTGATCTGTGGATCTACCACACACAAGGCTA
CTTCCCTGATTGGCAGAACTACACACCAGGACCAGGGATCAGATATCCACTGACCTTTGGATGGCGCTACAAGCTAGTAC
CAGTTGAGCCAGAGAAGTTAGAAGAAGCCAACAAAGGAGAGAACACCAGCTTGTTACACCCTGTGAGCCTGCATGGAATG
GATGACCCGGAGAGAGAAGTGTTAGAGTGGAGGTTTGACAGCCGCCTAGCATTTCATCACGTGGCCCGAGAGCTGCATCC
GGAGTACTTCAAGAACTGCTGATATCGAGCTTGCTACAAGGGACTTTCCGCTGGGGACTTTCCAGGGAGGCGTGGCCTGG
GCGGGACTGGGGAGTGGCGAGCCCTCAGATCCTGCATATAAGCAGCTGCTTTTTGCCTGTACTGGGTCTCTCTGGTTAGA
CCAGATCTGAGCCTGGGAGCTCTCTGGCTAACTAGGGAACCCACTGCTTAAGCCTCAATAAAGCTTGCCTTGAGTGCTTC
AAGTAGTGTGTGCCCATCTGTTGTGTGACTCTGGTAACTAGAGATCCCTCA
>3-22envLTR


```
--------------------------------------------------------------------------------
--------------------------------------------------------------------------------
--------------------------------------------------------------------------------
--------------------------------------------------------------------------------
--------------------------------------------------------------------------------
--------------------------------------------------------------------------------
--------------------------------------------------------------------------------
--------------------------------------------------------------------------------
--------------------------------------------------------------------------------
--------------------------------------------------------------------------------
--------------------------------------------------------------------------------
--------------------------------------------------------------------------------
--------------------------------------------------------------------------------
--------------------------------------------------------------------------------
--------------------------------------------------------------------------------
--------------------------------------------------------------------------------
--------------------------------------------------------------------------------
--------------------------------------------------------------------------------
--------------------------------------------------------------------------------
--------------------------------------------------------------------------------
--------------------------------------------------------------------------------
--------------------------------------------------------------------------------
--------------------------------------------------------------------------------
--------------------------------------------------------------------------------
--------------------------------------------------------------------------------
--------------------------------------------------------------------------------
--------------------------------------------------------------------------------
--------------------------------------------------------------------------------
--------------------------------------------------------------------------------
--------------------------------------------------------------------------------
--------------------------------------------------------------------------------
--------------------------------------------------------------------------------
--------------------------------------------------------------------------------
--------------------------------------------------------------------------------
--------------------------------------------------------------------------------
--------------------------------------------------------------------------------
--------------------------------------------------------------------------------
--------------------------------------------------------------------------------
--------------------------------------------------------------------------------
--------------------------------------------------------------------------------
--------------------------------------------------------------------------------
--------------------------------------------------------------------------------
--------------------------------------------------------------------------------
--------------------------------------------------------------------------------
--------------------------------------------------------------------------------
--------------------------------------------------------------------------------
--------------------------------------------------------------------------------
--------------------------------------------------------------------------------
--------------------------------------------------------------------------------
--------------------------------------------------------------------------------
--------------------------------------------------------------------------------
--------------------------------------------------------------------------------
--------------------------------------------------------------------------------
--------------------------------------------------------------------------------
--------------------------------------------------------------------------------
--------------------------------------------------------------------------------
--------------------------------------------------------------------------------
--------------------------------------------------------------------------------
--------------------------------------------------------------------------------
--------------------------------------------------------------------------------
--------------------------------------------------------------------------------
--------------------------------------------------------------------------------
--------------------------------------------------------------------------------
--------------------------------------------------------------------------------
--------------------------------------------------------------------------------
--------------------------------------------------------------------------------
--------------------------------------------------------------------------------
--------------------------------------------------------------------------------
--------------------------------------------------------------------------------
--------------------------------------------------------------------------------
--------------------------------------------------------------------------------
--------------------------------------------------------------------------------
--------------------------------------------------------------------------------
--------------------------------------------------------------------------------
-------------------------CTATTAACAAGAGATGGTGGTAATAACAACAATGGGTCCGAGATCTTCAGACCTG
GAGGAGGAGATNTGAGGGACAATTGGAGAAGTGAATTATATAAATATAAAGTAGTAAAAATTGAACCATTGGGAGTAGCA
CCCACCAAGGCAAAGAGAAGAGTGGTGCAGAGAGAAAAAAGAGCAGTGGGAATAGGAGCTTTGTTCCTTGGGTTCTTGGG
AGCAGCAGGAAGCACTATGGGCGCAGCGTCAATGACGCTGACGGTACAGGCCAGACAATTATTGTCTGGTATAGTGCAGC
AGCAGAACAATTTGCTGAGGGCTATTGAGGCGCAACAGCATCTGTTGCAACTCACAGTCTGGGGCATCAAGCAGCTCCAG
GCAAGAATCCTGGCTGTGGAAAGATACCTAAAGGATCAACAGCTCCTGGGGATTTGGGGTTGCTCTGGAAAACTCATTTG
CACCACTGCTGTGCCTTGGAATGCTAGTTGGAGTAATAAATCTCTGGAACAGATTTGGAATCACACGACCTGGATGGAGT
GGGACAGAGAAATTAACAATTACACAAGCTTAATACACTCCTTAATTGAAGAATCGCAAAACCAGCAAGAAAAGAATGAA
CAAGAATTATTGGAATTAGATAAATGGGCAAGTTTGTGGAATTGGTTTAACATAACAAATTGGCTGTGGTATATAAAATT
ATTCATAATGATAGTAGGAGGCTTGGTAGGTTTAAGAATAGTTTTTGCTGTACTTTCTGTAGTGAATAGAGTTAGGCAGG
GATATTCACCATTATCGTTTCAGACCCACCTCCCAATCCCGAGGGGACCCGACAGGCCCGAAGGAATAGAAGAAGAAGGT
GGAGAGAGAGACAGAGACAGATCCATTCGATTAGTGAACGGATCCTTAGCACTTATCTGGGACGATCTGCGGAGCCTGTG
CCTCTTCAGCTACCACCGCTTGAGAGACTTACTCTTGATTGTAACGAGGATTGTGGAACTTCTGGGACGCAGGGGGTGGG
AAGCCCTCAAATATTGGTGGAATCTCCTACAATATTGGAGTCAGGAGCTAAAGAATAGTGCTGTTAGCTTGCTCAATGCC
ACAGCTATAGCAGTAGCTGAGGGGACAGATAGGGTTATAGAAGTAGTACAAGAAGCTTATAGAGCTATTCGCCACATACC
TAGAAGAATAAGACAGGGCTTGGAAAGGATTTTGCTATAAGATGGGTGGCAAGTGGTCAAAAAGTAGTGTGGTTGGATGG
CCTGCTGTAAGGGAAAGAATGAGACGAGCTGAGCCAGCAGCAGATGGGGTGGGAGCAGCATCTCGAGACCTAGAAAAACA
TGGAGCAATCACAAGTAGCAACACAGCAGCTAACAATGCTGCTTGTGCCTGGCTAGAAGCACAAGAGGAGGAGAAGGTGG
GTTTTCCAGTCACACCTCAGGTACCTTTAAGACCAATGACTTACAAGGCAGCTGTAGATCTTAGCCACTTTTTAAAAGAA
AAGGGGGGACTGGAAGGGCTAATTCACTCCCAACGAAGACAAGATATCCTTGATCTGTGGATCTACCACACACAAGGCTA
```

CTTCCCTGATTGGCAGAACTACACACCAGGACCAGGGATCAGATATCCACTGACCTTTGGATGGCGCTACAAGCTAGTAC
CAGTTGAGCCAGAGAAGTTAGAAGAAGCCAACAAAGGAGAGAACACCAGCTTGTTACACCCTGTGAGCCTGCATGGAATG
GATGACCCGGAGAGAGAAGTGTTAGAGTGGAGGTTTGACAGCCGCCTAGCATTTCATCACGTGGCCCGAGAGCTGCATCC
GGAGTACTTCAAGAACTGCTGATATCGAGCTTGCTACAAGGGACTTTCCGCTGGGGACTTTCCAGGGAGGCGTGGCCTGG
GCGGGACTGGGGAGTGGCGAGCCCTCAGATCCTGCATATAAGCAGCTGCTTTTTGCCTGTACTGGGTCTCTCTGGTTAGA
CCAGATCTGAGCCTGGGAGCTCTCTGGCTAACTAGGGAACCCACTGCTTAAGCCTCAATAAAGCTTGCCTTGAGTGCTTC
AAGTAGTGTGTGCCCATCTGTTGTGTGACTCTGGTAACTAGAGATCCCTCA

>3-23envLTR

```
--------------------------------------------------------------------------------
--------------------------------------------------------------------------------
--------------------------------------------------------------------------------
--------------------------------------------------------------------------------
--------------------------------------------------------------------------------
--------------------------------------------------------------------------------
--------------------------------------------------------------------------------
--------------------------------------------------------------------------------
--------------------------------------------------------------------------------
--------------------------------------------------------------------------------
--------------------------------------------------------------------------------
--------------------------------------------------------------------------------
--------------------------------------------------------------------------------
--------------------------------------------------------------------------------
--------------------------------------------------------------------------------
--------------------------------------------------------------------------------
--------------------------------------------------------------------------------
--------------------------------------------------------------------------------
--------------------------------------------------------------------------------
--------------------------------------------------------------------------------
--------------------------------------------------------------------------------
--------------------------------------------------------------------------------
--------------------------------------------------------------------------------
--------------------------------------------------------------------------------
--------------------------------------------------------------------------------
--------------------------------------------------------------------------------
--------------------------------------------------------------------------------
--------------------------------------------------------------------------------
--------------------------------------------------------------------------------
--------------------------------------------------------------------------------
--------------------------------------------------------------------------------
--------------------------------------------------------------------------------
--------------------------------------------------------------------------------
--------------------------------------------------------------------------------
--------------------------------------------------------------------------------
--------------------------------------------------------------------------------
--------------------------------------------------------------------------------
--------------------------------------------------------------------------------
--------------------------------------------------------------------------------
--------------------------------------------------------------------------------
--------------------------------------------------------------------------------
--------------------------------------------------------------------------------
--------------------------------------------------------------------------------
--------------------------------------------------------------------------------
--------------------------------------------------------------------------------
--------------------------------------------------------------------------------
--------------------------------------------------------------------------------
--------------------------------------------------------------------------------
--------------------------------------------------------------------------------
--------------------------------------------------------------------------------
--------------------------------------------------------------------------------
--------------------------------------------------------------------------------
--------------------------------------------------------------------------------
--------------------------------------------------------------------------------
--------------------------------------------------------------------------------
--------------------------------------------------------------------------------
--------------------------------------------------------------------------------
--------------------------------------------------------------------------------
--------------------------------------------------------------------------------
--------------------------------------------------------------------------------
--------------------------------------------------------------------------------
--------------------------------------------------------------------------------
--------------------------------------------------------------------------------
--------------------------------------------------------------------------------
--------------------------------------------------------------------------------
--------------------------------------------------------------------------------
--------------------------------------------------------------------------------
--------------------------------------------------------------------------------
--------------------------------------------------------------------------------
--------------------------------------------------------------------------------
--------------------------------------------------------------------------------
--------------------------------------------------------------------------------
--------------------------------------------------------------------------------
--------------------------------------------------------------------------------
--------------------------------------------------------------------------------
--------------------------------------------------------------------------------
--------------------------------------------------------------------------------
--------------------------------------------------------------------------------
--------------------------------------------------------------------------------
--------------------------------------------------------------------------------
--------------------------------------------------------------------------------
--------------------------------------------------------------------------------
--------------------------------------------------------------------------------
--------------------------------------------------------------------------------
--------------------------------------------------------------------------------
--------------------------------------------------------------------------------
```


-----------------------------TAACAAGAGATGGTGGTAATAACAACAATGGGTCCGAGATCTTCGGACCTG
GAGGAGGAGATATGAGGGACAATTGGAGAAGTGAATTATATAAATATAAAGTAGTAAAAATTGAACCATTAGGAGTAGCA
CCCACCAAGGCAAAGAGAAGAGTGGTGCAGAGAGAAAAAAGAGCAGTGGGAATAGGAGCTTTGTTCCTTGGGTTCTTGGG
AGCAGCAGGAAGCACTATGGGCGCAGCGTCAATGACGCTGACGGTACAGGCCAGACAATTATTGTCTGGTATAGTGCAGC
AGCAGAACAATTTGCTGAGGGCTATTGAGGCGCAACAGCATCTGTTGCAACTCACAGTCTGGGGCATCAAGCAGCTCCAG
GCAAGAATCCTGGCTGTGGAAAGATACCTAAAGGATCAACAGCTCCTGGGGATTTGGGGTTGCTCTGGAAAACTCATTTG
CACCACTGCTGTGCCTTGGAATGCTAGTTGGAGTAATAAATCTCTGGAACAGATTTGGAATCACACGACCTGGATGGAGT
GGGACAGAGAAATTAACAATTACACAAGCTTAATACACTCCTTAATTGAAGAATCGCAAAACCAGCAAGAAAAGAATGAA
CAAGAATTATTGGAATTAGATAAATGGGCAAGTTTGTGGAATTGGTTTAACATAACAAATTGGCTGTGGTATATAAAATT
ATTCATAATGATAGTAGGAGGCTTGGTAGGTTTAAGAATAGTTTTTGCTGTACTTTCTGTAGTGAATAGAGTTAGGCAGG
GATATTCACCATTATCGTTTCAGACCCACCTCCCAATCCCGAGGGGACCCGACAGGCCCGAAGGAATAGAAGAAGAAGGT
GGAGAGAGAGACAGAGACAGATCCATTCGATTAGTGAACGGATCCTTAGCACTTATCTGGGACGATCTGCGGAGCCTGTG
CCTCTTCAGCTACCACCGCTTGAGAGACTTACTCTTGATTGTAACGAGGATTGTGGAACTTCTGGGACGCAGGGGGTGGG
AAGCCCTCAAATATTGGTGGAATCTCCTACAATATTGGAGTCAGGAGCTAAAGAATAGTGCTGTTAGCTTGCTCAATGCC
ACAGCTATAGCAGTAGCTGAGGGGACAGATAGGGTTATAGAAGTAGTACAAGAAGCTTATAGAGCTATTCGCCACATACC
TAGAAGAATAAGACAGGGCTTGGAAAGGATTTTGCTATAAGATGGGTGGCAAGTGGTCAAAAAGTAGTGTGGTTGGATGG
CCTGCTGTAAGGGAAAGAATGAGACGAGCTGAGCCAGCAGCAGATGGGGTGGGAGCAGCATCTCGAGACCTAGAAAAACA
TGGAGCAATCACAAGTAGCAACACAGCAGCTAACAATGCTGCTTGTGCCTGGCTAGAAGCACAAGAGGAGGAGAAGGTGG
GTTTTCCAGTCACACCTCAGGTACCTTTAAGACCAATGACTTACAAGGCAGCTGTAGATCTTAGCCACTTTTTAAAAGAA
AAGGGGGGACTGGAAGGGCTAATTCACTCCCAACGAAGACAAGATATCCTTGATCTGTGGATCTACCACACACAAGGCTA
CTTCCCTGATTGGCAGAACTACACACCAGGACCAGGGATCAGATATCCACTGACCTTTGGATGGCGCTACAAGCTAGTAC
CAGTTGAGCCAGAGAAGTTAGAAGAAGCCAACAAAGGAGAGAACACCAGCTTGTTACACCCTGTGAGCCTGCATGGAATG
GATGACCCGGAGAGAGAAGTGTTAGAGTGGAGGTTTGACAGCCGCCTAGCATTTCATCACGTGGCCCGAGAGCTGCATCC
GGAGTACTTCAAGAACTGCTGATATCGAGCTTGCTACAAGGGACTTTCCGCTGGGGACTTTCCAGGGAGGCGTGGCCTGG
GCGGGACTGGGGAGTGGCGAGCCCTCAGATCCTGCATATAAGCAGCTGCTTTTTGCCTGTACTGGGTCTCTCTGGTTAGA
CCAGATCTGAGCCTGGGAGCTCTCTGGCTAACTAGGGAACCCACTGCTTAAGCCTCAATAAAGCTTGCCTTGAGTGCTTC
AAGTAGTGTGTGCCCATCTGTTGTGTGACTCTGGTAACTAGAGATCCCTCA

>3-26envLTR


```
--------------------------------------------------------------------------------
--------------------------------------------------------------------------------
--------------------------------------------------------------------------------
--------------------------------------------------------------------------------
--------------------------------------------------------------------------------
--------------------------------------------------------------------------------
--------------------------------------------------------------------------------
--------------------------------------------------------------------------------
--------------------------------------------------------------------------------
--------------------------------------------------------------------------------
--------------------------------------------------------------------------------
--------------------------------------------------------------------------------
--------------------------------------------------------------------------------
--------------------------------------------------------------------------------
--------------------------------------------------------------------------------
--------------------------------------------------------------------------------
--------------------------------------------------------------------------------
--------------------------------------------------------------------------------
--------------------------------------------------------------------------------
--------------------------------------------------------------------------------
--------------------------------------------------------------------------------
--------------------------------------------------------------------------------
--------------------------------------------------------------------------------
--------------------------------------------------------------------------------
--------------------------------------------------------------------------------
--------------------------------------------------------------------------------
--------------------------------------------------------------------------------
--------------------------------------------------------------------------------
--------------------------------------------------------------------------------
--------------------------------------------------------------------------------
--------------------------------------------------------------------------------
--------------------------------------------------------------------------------
--------------------------------------------------------------------------------
--------------------------------------------------------------------------------
--------------------------------------------------------------------------------
--------------------------------------------------------------------------------
-------------------------CTATTAACAAGAGATGGTGGTAATAACAACAATGGGTCCGAGATCTTCAGACCTG
GAGGAGGAGATATGAGGGACAATTGGAGAAGTGAATTATATAAATATAAAGTAGTAAAAATTGAACCATTAGGAGTAGCA
CCCACCAAGGCAAAGAGAAGAGTGGTGCAGAGAGAAAAAAGAGCAGTAGGAATAGGAGCTTTGTTCCTTAGGTTCTTAGG
AGCAGCAGGAAGCACTATGGGCGCAGCGTCAATGACGCTGACGGTACAGGCCAGACAATTATTGTCTGGTATAGTGCAGC
AGCAGAACAATTTGCTGAGGGCTATTGAGGCGCAACAGCATCTGTTGCAACTCACAGTCTGGGGCATCAAGCAGCTCCAG
GCAAGAATCCTGGCTGTGGAAAGATACCTAAAGGATCAACAGCTCCTGGGGATTTGGGGTTGCTCTGGAAAACTCATTTG
CACCACTGCTGTGCCTTGGAATGCTAGTTGGAGTAATAAATCTCTAGAACAGATTTGGAATCACACGACCTGGATGGAGT
AGGACAGAGAAATTAACAATTACACAAGCTTAATACACTCCTTAATTGAAGAATCGCAAAACCAGCAAGAAAAGAATGAA
CAAGAATTATTGGAATTAGATAAATGGGCAAGTTTGTGGAATTGGTTTAACATAACAAATTGGCTGTGGTATATAAAATT
ATTCATAATGATAGTAGGAGGCTTGGTAGGTTTAAGAATAGTTTTTGCTGTACTTTCTGTAGTGAATAGAGTTAGGCAGG
GATATTCACCATTATCGTTTCAGACCCACCTCCCAATCCCGAGGGGACCCGACAGGCCCGAAGGAATAGAAGAAGAAGGT
GGAGAGAGAGACAGAGACAGATCCATTCGATTAGTGAACGGATCCTTAGCACTTATCTAGGACGATCTGCGGAGCCTGTG
CCTCTTCAGCTACCACCGCTTGAGAGACTTACTCTTGATTGTAACGAGGATTGTGGAACTTCTAGGACGCAGGGGGTGGG
AAGCCCTCAAATATTGGTGGAATCTCCTACAATATTGGAGTCAGGAGCTAAAGAATAGTGCTGTTAGCTTGCTCAATGCC
ACAGCTATAGCAGTAGCTGAGAGGACAGATAGGGTTATAGAAGTAGTACAAGAAGCTTATAGAGCTATTCGCCACATACC
TAGAAGAATAAGACAGGGCTTGGAAAGGATTTTGCTATAAGATAGGTGGCAAGTGGTCAAAAAGTAGTGTGGTTAGATGG
CCTGCTGTAAGGGAAAGAATGAGACGAGCTGAGCCAGCAGCAGATGGGGTGGGAGCAGCATCTCGAGACCTAGAAAAACA
TGGAGCAATCACAAGTAGCAACACAGCAGCTAACAATGCTGCTTGTGCCTGGCTAGAAGCACAAGAGGAGGAGAAGGTAG
GTTTTCCAGTCACACCTCAGGTACCTTTAAGACCAATGACTTACAAGGCAGCTGTAGATCTTAGCCACTTTTTAAAAGAA
AAGGGGGGACTGGAAGGGCTAATTCACTCCCAACGAAGACAAGATATCCTTGATCTGTAGATCTACCACACACAAGGCTA
CTTCCCTGATTGGCAGAACTACACACCAGGACCAGGGATCAGATATCCACTGACCTTTGGATGGCGCTACAAGCTAGTAC
CAGTTGAGCCAGAGAAGTTAGAAGAAGCCAACAAAGGAGAGAACACCAGCTTGTTACACCCTGTGAGCCTGCATGGAATG
GATGACCCGGAGAGAGAAGTGTTAGAGTGGAGGTTTGACAGCCGCCTAGCATTTCATCACGTGGCCCGAGAGCTGCATCC
AGAGTACTTCAAGAACTGCTGATATCGAGCTTGCTACAAAGGACTTTCCGCTGAAGACTTTCCAAGGAGGCGTGGCCTGG
GCGGGACTAGGGAGTGGCGAGCCCTCAGATCCTGCATATAAGCAGCTGCTTTTTGCCTGTACTAGGTCTCTCTGGTTAGA
CCAGATCTGAGCCTAGGAGCTCTCTGGCTAACTAGGGAACCCACTGCTTAAGCCTCAATAAAGCTTGCCTTGAGTGCTTC
AAGTAGTGTGTGCCCATCTGTTGTGTGACTCTGGTAACTAGAGATCCCTCA
>3-31envLTR
--------------------------------------------------------------------------------
--------------------------------------------------------------------------------
--------------------------------------------------------------------------------
--------------------------------------------------------------------------------
--------------------------------------------------------------------------------
--------------------------------------------------------------------------------
--------------------------------------------------------------------------------
--------------------------------------------------------------------------------
--------------------------------------------------------------------------------
--------------------------------------------------------------------------------
--------------------------------------------------------------------------------
--------------------------------------------------------------------------------
--------------------------------------------------------------------------------
--------------------------------------------------------------------------------
--------------------------------------------------------------------------------
--------------------------------------------------------------------------------
--------------------------------------------------------------------------------
--------------------------------------------------------------------------------
--------------------------------------------------------------------------------
--------------------------------------------------------------------------------
--------------------------------------------------------------------------------
--------------------------------------------------------------------------------
--------------------------------------------------------------------------------
--------------------------------------------------------------------------------
--------------------------------------------------------------------------------
--------------------------------------------------------------------------------
--------------------------------------------------------------------------------
--------------------------------------------------------------------------------
--------------------------------------------------------------------------------
--------------------------------------------------------------------------------
```

```
--------------------------------------------------------------------------------
--------------------------------------------------------------------------------
--------------------------------------------------------------------------------
--------------------------------------------------------------------------------
--------------------------------------------------------------------------------
--------------------------------------------------------------------------------
--------------------------------------------------------------------------------
--------------------------------------------------------------------------------
--------------------------------------------------------------------------------
--------------------------------------------------------------------------------
--------------------------------------------------------------------------------
--------------------------------------------------------------------------------
--------------------------------------------------------------------------------
--------------------------------------------------------------------------------
--------------------------------------------------------------------------------
--------------------------------------------------------------------------------
--------------------------------------------------------------------------------
--------------------------------------------------------------------------------
--------------------------------------------------------------------------------
--------------------------------------------------------------------------------
--------------------------------------------------------------------------------
--------------------------------------------------------------------------------
--------------------------------------------------------------------------------
--------------------------------------------------------------------------------
--------------------------------------------------------------------------------
--------------------------------------------------------------------------------
--------------------------------------------------------------------------------
--------------------------------------------------------------------------------
--------------------------------------------------------------------------------
--------------------------------------------------------------------------------
--------------------------------------------------------------------------------
--------------------------------------------------------------------------------
--------------------------------------------------------------------------------
--------------------------------------------------------------------------------
--------------------------------------------------------------------------------
--------------------------------------------------------------------------------
--------------------------------------------------------------------------------
--------------------------------------------------------------------------------
--------------------------------------------------------------------------------
--------------------------------------------------------------------------------
--------------------------------------------------------------------------------
--------------------------------------------------------------------------------
--------------------------------------------------------------------------------
--------------------------------------------------------------------------------
--------------------------------------------------------------------------------
--------------------------------------------------------------------------------
--------------------------------------------------------------------------------
--------------------------------------------------------------------------------
--------------------------------------------------------------------------------
--------------------------------------------------------------------------------
--------------------------------------------------------------------------------
--------------------------------------------------------------------------------
--------------------------------------------------------------------------------
--------------------------------------------------------------------------------
--------------------------------------------------------------------------------
--------------------------------------------------------------------------------
--------------------------------------------------------------------------------
--------------------------------------------------------------------------------
--------------------------------------------------------------------------------
--------------------------------------------------------------------------------
--------------------------------------------------------------------------------
--------------------------------------------------------------------------------
--------------------------------------------------------------------------------
--------------------------------------------------------------------------------
-----------------------TGCTATTAACAAGAGATGGTGGTAATAACAACAATGGGTCCGAGATCTTCAGACCTG
GAGGAGGAGATATGAGGGACAATTGGAGAAGTGAATTATATAAATATAAAGTAGTAAAAATTGAACCATTAGGAGTAGCA
CCCACCAAGGCAAAGAGAAGAGTGGTGCAGAGAGAAAAAAGAGCAGTGGGAATAGGAGCTTTGTTCCTTGGGTTCTTGGG
AGCAGCAGGAAGCACTATGGGCGCAGCGTCAATGACGCTGACGGTACAGGCCAGACAATTATTGTCTGGTATAGTGCAGC
AGCAGAACAATTTGCTGAGGGCTATTGAGGCGCAACAGCATCTGTTGCAACTCACAGTCTGGGGCATCAAGCAGCTCCAG
GCAAGAATCCTGGCTGTGGAAAGATACCTAAAGGATCAACAGCTCCTGGGGATTTGGGGTTGCTCTGGAAAACTCATTTG
CACCACTGCTGTGCCTTGGAATGCTAGTTGGAGTAATAAATCTCTGGAACAGATTTGGAATCACACGACCTGGATGGAGT
GGGACAGAGAAATTAACAATTACACAAGCTTAATACACTCCTTAATTGAAGAATCGCAAAACCAGCAAGAAAAGAATGAA
CAAGAATTATTGGAATTAGATAAATGGGCAAGTTTGTGGAATTGGTTTAACATAACAAATTGGCTGTGGTATATAAAATT
ATTCATAATGATAGTAGGAGGCTTGGTAGGTTTAAGAATAGTTTTTGCTGTACTTTCTGTAGTGAATAGAGTTAGGCAGG
GATATTCACCATTATCGTTTCAGACCCACCTCCCAATCCCGAGGGGACCCGACAGGCCCGAAGGATAGAAGAAGAAGGT
GGAGAGAGAGACAGAGACAGATCCATTCGATTAGTGAACGGATCCTTAGCACTTATCTGGGACGATCTGCGGAGCCTGTG
CCTCTTCAGCTACCACCGCTTGAGAGACTTACTCTTGATTGTAACGAGGATTGTGGAACTTCTGGGACGCAGGGGGTGGG
AAGCCCTCAAATATTGGTGGAATCTCCTACAATATTGGAGTCAGGAGCTAAAGAATAGTGCTGTTAGCTTGCTCAATGCC
ACAGCTATAGCAGTAGCTGAGGGGACAGATAGGGTTATAGAAGTAGTACAAGAAGCTTATAGAGCTATTCGCCACATACC
TAGAAGAATAAGACAGGGCTTGGAAAGGATTTTGCTATAAGATGGGTGGCAAGTGGTCAAAAAGTAGTGTGGTTGGATGG
CCTGCTGTAAGGGAAAGAATGAGACGAGCTGAGCCAGCAGCAGATGGGGTGGGAGCAGCATCTCGAGACCTAGAAAAACA
TGGAGCAATCACAAGTAGCAACACAGCAGCTAACAATGCTGCTTGTGCCTGGCTAGAAGCACAAGAGGAGGAGAAGGTGG
GTTTTCCAGTCACACCTCAGGTACCTTTAAGACCAATGACTTACAAGGCAGCTGTAGATCTTAGCCACTTTTTAAAAGAA
AAGGGGGGACTGGAAGGGCTAATTCACTCCCAACGAAGACAAGATATCCTTGATCTGTGGATCTACCACACACAAGGCTA
CTTCCCTGATTGGCAGAACTACACACCAGGACCAGGGATCAGATATCCACTGACCTTTGGATGGCGCTACAAGCTAGTAC
CAGTTGAGCCAGAGAAGTTAGAAGAAGCCAACAAAGGAGAGAACACCAGCTTGTTACACCCTGTGAGCCTGCATGGAATG
GATGACCCGGAGAGAGAAGTGTTAGAGTGGAGGTTTGACAGCCGCCTAGCATTTCATCACGTGGCCCGAGAGCTGCATCC
GGAGTACTTCAAGAACTGCTGATATCGAGCTTGCTACAAGGGACTTTCCGCTGGGGACTTTCCAGGGAGGCGTGGCCTGG
GCGGGACTGGGGAGTGGCGAGCCCTCAGATCCTGCATATAAGCAGCTGCTTTTTGCCTGTACTGGGTCTCTCTGGTTAGA
CCAGATCTGAGCCTGGGAGCTCTCTGGCTAACTAGGGAACCCACTGCTTAAGCCTCAATAAAGCTTGCCTTGAGTGCTTC
AAGTAGTGTGTGCCCATCTGTTGTGTGACTCTGGTAACTAGAGATCCCTCA
>3-38envLTR
--------------------------------------------------------------------------------
--------------------------------------------------------------------------------
```

```
--------------------------------------------------------------------------------
--------------------------------------------------------------------------------
--------------------------------------------------------------------------------
--------------------------------------------------------------------------------
--------------------------------------------------------------------------------
--------------------------------------------------------------------------------
--------------------------------------------------------------------------------
--------------------------------------------------------------------------------
--------------------------------------------------------------------------------
--------------------------------------------------------------------------------
--------------------------------------------------------------------------------
--------------------------------------------------------------------------------
--------------------------------------------------------------------------------
--------------------------------------------------------------------------------
--------------------------------------------------------------------------------
--------------------------------------------------------------------------------
--------------------------------------------------------------------------------
--------------------------------------------------------------------------------
--------------------------------------------------------------------------------
--------------------------------------------------------------------------------
--------------------------------------------------------------------------------
--------------------------------------------------------------------------------
--------------------------------------------------------------------------------
--------------------------------------------------------------------------------
--------------------------------------------------------------------------------
--------------------------------------------------------------------------------
--------------------------------------------------------------------------------
--------------------------------------------------------------------------------
--------------------------------------------------------------------------------
--------------------------------------------------------------------------------
--------------------------------------------------------------------------------
--------------------------------------------------------------------------------
--------------------------------------------------------------------------------
--------------------------------------------------------------------------------
--------------------------------------------------------------------------------
--------------------------------------------------------------------------------
--------------------------------------------------------------------------------
--------------------------------------------------------------------------------
--------------------------------------------------------------------------------
--------------------------------------------------------------------------------
--------------------------------------------------------------------------------
--------------------------------------------------------------------------------
--------------------------------------------------------------------------------
--------------------------------------------------------------------------------
--------------------------------------------------------------------------------
--------------------------------------------------------------------------------
--------------------------------------------------------------------------------
--------------------------------------------------------------------------------
--------------------------------------------------------------------------------
--------------------------------------------------------------------------------
--------------------------------------------------------------------------------
--------------------------------------------------------------------------------
--------------------------------------------------------------------------------
--------------------------------------------------------------------------------
--------------------------------------------------------------------------------
--------------------------------------------------------------------------------
--------------------------------------------------------------------------------
--------------------------------------------------------------------------------
--------------------------------------------------------------------------------
--------------------------------------------------------------------------------
--------------------------------------------------------------------------------
--------------------------------------------------------------------------------
--------------------------------------------------------------------------------
--------------------------------------------------------------------------------
--------------------------------------------------------------------------------
--------------------------------------------------------------------------------
--------------------------------------------------------------------------------
--------------------------------------------------------------------------------
--------------------------------------------------------------------------------
--------------------------------------------------------------------------------
--------------------------------------------------------------------------------
--------------------------------------------------------------------------------
--------------------------------------------------------------------------------
--------------------------------------------------------------------------------
--------------------------------------------------------------------------------
--------------------------------------------------------------------------------
--------------------------------------------------------------------------------
--------------------------------------------------------------------------------
--------------------------------------------------------------------------------
--------------------------------------------------------------------------------
--------------------------------------------------------------------------------
--------------------------------------------------------------------------------
--------------------------------------------------------------------------------
--------------------------------------------------------------------------------
--------------------------------------------------------------------------------
--------------------------------------------------------------------------------
--------------------------------------------------------------------------------
--------------------------------------------------------------------------------
--------------------------------------------------------------------------------
--------------------------------------------------------------------------------
--------------------------------------------------------------------------------
--------------------------------------------------------------------------------
----------------------------------------------------AACAATGGGTCCGAGATCTTCAGACCTG
GAGGAGGAGATATGAAGGACAATTGGAGAAGTGAATTATATAAATATAAAGTAGTAAAAATTGAACCATTAGGAGTAGCA
```

CCCACCAAGGCAAAGAGAAGAGTGGTGCAGAGAGAAAAAAGAGCAGTAGGAATAGGAGCTTTGTTCCTTAGGTTCTTAGG
AGCAGCAGGAAGCACTATAGGCGCAGCGTCAATGACGCTGACGGTACAGGCCAGACAATTATTGTCTGGTATAGTGCAGC
AGCAGAACAATTTGCTGAGGGCTATTGAGGCGCAACAGCATCTGTTGCAACTCACAGTCTGGGGCATCAAGCAGCTCCAG
GCAAGAATCCTGGCTGTGGAAAGATACCTAAAGGATCAACAGCTCCTAGGGATTTGGGGTTGCTCTAGAAAACTCATTTG
CACCACTGCTGTGCCTTGGAATGCTAGTTGGAGTAATAAATCTCTGGAACAGATTTGGAATCACACGACCTGGATGGAGT
GGGACAGAGAAATTAACAATTACACAAGCTTAATACACTCCTTAATTGAAGAATCGCAAAACCAGCAAGAAAAGAATGAA
CAAGAATTATTAGAATTAGATAAATGGGCAAGTTTGTAGAATTGGTTTAACATAACAAATTGGCTGTGGTATATAAAATT
ATTCATAATGATAGTAGGAGGCTTGGTAAGTTTAAGAATAGTTTTTGCTGTACTTTCTGTAGTGAATAGAGTTAGGCAAG
GATATTCACCATTATCGTTTCAGACCCACCTCCCAATCCCGAGGGGACCCGACAGGCCCGAAGGAATAGAAGAAGAAGGT
GGAGAGAGAGACAGAGACAGATCCATTCGATTAGTGAACGGATCCTTAGCACTTATCTAGGACGATCTGCGGAGCCTGTG
CCTCTTCAGCTACCACCGCTTGAGAGACTTACTCTTGATTGTAACGAGGATTGTAGAACTTCTAGGACGCAGGGGGTGGG
AAGCCCTCAAATATTGGTAGAATCTCCTACAATATTGGAGTCAGGAGCTAAAGAATAGTGCTGTTAGCTTGCTCAATGCC
ACAGCTATAGCAGTAGCTGAAGGGACAGATAAGGTTATAGAAGTAGTACAAGAAGCTTATAGAGCTATTCGCCACATACC
TAGAAGAATAAGACAGGGCTTGGAAAGGATTTTGCTATAAGATAGGTGGCAAGTGGTCAAAAAGTAGTGTGGTTAGATGG
CCTGCTGTAAAAGAAAGAATGAGACGAGCTGAGCCAGCAGCAGATGGGGTGGGAGCAGCATCTCGAGACCTAGAAAAACA
TGGAGCAATCACAAGTAGCAACACAGCAGCTAACAATGCTGCTTGTGCCTAGCTAGAAGCACAAGAGGAGGAGAAGGTAG
GTTTTCCAGTCACACCTCAGGTACCTTTAAGACCAATGACTTACAAGGCAGCTGTAGATCTTAGCCACTTTTTAAAAGAA
AAGGGGGGACTGGAAGGGCTAATTCACTCCCAACGAAGACAAGATATCCTTGATCTGTGGATCTACCACACACAAGGCTA
CTTCCCTGATTGGCAGAACTACACACCAGGACCAGGGATCAGATATCCACTGACCTTTGGATGGCGCTACAAGCTAGTAC
CAGTTGAGCCAGAGAAGTTAGAAGAAGCCAACAAAGGAGAGAACACCAGCTTGTTACACCCTGTGAGCCTGCATGGAATG
GATGACCCGGAGAGAGAAGTGTTAGAGTGGAGGTTTGACAGCCGCCTAGCATTTCATCACGTGGCCCGAGAGCTGCATCC
GGAGTACTTCAAGAACTGCTGATATCGAGCTTGCTACAAAGGACTTTCCGCTAAGGACTTTCCAGGGAGGCGTGGCCTAG
GCAAGACTGAAGAGTGGCGAGCCCTCAGATCCTGCATATAAGCAGCTGCTTTTTGCCTGTACTAAGTCTCTCTGGTTAGA
CCAGATCTGAGCCTAGGAGCTCTCTGGCTAACTAGGGAACCCACTGCTTAAGCCTCAATAAAGCTTGCCTTGAGTGCTTC
AAGTAGTGTGTGCCCATCTGTTGTGTGACTCTGGTAACTAGAGATCCCTCA

>3-45envLTR


```
--------------------------------------------------------------------------------
--------------------------------------------------------------------------------
--------------------------------------------------------------------------------
--------------------------------------------------------------------------------
--------------------------------------------------------------------------------
--------------------------------------------------------------------------------
--------------------------------------------------------------------------------
--------------------------------------------------------------------------------
--------------------------------------------------------------------------------
--------------------------------------------------------------------------------
--------------------------------------------------------------------------------
--------------------------------------------------------------------------------
--------------------------------------------------------------------------------
--------------------------------------------------------------------------------
--------------------------------------------------------------------------------
--------------------------------------------------------------------------------
--------------------------------------------------------------------------------
--------------------------------------------------------------------------------
--------------------------------------------------------------------------------
--------------------------------------------------------------------------------
--------------------------------------------------------------------------------
--------------------------------------------------------------------------------
--------------------------------------------------------------------------------
--------------------------------------------------------------------------------
--------------------------------------------------------------------------------
--------------------------------------------------------------------------------
--------------------------------------------------------------------------------
--------------------------------------------------------------------------------
--------------GAGAAGAGTGGTGCAGAGAGAAAAAAGAGCAGTGGGAATAGGAGCTTTGTTCCTTGGGTTCTTGGG
AGCAGCAGGAAGCACTATGGGCGCAGCGTCAATGACGCTGACGGTACAGGCCAGACAATTATTGTCTGGTATAGTGCAGC
AGCAGAACAATTTGCTGAGGGCTATTGAGGCGCAACAGCATCTGTTGCAACTCACAGTCTGGGGCATCAAGCAGCTCCAG
GCAAGAATCCTGGCTGTGGAAAGATACCTAAAGGATCAACAGCTCCTGGGGATTTGGGGTTGCTCTGGAAAACTCATTTG
CACCACTGCTGTGCCTTGGAATGCTAGTTGGAGTAATAAATCTCTGGAACAGATTTGGAATCACACGACCTGGATGGAGT
GGGACAGAGAAATTAACAATTACACAAGCTTAATACACTCCTTAATTGAAGAATCGCAAAACCAGCAAGAAAAGAATGAA
CAAGAATTATTGGAATTAGATAAATGGGCAAGTTTGTGGAATTGGTTTAACATAACAAATTGGCTGTGGTATATAAAATT
ATTCATAATGATAGTAGGAGGCTTGGTAGGTTTAAGAATAGTTTTTGCTGTACTTTCTGTAGTGAATAGAGTTAGGCAGG
GATATTCACCATTATCGTTTCAGACCCACCTCCCAATCCCGAGGGGACCCGACAGGCCCGAAGGAATAGAAGAAGAAGGT
GGAGAGAGAGACAGAGACAGATCCATTCGATTAGTGAACGGATCCTTAGCACTTATCTGGGACGATCTGCGGAGCCTGTG
CCTCTTCAGCTACCACCGCTTGAGAGACTTACTCTTGATTGTAACGAGGATTGTGGAACTTCTGGGACGCAGGGGGTGGG
AAGCCCTCAAATATTGGTGGAATCTCCTACAATATTGGAGTCAGGAGCTAAAGAATAGTGCTGTTAGCTTGCTCAATGCC
ACAGCTATAGCAGTAGCTGAGGGGACAGATAGGGTTATAGAAGTAGTACAAGAAGCTTATAGAGCTATTCGCCACATACC
TAGAAGAATAAGACAGGGCTTGGAAAGGATTTTGCTATAAGATGGGTGGCAAGTGGTCAAAAAGTAGTGTGGTTGGATGG
CCTGCTGTAAGGGAAAGAATGAGACGAGCTGAGCCAGCAGCAGATGGGGTGGGAGCAGCATCTCGAGACCTAGAAAAACA
TGGAGCAATCACAAGTAGCAACACAGCAGCTAACAATGCTGCTTGTGCCTGGCTAGAAGCACAAGAGGAGGAGAAGGTGG
GTTTTCCAGTCACACCTCAGGTACCTTTAAGACCAATGACTTACAAGGCAGCTGTAGATCTTAGCCACTTTTTAAAAGAA
AAGGGGGGACTGGAAGGGCTAATTCACTCCCAACGAAGACAAGATATCCTTGATCTGTGGATCTACCACACACAAGGCTA
CTTCCCTGATTGGCAGAACTACACACCAGGACCAGGGATCAGATATCCACTGACCTTTGGATGGCGCTACAAGCTAGTAC
CAGTTGAGCCAGAGAAGTTAGAAGAAGCCAACAAAGGAGAGAACACCAGCTTGTTACACCCTGTGAGCCTGCATGGAATG
GATGACCCGGAGAGAGAAGTGTTAGAGTGGAGGTTTGACAGCCGCCTAGCATTTCATCACGTGGCCCGAGAGCTGCATCC
GGAGTACTTCAAGAACTGCTGATATCGAGCTTGCTACAAGGGACTTTCCGCTGGGGACTTTCCAGGGAGGCGTGGCCTGG
GCGGGACTGGGGAGTGGCGAGCCCTCAGATCCTGCATATAAGCAGCTGCTTTTTGCCTGTACTGGGTCTCTCTGGTTAGA
CCAGATCTGAGCCTGGGAGCTCTCTGGCTAACTAGGGAACCCACTGCTTAAGCCTCAATAAAGCTTGCCTTGAGTGCTTC
                AAGTAGTGTGTGCCCATCTGTTGTGTGACTCTGGTAACTAGAGATCCCTCA
                                      >3-65envLTR
--------------------------------------------------------------------------------
--------------------------------------------------------------------------------
--------------------------------------------------------------------------------
--------------------------------------------------------------------------------
--------------------------------------------------------------------------------
--------------------------------------------------------------------------------
--------------------------------------------------------------------------------
--------------------------------------------------------------------------------
--------------------------------------------------------------------------------
--------------------------------------------------------------------------------
--------------------------------------------------------------------------------
--------------------------------------------------------------------------------
--------------------------------------------------------------------------------
--------------------------------------------------------------------------------
--------------------------------------------------------------------------------
--------------------------------------------------------------------------------
--------------------------------------------------------------------------------
--------------------------------------------------------------------------------
--------------------------------------------------------------------------------
--------------------------------------------------------------------------------
--------------------------------------------------------------------------------
--------------------------------------------------------------------------------
--------------------------------------------------------------------------------
--------------------------------------------------------------------------------
--------------------------------------------------------------------------------
--------------------------------------------------------------------------------
--------------------------------------------------------------------------------
--------------------------------------------------------------------------------
--------------------------------------------------------------------------------
--------------------------------------------------------------------------------
--------------------------------------------------------------------------------
--------------------------------------------------------------------------------
--------------------------------------------------------------------------------
--------------------------------------------------------------------------------
--------------------------------------------------------------------------------
--------------------------------------------------------------------------------
--------------------------------------------------------------------------------
--------------------------------------------------------------------------------
--------------------------------------------------------------------------------
--------------------------------------------------------------------------------
```


-------TCAAATATTACAGGGCTGCTATTAACAAGAGATGGTGGTAATAACAACAATGGGCCCGAGATCTTCAGACCTG
GAGGAGGAGATATGAAGGACAATTAGAGAAGTGAATTATATAAATATAAAGTAGTAAAAATTGAACCATTAGGAGTAGCA
CCCACCAAGGCAAAGAGAAGAGTGGTGCAGAGAGAAAAAAGAGCAGTGGGAATAGGAGCTTTGTTCCTTGGGTTCTTAGG
AGCAGCAGGAAGCACTATGGGCGCAGCGTCAATGACGCTGACGGTACAGGCCAGACAATTATTGTCTGGTATAGTGCAGC
AGCAGAACAATTTGCTGAGGGCTATTGAGGCGCAACAGCATCTGTTGCAACTCACAGTCTGGGGCATCAAGCAGCTCCAG
GCAAGAATCCTGGCTGTGGAAAGATACCTAAAGGATCAACAGCTCCTGGGGATTTAAGGTTGCTCTGGAAAACTCATTTG
CACCACTGCTGTGCCTTAGAATGCTAGTTGGAGTAATAAATCTCTGGAACAGATTTAGAATCACACGACCTGGATGGAGT
GGGACAGAGAAATTAACAATTACACAAGCTTAATACACTCCTTAATTGAAGAATCGCAAAACCAGCAAGAAAAGAATGAA
CAAGAATTATTGGAATTAGATAAATGGGCAAGTTTGTGGAATTGGTTTAACATAACAAATTGGCTGTGGTATATAAAATT
ATTCATAATGATAGTAGGAGGCTTGGTAGGTTTAAGAATAGTTTTTGCTGTACTTTCTGTAGTGAATAAAGTTAGGCAAG
GATATTCACCATTATCGTTTCAGACCCACCTCCCAATCCCGAGGGGACCCGACAGGCCCGAAGGAATAGAAGAAGAAGGT
GGAGAGAGAGACAGAGACAGATCCATTCGATTAGTGAACGGATCCTTAGCACTTATCTGGGACGATCTGCGGAGCCTGTG
CCTCTTCAGCTACCACCGCTTGAGAGACTTACTCTTGATTGTAACGAGGATTGTGGAACTTCTAGGACGCAGGGGGTGGG
AAGCCCTCAAATATTGGTGGAATCTCCTACAATATTGGAGTCAGGAGCTAAAGAATAGTGCTGTTAGCTTGCTCAATGCC
ACAGCTATAGCAGTAGCTGAGGGGACAGATAAGGTTATAGAAGTAGTACAAGAAGCTTATAGAGCTATTCGCCACATACC
TAGAAGAATAAGACAGGGCTTAGAAAGGATTTTGCTATAAGATGGGTGGCAAGTGGTCAAAAAGTAGTGTGGTTAGATGG
CCTGCTGTAAGGGAAAGAATGAGACGAGCTGAGCCAGCAGCAGATGAGGTAGGAGCAGCATCTCGAGACCTAGAAAAACA
TGGAGCAATCACAAGTAGCAACACAGCAGCTAACAATGCTGCTTGTGCCTGGCTAGAAGCACAAGAGGAGGAGAAGGTAA
GTTTTCCAGTCACACCTCAGGTACCTTTAAGACCAATGACTTACAAGGCAGCTGTAGATCTTAGCCACTTTTTAAAAGAA
AAGGGGGGACTGGAAGGGCTAATTCACTCCCAACGAAGACAAGATATCCTTGATCTGTGGATCTACCACACACAAGGCTA
CTTCCCTGATTGGCAGAACTACACACCAGGACCAGGGATCAGATATCCACTGACCTTTGGATGGCGCTACAAGCTAGTAC
CAGTTGAGCCAGAGAAGTTAGAAGAAGCCAACAAAGGAGAGAACACCAGCTTGTTACACCCTGTGAGCCTGCATGGAATG
GATGACCCGGAGAGAGAAGTGTTAGAGTGGAGGTTTGACAGCCGCCTAGCATTTCATCACGTGGCCCGAGAGCTGCATCC
GGAGTACTTCAAGAACTGCTGATATCGAGCTTGCTACAAAGGACTTTCCGCTGGGGACTTTCCAGGGAGGCGTGGCCTAG
GCGGGACTGGGGAGTGGCGAGCCCTCAGATCCTGCATATAAGCAGCTGCTTTTTGCCTGTACTGGGTCTCTCTAGTTAGA
CCAGATCTGAGCCTGAGAGCTCTCTGGCTAACTAGGGAACCCACTGCTTAAGCCTCAATAAAGCTTGCCTTGAGTGCTTC
AAGTAGTGTGTGCCCATCTGTTGTGTGACTCTGGTAACTAGAGATCCCTCA

>3-66envLTR

-------------GAGGGACAATTGGAGAAGTGAATTATATAAATATAAAGTAGTAAAAATTGAACCATTAGGAGTAGCA
CCCACCAAGGCAAAGAGAAGAGTGGTGCAGAGAGAAAAAAGAGCAGTGGGAATAGGAGCTTTGTTCCTTGGGTTCTTGGG
AGCAGCAGGAAGCACTATGGGCGCAGCGTCAATGACGCTGACGGTACAGGCCAGACAATTATTGTCTGGTATAGTGCAGC
AGCAGAACAATTTGCTGAGGGCTATTGAGGCGCAACAGCATCTGTTGCAACTCACAGTCTGGGGCATCAAGCAGCTCCAG
GCAAGAATCCTGGCTGTGGAAAGATACCTAAAGGATCAACAGCTCCTGGGGATTTGGGGTTGCTCTGGAAAACTCATTTG
CACCACTGCTGTGCCTTGGAATGCTAGTTGGAGTAATAAATCTCTGGAACAGATTTGGAATCACACGACCTGGATGGAGT
GGGACAGAGAAATTAACAATTACACAAGCTTAATACACTCCTTAATTGAAGAATCGCAAAACCAGCAAGAAAAGAATGAA
CAAGAATTATTGGAATTAGATAAATGGGCAAGTTTGTGGAATTGGTTTAACATAACAAATTGGCTGTGGTATATAAAATT
ATTCATAATGATAGTAGGAGGCTTGGTAGGGTTAAGAATAGTTTTTGCTGTACTTTCTGTAGTGAATAGAGTTAGGCAGG
GATATTCACCATTATCGTTTCAGACCCACCTCCCAATCCCGAGGGGACCCGACAGGCCCGAAGGAATAGAAGAAGAAGGT
GGAGAGAGAGACAGAGACAGATCCATTCGATTAGTGAACGGATCCTTAGCACTTATCTGGGACGATCTGCGGAGCCTGTG

```
CCTCTTCAGCTACCACCGCTTGAGAGACTTACTCTTGATTGTAACGAGGATTGTGGAACTTCTGGGACGCAGGGGGTGGG
AAGCCCTCAAATATTGGTGGAATCTCCTACAATATTGGAGTCAGGAGCTAAAGAATAGTGCTGTTAGCTTGCTCAATGCC
ACAGCTATAGCAGTAGCTGAGGGGACAGATAGGGTTATAGAAGTAGTACAAGAAGCTTATAGAGCTATTCGCCACATACC
TAGAAGAATAAGACAGGGCTTGGAAAGGATTTTGCTATAAGATGGGTGGCAAGTGGTCAAAAAGTAGTGTGGTTGGATGG
CCTGCTGTAAGGGAAAGAATGAGACGAGCTGAGCCAGCAGCAGATGGGGTGGGAGCAGCATCTCGAGACCTAGAAAAACA
TGGAGCAATCACAAGTAGCAACACAGCAGCTAACAATGCTGCTTGTGCCTGGCTAGAAGCACAAGAGGAGGAGAAGGTGG
GTTTTCCAGTCACACCTCAGGTACCTTTAAGACCAATGACTTACAAGGCAGCTGTAGATCTTAGCCACTTTTTAAAAGAA
AAGGGGGGACTGGAAGGGCTAATTCACTCCCAACGAAGACAAGATATCCTTGATCTGTGGATCTACCACACACAAGGCTA
CTTCCCTGATTGGCAGAACTACACACCAGGACCAGGGATCAGATATCCACTGACCTTTGGATGGCGCTACAAGCTAGTAC
CAGTTGAGCCAGAGAAGTTAGAAGAAGCCAACAAAGGAGAGAACACCAGCTTGTTACACCCTGTGAGCCTGCATGGAATG
GATGACCCGGAGAGAGAAGTGTTAGAGTGGAGGTTTGACAGCCGCCTAGCATTTCATCACGTGGCCCGAGAGCTGCATCC
GGAGTACTTCAAGAACTGCTGATATCGAGCTTGCTACAAGGGACTTTCCGCTGGGGACTTTCCAGGGAGGCGTGGCCTGG
GCGGGACTGGGGAGTGGCGAGCCCTCAGATCCTGCATATAAGCAGCTGCTTTTTGCCTGTACTGGGTCTCTCTGGTTAGA
CCAGATCTGAGCCTGGGAGCTCTCTGGCTAACTAGGGAACCCACTGCTTAAGCCTCAATAAAGCTTGCCTTGAGTGCTTC
AAGTAGTGTGTGCCCATCTGTTGTGTGACTCTGGTAACTAGAGATCCCTCA
```

>3-71envLTR

```
--------------------------------------------------------------------------------
--------------------------------------------------------------------------------
--------------------------------------------------------------------------------
--------------------------------------------------------------------------------
--------------------------------------------------------------------------------
--------------------------------------------------------------------------------
--------------------------------------------------------------------------------
--------------------------------------------------------------------------------
--------------------------------------------------------------------------------
--------------------------------------------------------------------------------
--------------------------------------------------------------------------------
--------------------------------------------------------------------------------
--------------------------------------------------------------------------------
--------------------------------------------------------------------------------
--------------------------------------------------------------------------------
--------------------------------------------------------------------------------
--------------------------------------------------------------------------------
--------------------------------------------------------------------------------
--------------------------------------------------------------------------------
--------------------------------------------------------------------------------
--------------------------------------------------------------------------------
--------------------------------------------------------------------------------
--------------------------------------------------------------------------------
--------------------------------------------------------------------------------
--------------------------------------------------------------------------------
--------------------------------------------------------------------------------
--------------------------------------------------------------------------------
--------------------------------------------------------------------------------
--------------------------------------------------------------------------------
--------------------------------------------------------------------------------
--------------------------------------------------------------------------------
--------------------------------------------------------------------------------
--------------------------------------------------------------------------------
--------------------------------------------------------------------------------
--------------------------------------------------------------------------------
--------------------------------------------------------------------------------
--------------------------------------------------------------------------------
--------------------------------------------------------------------------------
--------------------------------------------------------------------------------
--------------------------------------------------------------------------------
--------------------------------------------------------------------------------
--------------------------------------------------------------------------------
--------------------------------------------------------------------------------
--------------------------------------------------------------------------------
--------------------------------------------------------------------------------
--------------------------------------------------------------------------------
--------------------------------------------------------------------------------
--------------------------------------------------------------------------------
--------------------------------------------------------------------------------
--------------------------------------------------------------------------------
--------------------------------------------------------------------------------
--------------------------------------------------------------------------------
--------------------------------------------------------------------------------
--------------------------------------------------------------------------------
--------------------------------------------------------------------------------
--------------------------------------------------------------------------------
--------------------------------------------------------------------------------
--------------------------------------------------------------------------------
--------------------------------------------------------------------------------
--------------------------------------------------------------------------------
--------------------------------------------------------------------------------
--------------------------------------------------------------------------------
--------------------------------------------------------------------------------
--------------------------------------------------------------------------------
--------------------------------------------------------------------------------
--------------------------------------------------------------------------------
--------------------------------------------------------------------------------
--------------------------------------------------------------------------------
--------------------------------------------------------------------------------
--------------------------------------------------------------------------------
--------------------------------------------------------------------------------
--------------------------------------------------------------------------------
--------------------------------------------------------------------------------
--------------------------------------------------------------------------------
--------------------------------------------------------------------------------
--------------------------------------------------------------------------------
--------------------------------------------------------------------------------
--------------------------------------------------------------------------------
```


--AAAGCAATGTATGCCCCTCCCATCAGCGGACAAATTAG
ATGTTCATCAAATATTACAGGGCTGCTATTAACAAGAGATGGTGGTAATAACAACAATGGGTCCGAGATCTTCAGACCTG
GAGGAGGAGATATGAGGGACAATTGGAGAAGTGAATTATATAAATATAAAGTAGTAAAAATTGAACCATTAGGAGTAGCA
CCCACCAAGGCAAAGAGAAGAGTGGTGCAGAGAGAAAAAAGAGCAGTGGGAATAGGAGCTTTGTTCCTTGGGTTCTTGGG
AGCAGCAGGAAGCACTATGGGCGCAGCGTCAATGACGCTGACGGTACAGGCCAGACAATTATTGTCTGGTATAGTGCAGC
AGCAGAACAATTTGCTGAGGGCTATTGAGGCGCAACAGCATCTGTTGCAACTCACAGTCTGGGGCATCAAGCAGCTCCAG
GCAAGAATCCTGGCTGTGGAAAGATACCTAAAGGATCAACAGCTCCTGGGGATTTGGGGTTGCTCTGGAAAACTCATTTG
CACCACTGCTGTGCCTTGGAATGCTAGTTGGAGTAATAAATCTCTGGAACAGATTTGGAATCACACGACCTGGATGGAGT
GGGACAGAGAAATTAACAATTACACAAGCTTAATACACTCCTTAATTGAAGAATCGCAAAACCAGCAAGAAAAGAATGAA
CAAGAATTATTGGAATTAGATAAATGGGCAAGTTTGTGGAATTGGTTTAACATAACAAATTGGCTGTGGTATATAAAATT
ATTCATAATGATAGTAGGAGGCTTGGTAGGTTTAAGAATAGTTTTTGCTGTACTTTCTGTAGTGAATAGAGTTAGGCAGG
GATATTCACCATTATCGTTTCAGACCCACCTCCCAATCCCGAGGGGACCCGACAGGCCCGAAGGAATAGAAGAAGAAGGT
GGAGAGAGAGACAGAGACAGATCCATTCGATTAGTGAACGGATCCTTAGCACTTATCTGGGACGATCTGCGGAGCCTGTG
CCTCTTCAGCTACCACCGCTTGAGAGACTTACTCTTGATTGTAACGAGGATTGTGGAACTTCTGGGACGCAGGGGGTGGG
AAGCCCTCAAATATTGGTGGAATCTCCTACAATATTGGAGTCAGGAGCTAAAGAATAGTGCTGTTAGCTTGCTCAATGCC
ACAGCTATAGCAGTAGCTGAGGGGACAGATAGGGTTATAGAAGTAGTACAAGAAGCTTATAGAGCTATTCGCCACATACC
TAGAAGAATAAGACAGGGCTTGGAAAGGATTTTGCTATAAGATGGGTGGCAAGTGGTCAAAAAGTAGTGTGGTTGGATGG
CCTGCTGTAAGGGAAAGAATGAGACGAGCTGAGCCAGCAGCAGATGGGGTGGGAGCAGCATCTCGAGACCTAGAAAAACA
TGGAGCAATCACAAGTAGCAACACAGCAGCTAACAATGCTGCTTGTGCCTGGCTAGAAGCACAAGAGGAGGAGAAGGTGG
GTTTTCCAGTCACACCTCAGGTACCTTTAAGACCAATGACTTACAAGGCAGCTGTAGATCTTAGCCACTTTTTAAAAGAA
AAGGGGGGACTGGAAGGGCTAATTCACTCCCAACGAAGACAAGATATCCTTGATCTGTGGATCTACCACACACAAGGCTA
CTTCCCTGATTGGCAGAACTACACACCAGGACCAGGGATCAGATATCCACTGACCTTTGGATGGCGCTACAAGCTAGTAC
CAGTTGAGCCAGAGAAGTTAGAAGAAGCCAACAAAGGAGAGAACACCAGCTTGTTACACCCTGTGAGCCTGCATGGAATG
GATGACCCGGAGAGAGAAGTGTTAGAGTGGAGGTTTGACAGCCGCCTAGCATTTCATCACGTGGCCCGAGAGCTGCATCC
GGAGTACTTCAAGAACTGCTGATATCGAGCTTGCTACAAGGGACTTTCCGCTGGGGACTTTCCAGGGAGGCGTGGCCTGG
GCGGGACTGGGGAGTGGCGAGCCCTCAGATCCTGCATATAAGCAGCTGCTTTTTGCCTGTACTGGGTCTCTCTGGTTAGA
CCAGATCTGAGCCTGGGAGCTCTCTGGCTAACTAGGGAACCCACTGCTTAAGCCTCAATAAAGCTTGCCTTGAGTGCTTC
AAGTAGTGTGTGCCCATCTGTTGTGNGACTCTGGNAACTAGAGATCCCTCA
>3-72envLTR


```
--------------------------------------------------------------------------------
--------------------------------------------------------------------------------
--------------------------------------------------------------------------------
--------------------------------------------------------------------------------
--------------------------------------------------------------------------------
--------------------------------------------------------------------------------
--------------------------------------------------------------------------------
--------------------------------------------------------------------------------
--------------------------------------------------------------------------------
--------------------------------------------------------------------------------
--------------------------------------------------------------------------------
--------------------------------------------------------------------------------
--------------------------------------------------------------------------------
--------------------------------------------------------------------------------
--------------------------------------------------------------------------------
--------------------------------------------------------------------------------
--------------------------------------------------------------------------------
--------------------------------------------------------------------------------
--------------------------------------------------------------------------------
--------------------------------------------------------------------------------
--------------------------------------------------------------------------------
--------------------------------------------------------------------------------
--------------------------------------------------------------------------------
--------------------------------------------------------------------------------
--------------------------------------------------------------------------------
--------------------------------------------------------------------------------
--------------------------------------------------------------------------------
--------------------------------------------------------------------------------
--------------------------------------------------------------------------------
--------------------------------------------------------------------------------
--------------------------------------------------------------------------------
--------------------------------------------------------------------------------
--------------------------------------------------------------------------------
--------------------------------------------------------------------------------
--------------------------------------------------------------------------------
--------------------------------------------------------------------------------
--------------------------------------------------------------------------------
--------------------------------------------------------------------------------
--------------------------------------------------------------------------------
--------------------------------------------------------------------------------
--------------------------------------------------------------------------------
--------------------------------------------------------------------------------
--------------------------------------------------------------------------------
--------------------------------------------------------------------------------
---------AATATTACAGGGCTGCTATTAACAAGAGATGGTGGTAATAACAACAATGGGTCCGAGATCTTCAGACCTG
GAGGAGGAGATATGAGGGACAATTGGAGAAGTGAATTATATAAATATAAAGTAGTAAAAATTGAACCATTAGGAGTAGCA
CCCACCAAGGCAAAGAGAAGAGTGGTGCAGAGAGAAAAAAGAGCAGTAGGAATAGGAGCTTTGTTCCTTGGGTTCTTGGG
AGCAGCAGGAAGCACTATGGGCGCAGCGTCAATGACGCTGACGGTACAGGCCAGACAATTATTGTCTGGTATAGTGCAGC
AGCAGAACAATTTGCTGAGGGCTATTGAGGCGCAACAGCATCTGTTGCAACTCACAGTCTGGGGCATCAAGCAGCTCCAG
GCAAGAATCCTGGCTGTGGAAAGATACCTAAAGGATCAACAGCTCCTGGGGATTTGGGGTTGCTCTGGAAAACTCATTTG
CACCACTGCTGTGCCTTGGAATGCTAGTTGGAGTAATAAATCTCTGGAACAGATTTGGAATCACACGACCTGGATGGAGT
GGGACAGAGAAATTAACAATTACACAAGCTTAATACACTCCTTAATTGAAGAATCGCAAAACCAGCAAGAAAAGAATGAA
CAAGAATTATTGGAATTAGATAAATGGGCAAGTTTGTGGAATTGGTTTAACATAACAAATTGGCTGTGGTATATAAAATT
ATTCATAATGATAGTAGGAGGCTTGGTAGGTTTAAGAATAGTTTTTGCTGTACTTTCTGTAGTGAATAGAGTTAGGCAGG
GATATTCACCATTATCGTTTCAGACCCACCTCCCAATCCCGAGGGGACCCGACAGGCCCGAAGGAATAGAAGAAGAAGGT
GGAGAGAGAGACAGAGACAGATCCATTCGATTAGTGAACGGATCCTTAGCACTTATCTGGGACGATCTGCGGAGCCTGTG
CCTCTTCAGCTACCACCGCTTGAGAGACTTACTCTTGATTGTAACGAGGATTGTGGAACTTCTGGGACGCAGGGGGTGGG
AAGCCCTCAAATATTGGTGGAATCTCCTACAATATTGGAGTCAGGAGCTAAAGAATAGTGCTGTTAGCTTGCTCAATGCC
ACAGCTATAGCAGTAGCTGAGGGGACAGATAGGGTTATAGAAGTAGTACAAGAAGCTTATAGAGCTATTCGCCACATACC
TAGAAGAATAAGACAGGGCTTAGAAAGGATTTTGCTATAAGATGGGTGGCAAGTGGTCAAAAAGTAGTGTGGTTAGATGG
CCTGCTGTAAGGGAAAGAATGAGACGAGCTGAGCCAGCAGCAGATGAGGTGGGAGCAGCATCTCGAGACCTAGAAAAACA
TGGAGCAATCACAAGTAGCAACACAGCAGCTAACAATGCTGCTTGTGCCTGGCTAGAAGCACAAGAGGAGGAGAAGGTGG
GTTTTCCAGTCACACCTCAGGTACCTTTAAGACCAATGACTTACAAGGCAGCTGTAGATCTTAGCCACTTTTTAAAAGAA
AAGGGGGGACTGGAAGGGCTAATTCACTCCCAACGAAGACAAGATATCCTTGATCTGTGGATCTACCACACACAAGGCTA
CTTCCCTGATTGGCAGAACTACACACCAGGACCAGGGATCAGATATCCACTGACCTTTGGATGGCGCTACAAGCTAGTAC
CAGTTGAGCCAGAGAAGTTAGAAGAAGCCAACAAAGGAGAGAACACCAGCTTGTTACACCCTGTGAGCCTGCATGGAATG
GATGACC-GGAGAGAGAAGTGTTAGAGTGGAGGTTTGACAGCCGCCTAGCATTTCATCACGTGGCCCGAGAGCTGCATCC
GGAGTACTTCAAGAACTGCTGATATCGAGCTTGCTACAAGGGACTTTCCGCTGGGGACTTTCCAGGGAGGCGTGGCCTGG
GCGGGACTAAGGAGTGGCGAGCCCTCAGATCCTGCATATAAGCAGCTGCTTTTTGCCTGTACTAGGTCTCTCTGGTTAGA
CCAGATCTGAGCCTAGGAGCTCTCTGGCTAACTAGGGAACCCACTGCTTAAGCCTCAATAAAGCTTGCCTTAAGTGCTTC
AAGTAGTGTGTGCCCATCTGTTGTGTGACTCTGGTAACTAGAGATCCCTCA
>3-76envLTR
--------------------------------------------------------------------------------
--------------------------------------------------------------------------------
--------------------------------------------------------------------------------
--------------------------------------------------------------------------------
--------------------------------------------------------------------------------
--------------------------------------------------------------------------------
--------------------------------------------------------------------------------
--------------------------------------------------------------------------------
--------------------------------------------------------------------------------
--------------------------------------------------------------------------------
--------------------------------------------------------------------------------
--------------------------------------------------------------------------------
--------------------------------------------------------------------------------
--------------------------------------------------------------------------------
--------------------------------------------------------------------------------
--------------------------------------------------------------------------------
--------------------------------------------------------------------------------
--------------------------------------------------------------------------------
--------------------------------------------------------------------------------
--------------------------------------------------------------------------------
--------------------------------------------------------------------------------
--------------------------------------------------------------------------------
--------------------------------------------------------------------------------
```


---GGTAATAACAACAATGGGTCCGAGATCTTCAGACCTG
GAGGAGGAGATATGAGGGACAATTGGAGAAGTGAATTATATAAATATAAAGTAGTAAAAATTGAACCATTAGGAGTAGCA
CCCACCAAGGCAAAGAGAAGAGTGGTGCAGAGAGAAAAAAGAGCAGTGGGAATAGGAGCTTTGTTCCTTGGGTTCTTGGG
AGCAGCAGGAAGCACTATGGGCGCAGCGTCAATGACGCTGACGGTACAGGCCAGACAATTATTGTCTGGTATAGTGCAGC
AGCAGAACAATTTGCTGAGGGCTATTGAGGCGCAACAGCATCTGTTGCAACTCACAGTCTGGGGCATCAAGCAGCTCCAG
GCAAGAATCCTGGCTGTGGAAAGATACCTAAAGGATCAACAGCTCCTGGGGATTTGGGGTTGCTCTGGAAAACTCATTTG
CACCACTGCTGTGCCTTGGAATGCTAGTTGGAGTAATAAATCTCTGGAACAGATTTGGAATCACACGACCTGGATGGAGT
GGGACAGAGAAATTAACAATTACACAAGCTTAATACACTCCTTAATTGAAGAATCGCAAAACCAGCAAGAAAAGAATGAA
CAAGAATTATTGGAATTAGATAAATGGGCAAGTTTGTGGAATTGGTTTAACATAACAAATTGGCTGTGGTATATAAAATT
ATTCATAATGATAGTAGGAGGCTTGGTAGGTTTAAGAATAGTTTTTGCTGTACTTTCTGTAGTGAATAGAGTTAGGCAGG
GATATTCACCATTATCGTTTCAGACCCACCTCCCAATCCCGAGGGGACCCGACAGGCCCGAAGGAATAGAAGAAGAAGGT
GGAGAGAGAGACAGAGACAGATCCATTCGATTAGTGAACGGATCCTTAGCACTTATCTGGGACGATCTGCGGAGCCTGTG
CCTCTTCAGCTACCACCGCTTGAGAGACTTACTCTTGATTGTAACGAGGATTGTGGAACTTCTGGGACGCAGGGGGTGGG
AAGCCCTCAAATATTGGTGGAATCTCCTACAATATTGGAGTCAGGAGCTAAAGAATAGTGCTGTTAGCTTGCTCAATGCC
ACAGCTATAGCAGTAGCTGAGGGGACAGATAGGGTTATAGAAGTAGTACAAGAAGCTTATAGAGCTATTCGCCACATACC
TAGAAGAATAAGACAGGGCTTGGAAAGGATTTTGCTATAAGATGGGTGGCAAGTGGTCAAAAAGTAGTGTGGTTGGATGG
CCTGCTGTAAGGGAAAGAATGAGACGAGCTGAGCCAGCAGCAGATGGGGTGGGAGCAGCATCTCGAGACCTAGAAAAACA
TGGAGCAATCACAAGTAGCAACACAGCAGCTAACAATGCTGCTTGTGCCTGGCTAGAAGCACAAGAGGAGGAGAAGGTGG
GTTTTCCAGTCACACCTCAGGTACCTTTAAGACCAATGACTTACAAGGCAGCTGTAGATCTTAGCCACTTTTTAAAAGAA
AAGGGGGGACTGGAAGGGCTAATTCACTCCCAACGAAGACAAGATATCCTTGATCTGTGGATCTACCACACACAAGGCTA
CTTCCCTGATTGGCAGAACTACACACCAGGACCAGGGATCAGATATCCACTGACCTTTGGATGGCGCTACAAGCTAGTAC
CAGTTGAGCCAGAGAAGTTAGAAGAAGCCAACAAAGGAGAGAACACCAGCTTGTTACACCCTGTGAGCCTGCATGGAATG

```
GATGACCCGGAGAGAGAAGTGTTAGAGTGGAGGTTTGACAGCCGCCTAGCATTTCATCACGTGGCCCGAGAGCTGCATCC
GGAGTACTTCAAGAACTGCTGATATCGAGCTTGCTACAAGGGACTTTCCGCTGGGGACTTTCCAGGGAGGCGTGGCCTGG
GCGGGACTGGGGAGTGGCGAGCCCTCAGATCCTGCATATAAGCAGCTGCTTTTTGCCTGTACTGGGTCTCTCTGGTTAGA
CCAGATCTGAGCCTGGGAGCTCTCTGGCTAACTAGGGAACCCACTGCTTAAGCCTCAATAAAGCTTGCCTTGAGTGCTTC
AAGTAGTGTGTGCCCATCTGTTGTGTGACTCTGGTAACTAGAGATCCCTCA
>3-78envLTR
--------------------------------------------------------------------------------
--------------------------------------------------------------------------------
--------------------------------------------------------------------------------
--------------------------------------------------------------------------------
--------------------------------------------------------------------------------
--------------------------------------------------------------------------------
--------------------------------------------------------------------------------
--------------------------------------------------------------------------------
--------------------------------------------------------------------------------
--------------------------------------------------------------------------------
--------------------------------------------------------------------------------
--------------------------------------------------------------------------------
--------------------------------------------------------------------------------
--------------------------------------------------------------------------------
--------------------------------------------------------------------------------
--------------------------------------------------------------------------------
--------------------------------------------------------------------------------
--------------------------------------------------------------------------------
--------------------------------------------------------------------------------
--------------------------------------------------------------------------------
--------------------------------------------------------------------------------
--------------------------------------------------------------------------------
--------------------------------------------------------------------------------
--------------------------------------------------------------------------------
--------------------------------------------------------------------------------
--------------------------------------------------------------------------------
--------------------------------------------------------------------------------
--------------------------------------------------------------------------------
--------------------------------------------------------------------------------
--------------------------------------------------------------------------------
--------------------------------------------------------------------------------
--------------------------------------------------------------------------------
--------------------------------------------------------------------------------
--------------------------------------------------------------------------------
--------------------------------------------------------------------------------
--------------------------------------------------------------------------------
--------------------------------------------------------------------------------
--------------------------------------------------------------------------------
--------------------------------------------------------------------------------
--------------------------------------------------------------------------------
--------------------------------------------------------------------------------
--------------------------------------------------------------------------------
--------------------------------------------------------------------------------
--------------------------------------------------------------------------------
--------------------------------------------------------------------------------
--------------------------------------------------------------------------------
--------------------------------------------------------------------------------
--------------------------------------------------------------------------------
--------------------------------------------------------------------------------
--------------------------------------------------------------------------------
--------------------------------------------------------------------------------
--------------------------------------------------------------------------------
--------------------------------------------------------------------------------
--------------------------------------------------------------------------------
--------------------------------------------------------------------------------
--------------------------------------------------------------------------------
--------------------------------------------------------------------------------
--------------------------------------------------------------------------------
--------------------------------------------------------------------------------
--------------------------------------------------------------------------------
--------------------------------------------------------------------------------
--------------------------------------------------------------------------------
--------------------------------------------------------------------------------
--------------------------------------------------------------------------------
--------------------------------------------------------------------------------
--------------------------------------------------------------------------------
--------------------------------------------------------------------------------
--------------------------------------------------------------------------------
--------------------------------------------------------------------------------
--------------------------------------------------------------------------------
--------------------------------------------------------------------------------
--------------------------------------------------------------------------------
--------------------------------------------------------------------------------
--------------------------------------------------------------------------------
--------------------------------------------------------------------------------
--------------------------------------------------------------------------------
--------------------------------------------------------------------------------
--------------------------------------------------------------------------------
--------------------------------------------------------------------------------
--------------------------------------------------------------------------------
--------------------------------------------------------------------------------
--------------------------------------------------------------------------------
--------------------------------------------------------------------------------
--------------------------------------------------------------------------------
--------------------------------------------------------------------------------
--------------------------------------------------------------------------------
--------------------------------------------------------------------------------
--------------------------------------------------------------------------------
```


----------------GGACAATTGGAGAAGTGAATTATATAAATATAAAGTAGTAAAAATTGAACCATTAGGAGTAGCA
CCCACCAAGGCAAAGAGAAGAGTGGTGCAGAGAGAAAAAAGAGCAGTAAGAATAAGAGCTTTGTTCCTTAGGTTCTTAGG
AGCAGCAAGAAGCACTATAGGCGCAGCGTCAATGACGCTGACGGTACAGGCCAGACAATTATTGTCTGGTATAGTGCAGC
AGCAGAACAATTTGCTGAGGGCTATTGAGGCGCAACAGCATCTGTTGCAACTCACAGTCTGGGGCATCAAGCAGCTCCAG
GCAAGAATCCTGGCTGTAGAAAGATACCTAAAGGATCAACAGCTCCTGGGGATTTGGGGTTGCTCTGGAAAACTCATTTG
CACCACTGCTGTGCCTTGGAATGCTAGTTGGAGTAATAAATCTCTGGAACAGATTTAGAATCACACGACCTGGATGGAGT
AGGACAGAGAAATTAACAATTACACAAGCTTAATACACTCCTTAATTAAAGAATCGCAAAACCAGCAAGAAAAGAATGAA
CAAGAATTATTAGAATTAGATAAATAGGCAAGTTTGTGGAATTGGTTTAACATAACAAATTGGCTGTGGTATATAAAATT
ATTCATAATGATAGTAGGAGGCTTGGTAGGTTTAAGAATAGTTTTTGCTGTACTTTCTGTAGTGAATAGAGTTAGGCAGG
GATATTCACCATTATCGTTTCAGACCCACCTCCCAATCCCGAGGGGACCCGACAGGCCCGAAGGAATAGAAGAAGAAGGT
GGAGAGAGAGACAGAGACAGATCCATTCGATTAGTGAACGGATCCTTAGCACTTATCTGGGACGATCTGCGGAGCCTGTG
CCTCTTCAGCTACCACCGCTTGAGAGACTTACTCTTGATTGTAACGAGGATTGTGGAACTTCTAGGACGCAGGGGGTGGG
AAGCCCTCAAATATTGGTGGAATCTCCTACAATATTAGAGTCAGGAGCTAAAGAATAGTGCTGTTAGCTTGCTCAATGCC
ACAGCTATAGCAGTAGCTGAGGGGACAGATAAGGTTATAGAAGTAGTACAAGAAGCTTATAGAGCTATTCGCCACATACC
TAGAAGAATAAGACAGGGCTTGGAAAGGATTTTGCTATAAGATAGGTGGCAAGTGGTCAAAAAGTAGTGTAGTTGGATGG
CCTGCTGTAAGGGAAAGAATGAGACGAGCTGAGCCAGCAGCAGATGGGGTGGGAGCAGCATCTCGAGACCTAGAAAAACA
TGGAGCAATCACAAGTAGCAACACAGCAGCTAACAATGCTGCTTGTGCCTGGCTAGAAGCACAAGAGGAGGAGAAGGTAA
GTTTTCCAGTCACACCTCAGGTACCTTTAAGACCAATGACTTACAAGGCAGCTGTAGATCTTAGCCACTTTTTAAAAGAA
AAGGGGGGACTGGAAAGGCTAATTCACTCCCAACGAAGACAAGATATCCTTGATCTGTGGATCTACCACACACAAGGCTA
CTTCCCTGATTGGCAGAACTACACACCAGGACCAGGGATCAGATATCCACTGACCTTTGGATGGCGCTACAAGCTAGTAC
CAGTTGAGCCAGAGAAGTTAGAAGAAGCCAACAAAGGAGAGAACACCAGCTTGTTACACCCTGTGAGCCTGCATAGAATA
GATGACCCGGAGAGAGAAGTGTTAGAGTGGAGGTTTGACAGCCGCCTAGCATTTCATCACGTGGCCCGAGAGCTGCATCC
AGAGTACTTCAAGAACTGCTGATATCGAGCTTGCTACAAAGGACTTTCCGCTGAGGACTTTCCAGGGAGGCGTGGCCTAG
GCGGGACTAAGGAGTGGCGAGCCCTCAGATCCTGCATATAAGCAGCTGCTTTTTGCCTGTACTAGGTCTCTCTGGTTAGA
CCAGATCTGAGCCTAGGAGCTCTCTGGCTAACTAAGGAACCCACTGCTTAAGCCTCAATAAAGCTTGCCTTAAGTGCTTC
AAGTAGTGTGTGCCCATCTGTTGTGTGACTCTGGTAACTAGAGATCCCTCA
>4-BenvLTR

----------AATATTACAGGGCTGCTATTAACAAAAGATAGTAGTAATAACAACAATGGGTCCGAGATCTTCAGACCTA
GAAGAGGAGATATGAAGGACAATTAGAGAAGTGAATTATATAAATATAAAGTAGTAAAAATTGAACCATTAGGAGTAGCA
CCCACCAAGGCAAAGAGAAGAGTGGTGCAGAGAGAAAAAAGAGCAGTAAGAATAAGAGCTTTGTTCCTTAAGTTCTTAGG
AGCAGCAGGAAGCACTATAGGCGCAGCGTCAATGACGCTGACGGTACAGGCCAGACAATTATTGTCTGGTATAGTGCAGC
AGCAGAACAATTTGCTGAGGGCTATTGAGGCGCAACAGCATCTGTTGCAACTCACAGTCTAAGGCATCAAGCAGCTCCAG
GCAAGAATCCTGGCTGTAGAAAGATACCTAAAGGATCAACAGCTCCTGAGGATTTGGGGTTGCTCTAGAAAACTCATTTG
CACCACTGCTGTGCCTTAGAATGCTAGTTGGAGTAATAAATCTCTAGAACAGATTTAGAATCACACGACCTGGATGAAGT
AGGACAAAGAAATTAACAATTACACAAGCTTAATACACTCCTTAATTGAAGAATCGCAAAACCAGCAAGAAAAGAATGAA
CAAGAATTATTGGAATTAGATAAATGGGCAAGTTTGTGGAATTGGTTTAACATAACAAATTGGCTGTGGTATATAAAATT
ATTCATAATGATAGTAGGAGGCTTGGTAGGTTTAAGAATAGTTTTTGCTGTACTTTCTGTAGTGAATAGAGTTAGGCAAG
GATATTCACCATTATCGTTTCAGACCCACCTCCCAATCCCGAGGGGACCCGACAGGCCCGAAAGAATAGAAGAAGAAGGT
GGAGAGAGAGACAGAGACAGATCCATTCGATTAGTGAACGGATCCTTAGCACTTATCTAGGACGATCTGCGGAGCCTGTG
CCTCTTCAGCTACCACCGCTTGAGAGACTTACTCTTGATTGTAACGAGGATTGTAGAACTTCTAGGACGCAGGGGGTGGG
AAGCCCTCAAATATTGGTAGAATCTCCTACAATATTGGAGTCAGGAGCTAAAGAATAGTGCTGTTAGCTTGCTCAATGCC
ACAGCTATAGCAGTAGCTGAAAGGACAGATAAGGTTATAGAAGTAGTACAAGAAGCTTATAGAGCTATTCGCCACATACC
TAGAAGAATAAGACAGGGCTTGGAAAGGATTTTGCTATAAGATGGGTGGCAAGTGGTCAAAAAGTAGTGTGGTTAGATGG
CCTGCTGTAAGGGAAAGAATGAGACGAGCTAAGCCAGCAGCAGATGGGGTGGGAGCAGCGTCTCGAGACCTAGAAAAACA
TGGAGCAATCACAAGTAGCAACACAGCAGCTAACAATGCTGCTTGTGCCTGGCTAGAAGCACAAGAGGAGGAGAAGGTGG
GTTTTCCAGTCACACCTCAGGTACCTTTAAGACCAATGACTTACAAGGCAGCTGTAGATCTTAGCCACTTTTTAAAAGAA
AAGGGGGGACTGGAAGGGCTAATTCACTCCCAACGAAGACAAGATATCCTTGATCTGTGGATCTACCACACACAAGGCTA
CTTCCCTGATTGGCAGAACTACACACCAGGACCAGGGATCAGATATCCACTGACCTTTGGATGGCGCTACAAGCTAGTAC
CAGTTGAGCCAGAGAAGTTAGAAGAAGCCAACAAAGGAGAGAACACCAGCTTGTTACACCCTGTGAGCCTGCATGGAATG
GATGACCCGGAGAGAGAAGTGTTAGAGTGGAGGTTTGACAGCCGCCTAGCATTTCATCACGTGGCCCGAGAGCTGCATCC
GGAGTACTTCAAGAACTGCTGATATCGAGCTTGCTACAAGAGACTTTCCGCTAAGGACTTTCCAGGGAGGCGTGGCCTGG
GCAGGACTAGGGAGTGGCGAGCCCTCAGATCCTGCATATAAGCAGCTGCTTTTTGCCTGTACTAGGTCTCTCTAGTTAGA
CCAGATCTGAGCCTGGGAGCTCTCTGGCTAACTAAGGAACCCACTGCTTAAGCCTCAATAAAGCTTGCCTTGAGTGCTTC
AAGTAGTGTGTGCCCATCTGTTGTGTGACTCTGGTAACTAGAGATCCCTCA
>4-2envLTR

-----------------------------TAACAAGAGATGGTAGTAATAACAACAATGGGTCCGAGATCTTCAGACCTA
GAGGAGGAGATATGAGGGACAATTGGAGAAGTGAATTATATAAATATAAAGTAGTAAAAATTGAACCATTAGGAGTAGCA
CCCACCAAGGCAAAGAGAAGAGTGGTGCAGAGAGAAAAAAGAGCAGTGGGAATAGGAGCTTTGTTCCTTAGGTTCTTAGG
AGCAGCAGGAAGCACTATAGGCGCAGCGTCAATGACGCTGACGGTACAGGCCAGACAATTATTGTCTGGTATAGTGCAGC
AGCAGAACAATTTGCTGAGGGCTATTGAGGCGCAACAGCATCTGTTGCAACTCACAGTCTAGGGCATCAAGCAGCTCCAG
GCAAGAATCCTGGCTGTAGAAAGATACCTAAAAGATCAACAGCTCCTGAGGATTTACGGTTGCTCTGGAAAACTCATTTG
CACCACTGCTGTGCCTTAGAATGCTAGTTGGAGTAATAAATCTCTAGAACAGATTTAGAATCACACGACCTAGATGGAGT
AAGACAGAAAAATTAACAATTACACAAGCTTAATACACTCCTTAATTGAAGAATCGCAAAACCAGCAAGAAAAGAATGAA
CAAGAATTATTGGAATTAGATAAATGGGCAAGTTTGTAGAATTAGTTTAACATAACAAATTGGCTGTGGTATATAAAATT
ATTCATAATGATAGTAAGAGGCTTAGTAGGTTTAAGAATAGTTTTTGCTGTACTTTCTGTAGTGAATAGAGTTAGGCAAA
GATATTCACCATTATCGTTTCAGACCCACCTCCCAATCCCGAGGGGACCCGACAGGCCCGAAGGAATAGAAGAAGAAGGT
GGAGAGAGAGACAGAGACAGATCCATTCGATTAGTGAACGGATCCTTAGCACTTATCTAGGACGATCTGCGGAGCCTGTG
CCTCTTCAGCTACCACCGCTTAAGAGACTTACTCTTGATTGTAACGAGGATTGTAGAACTTCTAGGACGCAGGGGGTAGG
AAGCCCTCAAATATTGGTAGAATCTCCTACAATATTAGAGTCAGGAGCTAAAGAATAGTGCTGTTAGCTTGCTCAATGCC
ACAGCTATAGCAGTAGCTGAGAGGACAGATAAGGTTATAGAAGTAGTACAAGAAGCTTATAGAGCTATTCGCCACATACC
TAGAAGAATAAGACAGGGCTTAGAAAGGATTTTGCTATAAGATAGGTGGCAAGTGGTCAAAAAGTAGTGTGGTTAGATGG
CCTGCTGTAAAGGAAAGAATGAGACGAGCTAAGCCAGCAGCAGATGGGGTAGGAGCAGCATCTCGAGACCTAGAAAAACA
TGGAGCAATCACAAGTAGCAACACAGCAGCTAACAATGCTGCTTGTGCCTGGCTAGAAGCACAAGAGGAGGAGAAGGTAG
GTTTTCCAGTCACACCTCAGGTACCTTTAAGACCAATGACTTACAAGGCAGCTGTAGATCTTAGCCACTTTTTAAAAGAA
AAGGGGGGACTGGAAGGGCTAATTCACTCCCAACGAAGACAAGATATCCTTGATCTGTGGATCTACCACACACAAGGCTA
CTTCCCTGATTGGCAGAACTACACACCAGGACCAGGGATCAGATATCCACTGACCTTTGGATGGCGCTACAAGCTAGTAC
CAGTTGAGCCAGAGAAGTTAGAAGAAGCCAACAAAGGAGAGAACACCAGCTTGTTACACCCTGTGAGCCTGCATAGAATG
GATGACCCGGAGAGAGAAGTGTTAGAGTGGAGGTTTGACAGCCGCCTAGCATTTCATCACGTGGCCCGAGAGCTGCATCC
GGAGTACTTCAAGAACTGCTGATATCGAGCTTGCTACAAAGGACTTTCCGCTGGGGACTTTCCAGGGAGGCGTGGCCTAG
GCAAGACTGGGGAGTGGCGAGCCCTCAGATCCTGCATATAAGCAGCTGCTTTTTGCCTGTACTAGGTCTCTCTAGTTAGA
CCAGATCTGAGCCTAGGAGCTCTCTGGCTAACTAAGGAACCCACTGCTTAAGCCTCAATAAAGCTTGCCTTGAGTGCTTC
AAGTAGTGTGTGCCCATCTGTTGTGTGACTCTGGTAACTAGAGATCCCTCA

>4-3envLTR

--GGTGGTAATAACAACAATGGGTCCGAGATCTTCAGACCTG
GAGGAGGAGATATGAGGGACAATTGGAGAAGTGAATTATATAAATATAAAGTAGTAAAAATTGAACCATTAGGAGTAGCA
CCCACCAAGGCAAAGAGAAGAGTGGTGCAGAGAGAAAAAAGAGCAGTGGGAATAGGAGCTTTGTTCCTTGGGTTCTTGGG
AGCAGCAGGAAGCACTATGGGCGCAGCGTCAATGACGCTGACGGTACAGGCCAGACAATTATTGTCTGGTATAGTGCAGC

```
AGCAGAACAATTTGCTGAGGGCTATTGAGGCGCAACAGCATCTGTTGCAACTCACAGTCTGGGGCATCAAGCAGCTCCAG
GCAAGAATCCTGGCTGTGGAAAGATACCTAAAGGATCAACAGCTCCTGGGGATTTGGGGTTGCTCTGGAAAACTCATTTG
CACCACTGCTGTGCCTTGGAATGCTAGTTGGAGTAATAAATCTCTGGAACAGATTTGGAATCACACGACCTGGATGGAGT
GGGACAGAGAAATTAACAATTACACAAGCTTAATACACTCCTTAATTGAAGAATCGCAAAACCAGCAAGAAAAGAATGAA
CAAGAATTATTGGAATTAGATAAATGGGCAAGTTTGTGGAATTGGTTTAACATAACAAATTGGCTGTGGTATATAAAATT
ATTCATAATGATAGTAGGAGGCTTGGTAGGTTTAAGAATAGTTTTTGCTGTACTTTCTGTAGTGAATAGAGTTAGGCAGG
GATATTCACCATTATCGTTTCAGACCCCCCTCCCAATCCCGAGGGGACCCGACAGGCCCGAAGGAATAGAAGAAGAAGGT
GGAGAGAGAGACAGAGACAGATCCATTCGATTAGTGAACGGATCCTTAGCACTTATCTGGGACGATCTGCGGAGCCTGTG
CCTCTTCAGCTACCACCGCTTGAGAGACTTACTCTTGATTGTAACGAGGATTGTGGAACTTCTGGGACGCAGGGGGTGGG
AAGCCCTCAAATATTGGTGGAATCTCCTACAATATTGGAGTCAGGAGCTAAAGAATAGTGCTGTTAGCTTGCTCAATGCC
ACAGCTATAGCAGTAGCTGAGGGGACAGATAGGGTTATAGAAGTAGTACAAGAAGCTTATAGAGCTATTCGCCACATACC
TAGAAGAATAAGACAGGGCTTGGAAAGGATTTTGCTATAAGATGGGTGGCAAGTGGTCAAAAAGTAGTGTGGTTGGATGG
CCTGCTGTAAGGGAAAGAATGAGACGAGCTGAGCCAGCAGCAGATGGGGTGGGAGCAGCATCTCGAGACCTAGAAAAACA
TGGAGCAATCACAAGTAGCAACACAGCAGCTAACAATGCTGCTTGTGCCTGGCTAGAAGCACAAGAGGAGGAGAAGGTGG
GTTTTCCAGTCACACCTCAGGTACCTTTAAGACCAATGACTTACAAGGCAGCTGTAGATCTTAGCCACTTTTTAAAAGAA
AAGGGGGGACTGGAAGGGCTAATTCACTCCCAACGAAGACAAGATATCCTTGATCTGTGGATCTACCACACACAAGGCTA
CTTCCCTGATTGGCAGAACTACACACCAGGACCAGGGATCAGATATCCACTGACCTTTGGATGGCGCTACAAGCTAGTAC
CAGTTGAGCCAGAGAAGTTAGAAGAAGCCAACAAAGGAGAGAACACCAGCTTGTTACACCCTGTGAGCCTGCATGGAATG
GATGACCCGGAGAGAGAAGTGTTAGAGTGGAGGTTTGACAGCCGCCTAGCATTTCATCACGTGGCCCGAGAGCTGCATCC
GGAGTACTTCAAGAACTGCTGATATCGAGCTTGCTACAAGGGACTTTCCGCTGGGGACTTTCCAGGGAGGCGTGGCCTGG
GCGGGACTGGGGAGTGGCGAGCCCTCAGATCCTGCATATAAGCAGCTGCTTTTTGCCTGTACTGGGTCTCTCTGGTTAGA
CCAGATCTGAGCCTGGGAGCTCTCTGGCTAACTAGGGAACCCACTGCTTAAGCCTCAATAAAGCTTGCCTTGAGTGCTTC
AAGTAGTGTGTGCCCATCTGTTGTGTGACTCTGGTAACTAGAGATCCCTCA
>4-17envLTR
--------------------------------------------------------------------------------
--------------------------------------------------------------------------------
--------------------------------------------------------------------------------
--------------------------------------------------------------------------------
--------------------------------------------------------------------------------
--------------------------------------------------------------------------------
--------------------------------------------------------------------------------
--------------------------------------------------------------------------------
--------------------------------------------------------------------------------
--------------------------------------------------------------------------------
--------------------------------------------------------------------------------
--------------------------------------------------------------------------------
--------------------------------------------------------------------------------
--------------------------------------------------------------------------------
--------------------------------------------------------------------------------
--------------------------------------------------------------------------------
--------------------------------------------------------------------------------
--------------------------------------------------------------------------------
--------------------------------------------------------------------------------
--------------------------------------------------------------------------------
--------------------------------------------------------------------------------
--------------------------------------------------------------------------------
--------------------------------------------------------------------------------
--------------------------------------------------------------------------------
--------------------------------------------------------------------------------
--------------------------------------------------------------------------------
--------------------------------------------------------------------------------
--------------------------------------------------------------------------------
--------------------------------------------------------------------------------
--------------------------------------------------------------------------------
--------------------------------------------------------------------------------
--------------------------------------------------------------------------------
--------------------------------------------------------------------------------
--------------------------------------------------------------------------------
--------------------------------------------------------------------------------
--------------------------------------------------------------------------------
--------------------------------------------------------------------------------
--------------------------------------------------------------------------------
--------------------------------------------------------------------------------
--------------------------------------------------------------------------------
--------------------------------------------------------------------------------
--------------------------------------------------------------------------------
--------------------------------------------------------------------------------
--------------------------------------------------------------------------------
--------------------------------------------------------------------------------
--------------------------------------------------------------------------------
--------------------------------------------------------------------------------
--------------------------------------------------------------------------------
--------------------------------------------------------------------------------
--------------------------------------------------------------------------------
--------------------------------------------------------------------------------
--------------------------------------------------------------------------------
--------------------------------------------------------------------------------
--------------------------------------------------------------------------------
--------------------------------------------------------------------------------
--------------------------------------------------------------------------------
--------------------------------------------------------------------------------
--------------------------------------------------------------------------------
--------------------------------------------------------------------------------
--------------------------------------------------------------------------------
--------------------------------------------------------------------------------
--------------------------------------------------------------------------------
--------------------------------------------------------------------------------
--------------------------------------------------------------------------------
--------------------------------------------------------------------------------
--------------------------------------------------------------------------------
--------------------------------------------------------------------------------
--------------------------------------------------------------------------------
--------------------------------------------------------------------------------
--------------------------------------------------------------------------------
```

```
--------------------------------------------------------------------------------
--------------------------------------------------------------------------------
--------------------------------------------------------------------------------
--------------------------------------------------------------------------------
--------------------------------------------------------------------------------
--------------------------------------------------------------------------------
--------------------------------------------------------------------------------
--------------------------------------------------------------------------------
--------------------------------------------------------------------------------
--------------------------------------------------------------------------------
--------------------------------------------------------------------------------
--------------------------------------------------------------------------------
--------------------------------------------------------------------------------
--------------------------------------------------------------------------------
--------------------------------------------------------------------------------
--------------------------------------------------------------------------------
--------------------------------------------------------------------------------
--------------------------------------------------------------------------------
--------------------------------------------------------------------------------
--------------------------------------------------------------------------------
--------------------------------------------------------------------------------
--------------------------------------------------------------------------------
--------------------------------------------------------------------------------
------------------------------------------AAAGCAATGTATGCCCCTCCCATCAGCGGACAAATTAG
ATGTTCATCAAATATTACAGGGCTGCTATTAACAAGAGATGGTGGTAATAACAACAATGGGTCCGAGATCTTCAGACCTA
GAGGAGGAGATATGAAGGACAATTGGAGAAGTGAATTATATAAATATAAAGTAGTAAAAATTGAACCATTAGGAGTAGCA
CCCACCAAGGCAAAGAGAAGAGTGGTGCAGAGAGAAAAAAGAGCAATAGGAATAGGAGCTTTGTTCCTTAGGTTCTTAGG
AGCAGCAGGAAGCACTATAGGCGCAGCGTCAATGACGCTGACGGTACAGGCCAGACAATTATTGTCTGGTATAGTGCAGC
AGCAGAACAATTTGCTGAGGGCTATTGAGGCGCAACAGCATCTGTTGCAACTCACAGTCTAGGGCATCAAGCAGCTCCAG
GCAAGAATCCTGGCTGTAGAAAGATACCTAAAAGATCAACAGCTCCTGGGGATTTGGGGTTGCTCTGGAAAACTCATTTG
CACCACTGCTGTGCCTTAGAATGCTAGTTAGAGTAATAAATCTCTAGAACAGATTTAGAATCACACGACCTGGATGGAGT
AGGACAGAGAAATTAACAATTACACAAGCTTAATACACTCCTTAATTGAAGAATCGCAAAACCAGCAAGAAAAAAATGAA
CAAGAATTATTGGAATTAGATAAATGGGCAAGTTTGTAAAATTAGTTTAACATAACAAATTGGCTGTGGTATATAAAATT
ATTCATAATGATAGTAGGAGGCTTAGTAAGTTTAAGAATAGTTTTTGCTGTACTTTCTGTAGTGAATAAAGTTAGGCAGA
GATATTCACCATTATCGTTTCAGACCCACCTCCCAATCCCGAGGGGACCCGACAGGCCCGAAGGAATAGAAGAAGAAGGT
AGAGAGAGAGACAGAGACAGATCCATTCGATTAGTGAACGGATCCTTAGCACTTATCTAGGACGATCTGCGGAGCCTGTG
CCTCTTCAGCTACCACCGCTTGAGAGACTTACTCTTGATTGTAACGAGGATTGTAGAACTTCTAGGACGCAGGAGGTAGG
AAGCCCTCAAATATTGGTGGAATCTCCTACAATATTGGAGTCAGGAGCTAAAGAATAGTGCTGTTAGCTTGCTCAATGCC
ACAGCTATAGCAGTAGCTGAGGGGACAGATAAGGTTATAGAAGTAGTACAAGAAGCTTATAGAGCTATTCGCCACATACC
TAGAAGAATAAGACAAGGCTTAGAAAAGATTTTGCTATAAGATAGGTGGCAAGTGGTCAAAAAGTAGTGTGGTTAGATGG
CCTGCTGTAAAGGAAAGAATGAGACAAGCTGAGCCAGCAGCAGATGAGGTAGGAGCAGCATCTCAAGACCTAGAAAAACA
TGGAGCAATCACAAGTAGCAACACAGCAGCTAACAATGCTGCTTGTGCCTGGCTAGAAGCACAAGAGGAAGAGAAGGTAA
GTTTTCCAGTCACACCTCAGGTACCTTTAAGACCAATGACTTACAAGGCAGCTGTAGATCTTAGCCACTTTTTAAAAGAA
AAGGGGGGACTGGAAGGGCTAATTCACTCCCAACGAAGACAAGATATCCTTGATCTGTGGATCTACCACACACAAGGCTA
CTTCCCTGATTGGCAGAACTACACACCAGGACCAGGGATCAGATATCCACTGACCTTTAGATGGCGCTACAAGCTAGTAC
CAGTTGAGCCAGAGAAGTTAGAAGAAGCCAACAAAGGAGAGAACACCAGCTTGTTACACCCTGTGAGCCTGCATGGAATG
GATGACCCGGAGAGAGAAGTGTTAGAGTGGAGGTTTGACAGCCGCCTAGCATTTCATCACGTGGCCCGAGAGCTGCATCC
GGAGTACTTCAAGAACTGCTGATATCGAGCTTGCTACAAGGGACTTTCCGCTGAGGACTTTCCAGGGAGGCGTGGCCTAG
GCGGGACTGGGGAGTGGCGAGCCCTCAGATCCTGCATATAAGCAGCTGCTTTTTGCCTGTACTGGGTCTCTCTGGTTAGA
CCAGATCTGAGCCTAGGAGCTCTCTGGCTAACTAAGGAACCCACTGCTTAAGCCTCAATAAAGCTTGCCTTGAGTGCTTC
AAGTAGTGTGTGCCCATCTGTTGTGTGACTCTGGTAACTAGAGATCCCTCA
>4-32envLTR
--------------------------------------------------------------------------------
--------------------------------------------------------------------------------
--------------------------------------------------------------------------------
--------------------------------------------------------------------------------
--------------------------------------------------------------------------------
--------------------------------------------------------------------------------
--------------------------------------------------------------------------------
--------------------------------------------------------------------------------
--------------------------------------------------------------------------------
--------------------------------------------------------------------------------
--------------------------------------------------------------------------------
--------------------------------------------------------------------------------
--------------------------------------------------------------------------------
--------------------------------------------------------------------------------
--------------------------------------------------------------------------------
--------------------------------------------------------------------------------
--------------------------------------------------------------------------------
--------------------------------------------------------------------------------
--------------------------------------------------------------------------------
--------------------------------------------------------------------------------
--------------------------------------------------------------------------------
--------------------------------------------------------------------------------
--------------------------------------------------------------------------------
--------------------------------------------------------------------------------
--------------------------------------------------------------------------------
--------------------------------------------------------------------------------
--------------------------------------------------------------------------------
--------------------------------------------------------------------------------
--------------------------------------------------------------------------------
--------------------------------------------------------------------------------
--------------------------------------------------------------------------------
--------------------------------------------------------------------------------
--------------------------------------------------------------------------------
--------------------------------------------------------------------------------
--------------------------------------------------------------------------------
--------------------------------------------------------------------------------
--------------------------------------------------------------------------------
--------------------------------------------------------------------------------
--------------------------------------------------------------------------------
--------------------------------------------------------------------------------
--------------------------------------------------------------------------------
--------------------------------------------------------------------------------
```


---ATAACAACAATGGGTCCGAGATCTTCANACCTG
AAGGAGGAGATATGAGGGACAATTGGAGAAGTGAATTATATAAATATAAAGTAGTAAAAATTGAACCATTAGGAGTAGCA
CCCACCAAGGCAAAGAGAAGAGTGGTGCAGAGAGAAAAAAGAGCAGTAAGAATAGGAGCTTTGTTCCTTAGGTTCTTAGG
AGCAGCAGGAAGCACTATAGGCGCAGCGTCAATGACGCTGACGGTACAGGCCAGACAATTATTGTCTGGTATAGTGCAGC
AGCAGAACAATTTGCTGAGGGCTATTGAGGCGCAACAGCATCTGTTGCAACTCACAGTCTGGGGCATCAAGCAGCTCCAG
GCAAGAATCCTGGCTGTGGAAAGATACCTAAAGGATCAACAGCTCCTGGGGATTTGGGGTTGCTCTGGAAAACTCATTTG
CACCACTGCTGTGCCTTGGAATGCTAGTTAGAGTAATAAATCTCTGGAACAGATTTGGAATCACACGACCTGGATGGAGT
GGGACAGAGAAATTAACAATTACACAAGCTTAATACACTCCTTAATTGAAGAATCGCAAAACCAGCAAGAAAAGAATGAA
CAAGAATTATTGGAATTAGATAAATGGGCAAGTTTGTGGAATTGGTTTAACATAACAAATTGGCTGTGGTATATAAAATT
ATTCATAATGATAGTAGGAGGCTTAGTAGGTTTAAGAATAGTTTTTGCTGTACTTTCTGTAGTGAATAGAGTTAGGCAGG
GATATTCACCATTATCGTTTCAGACCCACCTCCCAATCCCGAGGGGACCCGACAGGCCCGAAGGAATAGAAGAAGAAGGT
GGAGAGAGAGACAGAGACAGATCCATTCGATTAGTGAACGGATCCTTAGCACTTATCTAGGACGATCTGCGGAGCCTGTG
CCTCTTCAGCTACCACCGCTTGAGAGACTTACTCTTGATTGTAACGAGGATTGTGGAACTTCTGGGACGCAGGGGGTAGG
AAGCCCTCAAATATTGGTGGAATCTCCTACAATATTGGAGTCAGGAGCTAAAGAATAGTGCTGTTAGCTTGCTCAATGCC
ACAGCTATAGCAGTAGCTGAGAGGACAGATAAGGTTATAGAAGTAGTACAAGAAGCTTATAGAGCTATTCGCCACATACC
TAGAAGAATAAGACAGGGCTTGGAAAGGATTTTGCTATAAGATAGGTGGCAAGTGGTCAAAAAGTAGTGTGGTTGGATGG
CCTGCTGTAAGGGAAAGAATGAGACGAGCTGAGCCAGCAGCAGATGGGGTAGGAGCAGCATCTCGAGACCTAGAAAAACA
TGGAGCAATCACAAGTAGCAACACAGCAGCTAACAATGCTGCTTGTGCCTGGCTAGAAGCACAAGAGGAGGAGAAGGTAG
GTTTTCCAGTCACACCTCAGGTACCTTTAAGACCAATGACTTACAAGGCAGCTGTAGATCTTAGCCACTTTTTAAAAGAA
AAGGGGGGACTGGAAGGGCTAATTCACTCCCAACGAAGACAAGATATCCTTGATCTGTGGATCTACCACACACAAGGCTA
CTTCCCTGATTGGCAGAACTACACACCAGGACCAGGGATCAGATATCCACTGACCTTTGGATGGCGCTACAAGCTAGTAC
CAGTTGAGCCAGAGAAGTTAGAAGAAGCCAACAAAGGAGAGAACACCAGCTTGTTACACCCTGTGAGCCTGCATGGAATG
GATGACCCGGAGAGAGAAGTGTTAGAGTGGAGGTTTGACAGCCGCCTAGCATTTCATCACGTGGCCCAAGAGCTGCATCC
GGAGTACTTCAAGAACTGCTGATATCGAGCTTGCTACAAGGGACTTTCCGCTGGGGACTTTCCAGGGAGGCGTGGCCTGG
GCGGGACTGGGGAGTGGCGAGCCCTCAGATCCTGCATATAAGCAGCTGCTTTTTGCCTGTACTAGGTCTCTCTGGTTAGA
CCAGATCTGAGCCTAGGAGCTCTCTGGCTAACTAGGGAACCCACTGCTTAAGCCTCAATAAAGCTTGCCTTGAGTGCTTC
AAGTAGTGTGTGCCCATCTGTTGTGTGACTCTGGTAACTAGAGATCCCTCA
>4-34envLTR


```
--------------------------------------------------------------------------------
--------------------------------------------------------------------------------
--------------------------------------------------------------------------------
--------------------------------------------------------------------------------
--------------------------------------------------------------------------------
--------------------------------------------------------------------------------
--------------------------------------------------------------------------------
--------------------------------------------------------------------------------
--------------------------------------------------------------------------------
--------------------------------------------------------------------------------
--------------------------------------------------------------------------------
--------------------------------------------------------------------------------
--------------------------------------------------------------------------------
--------------------------------------------------------------------------------
--------------------------------------------------------------------------------
--------------------------------------------------------------------------------
--------------------------------------------------------------------------------
--------------------------------------------------------------------------------
--------------------------------------------------------------------------------
--------------------------------------------------------------------------------
--------------------------------------------------------------------------------
--------------------------------------------------------------------------------
--------------------------------------------------------------------------------
--------------------------------------------------------------------------------
--------------------------------------------------------------------------------
--------------------------------------------------------------------------------
--------------------------------------------------------------------------------
--------------------------------------------------------------------------------
--------------------------------------------------------------------------------
--------------------------------------------------------------------------------
--------------------------------------------------------------------------------
--------------------------------------------------------------------------------
--------------------------------------------------------------------------------
--------------------------------------------------------------------------------
--------------------------------------------------------------------------------
--------------------------------------------------------------------------------
--------------------------------------------------------------------------------
--------------------------------------------------------------------------------
--------------------------------------------------------------------------------
--------------------------------------------------------------------------------
--------------------------------------------------------------------------------
--------------------------------------------------------------------------------
--------------------------------------------------------------------------------
--------------------------------------------------------------------------------
--------------------------------------------------------------------------------
--------------------------------------------------------------------------------
--------------------------------------------------------------------------------
--------------------------------------------------------------------------------
--------------------------------------------------------------------------------
--------------------------------------------------------------------------------
--------------------------------------------------------------------------------
--------------------------------------------------------------------------------
--------------------------------------------------------------------------------
--------------------------------------------------------------------------------
--------------------------------------------------------------------------------
--------------------------------------------------------------------------------
--------------------------------------------------------------------------------
--------------------------------------------------------------------------------
--------------------------------------------------------------------------------
--------------------------------------------------------------------------------
--------------------------------------------------------------------------------
--------------------------------------------------------------------------------
--------------------------------------------------------------------------------
--------------------------------------------------------------------------------
--------------------------------------------------------------------------------
--------------------------------------------------------------------------------
--------------------------------------------------------------------------------
--------------------------------------------------------------------------------
--------------------------------------------------------------------------------
--------------------------------------------------------------------------------
--------------------------------------------------------------------------------
--------------------------------------------------------------------------------
--------------------------------------------------------------------------------
--------------------------------------------------------------------------------
--------------------------------------------------------------------------------
--------------------------------------------------------------------------------
--------------------------------------------------------------------------------
--------------------------------------------------------------------------------
--------------------------------------------------------------------------------
--------------------------------------------------------------------------------
-----------------------------------AAGATAGTAGTAATAACAACAATAGGTCCGAGATCTTCANACCCG
GAGGAGGAGATGTGAANGACAATTGAAGAAGTGAATTATATAAATATAAAGTAGTAAAAATTGAACCATTAAGAGTAGCA
CCCACCAAGGCAAAGAGAAGAGTGGTGCAGAGAGAAAAAAGAGCAGTAAGAATAAGAGCTTTGTTCCTTAAGTTCTTAGG
AGCAGCAAGAAGCACTATAGGCGCAGCGTCAATGACGCTGACGGTACAGGCCAGACAATTATTGTCTAGTATAGTGCAGC
AGCAGAACAATTTGCTGAAGGCTATTGAAGCGCAACAGCATCTGTTGCAACTCACAGTCTAAGGCATCAAGCAGCTCCAG
GCAAGAATCCTGGCTGTAGAAAGATACCTAAAAGATCAACAGCTCCTAAGGATTTAAGGTTGCTCTAGAAAACTCATTTG
CACCACTGCTGTGCCTTAGAATGCTAGTTAGAGTAATAAATCTCTAGAACAGATTTAGAATCACACGACCTAGATAGAGT
AAGACAGAGAAATTAACAATTACACAAGCTTAATACACTCCTTAATTGAAGAATCGCAAAACCAGCAAGAAAAGAATGAA
CAAGAATTATTAGAATTAGATAAATAGGCAAGTTTGTAGAATTAGTTTAACATAACAAATTGGCTGTAGTATATAAAATT
ATTCATAATGATAGTAAGAGGCTTAGTAAGTTTAAGAATAGTTTTTGCTGTACTTTCTGTAGTGAATAAAGTTAGGCAAA
GATATTCACCATTATCGTTTCAGACCCACCTCCCAATCCCGAGGGGACCCGACAGGCCCGAAAGAATAGAAGAAGAAGGT
GGAGAGAGAGACAGAGACAGATCCATTCGATTAGTGAACGGATCCTTAGCACTTATCTGGGACGATCTGCGGAGCCTGTG
CCTCTTCAGCTACCACCGCTTGAGAGACTTACTCTTGATTGTAACGAGGATTGTGGAACTTCTGGGACGCAGGGGGTGGG
AAGCCCTCAAATATTGGTGGAATCTCCTACAATATTGGAGTCAGGAGCTAAAGAATAGTGCTGTTAGCTTGCTCAATGCC
```

```
ACAGCTATAGCAGTAGCTGAGGGGACAGATAGGGTTATAGAAGTAGTACAAGAAGCTTATAGAGCTATTCGCCACATACC
TAGAAGAATAAGACAGGGCTTGGAAAGGATTTTGCTATAAGATGGGTGGCAAGTGGTCAAAAAGTAGTGTGGTTGGATGG
CCTGCTGTAAGGGAAAGAATGAGACGAGCTGAGCCAGCAGCAGATGGGGTGGGAGCAGCATCTCGAGACCTAGAAAAACA
TGGAGCAATCACAAGTAGCAACACAGCAGCTAACAATGCTGCTTGTGCCTGGCTAGAAGCACAAGAGGAGGAGAAGGTGG
GTTTTCCAGTCACACCTCAGGTACCTTTAAGACCAATGACTTACAAGGCAGCTGTAGATCTTAGCCACTTTTTAAAAGAA
AAGGGGGGACTGGAAAGGCTAATTCACTCCCAACGAAGACAAGATATCCTTGATCTGTGGATCTACCACACACAAGGCTA
CTTCCCTGATTGGCAGAACTACACACCAGGACCAGGGATCAGATATCCACTGACCTTTAGATGGCGCTACAAGCTAGTAC
CAGTTGAGCCAGAGAAGTTAGAAGAAGCCAACAAAGGAGAGAACACCAGCTTGTTACACCCTGTGAGCCTGCATAGAATA
GATGACCCGGAGAGAGAAGTGTTAGAGTGGAGGTTTGACAGCCGCCTNGCATTTCATCACGTGGCCCAAGAGCTGCATCC
GGAGTACTTCAAGAACTGCTGATATCGAGCTTGCTACAAGGGACTTTCCGCTAGGGACTTTCCAGGGAGGCGTGGCCTAG
GCGGGACTAGAGAGTGGCGAGCCCTCAGATCCTGCATATAAGCAGCTGCTTTTTGCCTGTACTAGGTCTCTCTGGTTAGA
CCAGATCTGAGCCTGGGAGCTCTCTGGCTAACTAAGGAACCCACTGCTTAAGCCTCAATAAAGCTTGCCTTGAGTGCTTC
AAGTAGTGTGTGCCCATCTGTTGTGTGACTCTAGTAACTAGAGATCCCTCA
>4-38envLTR
--------------------------------------------------------------------------------
--------------------------------------------------------------------------------
--------------------------------------------------------------------------------
--------------------------------------------------------------------------------
--------------------------------------------------------------------------------
--------------------------------------------------------------------------------
--------------------------------------------------------------------------------
--------------------------------------------------------------------------------
--------------------------------------------------------------------------------
--------------------------------------------------------------------------------
--------------------------------------------------------------------------------
--------------------------------------------------------------------------------
--------------------------------------------------------------------------------
--------------------------------------------------------------------------------
--------------------------------------------------------------------------------
--------------------------------------------------------------------------------
--------------------------------------------------------------------------------
--------------------------------------------------------------------------------
--------------------------------------------------------------------------------
--------------------------------------------------------------------------------
--------------------------------------------------------------------------------
--------------------------------------------------------------------------------
--------------------------------------------------------------------------------
--------------------------------------------------------------------------------
--------------------------------------------------------------------------------
--------------------------------------------------------------------------------
--------------------------------------------------------------------------------
--------------------------------------------------------------------------------
--------------------------------------------------------------------------------
--------------------------------------------------------------------------------
--------------------------------------------------------------------------------
--------------------------------------------------------------------------------
--------------------------------------------------------------------------------
--------------------------------------------------------------------------------
--------------------------------------------------------------------------------
--------------------------------------------------------------------------------
--------------------------------------------------------------------------------
--------------------------------------------------------------------------------
--------------------------------------------------------------------------------
--------------------------------------------------------------------------------
--------------------------------------------------------------------------------
--------------------------------------------------------------------------------
--------------------------------------------------------------------------------
--------------------------------------------------------------------------------
--------------------------------------------------------------------------------
--------------------------------------------------------------------------------
--------------------------------------------------------------------------------
--------------------------------------------------------------------------------
--------------------------------------------------------------------------------
--------------------------------------------------------------------------------
--------------------------------------------------------------------------------
--------------------------------------------------------------------------------
--------------------------------------------------------------------------------
--------------------------------------------------------------------------------
--------------------------------------------------------------------------------
--------------------------------------------------------------------------------
--------------------------------------------------------------------------------
--------------------------------------------------------------------------------
--------------------------------------------------------------------------------
--------------------------------------------------------------------------------
--------------------------------------------------------------------------------
--------------------------------------------------------------------------------
--------------------------------------------------------------------------------
--------------------------------------------------------------------------------
--------------------------------------------------------------------------------
--------------------------------------------------------------------------------
--------------------------------------------------------------------------------
--------------------------------------------------------------------------------
--------------------------------------------------------------------------------
--------------------------------------------------------------------------------
--------------------------------------------------------------------------------
--------------------------------------------------------------------------------
--------------------------------------------------------------------------------
--------------------------------------------------------------------------------
--------------------------------------------------------------------------------
--------------------------------------------------------------------------------
--------------------------------------------------------------------------------
--------------------------------------------------------------------------------
--------------------------------------------------------------------------------
--------------------------------------------------------------------------------
```


--TG
GAGGAGGAGANATGAAGGACAATTGGAGAAGTGAATTATATAAATATAAAGTAGTAAAATTGAACCNTTAGGAGTAGCA
CCCACCAAGGCAAAGAGAAGAGTGGTGCAGAGAGAAAAAAGAGCAGTAGGAATAGGAGCTTTGTTCCTTGGGTTCTTAGG
AGCAGCAGGAAGCACTATAGGCGCAGCGTCAATGACGCTGACGGTACAGGCCAGACAATTATTGTCTGGTATAGTGCAGC
AGCAGAACAATTTGCTGAGGGCTATTGAGGCGCAACAGCATCTGTTGCAACTCACAGTCTGGGGCATCAAGCAGCTCCAG
GCAAGAATCCTGGCTGTGGAAAGATACCTAAAGGATCAACAGCTCCTAAGGATTTGAGGTTGCTCTGGAAAACTCATTTG
CACCACTGCTGTGCCTTGGAATGCTAGTTGGAGTAATAAATCTCTGGAACAGATTTAGAATCACACGACCTGGATGGAGT
GGGACAGAGAAATTAACAATTACACAAGCTTAATACACTCCTTAATTGAAGAATCGCAAAACCAGCAAGAAAAGAATGAA
CAAGAATTATTAGAATTAGATAAATGGGCAAGTTTGTGGAATTAGTTTAACATAACAAATTGGCTGTGGTATATAAAATT
ATTCATAATGATAGTAGGAGGCTTGGTAGGTTTAAGAATAGTTTTTGCTGTACTTTCTGTAGTGAATAGAGTTAGGCAGG
GATATTCACCATTATCGTTTCAGACCCACCTCCCAATCCCGAGGGGACCCGACAGGCCCGAAGGAATAGAAGAAGAAGGT
GGAGAGAGAGACAGAGACAGATCCATTCGATTAGTGAACGGATCCTTAGCACTTATCTGGGACGATCTGCGGAGCCTGTG
CCTCTTCAGCTACCACCGCTTGAGAGACTTACTCTTGATTGTAACGAGGATTGTGGAACTTCTGGGACGCAGGGGGTGGG
AAGCCCTCAAATATTGGTGGAATCTCCTACAATATTGGAGTCAGGAGCTAAAGAATAGTGCTGTTAGCTTGCTCAATGCC
ACAGCTATAGCAGTAGCTGAGGGGACAGATAGGGTTATAGAAGTAGTACAAGAAGCTTATAGAGCTATTCGCCACATACC
TAGAAGAATAAGACAGGGCTTGGAAAGGATTTTGCTATAAGATAGGTGGCAAGTGGTCAAAAAGTAGTGTAGTTAGATGG
CCTGCTGTAAAGGAAAGAATGAGACGAGCTGAGCCAGCAGCAGATAGGGTAGGAGCAGCATCTCGAGACCTAGAAAAACA
TGGAGCAATCACAAGTAGCAACACAGCAGCTAACAATGCTGCTTGTGCCTGGCTAGAAGCACAAGAGGAAGAGAAGGTAA
GTTTTCCAGTCACACCTCAGGTACCTTTAAGACCAATGACTTACAAGGCAGCTGTAGATCTTAGCCACTTTTTAAAAGAA
AAGGGGGGACTGGAAGGGCTAATTCACTCCCAACGAAGACAAGATATCCTTGATCTGTGGATCTACCACACACAAGGCTA
CTTCCCTGATTGGCAGAACTACACACCAGGACCAGGGATCAGATATCCACTGACCTTTGGATGGCGCTACAAGCTAGTAC
CAGTTGAGCCAGAGAAGTTAGAAGAAGCCAACAAAGGAGAGAACACCAGCTTGTTACACCCTGTGAGCCTGCATGGAATG
GATGACCCGGAGAGAGAAGTGTTAGAGTGGAGGTTTGACAGCCGCCTAGCATTTCATCACGTGGCCCGAGAGCTGCATCC
GGAGTACTTCAAGAACTGCTGATATCGAGCTTGCTACAAGGGACTTTCCGCTGGGGACTTTCCAGGGAGGCGTGGCCTGG
GCGGGACTGGGGAGTGGCGAGCCCTCAGATCCTGCATATAAGCAGCTGCTTTTTGCCTGTACTGGGTCTCTCTGGTTAGA
CCAGATCTGAGCCTGGGAGCTCTCTGGCTAACTAGGGAACCCACTGCTTAAGCCTCAATAAAGCTTGCCTTGAGTGCTTC
AAGTAGTGTGTGCCCATCTGTTGTGTGACTCTGGTAACTAGAGATCCCTCA
>4-44envLTR


```
--------------------------------------------------------------------------------
--------------------------------------------------------------------------------
--------------------------------------------------------------------------------
--------------------------------------------------------------------------------
--------------------------------------------------------------------------------
--------------------------------------------------------------------------------
--------------------------------------------------------------------------------
--------------------------------------------------------------------------------
--------------------------------------------------------------------------------
--------------------------------------------------------------------------------
--------------------------------------------------------------------------------
--------------------------------------------------------------------------------
--------------------------------------------------------------------------------
--------------------------------------------------------------------------------
--------------------------------------------------------------------------------
--------------------------------------------------------------------------------
--------------------------------------------------------------------------------
--------------------------------------------------------------------------------
--------------------------------------------------------------------------------
--------------------------------------------------------------------------------
--------------------------------------------------------------------------------
--------------------------------------------------------------------------------
--------------------------------------------------------------------------------
--------------------------------------------------------------------------------
--------------------------------------------------------------------------------
--------------------------------------------------------------------------------
--------------------------------------------------------------------------------
--------------------------------------------------------------------------------
--------------------------------------------------------------------------------
--------------------------------------------------------------------------------
--------------------------------------------------------------------------------
--------------------------------------------------------------------------------
--------------------------------------------------------------------------------
--------------------------------------------------------------------------------
--------------------------------------------------------------------------------
--------------------------------------------------------------------------------
--------------------------------------------------------------------------------
--------------------------------------------------------------------------------
--------------------------------------------------------------------------------
--------------------------------------------------------------------------------
--------------------------------------------------------------------------------
--------------------------------------------------------------------------------
-----------------------------------------------ATAACAACAATGGGTCCGAGATCTNCANACCTG
GAGGAGGAGATATGAGGGACAATTGGAGAAGTGAATNATATAAATATAAAGTAGTAAAAATTGAACCATTAGGAGTAGCA
CCCACCAAGGCAAAGAGAAGAGTGGTGCAGAGAGAAAAAAGAGCAGTAGGAATAGGAGCTTTGTTCCTTAGGTTCTTAGG
AGCAGCAGGAAGCACTATGGGCGCAGCGTCAATGACGCTGACGGTACAGGCCAGACAATTATTGTCTGGTATAGTGCAGC
AGCAGAACAATTTGCTGAGGGCTATTGAGGCGCAACAGCATCTGTTGCAACTCACAGTCTGGGGCATCAAGCAGCTCCAG
GCAAGAATCCTGGCTGTGGAAAGATACCTAAAGGATCAACAGCTCCTAAGGATTTGAGGTTGCTCTGGAAAACTCATTTG
CACCACTGCTGTGCCTTGGAATGCTAGTTGGAGTAATAAATCTCTGGAACAGATTTAGAATCACACGACCTGGATGGAGT
GGGACAGAGAAATTAACAATTACACAAGCTTAATACACTCCTTAATTGAAGAATCGCAAAACCAGCAAGAAAAGAATGAA
CAAGAATTATTAGAATTAGATAAATGGGCAAGTTTGTGGAATTAGTTTAACATAACAAATTGGCTGTGGTATATAAAATT
ATTCATAATGATAGTAGGAGGCTTGGTAGGTTTAAGAATAGTTTTTGCTGTACTTTCTGTAGTGAATAAAGTTAGGCAGG
GATATTCACCATTATCGTTTCAGACCCACCTCCCAATCCCGAGGGGACCCGACAGGCCCGAAGGAATAGAAGAAGAAGGT
GGAGAGAGAGACAGAGACAGATCCATTCGATTAGTGAACGGATCCTTAGCACTTATCTGGGACGATCTGCGGAGCCTGTG
CCTCTTCAGCTACCACCGCTTGAGAGACTTACTCTTGATTGTAACGAGGATTGTGGAACTTCTAGGACGCAGGGGGTGGG
AAGCCCTCAAATATTGGTGGAATCTCCTACAATATTGGAGTCAGGAGCTAAAGAATAGTGCTGTTAGCTTGCTCAATGCC
ACAGCTATAGCAGTAGCTGAGAGGACAGATAAGGTTATAGAAGTAGTACAAGAAGCTTATAGAGCTATTCGCCACATACC
TAGAAGAATAAGACAGGGCTTGGAAAGGATTTTGCTATAAGATAGGTGGCAAGTGGTCAAAAAGTAGTGTGGTTAGATGG
CCTGCTGTAAAGGAAAGAATGAGACGAGCTGAGCCAGCAGCAGATGAGGTAGGAGCAGCATCTCGAGACCTAGAAAAACA
TGGAGCAATCACAAGTAGCAACACAGCAGCTAACAATGCTGCTTGTGCCTGGCTAGAAGCACAAGAGGAGGAGAAAGTAG
GTTTTCCAGTCACACCTCAGGTACCTTTAAGACCAATGACTTACAAGGCAGCTGTAGATCTTAGCCACTTTTTAAAAGAA
AAGGGGGGACTGGAAGGGCTAATTCACTCCCAACGAAGACAAGATATCCTTGATCTGTGGATCTACCACACACAAGGCTA
CTTCCCTGATTGGCAGAACTACACACCAGGACCAGGGATCAGATATCCACTGACCTTTGGATGGCGCTACAAGCTAGTAC
CAGTTGAGCCAGAGAAGTTAGAAGAAGCCAACAAAGGAGAGAACACCAGCTTGTTACACCCTGTGAGCCTGCATGGAATG
GATGACCCGGAGAGAGAAGTGTTAGAGTGGAGGTTTGACAGCCGCCTAGCATTTCATCACGTGGCCCGAGAGCTGCATCC
GGAGTACTTCAAGAACTGCTGATATCGAGCTTGCTACAAGGGACTTTCCGCTGGGGACTTTCCAGGGAGGCGTGGCCTGG
GCGGGACTGGGGAGTGGCGAGCCCTCAGATCCTGCATATAAGCAGCTGCTTTTTGCCTGTACTGGGTCTCTCTGGTTAGA
CCAGATCTGAGCCTGGGAGCTCTCTGGCTAACTAGGGAACCCACTGCTTAAGCCTCAATAAAGCTTGCCTTGAGTGCTTC
                AAGTAGTGTGTGCCCATCTGTTGTGTGACTCTGGTAACTAGAGATCCCTCA
                                   >4-46envLTR
--------------------------------------------------------------------------------
--------------------------------------------------------------------------------
--------------------------------------------------------------------------------
--------------------------------------------------------------------------------
--------------------------------------------------------------------------------
--------------------------------------------------------------------------------
--------------------------------------------------------------------------------
--------------------------------------------------------------------------------
--------------------------------------------------------------------------------
--------------------------------------------------------------------------------
--------------------------------------------------------------------------------
--------------------------------------------------------------------------------
--------------------------------------------------------------------------------
--------------------------------------------------------------------------------
--------------------------------------------------------------------------------
--------------------------------------------------------------------------------
--------------------------------------------------------------------------------
--------------------------------------------------------------------------------
--------------------------------------------------------------------------------
--------------------------------------------------------------------------------
--------------------------------------------------------------------------------
--------------------------------------------------------------------------------
--------------------------------------------------------------------------------
--------------------------------------------------------------------------------
--------------------------------------------------------------------------------
```


---ACCTG
GAGGAGGAGATATGAGGGACAATTGGAGAAGTGAATTATATAAATATAAAGTAGTAAAAATTGAACCATTAGGAGTAGCA
CCCACCAAGGCAAAGAGAAGAGTGGTGCAGAGAGAAAAAAGAGCAGTGGGAATAGGAGCTTTGTTCCTTAGGTTCTTAGG
AGCAGCAGGAAGCACTATGGGCGCAGCGTCAATGACGCTGACGGTACAGGCCAGACAATTATTGTCTGGTATAGTGCAGC
AGCAGAACAATTTGCTGAGGGCTATTGAGGCGCAACAGCATCTGTTGCAACTCACAGTCTAAGGCATCAAGCAGCTCCAG
GCAAGAATCCTGGCTGTGGAAAGATACCTAAAGGATCAACAGCTCCTGAAGATTTAGGGTTGCTCTGGAAAACTCATTTG
CACCACTGCTGTGCCTTAGAATGCTAGTTGGAGTAATAAATCTCTAGAACAGATTTAGAATCACACGACCTGGATGGAGT
AGGACAGAGAAATTAACAATTACACAAGCTTAATACACTCCTTAATTGAAGAATCGCAAAACCAGCAAGAAAAGAATGAA
CAAGAATTATTAGAATTAGATAAATGGGCAAGTTTGTGGAATTGGTTTAACATAACAAATTGGCTGTGGTATATAAAATT
ATTCATAATGATAGTAGGAGGCTTGGTAGGTTTAAGAATAGTTTTTGCTGTACTTTCTGTAGTGAATAGAGTTAGGCAGG
GATATTCACCATTATCGTTTCAGACCCACCTCCCAATCCCGAGGGGACCCGACAGGCCCGAAGGAATAGAAGAAGAAGGT
AGAGAGAGAGACAGAGACAGATCCATTCGATTAGTGAACGGATCCTTAGCACTTATCTAGGACGATCTGCGGAGCCTGTG
CCTCTTCAGCTACCACCGCTTGAGAGACTTACTCTTGATTGTAACGAAGATTGTAGAACTTCTGGGACGCAGGGGGTAGG
AAGCCCTCAAATATTGGTGGAATCTCCTACAATATTGGAGTCAGGAGCTAAAGAATAGTGCTGTTAGCTTGCTCAATGCC
ACAGCTATAGCAGTAGCTGAGGGGACAGATAAGGTTATAGAAGTAGTACAAGAAGCTTATAGAGCTATTCGCCACATACC
TAGAAGAATAAGACAAGGCTTAGAAAGGATTTTGCTATAAGATGGGTGGCAAGTGGTCAAAAAGTGGTGTGGTTGGATGG
CCTGCTGTAAGGGAAAGAATGAGACGAGCTGAGCCAGCAGCAGATAAGGTAGGAGCAGCATCTCGAGACCTAGAAAAACA
TAGAGCAATCACAAGTAGCAACACAGCAGCTAACAATGCTGCTTGTGCCTGGCTAGAAGCACAAGAGGAGGAGAAGGTAG
GTTTTCCAGTCACACCTCAGGTACCTTTAAGACCAATGACTTACAAGGCAGCTGTAGATCTTAGCCACTTTTTAAAAGAA
AAGGGGGGACTGGAAGGGCTAATTCACTCCCAACGAAGACAAGATATCCTTGATCTGTGGATCTACCACACACAAGGCTA
CTTCCCTGATTGGCAGAACTACACACCAGGACCAGGGATCAGATATCCACTGACCTTTGGATGGCGCTACAAGCTAGTAC
CAGTTGAGCCAGAGAAGTTAGAAGAAGCCAACAAAGGAGAGAACACCAGCTTGTTACACCCTGTGAGCCTGCATGGAATG
GATGACCCGGAGAGAGAAGTGTTAGAGTGGAGGTTTGACAGCCGCCTAGCATTTCATCACGTGGCCCGAGAGCTNCATCC
GGAGTACTTCAAGAACTGCTGATATCGAGCTTGCTACAAAGGACTTTCCGCTGAGGACTTTCCAGGGAGGCGTGGCCTAG

```
GCAGGACTAAGGAGTGGCGAGCCCTCAGATCCTGCATATAAGCAGCTGCTTTTTGCCTGTACTAGGTCTCTCTGGTTAGA
CCAGATCTGAGCCTAGGAGCTCTCTGGCTAACTAAAGAACCCACTGCTTAAGCCTCAATAAAGCTTGCCTTGAGTGCTTC
AAGTAGTGTGTGCCCATCTGTTGTGTGACTCTGGTAACTAGAGATCCCTCA
>4-52envLTR
--------------------------------------------------------------------------------
--------------------------------------------------------------------------------
--------------------------------------------------------------------------------
--------------------------------------------------------------------------------
--------------------------------------------------------------------------------
--------------------------------------------------------------------------------
--------------------------------------------------------------------------------
--------------------------------------------------------------------------------
--------------------------------------------------------------------------------
--------------------------------------------------------------------------------
--------------------------------------------------------------------------------
--------------------------------------------------------------------------------
--------------------------------------------------------------------------------
--------------------------------------------------------------------------------
--------------------------------------------------------------------------------
--------------------------------------------------------------------------------
--------------------------------------------------------------------------------
--------------------------------------------------------------------------------
--------------------------------------------------------------------------------
--------------------------------------------------------------------------------
--------------------------------------------------------------------------------
--------------------------------------------------------------------------------
--------------------------------------------------------------------------------
--------------------------------------------------------------------------------
--------------------------------------------------------------------------------
--------------------------------------------------------------------------------
--------------------------------------------------------------------------------
--------------------------------------------------------------------------------
--------------------------------------------------------------------------------
--------------------------------------------------------------------------------
--------------------------------------------------------------------------------
--------------------------------------------------------------------------------
--------------------------------------------------------------------------------
--------------------------------------------------------------------------------
--------------------------------------------------------------------------------
--------------------------------------------------------------------------------
--------------------------------------------------------------------------------
--------------------------------------------------------------------------------
--------------------------------------------------------------------------------
--------------------------------------------------------------------------------
--------------------------------------------------------------------------------
--------------------------------------------------------------------------------
--------------------------------------------------------------------------------
--------------------------------------------------------------------------------
--------------------------------------------------------------------------------
--------------------------------------------------------------------------------
--------------------------------------------------------------------------------
--------------------------------------------------------------------------------
--------------------------------------------------------------------------------
--------------------------------------------------------------------------------
--------------------------------------------------------------------------------
--------------------------------------------------------------------------------
--------------------------------------------------------------------------------
--------------------------------------------------------------------------------
--------------------------------------------------------------------------------
--------------------------------------------------------------------------------
--------------------------------------------------------------------------------
--------------------------------------------------------------------------------
--------------------------------------------------------------------------------
--------------------------------------------------------------------------------
--------------------------------------------------------------------------------
--------------------------------------------------------------------------------
--------------------------------------------------------------------------------
--------------------------------------------------------------------------------
--------------------------------------------------------------------------------
--------------------------------------------------------------------------------
--------------------------------------------------------------------------------
--------------------------------------------------------------------------------
--------------------------------------------------------------------------------
--------------------------------------------------------------------------------
--------------------------------------------------------------------------------
--------------------------------------------------------------------------------
--------------------------------------------------------------------------------
--------------------------------------------------------------------------------
--------------------------------------------------------------------------------
--------------------------------------------------------------------------------
--------------------------------------------------------------------------------
--------------------------------------------------------------------------------
--------------------------------------------------------------------------------
--------------------------------------------------------------------------------
--------------------------------------------------------------------------------
--------------------------------------------------------------------------------
--------------------------------------------------------------------------------
--------------------------------------------------------------------------------
--------------------------------------------------------------------------------
--------------------------------------------------------------------------------
--------------------------------------------------------------------------------
--------------------------------------------------------------------------------
--------------------------------------------------------------------------------
```

```
--------------------------------------------------------------------------------
--------------------------------------------------------------------------------
--------------------------------------------------------------------------------
------------------------------------------AAAGCAATGTATGCCCCTCCCATCAGCGGACAAATTAG
ATGTTCATCAAATATTACAGGGCTGCTATTAACAAAAGATAGTAGTAATAACAACAATAGGTCCGAGATCTTCAGACCTA
GAGGAGGAGATATGAAGGACAATTAAAGAAGTGAATTATATAAATATAAAGTAGTAAAAATTGAACCATTAGGAGTAGCA
CCCACCAAGGCAAAGAGAAGAGTGGTGCAGAGAGAAAAAAGAGCAGTAAGAATAAGAGCTTTGTTCCTTAAGTTCTTAGG
AGCAGCAGGAAGCACTATAGGCGCAGCGTCAATGACGCTGACGGTACAGGCCAGACAATTATTGTCTAGTATAGTGCAGC
AGCAGAACAATTTGCTGAAGGCTATTGAGGCGCAACAGCATCTGTTGCAACTCACAGTCTAAGGCATCAAGCAGCTCCAG
GCAAGAATCCTGGCTGTAGAAAGATACCTAAAGGATCAACAGCTCCTAAAGATTTGAAGTTGCTCTAGAAAACTCATTTG
CACCACTGCTGTGCCTTAGAATGCTAGTTGGAGTAATAAATCTCTTGAACAGATTTAGAATCACACGACCTCGATAGAGT
GGGACAGAGAAATTAACAATTACACAAGCTTAATACACTCCTTAATTGAAGAATCGCAAAACCAGCAAGAAAAGAATGAA
CAAGAATTATTAGAATTAAATAAATAGGCAAGTTTGTAGAATTTGTTTAACATAACAAATTAGCTGTGGTATATAAAATT
ATTCATAATGATAGTAGGAGGCTTGGTATGTTTAAAAATAGTTTTTGCTGTACTTTCTGTAGTGAATATAGTTAAGCAAA
GATATTCACCATTATCGTTTCAGACCCACCTCCCAATCCCGAGGGGACCCGACAGGCCCGAAGGAATAGAAGAAGAAGGT
GGAGAGAGAGACAGAGACAGATCCATTCAATTAGTGAACGGATCCTTAGCACTTATCTGGGACGATCTGCGGAGCCTGTG
CCTCTTCAGCTACCACCGCTTGAGAGACTTACTCTTCATTGTAACGAGGATTGTGGAACTTCTAGGACGCAGGGGGTGGG
AAGCACTCAAATATTGGTGGAATCTCCTACAATATTGGAGTCAGGAGCTAAAGAATAGTGCTGTTAGCTTGCTCAATGCC
ACAGCTATAGCAGTAGCTGAGAGGACAGATAGGGTTATAGAAGTAGTACAAGAAGCTTATAGAGCTATTCGCCACATACC
TAGAAGAATAAGACAGGGCTTGGAAAGGATTTTGCTATAAGATGGGTGGCAAGTGGTCAAAAGTAGTGTGGTTGGATGG
CCTGCTGTAATGGAAAGAATGAGACGAGCTGAGCCAGCAGCAGATGGGGTGGGAGCAGCATCTCGAGACCTAGAAAAACA
TGGAGCAATCACAAGTAGCAACACAGCAGCTAACAATGCTGCTTGTGCCTGGCTAGAAGCACAAGAGGAGGAGAAGGTAG
GTTTTCCAGTCACACCTCAGGTACCTTTAAGACCAATGACTTACAAGGCAGCTGTAGATCTTAGCCACTTTTTAAAAGAA
AAGGGGGGACTGGAAGGGCTAATTCACTCCCAACGAAGACAAGATATCCTTGATCTGTGGATCTACCACACACAAGGCTA
CTTCCCTGATTGGCAGAACTACACACCAGGACCAGGGATCAGATATCCACTGACCTTTGGATGGCGCTACAAGCTAGTAC
CAGTTGAGCCAGAGAAGTTAGAAGAAGCCAACAAAGGAGAGAACACCAGCTTGTTACACCCTGTGAGCCTGCATGGAATG
GATGACCCGGAGAGAGAAGTGTTAGAGTGGAGGTTTGACAGCCGCCTAGCATTTCATCACGTGGCCCGAGAGCTGCATCC
GGAGTACTTCAAGAACTGCTGATATCGAGCTTGCTACAAGGGACTTTCCGCTGAGGACTTTCCAGGGAGGCGTGGCCTAG
GCAGGACTGAGGAGTGGCGAGCCCTCAGATCCTGCATATAAGCAGCTGCTTTTTGCCTGTACTAGGTCTCTCTAGTTAGA
CCAGATCTGAGCCTAGGAGCTCTCTGGCTAACTAAGGAACCCACTGCTTAAGCCTCAATAAAGCTTGCCTTGAGTGCTTC
AAGTAGTGTGTGCCCATCTGTTGTGTGACTCTGGTAACTAGAGATCCCTCA
>4-57envLTR
--------------------------------------------------------------------------------
--------------------------------------------------------------------------------
--------------------------------------------------------------------------------
--------------------------------------------------------------------------------
--------------------------------------------------------------------------------
--------------------------------------------------------------------------------
--------------------------------------------------------------------------------
--------------------------------------------------------------------------------
--------------------------------------------------------------------------------
--------------------------------------------------------------------------------
--------------------------------------------------------------------------------
--------------------------------------------------------------------------------
--------------------------------------------------------------------------------
--------------------------------------------------------------------------------
--------------------------------------------------------------------------------
--------------------------------------------------------------------------------
--------------------------------------------------------------------------------
--------------------------------------------------------------------------------
--------------------------------------------------------------------------------
--------------------------------------------------------------------------------
--------------------------------------------------------------------------------
--------------------------------------------------------------------------------
--------------------------------------------------------------------------------
--------------------------------------------------------------------------------
--------------------------------------------------------------------------------
--------------------------------------------------------------------------------
--------------------------------------------------------------------------------
--------------------------------------------------------------------------------
--------------------------------------------------------------------------------
--------------------------------------------------------------------------------
--------------------------------------------------------------------------------
--------------------------------------------------------------------------------
--------------------------------------------------------------------------------
--------------------------------------------------------------------------------
--------------------------------------------------------------------------------
--------------------------------------------------------------------------------
--------------------------------------------------------------------------------
--------------------------------------------------------------------------------
--------------------------------------------------------------------------------
--------------------------------------------------------------------------------
--------------------------------------------------------------------------------
--------------------------------------------------------------------------------
--------------------------------------------------------------------------------
--------------------------------------------------------------------------------
--------------------------------------------------------------------------------
--------------------------------------------------------------------------------
--------------------------------------------------------------------------------
--------------------------------------------------------------------------------
--------------------------------------------------------------------------------
--------------------------------------------------------------------------------
--------------------------------------------------------------------------------
--------------------------------------------------------------------------------
--------------------------------------------------------------------------------
--------------------------------------------------------------------------------
--------------------------------------------------------------------------------
--------------------------------------------------------------------------------
--------------------------------------------------------------------------------
--------------------------------------------------------------------------------
--------------------------------------------------------------------------------
--------------------------------------------------------------------------------
--------------------------------------------------------------------------------
--------------------------------------------------------------------------------
```

```
--------------------------------------------------------------------------------
--------------------------------------------------------------------------------
--------------------------------------------------------------------------------
--------------------------------------------------------------------------------
--------------------------------------------------------------------------------
--------------------------------------------------------------------------------
--------------------------------------------------------------------------------
--------------------------------------------------------------------------------
--------------------------------------------------------------------------------
--------------------------------------------------------------------------------
--------------------------------------------------------------------------------
--------------------------------------------------------------------------------
--------------------------------------------------------------------------------
--------------------------------------------------------------------------------
--------------------------------------------------------------------------------
--------------------------------------------------------------------------------
--------------------------------------------------------------------------------
--------------------------------------------------------------------------------
--------------------------------------------------------------------------------
--------------------------------------------------------------------------------
--------------------------------------------------------------------------------
--------------------------------------------------------------------------------
--------------------------------------------------------------------------------
--------------------------------------------------------------------------------
--------------------------------------------------------------------------------
--------------------------------------------------------------------------------
--------------------------------------------------------------------------------
--------------------------------------------------------------------------------
--------------------------------------------------------------------------------
--------------------------------------------------------------------------------
--------------------------------------------------------------------------------
------------------------------------------AAAGCAATGTATGCCCCTCCCATCAGCGGACAAATTAG
ATGTTCATCAAATATTACAGGGCTGCTATTAACAAGAGATGGTGGTAATAACAACAATAGGTCCGAGATCTTCAGACCTA
GAGGAGGAGATATGAAAGACAATTGGAGAAGTGAATTATATAAATATAAAGTAGTAAAAATTGAACCATTAGGAGTAGCA
CCCACCAAGGCAAAGAGAAGAGTGGTGCAGAGAGAAAAAAGAGCAGTAGGAATAGGAGCTTTGTTCCTTAGGTTCTTAGG
AGCAGCAGGAAGCACTATAGGCGCAGCGTCAATGACGCTGACGGTACAGGCCAGACAATTATTGTCTGGTATAGTGCAGC
AGCAGAACAATTTGCTGAGGGCTATTGAGGCGCAACAGCATCTGTTGCAACTCACAGTCTAAGGCATCAAGCAGCTCCAG
GCAAGAATCCTGGCTGTGGAAAGATACCTAAAGGATCAACAGCTCCTAAGGATTTAAGGTTGCTCTGGAAAACTCATTTG
CACCACTGCTGTGCCTTAGAATGCTAGTTAGAGTAATAAATCTCTAGAACAGATTTAGAATCACACGACCTAGATGGAGT
AGGACAGAGAAATTAACAATTACACAAGCTTAATACACTCCTTAATTGAAGAATCGCAAAACCAGCAAGAAAAGAATGAA
CAAGAATTATTGGAATTAGATAAATGGGCAAGTTTGTGGAATTAATTTAACATAACAAATTGGCTGTGGTATATAAAATT
ATTCATAATGATAGTAGGAGGCTTAGTAAGTTTAAGAATAGTTTTTGCTGTACTTTCTGTAGTGAATAGAGTTAGGCAGG
GATATTCACCATTATCGTTTCAGACCCACCTCCCAATCCCGAGGGGACCCGACAGGCCCGAAGGAATAGAAGAAGAAGGT
GGAGAGAGAGACAGAGACAGATCCATTCGATTAGTGAACGGATCCTTAGCACTTATCTAGGACGATCTGCGGAGCCTGTG
CCTCTTCAGCTACCACCGCTTGAGAGACTTACTCTTGATTGTAACGAGGATTGTGGAACTTCTAGGACGCAGGGGGTAGG
AAGCCCTCAAATATTGGTAGAATCTCCTACAATATTGGAGTCAGGAGCTAAAAAATAGTGCTGTTAGCTTGCTCAATGCC
ACAGCTATAGCAGTAGCTGAGGGGACAGATAGGGTTATAGAAGTAGTACAAGAAGCTTATAGAGCTATTCGCCACATACC
TAGAAGAATAAGACAGGGCTTGGAAAGAATTTTGCTATAAGATAGGTGGCAAGTGGTCAAAAAGTAGTGTGGTTAGATGG
CCTGCTGTAAGGGAAAGAATGAGACGAGCTGAGCCAGCAGCAGATGGGGTGGGAGCAGCATCTCGAGACCTAGAAAAACA
TGGAGCAATCACAAGTAGCAACACAGCAGCTAACAATGCTGCTTGTGCCTGGCTAGAAGCACAAGAGGAGGAGAAGGTAG
GTTTTCCAGTCACACCTCAGGTACCTTTAAGACCAATGACTTACAAGGCAGCTGTAGATCTTAGCCACTTTTTAAAAGAA
AAGGGGGGACTGGAAGGGCTAATTCACTCCCAACGAAGACAAGATATCCTTGATCTGTGGATCTACCACACACAAGGCTA
CTTCCCTGATTGGCAGAACTACACACCAGGACCAGGGATCAGATATCCACTGACCTTTGGATGGCGCTACAAGCTAGTAC
CAGTTGAGCCAGAGAAGTTAGAAGAAGCCAACAAAGGAGAGAACACCAGCTTGTTACACCCTGTGAGCCTGCATAGAATG
GATGACCCGGAGAGAGAAGTGTTAGAGTGGAGGTTTGACAGCCGCCTAGCATTTCATCACGTGGCCCGAGAGCTGCATCC
GGAGTACTTCAAGAACTGCTGATATCGAGCTTGCTACAAGGGACTTTCCGCTAGGGACTTTCCAGGGAGGCGTGGCCTGG
GCGGGACTGGGGAGTGGCGAGCCCTCAGATCCTGCATATAAGCAGCTGCTTTTTGCCTGTACTGGGTCTCTCTGGTTAGA
CCAGATCTGAGCCTAGGAGCTCTCTGGCTAACTAGGGAACCCACTGCTTAAGCCTCAATAAAGCTTGCCTTGAGTGCTTC
AAGTAGTGTGTGCCCATCTGTTGTGTGACTCTGGTAACTAGAGATCCCTCA
>4-59envLTR
--------------------------------------------------------------------------------
--------------------------------------------------------------------------------
--------------------------------------------------------------------------------
--------------------------------------------------------------------------------
--------------------------------------------------------------------------------
--------------------------------------------------------------------------------
--------------------------------------------------------------------------------
--------------------------------------------------------------------------------
--------------------------------------------------------------------------------
--------------------------------------------------------------------------------
--------------------------------------------------------------------------------
--------------------------------------------------------------------------------
--------------------------------------------------------------------------------
--------------------------------------------------------------------------------
--------------------------------------------------------------------------------
--------------------------------------------------------------------------------
--------------------------------------------------------------------------------
--------------------------------------------------------------------------------
--------------------------------------------------------------------------------
--------------------------------------------------------------------------------
--------------------------------------------------------------------------------
--------------------------------------------------------------------------------
--------------------------------------------------------------------------------
--------------------------------------------------------------------------------
--------------------------------------------------------------------------------
--------------------------------------------------------------------------------
--------------------------------------------------------------------------------
--------------------------------------------------------------------------------
--------------------------------------------------------------------------------
--------------------------------------------------------------------------------
--------------------------------------------------------------------------------
--------------------------------------------------------------------------------
--------------------------------------------------------------------------------
--------------------------------------------------------------------------------
--------------------------------------------------------------------------------
```

```
--------------------------------------------------------------------------------
--------------------------------------------------------------------------------
--------------------------------------------------------------------------------
--------------------------------------------------------------------------------
--------------------------------------------------------------------------------
--------------------------------------------------------------------------------
--------------------------------------------------------------------------------
--------------------------------------------------------------------------------
--------------------------------------------------------------------------------
--------------------------------------------------------------------------------
--------------------------------------------------------------------------------
--------------------------------------------------------------------------------
--------------------------------------------------------------------------------
--------------------------------------------------------------------------------
--------------------------------------------------------------------------------
--------------------------------------------------------------------------------
--------------------------------------------------------------------------------
--------------------------------------------------------------------------------
--------------------------------------------------------------------------------
--------------------------------------------------------------------------------
--------------------------------------------------------------------------------
--------------------------------------------------------------------------------
--------------------------------------------------------------------------------
--------------------------------------------------------------------------------
--------------------------------------------------------------------------------
--------------------------------------------------------------------------------
--------------------------------------------------------------------------------
--------------------------------------------------------------------------------
--------------------------------------------------------------------------------
--------------------------------------------------------------------------------
--------------------------------------------------------------------------------
--------------------------------------------------------------------------------
--------------------------------------------------------------------------------
--------------------------------------------------------------------------------
--------------------------------------------------------------------------------
--------------------------------------------------------------------------------
--------------------------------------------------------------------------------
--------------------------------------------------------------------------------
--------------------------------------------------------------------------------
--------------------------------------------------------------------------------
--------------------------------------------------------------------------------
--------------------------------------------------------------------------------
--------------------------------------------------------------------------------
--------------------------------------------------------------------------------
--------------------------------------------------------------------------------
--------------------------------------------------------------------------------
--------------------------------------------------------------------------------
--------------------------------------------------------------------------------
--------------------------------------------------------------------------------
--------------------------------------------------------------------------------
--------------------------------------------------------------------------------
--------------------------------------------------------------------------------
--------------------------------------------------------------------------------
--------------------------------------------------------------------------------
--------------------------------------------------------------------------------
--------------------------------------------------------------------------------
--------------------------------------------------------------------------------
--------------------------------------------------------------------------------
--------------------------------------------------------------------------------
-------------------------------------------AAGCAATGTATGCCCCTCCCATCAGCGGACAAATTAG
ATGTTCATCAAATATTACAGGGCTGCTATTAACAAGAGATGGTAGTAATAACAACAATGGGTCCGAGATCTTCAGACCTG
GAGGAGGAGATATGAAGGACAATTGGAGAAGTGAATTATATAAATATAAAGTAGTAAAAATTGAACCATTAGGAGTAGCA
CCCACCAAGGCAAAGAGAAGAGTGGTGCAGAGAGAAAAAAGAGCAGTAGGAATAGGAGCTTTGTTCCTTAGGTTCTTAGG
AGCAGCAGGAAGCACTATAGGCGCAGCGTCAATGACGCTGACAGTACAGGCCAGACAATTATTGTCTGGTATAGTGCAGC
AGCAGAACAATTTGCTGAGGGCTATTGAGGCGCAACAGCATCTGTTGCAACTCACAGTCTGGGGCATCAAGCAGCTCCAG
GCAAGAATCCTGGCTGTGGAAAGATACCTAAAAGATCAACAGCTCCTGGGGATTTGGGGTTGCTCTGGAAAACTCATTTG
CACCACTGCTGTGCCTTAGAATGCTAGTTGGAGTAATAAATCTCTGGAACAGATTTGGAATCACACGACCTGGATGGAGT
GGGACAGAGAAATTAACAATTACACAAGCTTAATACACTCCTTAATTGAAGAATCGCAAAACCAGCAAGAAAAGAATGAA
CAAGAATTATTGGAATTAGATAAATAGGCAAGTTTGTGGAATTGGTTTAACATAACAAATTGGCTGTGGTATATAAAATT
ATTCATAATGATAGTAGGAGGCTTGGTAGGTTTAAGAATAGTTTTTGCTGTACTTTCTGTAGTGAATAGAGTTAGGCAAG
GATATTCACCATTATCGTTTCAGACCCACCTCCCAATCCCGAGGGGACCCGACAGGCCCGAAGGAATAGAAGAAGAAGGT
GGAGAGAGAGACAGAGACAGATCCATTCGATTAGTGAACGGATCCTTAGCACTTATCTAGGACGATCTGCGGAGCCTGTG
CCTCTTCAGCTACCACCGCTTGAGAGACTTACTCTTGATTGTAACGAGGATTGTGGAACTTCTAGGACGCAGGGGGTGGG
AAGCCCTCAAATATTGGTGGAATCTCCTACAATATTAGAGTCAGGAGCTAAAGAATAGTGCTGTTAGCTTGCTCAATGCC
ACAGCTATAGCAGTAGCTGAAGGGACAGATAGGGTTATAGAAGTAGTACAAGAAGCTTATAGAGCTATTCGCCACATACC
TAGAAGAATAAGACAGGGCTTAGAAAGGATTTTGCTATAAGATAGGTGGCAAGTAGTCAAAAAGTAGTGTGGTTAGATGG
CCTGCTGTAAAGGAAAGAATGAGACGAGCTGAGCCAGCAGCAGATGGAGTAGGAGCAGCATCTCGAGACCTAGAAAAACA
TGGAGCAATCACAAGTAGCAACACAGCAGCTAACAATGCTGCTTGTGCCTGGCTAGAAGCACAAGAGGAGGAGAAGGTAG
GTTTTCCAGTCACACCTCAGGTACCTTTAAGACCAATGACTTACAAGGCAGCTGTAGATCTTAGCCACTTTTTAAAAGAA
AAGGGGGGACTGGAAGGGCTAATTCACTCCCAACGAAGACAAGATATCCTTGATCTGTGGATCTACCACACACAAGGCTA
CTTCCCTGATTGGCAGAACTACACACCAGGACCAGGGATCAGATATCCACTGACCTTTGGATGGCGCTACAAGCTAGTAC
CAGTTGAGCCAGAGAAGTTAGAAGAAGCCAACAAAGGAGAGAACACCAGCTTGTTACACCCTGTGAGCCTGCATGGAATG
GATGACCCGGAGAGAGAAGTGTTAGAGTGGAGGTTTGACAGCCGCCTAGCATTTCATCACGTGGCCCGAGAGCTGCATCC
GGAGTACTTCAAGAACTGCTGATATCGAGCTTGCTACAAGGGACTTTCCGCTGGGGACTTTCCAGGGAGGCGTGGCCTGG
GCGGGACTGGGGAGTGGCGAGCCCTCAGATCCTGCATATAAGCAGCTGCTTTTTGCCTGTACTGGGTCTCTCTGGTTAGA
CCAGATCTGAGCCTGGGAGCTCTCTGGCTAACTAGGGAACCCACTGCTTAAGCCTCAATAAAGCTTGCCTTGAGTGCTTC
AAGTAGTGTGTGCCCATCTGTTGTGTGACTCTGGTAACTAGAGATCCCTCA
>4-60envLTR
--------------------------------------------------------------------------------
--------------------------------------------------------------------------------
--------------------------------------------------------------------------------
--------------------------------------------------------------------------------
--------------------------------------------------------------------------------
--------------------------------------------------------------------------------
```


--GGTGGTAATAACAACAATGGGTCCGAGATCTTCAGACCTG
GAGGAGGAGATATGAGGGACAATTGGAGAAGTGAATTATATAAATATAAAGTAGTAAAAATTGAACCATTAGGAGTAGCA
CCCACCAAGGCAAAGAGAAGAGTGGTGCAGAGAGAAAAAAGAGCAGTGGGAATAGGAGCTTTGTTCCTTGGGTTCTTGGG
AGCAGCAGGAAGCACTATGGGCGCAGCGTCAATGACGCTGACGGTACAGGCCAGACAATTATTGTCTGGTATAGTGCAGC
AGCAGAACAATTTGCTGAGGGCTATTGAGGCGCAACAGCATCTGTTGCAACTCACAGTCTGGGGCATCAAGCAGCTCCAG
GCAAGAATCCTGGCTGTGGAAAGATACCTAAAGGATCAACAGCTCCTGGGGATTTGGGGTTGCTCTGGAAAACTCATTTG

```
CACCACTGCTGTGCCTTGGAATGCTAGTTGGAGTAATAAATCTCTGGAACAGATTTGGAATCACACGACCTGGATGGAGT
GGGACAGAGAAATTAACAATTACACAAGCTTAATACACTCCTTAATTGAAGAATCGCAAAACCAGCAAGAAAAGAATGAA
CAAGAATTATTGGAATTAGATAAATGGGCAAGTTTGTGGAATTGGTTTAACATAACAAATTGGCTGTGGTATATAAAATT
ATTCATAATGATAGTAGGAGGCTTGGTAGGTTTAAGAATAGTTTTTGCTGTACTTTCTGTAGTGAATAGAGTTAGGCAGG
GATATTCACCATTATCGTTTCAGACCCACCTCCCAATCCCGAGGGGACCCGACAGGCCCGAAGGAATAGAAGAAGAAGGT
GGAGAGAGAGACAGAGACAGATCCATTCGATTAGTGAACGGATCCTTAGCACTTATCTGGGACGATCTGCGGAGCCTGTG
CCTCTTCAGCTACCACCGCTTGAGAGACTTACTCTTGATTGTAACGAGGATTGTGGAACTTCTGGGACGCAGGGGGTGGG
AAGCCCTCAAATATTGGTGGAATCTCCTACAATATTGGAGTCAGGAGCTAAAGAATAGTGCTGTTAGCTTGCTCAATGCC
ACAGCTATAGCAGTAGCTGAGGGGACAGATAGGGTTATAGAAGTAGTACAAGAAGCTTATAGAGCTATTCGCCACATACC
TAGAAGAATAAGACAGGGCTTGGAAAGGATTTTGCTATAAGATGGGTGGCAAGTGGTCAAAAAGTAGTGTGGTTGGATGG
CCTGCTGTAAGGGAAAGAATGAGACGAGCTGAGCCAGCAGCAGATGGGGTGGGAGCAGCATCTCGAGACCTAGAAAAACA
TGGAGCAATCACAAGTAGCAACACAGCAGCTAACAATGCTGCTTGTGCCTGGCTAGAAGCACAAGAGGAGGAGAAGGTGG
GTTTTCCAGTCACACCTCAGGTACCTTTAAGACCAATGACTTACAAGGCAGCTGTAGATCTTAGCCACTTTTTAAAAGAA
AAGGGGGGACTGGAAGGGCTAATTCACTCCCAACGAAGACAAGATATCCTTGATCTGTGGATCTACCACACACAAGGCTA
CTTCCCTGATTGGCAGAACTACACACCAGGACCAGGGATCAGATATCCACTGACCTTTGGATGGCGCTACAAGCTAGTAC
CAGTTGAGCCAGAGAAGTTAGAAGAAGCCAACAAAGGAGAGAACACCAGCTTGTTACACCCTGTGAGCCTGCATGGAATG
GATGACCCGGAGAGAGAAGTGTTAGAGTGGAGGTTTGACAGCCGCCTAGCATTTCATCACGTGGCCCGAGAGCTGCATCC
GGAGTACTTCAAGAACTGCTGATATCGAGCTTGCTACAAGGGACTTTCCGCTGGGGACTTTCCAGGGAGGCGTGGCCTGG
GCGGGACTGGGGAGTGGCGAGCCCTCAGATCCTGCATATAAGCAGCTGCTTTTTGCCTGTACTGGGTCTCTCTGGTTAGA
CCAGATCTGAGCCTGGGAGCTCTCTGGCTAACTAGGGAACCCACTGCTTAAGCCTCAATAAAGCTTGCCTTGAGTGCTTC
AAGTAGTGTGTGCCCATCTGTTGTGTGACTCTGGTAACTAGAGATCCCTCA
>4-81envLTR
--------------------------------------------------------------------------------
--------------------------------------------------------------------------------
--------------------------------------------------------------------------------
--------------------------------------------------------------------------------
--------------------------------------------------------------------------------
--------------------------------------------------------------------------------
--------------------------------------------------------------------------------
--------------------------------------------------------------------------------
--------------------------------------------------------------------------------
--------------------------------------------------------------------------------
--------------------------------------------------------------------------------
--------------------------------------------------------------------------------
--------------------------------------------------------------------------------
--------------------------------------------------------------------------------
--------------------------------------------------------------------------------
--------------------------------------------------------------------------------
--------------------------------------------------------------------------------
--------------------------------------------------------------------------------
--------------------------------------------------------------------------------
--------------------------------------------------------------------------------
--------------------------------------------------------------------------------
--------------------------------------------------------------------------------
--------------------------------------------------------------------------------
--------------------------------------------------------------------------------
--------------------------------------------------------------------------------
--------------------------------------------------------------------------------
--------------------------------------------------------------------------------
--------------------------------------------------------------------------------
--------------------------------------------------------------------------------
--------------------------------------------------------------------------------
--------------------------------------------------------------------------------
--------------------------------------------------------------------------------
--------------------------------------------------------------------------------
--------------------------------------------------------------------------------
--------------------------------------------------------------------------------
--------------------------------------------------------------------------------
--------------------------------------------------------------------------------
--------------------------------------------------------------------------------
--------------------------------------------------------------------------------
--------------------------------------------------------------------------------
--------------------------------------------------------------------------------
--------------------------------------------------------------------------------
--------------------------------------------------------------------------------
--------------------------------------------------------------------------------
--------------------------------------------------------------------------------
--------------------------------------------------------------------------------
--------------------------------------------------------------------------------
--------------------------------------------------------------------------------
--------------------------------------------------------------------------------
--------------------------------------------------------------------------------
--------------------------------------------------------------------------------
--------------------------------------------------------------------------------
--------------------------------------------------------------------------------
--------------------------------------------------------------------------------
--------------------------------------------------------------------------------
--------------------------------------------------------------------------------
--------------------------------------------------------------------------------
--------------------------------------------------------------------------------
--------------------------------------------------------------------------------
--------------------------------------------------------------------------------
--------------------------------------------------------------------------------
--------------------------------------------------------------------------------
--------------------------------------------------------------------------------
--------------------------------------------------------------------------------
--------------------------------------------------------------------------------
--------------------------------------------------------------------------------
--------------------------------------------------------------------------------
--------------------------------------------------------------------------------
--------------------------------------------------------------------------------
--------------------------------------------------------------------------------
--------------------------------------------------------------------------------
--------------------------------------------------------------------------------
```


-----------------------------TAACAAGAGATGGTGGTAATAACAACAATGGGTCCGAGATCTTCAGACCTA
GAGGAGGAGATATGAGGGACAATTGGAGAAGTGAATTATATAAATATAAAGTAGTAAAAATTGAACCATTAGGAGTAGCA
CCCACCAAGGCAAAGAGAAGAGTGGTGCAGAGAGAAAAAAGAGCAGTAGGAATAGGAGCTTTGTTCCTTAGGTTCTTAGA
AGCAGCAGGAAGCACTATGGGCGCAGCGTCAATGACGCTGACGGTACAGGCCAGACAATTATTGTCTGGTATAGTGCAGC
AGCAGAACAATTTGCTGAGGGCTATTGAGGCGCAACAGCATCTGTTGCAACTCACAGTCTGGGGCATCAAGCAGCTCCAG
GCAAGAATCCTGGCTGTGGAAAGATACCTAAAGGATCAACAGCTCCTGAAGATTTGGGGTTGCTCTGGAAAACTCATTTG
CACCACTGCTGTGCCTTAGAATGCTAGTTGGAGTAATAAATCTCTGGAACAGATTTGGAATCACACGACCTGGATGGAGT
GGGACAGAGAAATTAACAATTACACAAGCTTAATACACTCCTTAATTGAAGAATCGCAAAACCAGCAAGAAAAGAATGAA
CAAGAATTATTGGAATTAGATAAATGGGCAAGTTTGTGGAATTAGTTTAACATAACAAATTGGCTGTAGTATATAAAATT
ATTCATAATGATAGTAGGAGGCTTGGTAGGTTTAAGAATAGTTTTTGCTGTACTTTCTGTAGTGAATAGAGTTAGGCAGG
GATATTCACCATTATCGTTTCAGACCCACCTCCCAATCCCGAGGGGACCCGACAGGCCCGAAGGAATAGAAGAAGAAGGT
GGAGAGAGAGACAGAGACAGATCCATTCGATTAGTGAACGGATCCTTAGCACTTATCTGGGACGATCTGCGGAGCCTGTG
CCTCTTCAGCTACCACCGCTTGAGAGACTTACTCTTGATTGTAACGAGGATTGTGGAACTTCTAGGACGCAGGGAGTAGG
AAGCCCTCAAATATTGGTGGAATCTCCTACAATATTGGAGTCAGGAGCTAAAGAATAGTGCTGTTAGCTTGCTCAATGCC
ACAGCTATAGCAGTAGCTGAGGGGACAGATAAGGTTATAGAAGTAGTACAAGAAGCTTATAGAGCTATTCGCCACATACC
TAGAAGAATAAGACAGGGCTTAGAAAGGATTTTGCTATAAGATAGGTGGCAAATGGTCAAAAAGTAGTGTGGTTGGATGG
CCTGCTGTAAGGGAAAGAATGAGACGAGCTGAGCCAGCAGCAGATGGGGTAGGAGCAGCATCTCGAGACCTAGAAAAACA
TGGAGCAATCACAAGTAGCAACACAGCAGCTAACAATGCTGCTTGTGCCTGGCTAGAAGCACAAGAGGAGGAGAAGGTAG
GTTTTCCAGTCACACCTCAGGTACCTTTAAGACCAATGACTTACAAGGCAGCTGTAGATCTTAGCCACTTTTTAAAAGAA
AAGGGGGGACTGGAAGGGCTAATTCACTCCCAACGAAGACAAGATATCCTTGATCTGTGGATCTACCACACACAAGGCTA
CTTCCCTGATTGGCAGAACTACACACCAGGACCAGGGATCAGATATCCACTGACCTTTGGATGGCGCTACAAGCTAGTAC
CAGTTGAGCCAGAGAAGTTAGAAGAAGCCAACAAAGGAGAGAACACCAGCTTGTTACACCCTGTGAGCCTGCATGGAATG
GATGACCCGGAGAGAGAAGTGTTAGAGTGGAGGTTTGACAGCCGCCTAGCATTTCATCACGTGGCCCGAGAGCTGCATCC
GGAGTACTTCAAGAACTGCTGATATCGAGCTTGCTACAAGGGACTTTCCGCTGGGGACTTTCCAGGGAGGCGTGGCCTGG
GCGGGACTGGGGAGTGGCGAGCCCTCAGATCCTGCATATAAGCAGCTGCTTTTTGCCTGTACTGGGTCTCTCTGGTTAGA
CCAGATCTGAGCCTAGGAGCTCTCTGGCTAACTAAGGAACCCACTGCTTAAGCCTCAATAAAGCTTGCCTTGAGTGCTTC
AAGTAGTGTGTGCCCATCTGTTGTGTGACTCTAGTAACTAGAGATCCCTCA
>4-86envLTR

-------------------------------ACAAGAGATGGTGGTAATAACAACAATGGGTCCGAGATCTTCAGACCTG
GAGGAGGAGATATGAGGGACAATTGGAGAAGTGAATTATATAAATATAAAGTAGTAAAAATTGAACCATTAGGAGTAGCA
CCCACCAAGGCAAAGAGAAGAGTGGTGCAGAGAGAAAAAAGAGCAGTGGGAATAGGAGCTTTGTTCCTTGGGTTCTTGGG
AGCAGCAGGAAGCACTATGGGCGCAGCGTCAATGACGCTGACGGTACAGGCCAGACAATTATTGTCTGGTATAGTGCAGC
AGCAGAACAATTTGCTGAGGGCTATTGAGGCGCAACAGCATCTGTTGCAACTCACAGTCTGGGGCATCAAGCAGCTCCAG
GCAAGAATCCTGGCTGTGGAAAGATACCTAAAGGATCAACAGCTCCTGGGGATTTGGGGTTGCTCTGGAAAACTCATTTG
CACCACTGCTGTGCCTTGGAATGCTAGTTGGAGTAATAAATCTCTGGAACAGATTTGGAATCACACGACCTGGATGGAGT
GGGACAGAGAAATTAACAATTACACAAGCTTAATACACTCCTTAATTGAAGAATCGCAAAACCAGCAAGAAAAGAATGAA
CAAGAATTATTGGAATTAGATAAATGGGCAAGTTTGTGGAATTGGTTTAACATAACAAATTGGCTGTGGTATATAAAATT
ATTCATAATGATAGTAGGAGGCTTGGTAGGTTTAAGAATAGTTTTTGCTGTACTTTCTGTAGTGAATAGAGTTAGGCAGG
GATATTCACCATTATCGTTTCAGACCCACCTCCCAATCCCGAGGGGACCCGACAGGCCCGAAGGAATAGAAGAAGAAGGT
GGAGAGAGAGACAGAGACAGATCCATTCGATTAGTGAACGGATCCTTAGCACTTATCTGGGACGATCTGCGGAGCCTGTG
CCTCTTCAGCTACCACCGCTTGAGAGACTTACTCTTGATTGTAACGAGGATTGTGGAACTTCTGGGACGCAGGGGGTGGG
AAGCCCTCAAATATTGGTGGAATCTCCTACAATATTGGAGTCAGGAGCTAAAGAATAGTGCTGTTAGCTTGCTCAATGCC
ACAGCTATAGCAGTAGCTGAGGGGACAGATAGGGTTATAGAAGTAGTACAAGAAGCTTATAGAGCTATTCGCCACATACC
TAGAAGAATAAGACAGGGCTTGGAAAGGATTTTGCTATAAGATGGGTGGCAAGTGGTCAAAAAGTAGTGTGGTTGGATGG
CCTGCTGTAAGGGAAAGAATGAGACGAGCTGAGCCAGCAGCAGATGGGGTGGGAGCAGCATCTCGAGACCTAGAAAAACA
TGGAGCAATCACAAGTAGCAACACAGCAGCTAACAATGCTGCTTGTGCCTGGCTAGAAGCACAAGAGGAGGAGAAGGTGG
GTTTTCCAGTCACACCTCAGGTACCTTTAAGACCAATGACTTACAAGGCAGCTGTAGATCTTAGCCACTTTTTAAAAGAA
AAGGGGGGACTGGAAGGGCTAATTCACTCCCAACGAAGACAAGATATCCTTGATCTGTGGATCTACCACACACAAGGCTA
CTTCCCTGATTGGCAGAACTACACACCAGGACCAGGGATCAGATATCCACTGACCTTTGGATGGCGCTACAAGCTAGTAC
CAGTTGAGCCAGAGAAGTTAGAAGAAGCCAACAAAGGAGAGAACACCAGCTTGTTACACCCTGTGAGCCTGCATGGAATG
GATGACCCGGAGAGAGAAGTGTTAGAGTGGAGGTTTGACAGCCGCCTAGCATTTCATCACGTGGCCCGAGAGCTGCATCC
GGAGTACTTCAAGAACTGCTGATATCGAGCTTGCTACAAGGGACTTTCCGCTGGGGACTTTCCAGGGAGGCGTGGCCTGG
GCGGGACTGGGGAGTGGCGAGCCCTCAGATCCTGCATATAAGCAGCTGCTTTTTGCCTGTACTGGGTCTCTCTGGTTAGA
CCAGATCTGAGCCTGGGAGCTCTCTGGCTAACTAGGGAACCCACTGCTTAAGCCTCAATAAAGCTTGCCTTGAGTGCTTC
AAGTAGTGTGTGCCCATCTGTTGTGTGACTCTGGTAACTAGAGATCCCTCA

>4-87envLTR

--AAAGCAATGTATGCCCCTCCCATCAGCGGACAAATTAG
ATGTTCATCAAATATTACAGGGCTGCTATTAACAAGAGATGGTGGTAATAACAACAATAGGTCCGAGATCTTCAGACCTG
GAGGAGGAGATATGAAGGACAATTGGAGAAGTGAATTATATAAATATAAAGTAGTAAAAATTGAACCATTAGGAGTAGCA
CCCACCAAGGCAAAGAGAAGAGTGGTGCAGAGAGAAAAAAGAGCAGTAGGAATAGGAGCTTTGTTCCTTAGGTTCTTAGG
AGCAGCAGGAAGCACTATAGGCGCAGCGTCAATGACGCTGACGGTACAGGCCAGACAATTATTGTCTGGTATAGTGCAGC
AGCAGAACAATTTGCTGAGGGCTATTGAGGCGCAACAGCATCTGTTGCAACTCACAGTCTGAGGCATCAAGCAGCTCCAG
GCAAGAATCCTGGCTGTGGAAAGATACCTAAAGGATCAACAGCTCCTGGGGATTTGGGGTTGCTCTAGAAAACTCATTTG
CACCACTGCTGTGCCTTAGAATGCTAGTTAGAGTAATAAATCTCTGGAACAGATTTGGAATCACACGACCTGGATGGAGT
GGGACAGAGAAATTAACAATTACACAAGCTTAATACACTCCTTAATTGAAGAATCGCAAAACCAGCAAGAAAAGAATGAA
CAAGAATTATTAGAATTAGATAAATGGGCAAGTTTGTGGAATTGGTTTAACATAACAAATTGGCTGTGGTATATAAAATT
ATTCATAATGATAGTAGGAGGCTTAGTAAGTTTAAGAATAGTTTTTGCTGTACTTTCTGTAGTGAATAGAGTTAGGCAGG
GATATTCACCATTATCGTTTCAGACCCACCTCCCAATCCCGAGAGGACCCGACAGGCCCGAAGGAATAGAAGAAGAAGGT
GGAGAAAGAGACAGAGACAGATCCATTCGATTAGTGAACAGATCCTTAGCACTTATCTAGGACGATCTGCGGAGCCTGTG
CCTCTTCAGCTACCACCGCTTGAGAGACTTACTCTTGATTGTAACGAGGATTGTGGAACTTCTAGGACGCAGGAGGTAGG
AAGCCCTCAAATATTGGTGGAATCTCCTACAATATTGGAGTCAGGAGCTAAAGAATAGTGCTGTTAGCTTGCTCAATGCC
ACAGCTATAGCAGTAGCTGAGAGGACAGATAAGGTTATAGAAGTAGTACAAGAAGCTTATAGAGCTATTCGCCACATACC
TAGAAGAATAAGACAGGGCTTAGAAAGGATTTTGCTATAAGATGGGTGGCAAGTGGTCAAAAAGTAGTGTGGTTAGATGG

CCTGCTGTAAGGGAAAGAATGAGACGAGCTGAGCCAGCAGCAGATGAGGTAGGAGCAGCATCTCGAGACCTAGAAAAACA
TGGAGCAATCACAAGTAGCAACACAGCAGCTAACAATGCTGCTTGTGCCTGGCTAGAAGCACAAGAGGAGGAGAAAGTAG
GTTTTCCAGTCACACCTCAGGTACCTTTAAGACCAATGACTTACAAGGCAGCTGTAGATCTTAGCCACTTTTTAAAAGAA
AAGGGGGGACTGGAAGGGCTAATTCACTCCCAACGAAGACAAGATATCCTTGATCTGTGGATCTACCACACACAAGGCTA
CTTCCCTGATTGGCAGAACTACACACCAGGACCAGGGATCAGATATCCACTGACCTTTGGATGGCGCTACAAGCTAGTAC
CAGTTGAGCCAGAGAAGTTAGAAGAAGCCAACAAAGGAGAGAACACCAGCTTGTTACACCCTGTGAGCCTGCATGGAATG
GATGACCCGGAGAGAGAAGTGTTAGAGTGGAGGTTTGACAGCCGCCTAGCATTTCATCACGTGGCCCGAGAGCTGCATCC
GGAGTACTTCAAGAACTGCTGATATCGAGCTTGCTACAAGGGACTTTCCGCTGGGGACTTTCCAGGGAGGCGTGGCCTAG
GCGGGACTAAGGAGTGGCGAGCCCTCAGATCCTGCATATAAGCAGCTGCTTTTTGCCTGTACTAGGTCTCTCTGGTTAGA
CCAGATCTGAGCCTGGGAGCTCTCTGGCTAACTAGGGAACCCACTGCTTAAGCCTCAATAAAGCTTGCCTTGAGTGCTTC
AAGTAGTGTGTGCCCATCTGTTGTGTGACTCTAGTAACTAGAGATCCCTCA

>4-92envLTR

---G
GAGGAGGAGATATGAAGGACAATTGGAGAAGTGAATTATATAAATATAAAGTAGTAAAAATTGAACCATTAGGAGTAGCA
CCCACCAAGGCAAAGAGAAGAGTGGTGCAGAGAGAAAAAAGAGCAGTAAGAATAGGAGCTTTGTTCCTTAGGTTCTTAGG
AGCAGCAGGAAGCACTATGGGCGCAGCGTCAATGACGCTGACGGTACAGGCCAGACAATTATTGTCTGGTATAGTGCAGC
AGCAGAACAATTTGCTGAGGGCTATTGAGGCGCAACAGCATCTGTTGCAACTCACAGTCTGGGGCATCAAGCAGCTCCAG
GCAAGAATCCTGGCTGTGGAAAGATACCTAAAAGATCAACAGCTCCTGGGGATTTGGGGTTGCTCTGGAAAACTCATTTG
CACCACTGCTGTGCCTTGGAATGCTAGTTAGAGTAATAAATCTCTGGAACAGATTTAGAATCACACGACCTGGATGGAGT
GGGACAGAGAAATTAACAATTACACAAGCTTAATACACTCCTTAATTGAAGAATCGCAAAACCAGCAAGAAAAGAATGAA
CAAGAATTATTGGAATTAGATAAATAGGCAAGTTTGTGGAATTGGTTTAACATAACAAATTGGCTGTGGTATATAAAATT
ATTCATAATGATAGTAGGAGGCTTGGTAAGTTTAAGAATAGTTTTTGCTGTACTTTCTGTAGTGAATAGAGTTAGGCAAA
GATATTCACCATTATCGTTTCAGACCCACCTCCCAATCCCGAGGGGACCCGACAGGCCCGAAAGAATAGAAGAAGAAGGT
GGAGAGAGAGACAGAGACAGATCCATTCGATTAGTGAACGGATCCTTAGCACTTATCTAGGACGATCTGCGGAGCCTGTG
CCTCTTCAGCTACCACCGCTTGAGAGACTTACTCTTGATTGTAACGAGGATTGTGGAACTTCTAGGACGCAGGGGGTGGG
AAGCCCTCAAATATTGGTGGAATCTCCTACAATATTGGAGTCAGGAGCTAAAGAATAGTGCTGTTAGCTTGCTCAATGCC
ACAGCTATAGCAGTAGCTGAGGGGACAGATAAGGTTATAGAAGTAGTACAAGAAGCTTATAGAGCTATTCGCCACATACC
TAGAAGAATAAGACAGGGCTTAGAAAAGATTTTGCTATAAGATAGGTGGCAAGTGGTCAAAAAGTAGTGTGGTTAGATGG
CCTGCTGTAAGGGAAAGAATGAGACGAGCTGAGCCAGCAGCAGATGAGGTGGGAGCAGCATCTCGAGACCTAGAAAAACA
TGGAGCAATCACAAGTAGCAACACAGCAGCTAACAATGCTGCTTGTGCCTGGCTAGAAGCACAAGAGGAGGAGAAGGTAG
GTTTTCCAGTCACACCTCAGGTACCTTTAAGACCAATGACTTACAAGGCAGCTGTAGATCTTAGCCACTTTTTAAAAGAA
AAGGGGGGACTGGAAGGGCTAATTCACTCCCAACGAAGACAAGATATCCTTGATCTGTGGATCTACCACACACAAGGCTA
CTTCCCTGATTGGCAGAACTACACACCAGGACCAGGGATCAGATATCCACTGACCTTTGGATGGCGCTACAAGCTAGTAC
CAGTTGAGCCAGAGAAGTTAGAAGAAGCCAACAAAGGAGAGAACACCAGCTTGTTACACCCTGTGAGCCTGCATGGAATG
GATGACCCGGAGAGAGAAGTGTTAGAGTGGAGGTTTGACAGCCGCCTAGCATTTCATCACGTGGCCCGAGAGCTGCATCC
GGAGTACTTCAAGAACTGCTGATATCGAGCTTGCTACAAGGGACTTTCCGCTGGGGACTTTCCAGGGAGGCGTGGCCTGG
GCAGGACTGGGGAGTGGCGAGCCCTCAGATCCTGCATATAAGCAGCTGCTTTTTGCCTGTACTGGGTCTCTCTGGTTAGA
CCAGATCTGAGCCTGGGAGCTCTCTGGCTAACTAGGGAACCCACTGCTTAAGCCTCAATAAAGCTTGCCTTGAGTGCTTC
AAGTAGTGTGTGCCCATCTGTTGTGTGACTCTGGTAACTAGAGATCCCTCA

>4-94envLTR


```
--------------------------------------------------------------------------------
--------------------------------------------------------------------------------
--------------------------------------------------------------------------------
--------------------------------------------------------------------------------
--------------------------------------------------------------------------------
--------------------------------------------------------------------------------
--------------------------------------------------------------------------------
--------------------------------------------------------------------------------
--------------------------------------------------------------------------------
--------------------------------------------------------------------------------
--------------------------------------------------------------------------------
--------------------------------------------------------------------------------
--------------------------------------------------------------------------------
--------------------------------------------------------------------------------
--------------------------------------------------------------------------------
--------------------------------------------------------------------------------
--------------------------------------------------------------------------------
--------------------------------------------------------------------------------
--------------------------------------------------------------------------------
--------------------------------------------------------------------------------
--------------------------------------------------------------------------------
--------------------------------------------------------------------------------
--------------------------------------------------------------------------------
--------------------------------------------------------------------------------
--------------------------------------------------------------------------------
--------------------------------------------------------------------------------
--------------------------------------------------------------------------------
--------------------------------------------------------------------------------
--------------------------------------------------------------------------------
--------------------------------------------------------------------------------
--------------------------------------------------------------------------------
--------------------------------------------------------------------------------
--------------------------------------------------------------------------------
--------------------------------------------------------------------------------
--------------------------------------------------------------------------------
--------------------------------------------------------------------------------
--------------------------------------------------------------------------------
--------------------------------------------------------------------------------
--------------------------------------------------------------------------------
--------------------------------------------------------------------------------
--------------------------------------------------------------------------------
---------------------------------------------------------------------TAGGAGTAGCA
CCCACCAAGGCNAANAGAAGAGNGGTGCAGAGAGAAAAAAGAGCAGTAGGAATAGGAGCTTTGTTCCTTAGGTTCTTGGG
AGCAGCAGGAAGCACTATGGGCGCAGCGTCAATGACGCTGACGGTACAGGCCAGACAATTATTGTCTGGTATAGTGCAGC
AGCAGAACAATTTGCTGAGGGCTATTGAGGCGCAACAGCATCTGTTGCAACTCACAGTCTGGGGCATCAAGCAGCTCCAG
GCAAGAATCCTGGCTGTGGAAAGATACCTAAAGGATCAACAGCTCCTGGGGATTTGGGGTTGCTCTGGAAAACTCATTTG
CACCACTGCTGTGCCTTGGAATGCTAGTTGGAGTAATAAATCTCTGGAACAGATTTGGAATCACACGACCTGGATGGAGT
GGGACAGAGAAATTAACAATTACACAAGCTTAATACACTCCTTAATTGAAGAATCGCAAAACCAGCAAGAAAAGAATGAA
CAAGAATTATTGGAATTAGATAAATGGGCAAGTTTGTGGAATTGGTTTAACATAACAAATTGGCTGTGGTATATAAAATT
ATTCATAATGATAGTAGGAGGCTTGGTAGGTTTAAGAATAGTTTTTGCTGTACTTTCTGTAGTGAATAGAGTTAGGCAGG
GATATTCACCATTATCGTTTCAGACCCACCTCCCAATCCCGAGGGGACCCGACAGGCCCGAAGGAATAGAAGAAGAAGGT
GGAGAGAGAGACAGAGACAGATCCATTCGATTAGTGAACGGATCCTTAGCACTTATCTGGGACGATCTGCGGAGCCTGTG
CCTCTTCAGCTACCACCGCTTGAGAGACTTACTCTTGATTGTAACGAGGATTGTGGAACTTCTGGGACGCAGGGGGTGGG
AAGCCCTCAAATATTGGTGGAATCTCCTACAATATTGGAGTCAGGAGCTAAAGAATAGTGCTGTTAGCTTGCTCAATGCC
ACAGCTATAGCAGTAGCTGAGGGGACAGATAAGGTTATAGAAGTAGTACAAGAAGCTTATAGAGCTATTCGCCACATACC
TAGAAGAATAAGACAGGGCTTGGAAAGGATTTTGCTATAAGATAGGTGGCAAGTGGTCAAAAAGTAGTGTGGTTGGATGG
CCTGCTGTAAGGGAAAGAATGAGACGAGCTGAGCCAGCAGCAGATGGGGTGGGAGCAGCATCTCGAGACCTAGAAAAACA
TGGAGCAATCACAAGTAGCAACACAGCAGCTAACAATGCTGCTTGTGCCTGGCTAGAAGCACAAGAGGAGGAGAAGGTGG
GTTTTCCAGTCACACCTCAGGTACCTTTAAGACCAATGACTTACAAGGCAGCTGTAGATCTTAGCCACTTTTTAAAAGAA
AAGGGGGGACTGGAAGGGCTAATTCACTCCCAACGAAGACAAGATATCCTTGATCTGTGGATCTACCACACACAAGGCTA
CTTCCCTGATTGGCAGAACTACACACCAGGACCAGGGATCAGATATCCACTGACCTTTGGATGGCGCTACAAGCTAGTAC
CAGTTGAGCCAGAGAAGTTAGAAGAAGCCAACAAAGGAGAGAACACCAGCTTGTTACACCCTGTGAGCCTGCATAGAATG
GATGACCCGGAGAGAGAAGTGTTAGAGTGGAGGTTTGACAGCCGCCTAGCATTTCATCACGTGGCCCGAGAGCTGCATCC
GGAGTACTTCAAGAACTGCTGATATCGAGCTTGCTACAAAGGACTTTCCGCTGGGGACTTTCCAGGGAGGCGTGGCCTAG
GCGGGACTGGGGAGTGGCGAGCCCTCAGATCCTGCATATAAGCAGCTGCTTTTTGCCTGTACTAGGTCTCTCTGGTTAGA
CCAGATCTGAGCCTAGGAGCTCTCTGGCTAACTAGGGAACCCACTGCTTAAGCCTCAATAAAGCTTGCCTTAAGTGCTTC
AAGTAGTGTGTGCCCATCTGTTGTGTGACTCTGGTAACTAGAGATCCCTCA
>4-98envLTR
--------------------------------------------------------------------------------
--------------------------------------------------------------------------------
--------------------------------------------------------------------------------
--------------------------------------------------------------------------------
--------------------------------------------------------------------------------
--------------------------------------------------------------------------------
--------------------------------------------------------------------------------
--------------------------------------------------------------------------------
--------------------------------------------------------------------------------
--------------------------------------------------------------------------------
--------------------------------------------------------------------------------
--------------------------------------------------------------------------------
--------------------------------------------------------------------------------
--------------------------------------------------------------------------------
--------------------------------------------------------------------------------
--------------------------------------------------------------------------------
--------------------------------------------------------------------------------
--------------------------------------------------------------------------------
--------------------------------------------------------------------------------
--------------------------------------------------------------------------------
--------------------------------------------------------------------------------
--------------------------------------------------------------------------------
--------------------------------------------------------------------------------
--------------------------------------------------------------------------------
--------------------------------------------------------------------------------
--------------------------------------------------------------------------------
```

```
--------------------------------------------------------------------------------
--------------------------------------------------------------------------------
--------------------------------------------------------------------------------
--------------------------------------------------------------------------------
--------------------------------------------------------------------------------
--------------------------------------------------------------------------------
--------------------------------------------------------------------------------
--------------------------------------------------------------------------------
--------------------------------------------------------------------------------
--------------------------------------------------------------------------------
--------------------------------------------------------------------------------
--------------------------------------------------------------------------------
--------------------------------------------------------------------------------
--------------------------------------------------------------------------------
--------------------------------------------------------------------------------
--------------------------------------------------------------------------------
--------------------------------------------------------------------------------
--------------------------------------------------------------------------------
--------------------------------------------------------------------------------
--------------------------------------------------------------------------------
--------------------------------------------------------------------------------
--------------------------------------------------------------------------------
--------------------------------------------------------------------------------
--------------------------------------------------------------------------------
--------------------------------------------------------------------------------
--------------------------------------------------------------------------------
--------------------------------------------------------------------------------
--------------------------------------------------------------------------------
--------------------------------------------------------------------------------
--------------------------------------------------------------------------------
--------------------------------------------------------------------------------
--------------------------------------------------------------------------------
--------------------------------------------------------------------------------
--------------------------------------------------------------------------------
--------------------------------------------------------------------------------
--------------------------------------------------------------------------------
--------------------------------------------------------------------------------
--------------------------------------------------------------------------------
--------------------------------------------------------------------------------
--------------------------------------------------------------------------------
--------------------------------------------------------------------------------
--------------------------------------------------------------------------------
--------------------------------------------------------------------------------
--------------------------------------------------------------------------------
--------------------------------------------------------------------------------
--------------------------------------------------------------------------------
--------------------------------------------------------------------------------
--------------------------------------------------------------------------------
--------------------------------------------------------------------------------
--------------------------------------------------------------------------------
--------------------------------------------------------------------------------
--------------------------------------------------------------------------------
--------------------------------------------------------------------------------
--------------------------------------------------------------------------------
--------------------------------------------------------------------------------
--------------------------------------------------------------------------------
--------------------------------------------------------------------------------
--------------------------------------------------------------------------------
--------------------------------------------------------------------------------
--------------------------------------------------------------------------------
--------------------------------------------------------------------------------
--------------------------------------------------------------------------------
--------------------------------------------------------------------------------
--------------------------------------------------------------------------------
--------------------------------------------------------------------------------
--------------------------------------------------------------------------------
--------------------------------------------------------------------------------
--------------------------------------------------------------------------------
---------------------------------------------------------------------------ACCTG
GAGGAGGAGATATGAGGGACAATTGGAGAAGTGAATTATATAAATATAAAGTAGTAAAAATTGAACCATTAGGAGTAGCA
CCCACCAAGGCAAAGAGAAGAGTGGTGCAGAGAGAAAAAAGAGCAGTGGGAATAGGAGCTTTGTTCCTTGGGTTCTTGGG
AGCAGCAGGAAGCACTATGGGCGCAGCGTCAATGACGCTGACGGTACAGGCCAGACAATTATTGTCTGGTATAGTGCAGC
AGCAGAACAATTTGCTGAGGGCTATTGAGGCGCAACAGCATCTGTTGCAACTCACAGTCTGGGGCATCAAGCAGCTCCAG
GCAAGAATCCTGGCTGTGGAAAGATACCTAAAGGATCAACAGCTCCTGGGGATTTGGGGTTGCTCTGGAAAACTCATTTG
CACCACTGCTGTGCCTTGGAATGCTAGTTGGAGTAATAAATCTCTGGAACAGATTTGGAATCACACGACCTGGATGGAGT
GGGACAGAGAAATTAACAATTACACAAGCTTAATACACTCCTTAATTGAAGAATCGCAAAACCAGCAAGAAAAGAATGAA
CAAGAATTATTGGAATTAGATAAATGGGCAAGTTTGTGGAATTGGTTTAACATAACAAATTGGCTGTGGTATATAAAATT
ATTCATAATGATAGTAGGAGGCTTGGTAGGTTTAAGAATAGTTTTTGCTGTACTTTCTGTAGTGAATAGAGTTAGGCAGG
GATATTCACCATTATCGTTTCAGACCCACCTCCCAATCCCGAGGGGACCCGACAGGCCCGAAGGAATAGAAGAAGAAGGT
GGAGAGAGAGACAGAGACAGATCCATTCGATTAGTGAACGGATCCTTAGCACTTATCTGGGACGATCTGCGGAGCCTGTG
CCTCTTCAGCTACCACCGCTTGAGAGACTTACTCTTGATTGTAACGAGGATTGTGGAACTTCTGGGACGCAGGGGGTGGG
AAGCCCTCAAATATTGGTGGAATCTCCTACAATATTGGAGTCAGGAGCTAAAGAATAGTGCTGTTAGCTTGCTCAATGCC
ACAGCTATAGCAGTAGCTGAGGGGACAGATAGGGTTATAGAAGTAGTACAAGAAACTTATAGAGCTATTCGCCACATACC
TAGAAGAATAAGACAGGGCTTGGAAAGGATTTTGCTATAAGATGGGTGGCAAGTGGTCAAAAAGTAGTGTGGTTGGATGG
CCTGCTGTAAGGGAAAGAATGAGACGAGCTGAGCCAGCAGCAGATGGGGTGGGAGCAGCATCTCGAGACCTAGAAAAACA
TGGAGCAATCACAAGTAGCAACACAGCAGCTAACAATGCTGCTTGTGCCTGGCTAGAAGCACAAGAGGAGGAGAAGGTGG
GTTTTCCAGTCACACCTCAGGTACCTTTAAGACCAATGACTTACAAGGCAGCTGTAGATCTTAGCCACTTTTTAAAAGAA
AAGGGGGGACTGGAAGGGCTAATTCACTCCCAACGAAGACAAGATATCCTTGATCTGTGGATCTACCACACACAAGGCTA
CTTCCCTGATTGGCAGAACTACACACCAGGACCAGGGATCAGATATCCACTGACCTTTGGATGGCGCTACAAGCTAGTAC
CAGTTGAGCCAGAGAAGTTAGAAGAAGCCAACAAAGGAGAGAACACCAGCTTGTTACACCCTGTGAGCCTGCATGGAATG
GATGACCCGGAGAGAGAAGTGTTAGAGTGGAGGTTTGACAGCCGCCTAGCATTTCATCACGTGGCCCGAGAGCTGCATCC
GGAGTACTTCAAGAACTGCTGATATCGAGCTTGCTACAAGGGACTTTCCGCTGGGGACTTTCCAGGGAGGCGTGGCCTGG
GCGGGACTGGGGAGTGGCGAGCCCTCAGATCCTGCATATAAGCAGCTGCTTTTTGCCTGTACTGGGTCTCTCTGGTTAGA
CCAGATCTGAGCCTGGGAGCTCTCTGGCTAACTAGGGAACCCACTGCTTAAGCCTCAATAAAGCTTGCCTTGAGTGCTTC
```

AAGTAGTGTGTGCCCATCTGTTGTGTGACTCTGGTAACTAGAGATCCCTCA
>5-3envLTR

------------------------------AACAAGAGATGGTGGTAATAACAACAATGGGTCCGAGATCTTCAGACCTG
GAGGAGGAGATATGAAGGACAATTGGAGAAGTGAATTATATAAATATAAAGTAGTAAAAATTGAACCATTAGGAGTAGCA
CCCACCAAGGCAAAGAGAAGAGTGGTGCAGAGAGAAAAAAGAGCAGTAGGAATAGGAGCTTTGTTCCTTAGGTTCTTAGG
AGCAGCAGGAAGCACTATGGGCGCAGCGTCAATGACGCTGACGGTACAGGCCAGACAATTATTGTCTGGTATAGTGCAGC
AGCAGAACAATTTGCTGAGGGCTATTGAGGCGCAACAGCATCTGTTGCAACTCACAGTCTAGGGCATCAAGCAGCTCCAG
GCAAGAATCCTGGCTGTGGAAAGATACCTAAAGGATCAACAGCTCCTGAGGATTTAGGGTTGCTCTGGAAAACTCATTTG
CACCACTGCTGTGCCTTGGAATGCTAGTTGGAGTAATAAATCTCTGGAACAGATTTGGAATCACACGACCTGGATGGAGT
GGGACAGAGAAATTAACAATTACACAAGCTTAATACACTCCTTAATTGAAGAATCGCAAAACCAGCAAGAAAAGAATGAA
CAAGAATTATTGGAATTAGATAAATGGGCAAGTTTGTAGAATTGGTTTAACATAACAAATTGGCTGTGGTATATAAAATT
ATTCATAATGATAGTAGGAGGCTTGGTAGGTTTAAGAATAGTTTTTGCTGTACTTTCTGTAGTGAATAAAGTTAGGCAGG
GATATTCACCATTATCGTTTCAGACCCACCTCCCAATCCCGAGGGGACCCGACAGGCCCGAAGGAATAGAAGAAGAAGGT
GGAGAGAGAGACAGAGACAGATCCATTCGATTAGTGAACGGATCCTTAGCACTTATCTAGGACGATCTGCGGAGCCTGTG
CCTCTTCAGCTACCACCGCTTGAGAGACTTACTCTTGATTGTAACGAGGATTGTGGAACTTCTAGGACGCAGGGGGTGGG
AAGCCCTCAAATATTGGTGGAATCTCCTACAATATTGGAGTCAGGAGCTAAAGAATAGTGCTGTTAGCTTGCTCAATGCC
ACAGCTATAGCAGTAGCTGAGGGGACAGATAAGGTTATAGAAGTAGTACAAGAAGCTTATAGAGCTATTCGCCACATACC
TAGAAGAATAAGACAGGGCTTGGAAAGGATTTTGCTATAAGATGGGTGGCAAGTGGTCAAAAAGTAGTGTGGTTAGATGG
CCTGCTGTAAGGGAAAGAATGAGACGAGCTGAGCCAGCAGCAGATGGGGTGGGAGCAGCATCTCGAGACCTAGAAAAACA
TGGAGCAATCACAAGTAGCAACACAGCAGCTAACAATGCTGCTTGTGCCTGGCTAGAAGCACAAGAGGAGGAGAAGGTGA
GTTTTCCAGTCACACCTCAGGTACCTTTAAGACCAATGACTTACAAGGCAGCTGTAGATCTTAGCCACTTTTTAAAAGAA
AAGGGGGGACTGGAAGGGCTAATTCACTCCCAACGAAGACAAGATATCCTTGATCTGTGGATCTACCACACACAAGGCTA
CTTCCCTGATTGGCAGAACTACACACCAGGACCAGGGATCAGATATCCACTGACCTTTGGATGGCGCTACAAGCTAGTAC
CAGTTGAGCCAGAGAAGTTAGAAGAAGCCAACAAAGGAGAGAACACCAGCTTGTTACACCCTGTGAGCCTGCATGGAATG
GATGACCCGGAGAGAGAAGTGTTAGAGTGGAGGTTTGACAGCCGCCTAGCATTTCATCACGTGGCCCGAGAGCTGCATCC
GGAGTACTTCAAGAACTGCTGATATCGAGCTTGCTACAAGGGACTTTCCGCTGGGGACTTTCCAGGGAGGCGTGGCCTGG
GCGGGACTGGGGAGTGGCGAGCCCTCAGATCCTGCATATAAGCAGCTGCTTTTTGCCTGTACTNGGTCTCTCTGGTTAGA
CCAGATCTGAGCCTGGGAGCTCTCTGGCTAACTAAGGAACCCACTGCTTAAGCCTCAATAAAGCTTGCCTTGAGTGCTTC
AAGTAGTGTGTGCCCATCTGTTGTGTGACTCTGGTAACTAGAGATCCCTCA

>5-6envLTR


```
--------------------------------------------------------------------------------
--------------------------------------------------------------------------------
--------------------------------------------------------------------------------
--------------------------------------------------------------------------------
--------------------------------------------------------------------------------
--------------------------------------------------------------------------------
--------------------------------------------------------------------------------
--------------------------------------------------------------------------------
--------------------------------------------------------------------------------
--------------------------------------------------------------------------------
--------------------------------------------------------------------------------
--------------------------------------------------------------------------------
--------------------------------------------------------------------------------
--------------------------------------------------------------------------------
--------------------------------------------------------------------------------
--------------------------------------------------------------------------------
--------------------------------------------------------------------------------
--------------------------------------------------------------------------------
--------------------------------------------------------------------------------
--------------------------------------------------------------------------------
--------------------------------------------------------------------------------
--------------------------------------------------------------------------------
--------------------------------------------------------------------------------
--------------------------------------------------------------------------------
--------------------------------------------------------------------------------
--------------------------------------------------------------------------------
--------------------------------------------------------------------------------
--------------------------------------------------------------------------------
--------------------------------------------------------------------------------
--------------------------------------------------------------------------------
-------------------------CTATTAACAAAAGATAGTAGTAATAACAACAATAAGTCCGAGATCTTCAGACCTA
AAAGAGGAGATATGAAGGACAATTAAAGAAGTGAATTATATAAATATAAAGTAGTAAAAATTGAACCATTAGGAGTAGCA
CCCACCAAGGCAAAGAGAAGAGTGGTGCAGAGAGAAAAAAGAGCAGTGAGAATAAGAGCTTTGTTCCTTAAGTTCTTAAG
AGCAGCAAGAAGCACTATAGGCGCAGCGTCAATGACGCTGACGGTACAGGCCAGACAATTATTGTCTGGTATAGTGCAGC
AGCAGAACAATTTGCTGAGGGCTATTGAGGCGCAACAGCATCTGTTGCAACTCACAGTCTAGGGCATCAAGCAGCTCCAG
GCAAGAATCCTGGCTGTAGAAAGATACCTAAAGGATCAACAGCTCCTAAGGATTTGAGGTTGCTCTAGAAAACTCATTTG
CACCACTGCTGTGCCTTAGAATGCTAGTTAAAGTAATAAATCTCTAGAACAGATTTAGAATCACACGACCTAAATAGAGT
AAGACAGAGAAATTAACAATTACACAAGCTTAATACACTCCTTAATTAAAAAATCGCAAAACCAGCAAGAAAAGAATGAA
CAAGAATTATTAGAATTAAATAAATAGGCAAGTTTGTAGAATTAGTTTAACATAACAAATTGGCTGTGGTATATAAAATT
ATTCATAATAATAGTAAGAGGCTTAGTAAGTTTAAGAATAGTTTTTGCTATACTTTCTGTAGTGAATAAAGTTAGGCAAA
GATATTCACCATTATCGTTTCAGACCCACCTCCCAATCCCGAGGGGACCCGACAGGCCCGAAAGAATAGAAGAAGAAGGT
GGAGAAAAAGACAAAGACAGATCCATTCGATTAGTGAACGGATCCTTAGCACTTATCTAGGACGATCTGCAGAGCCTGTG
CCTCTTCAGCTACCACCGCTTAAGAGACTTACTCTTGATTGTAACGAGGATTGTAGAACTTCTAAGACGCAGGAAGTAAG
AAGCCCTCAAATATTAGTAGAATCTCCTACAATATTGGAGTCAGGAGCTAAAGAATAGTGCTGTTAGCTTGCTCAATGCC
ACAGCTATAGCAGTAGCTGAGGGGACAGATAAAGTTATAGAAGTAGTACAAGAAGCTTATAAAGCTATTCGCCACATACC
TAGAAGAATAAGACAGGGCTTAGAAAAGATTTTGCTATAAGATAAGTGGCAAGTAGTCAAAAAGTAGTGTAGTTAGATGG
CCTGCTGTAAAAGAAAGAATAAGACAAGCTAAGCCAGCAGCAGATGAGGTAAGAGCAGCATCTCGAGACCTAGAAAAACA
TAGAGCAATCACAAGTAGCAACACAGCAGCTAACAATGCTGCTTGTGCCTAGCTAAAAGCACAAAAAGAAAAGAAAGTAA
GTTTTCCAGTCACACCTCAGGTACCTTTAAGACCAATGACTTACAAGGCAGCTGTAGATCTTAGCCACTTTTTAAAAGAA
AAGGGGGGACTGGAAGGGCTAATTCACTCCCAACGAAGACAAGATATCCTTGATCTGTGGATCTACCACACACAAGGCTA
CTTCCCTGATTGGCAGAACTACACACCAGGACCAGGGATCAGATATCCACTGACCTTTAGATGGCGCTACAAGCTAGTAC
CAGTTGAGCCAGAGAAGTTAGAAGAAGCCAACAAAGGAGAGAACACCAGCTTGTTACACCCTGTGAGCCTGCATAGAATG
GATGACCCGGAGAGAGAAGTGTTAGAGTGGAGGTTTGACAGCCGCCTAGCATTTCATCACGTGGCCCGAGAGCTGCATCC
GGAGTACTTCAAGAACTGCTGATATCGAGCTTGCTACAAAAGACTTTCCGCTGAGGACTTTCCAGGGAGGCGTGGCCTAG
GCAGGACTGAAGAGTGGCGAGCCCTCAGATCCTGCATATAAGCAGCTGCTTTTTGCCTGTACTAGGTCTCTCTGGTTAGA
CCAGATCTGAGCCTAGGAGCTCTCTGGCTAACTAAAGAACCCACTGCTTAAGCCTCAATAAAGCTTGCCTTAAGTGCTTC
AAGTAGTGTGTGCCCATCTGTTGTGTGACTCTAGTAACTAGAGATCCCTCA
>5-8envLTR
--------------------------------------------------------------------------------
--------------------------------------------------------------------------------
--------------------------------------------------------------------------------
--------------------------------------------------------------------------------
--------------------------------------------------------------------------------
--------------------------------------------------------------------------------
--------------------------------------------------------------------------------
--------------------------------------------------------------------------------
--------------------------------------------------------------------------------
--------------------------------------------------------------------------------
--------------------------------------------------------------------------------
--------------------------------------------------------------------------------
--------------------------------------------------------------------------------
--------------------------------------------------------------------------------
--------------------------------------------------------------------------------
--------------------------------------------------------------------------------
--------------------------------------------------------------------------------
--------------------------------------------------------------------------------
--------------------------------------------------------------------------------
--------------------------------------------------------------------------------
--------------------------------------------------------------------------------
--------------------------------------------------------------------------------
--------------------------------------------------------------------------------
--------------------------------------------------------------------------------
--------------------------------------------------------------------------------
--------------------------------------------------------------------------------
--------------------------------------------------------------------------------
--------------------------------------------------------------------------------
--------------------------------------------------------------------------------
--------------------------------------------------------------------------------
--------------------------------------------------------------------------------
--------------------------------------------------------------------------------
--------------------------------------------------------------------------------
--------------------------------------------------------------------------------
--------------------------------------------------------------------------------
--------------------------------------------------------------------------------
```

```
--------------------------------------------------------------------------------
--------------------------------------------------------------------------------
--------------------------------------------------------------------------------
--------------------------------------------------------------------------------
--------------------------------------------------------------------------------
--------------------------------------------------------------------------------
--------------------------------------------------------------------------------
--------------------------------------------------------------------------------
--------------------------------------------------------------------------------
--------------------------------------------------------------------------------
--------------------------------------------------------------------------------
--------------------------------------------------------------------------------
--------------------------------------------------------------------------------
--------------------------------------------------------------------------------
--------------------------------------------------------------------------------
--------------------------------------------------------------------------------
--------------------------------------------------------------------------------
--------------------------------------------------------------------------------
--------------------------------------------------------------------------------
--------------------------------------------------------------------------------
--------------------------------------------------------------------------------
--------------------------------------------------------------------------------
--------------------------------------------------------------------------------
--------------------------------------------------------------------------------
--------------------------------------------------------------------------------
--------------------------------------------------------------------------------
--------------------------------------------------------------------------------
--------------------------------------------------------------------------------
--------------------------------------------------------------------------------
--------------------------------------------------------------------------------
--------------------------------------------------------------------------------
--------------------------------------------------------------------------------
--------------------------------------------------------------------------------
--------------------------------------------------------------------------------
--------------------------------------------------------------------------------
--------------------------------------------------------------------------------
--------------------------------------------------------------------------------
--------------------------------------------------------------------------------
--------------------------------------------------------------------------------
--------------------------------------------------------------------------------
--------------------------------------------------------------------------------
--------------------------------------------------------------------------------
--------------------------------------------------------------------------------
--------------------------------------------------------------------------------
--------------------------------------------------------------------------------
--------------------------------------------------------------------------------
--------------------------------------------------------------------------------
--------------------------------------------------------------------------------
--------------------------------------------------------------------------------
--------------------------------------------------------------------------------
--------------------------------------------------------------------------------
--------------------------------------------------------------------------------
--------------------------------------------------------------------------------
--------------------------------------------------------------------------------
--------------------------------------------------------------------------------
--------------------------------------------------------------------------------
--------------------------------------------------------------------------------
--------------------------------------------------------------------------------
----------------------------TTAACAAGAGATGGTAGTAATAACAACAATAAGTCCGAGATCTTCAGACCTA
GAAGAAGAGATATGAAGGACAATTGGAGAAGTGAATTATATAAATATAAAGTAGTAAAAATTGAACCATTAGGAGTAGCA
CCCACCAAGGCAAAGAGAAGAGTGGTGCAGAGAGAAAAAAGAGCAGTAAGAATAAGAGCTTTGTTCCTTAGGTTCTTAGG
AGCAGCAGGAAGCACTATAGGCGCAGCGTCAATGACGCTGACGGTACAGGCCAGACAATTATTGTCTAGTATAGTGCAGC
AGCAGAACAATTTGCTGAAGGCTATTAAGGCGCAACAGCATCTGTTGCAACTCACAGTCTGGGGCATCAAGCAGCTCCAG
GCAAGAATCCTGGCTGTGGAAAGATACCTAAAGGATCAACAGCTCCTGAGGATTTAAAGTTGCTCTGGAAAACTCATTTG
CACCACTGCTGTGCCTTGGAATGCTAGTTGGAGTAATAAATCTCTAGAACAGATTTAGAATCACACGACCTAGATGGAGT
AGGACAGAGAAATTAACAATTACACAAGCTTAATACACTCCTTAATTGAAGAATCGCAAAACCAGCAAAAAAAGAATGAA
CAAGAATTATTAGAATTAGATAAATGGGCAAGTTTGTAGAATTAGTTTAACATAACAAATTGGCTGTGGTATATAAAATT
ATTCATAATGATAGTAGGAGGCCTGGTAGGTTTAAGAATAGTTTTTGCTGTACTTTCTGTAGTGAATAAAGTTAGGCAAG
GATATTCACCATTATCGTTTCAGACCCACCTCCCAATCCCGAAGGGACCCGACAGGCCCGAAGGAATAGAAGAAGAAGGT
AGAGAGAGAGACAGAGACAGATCCATTCGATTAGTGAACGGATCCTTAGCACTTATCTAAGACGATCTGCGGAGCCTGTG
CCTCTTCAGCTACCACCGCTTGAGAGACTTACTCTTAATTGTAACGAGGATTGTAGAACTTCTAGGACGCAGGGGGTGGG
AAGCCCTCAAATATTAGTAGAATCTCCTACAATATTAGAGTCAGGAGCTAAAGAATAGTGCTGTTAGCTTGCTCAATGCC
ACAGCTATAGCAGTAGCTGAGAGGACAGATAAAGTTATAGAAGTAGTACAAGAAGCTTATAGAGCTATTCGCCACATACC
TAGAAGAATAAGACAAGGCTTGGAAAGGATTTTGCTATAAGATAGGTGGCAAGTGGTCAAAAAGTAGTGTGGTTAGATGG
CCTGCTGTAAAAGAAAGAATGAGACGAGCTGAGCCAGCAGCAGATAAGGTAAGAGCAGCATCTCGAGACCTAGAAAAACA
TGGAGCAATCACAAGTAGCAACACAGCAGCTAACAATGCTGCTTGTGCCTGGCTAAAAGCACAAGAGGAAGAGAAGGTAG
GTTTTCCAGTCACACCTCAGGTACCTTTAAGACCAATGACTTACAAGGCAGCTGTAGATCTTAGCCACTTTTTAAAAGAA
AAGGGGGGACTGGAAAGGCTAATTCACTCCCAACGAAGACAAGATATCCTTGATCTGTGGATCTACCACACACAAGGCTA
CTTCCCTGATTGGCAGAACTACACACCAGGACCAGGGATCAGATATCCACTGACCTTTGGATGGCGCTACAAGCTAGTAC
CAGTTGAGCCAGAGAAGTTAGAAGAAGCCAACAAAGGAGAGAACACCAGCTTGTTACACCCTGTGAGCCTGCATGGAATG
GATGACCCGGAGAGAGAAGTGTTAGAGTGGAGGTTTGACAGCCGCCTAGCATTTCATCACGTGGCCCGAGAGCTGCATCC
GGAGTACTTCAAGAACTGCTGATATCGAGCTTGCTACAAGGGACTTTCCGCTGGGGACTTTCCAGGGAGGCGTGGCCTGG
GCAGGACTGGGGAGTGGCGAGCCCTCAGATCCTGCATATAAGCAGCTGCTTTTTGCCTGTACTAGGTCTCTCTGGTTAGA
CCAGATCTGAGCCTAGGAGCTCTCTGGCTAACTAGGGAACCCACTGCTTAAGCCTCAATAAAGCTTGCCTTGAGTGCTTC
AAGTAGTGTGTGCCCATCTGTTGTGTGACTCTGGTAACTAGAGATCCCTCA
>5-10envLTR
--------------------------------------------------------------------------------
--------------------------------------------------------------------------------
--------------------------------------------------------------------------------
--------------------------------------------------------------------------------
--------------------------------------------------------------------------------
--------------------------------------------------------------------------------
--------------------------------------------------------------------------------
--------------------------------------------------------------------------------
```


----------------------CTGCTATTAACAAGAGATGGTGGTAATAACAACAATGGGTCCGAGATCTTCAGACCTG
GAGGAGGAGATATGAGGGACAATTGGAGAAGTGAATTATATAAATATAAAGTAGTAAAAATTGAACCATTAGGAGTAGCA
CCCACCAAGGCAAAGAGAAGAGTGGTGCAGAGAGAAAAAAGAGCAGTGGGAATAGGAGCTTTGTTCCTTGGGTTCTTGGG
AGCAGCAGGAAGCACTATGGGCGCAGCGTCAATGACGCTGACGGTACAGGCCATACAATTATTGTCTGGTATAGTGCAGC
AGCAGAACAATTTGCTGAGGGCTATTGAGGCGCAACAGCATCTGTTGCAACTCACAGTCTGGGGCATCAAGCAGCTCCAG
GCAAGAATCCTGGCTGTGGAAAGATACCTAAAGGATCAACAGCTCCTGGGGATTTGGGGTTGCTCTGGAAAACTCATTTG
CACCACTGCTGTGCCTTGGAATGCTAGTTGGAGTAATAAATCTCTGGAACAGATTTGGAATCACACGACCTGGATGGAGT
GGGACAGAGAAATTAACAATTACACAAGCTTAATACACTCCTTAATTGAAGAATCGCAAAACCAGCAAGAAAAGAATGAA

CAAGAATTATTGGAATTAGATAAATGGGCAAGTTTGTGGAATTGGTTTAACATAACAAATTGGCTGTGGTATATAAAATT
ATTCATAATGATAGTAGGAGGCTTGGTAGGTTTAAGAATAGTTTTTGCTGTACTTTCTGTAGTGAATAGAGTTAGGCAGG
GATATTCACCATTATCGTTTCAGACCCACCTCCCAATCCCGAGGGGACCCGACAGGCCCGAAGGAATAGAAGAAGAAGGT
GGAGAGAGAGACAGAGACAGATCCATTCGATTAGTGAACGGATCCTTAGCACTTATCTGGGACGATCTGCGGAGCCTGTG
CCTCTTCAGCTACCACCGCTTGAGAGACTTACTCTTGATTGTAACGAGGATTGTGGAACTTCTGGGACGCAGGGGGTGGG
AAGCCCTCAAATATTGGTGGAATCTCCTACAATATTGGAGTCAGGAGCTAAAGAATAGTGCTGTTAGCTTGCTCAATGCC
ACAGCTATAGCAGTAGCTGAGGGGACAGATAGGGTTATAGAAGTAGTACAAGAAGCTTATAGAGCTATTCGCCACATACC
TAGAAGAATAAGACAGGGCTTGAAAAGGATTTTGCTATAAGATGGGTGGCAAGTGGTCAAAAAGTAGTGTGGTTGGATGG
CCTGCTGTAAGGGAAAGAATGAGACGAGCTGAGCCAGCAGCAGATGGGGTGGGAGCAGCATCTCGAGACCTAGAAAAACA
TGGAGCAATCACAAGTAGCAACACAGCAGCTAACAATGCTGCTTGTGCCTGGCTAGAAGCACAAGAGGAGGAGAAGGTGG
GTTTTCCAGTCACACCTCAGGTACCTTTAAGACCAATGACTTACAAGGCAGCTGTAGATCTTAGCCACTTTTTAAAAGAA
AAGGGGGGACTGGAAGGGCTAATTCACTCCCAACGAAGACAAGATATCCTTGATCTGTGGATCTACCACACACAAGGCTA
CTTCCCTGATTGGCAGAACTACACACCAGGACCAGGGATCAGATATCCACTGACCTTTGGATGGCGCTACAAGCTAGTAC
CAGTTGAGCCAGAGAAGTTAGAAGAAGCCAACAAAGGAGAGAACACCAGCTTGTTACACCCTGTGAGCCTGCATGGAATG
GATGACCCGGAGAGAGAAGTGTTAGAGTGGAGGTTTGACAGCCGCCTAGCATTTCATCACGTGGCCCGAGAGCTGCATCC
GGAGTACTTCAAGAACTGCTGATATCGAGCTTGCTACAAGGGACTTTCCGCTGGGGACTTTCCAGGGAGGCGTGGCCTGG
GCGGGACTGGGGAGTGGCGAGCCCTCAGATCCTGCATATAAGCAGCTGCTTTTTGCCTGTACTGGGTCTCTCTGGTTAGA
CCAGATCTGAGCCTGGGAGCTCTCTGGCTAACTAGGGAACCCACTGCTTAAGCCTCAATAAAGCTTGCCTTGAGTGCTTC
AAGTAGTGTGTGCCCATCTGTTGTGTGACTCTGGTAACTAGAGATCCCTCA

>5-11envLTR

--CCGAGATCTTCANACCTG
GAAGAGGAGATATGAAGGACAATTAGAGAAGTGAATTATATAAATATAAAGTAGTAAAAATTGAACCATTAGGAGTAGCA
CCCACCAAGGCAAAGAGAAGAGTGGTGCAGAGAGAAAAAAGAGCAGTAAGAATAAGAGCTTTGTTCCTTAAGTTCTTAAG
AGCAGCAGGAAGCACTATAGGCGCAGCGTCAATGACGCTGACAGTACAGGCCAGACAATTATTGTCTGGTATAGTGCAGC
AGCAGAACAATTTGCTGAGGGCTATTGAGGCGCAACAGCATCTGTTGCAACTCACAGTCTAAGGCATCAAGCAGCTCCAG
GCAAGAATCCTGGCTGTAGAAAGATACCTAAAAGATCAACAGCTCCTAAAGATTTAAGGTTGCTCTAGAAAACTCATTTG
CACCACTGCTGTGCCTTAGAATGCTAGTTAAAGTAATAAATCTCTAGAACAGATTTAGAATCACACGACCTAGATGGAGT
AGGACAGAGAAATTAACAATTACACAAGCTTAATACACTCCTTAATTGAAGAATCGCAAAACCAGCAAGAAAAGAATGAA
CAAGAATTATTAGAATTAGATAAATAGGCAAGTTTGTAGAATTAGTTTAACATAACAAATTGGCTGTAGTATATAAAATT
ATTCATAATGATAGTAAGAAGCTTAGTATGTTTAAGAATAGTTTTTGCTGTACTTTCTGTAGTGAATAGAGTTAGGCAGA
GATATTCACCATTATCGTTTCAGACCCACCTCCCAATCCCGAGGGGACCCGACAGGCCCGAAAGAATAGAAGAAGAAGGT
AGAGAAAAAGACAGAGACAGATCCATTCGATTAGTGAACAGATCCTTAGCACTTATCTAAGACGATCTGCGGAGCCTGTG
CCTCTTCAGCTACCACCGCTTGAAAGACTTACTCTTGATTGTAACGAAGATTGTAGAACTTCTAAGACGCAGAGGGTAAG
AAGCCCTCAAATATTAGTAGAATCTCCTACAATATTAGAGTCAAGAGCTAAAGAATAGTGCTGTTAGCTTGCTCAATGCC
ACAGCTATAGCAGTAGCTGAAAGGACAGATAAGGTTATAGAAGTAGTACAAAAAGCTTATAGAGCTATTCGCCACATACC
TAGAAGAATAAGACAAGGCTTAGAAAAGATTTTGCTATAAGATAAGTGGCAAGTGGTCAAAAAGTAGTGTGGTTAGATGG
CCTGCTGTAAAGGAAAGAATGAGACGAGCTAAGCCAGCAGCAGATGAGGTGAGAGCAGCATCTCGAGACCTAGAAAAACA
TAGAGCAATCACAAGTAGCAACACAGCAGCTAACAATGCTGCTTGTGCCTGGCTAGAAGCACAAAAAGAAGAGAAAGTAG
GTTTTCCAGTCACACCTCAGGTACCTTTAAGACCAATGACTTACAAGGCAGCTGTAGATCTTAGCCACTTTTTAAAAGAA
AAGGGGGGACTGGAAGGGCTAATTCACTCCCAACGAAGACAAGATATCCTTGATCTGTGGATCTACCACACACAAGGCTA
CTTCCCTGATTGGCAGAACTACACACCAGGACCAGGGATCAGATATCCACTGACCTTTAGATGGCGCTACAAGCTAGTAC
CAGTTGAGCCAGAGAAGTTAGAAGAAGCCAACAAAGGAGAGAACACCAGCTTGTTACACCCTGTGAGCCTGCATGGAATG
GATGACCCGGAGAGAGAAGTGTTAGAGTGGAGGTTTGACAGCCGCCTAGCATTTCATCACGTGGCCCGAGAGCTGCATCC
GGAGTACTTCAAGAACTGCTGATATCGAGCTTGCTACAAGGGACTTTCCGCTAAGGACTTTCCAAGGAGGCGTGGCCTGG
GCAGGACTGGGGAGTGGCGAGCCCTCAGATCCTGCATATAAGCAGCTGCTTTTTGCCTGTACTAGGTCTCTCTGGTTAGA
CCAGATCTGAGCCTAGGAGCTCTCTGGCTAACTAAGGAACCCACTGCTTAAGCCTCAATAAAGCTTGCCTTGAGTGCTTC
AAGTAGTGTGTGCCCATCTGTTGTGTGACTCTAGTAACTAGAGATCCCTCA
>5-13envLTR


```
--------------------------------------------------------------------------------
--------------------------------------------------------------------------------
--------------------------------------------------------------------------------
--------------------------------------------------------------------------------
--------------------------------------------------------------------------------
--------------------------------------------------------------------------------
--------------------------------------------------------------------------------
--------------------------------------------------------------------------------
--------------------------------------------------------------------------------
--------------------------------------------------------------------------------
--------------------------------------------------------------------------------
--------------------------------------------------------------------------------
--------------------------------------------------------------------------------
--------------------------------------------------------------------------------
--------------------------------------------------------------------------------
--------------------------------------------------------------------------------
--------------------------------------------------------------------------------
--------------------------------------------------------------------------------
--------------------------------------------------------------------------------
--------------------------------------------------------------------------------
--------------------------------------------------------------------------------
--------------------------------------------------------------------------------
--------------------------------------------------------------------------------
--------------------------------------------------------------------------------
--------------------------------------------------------------------------------
--------------------------------------------------------------------------------
--------------------------------------------------------------------------------
--------------------------------------------------------------------------------
--------------------------------------------------------------------------------
--------------------------------------------------------------------------------
--------------------------------------------------------------------------------
--------------------------------------------------------------------------------
--------------------------------------------------------------------------------
--------------------------------------------------------------------------------
--------------------------------------------------------------------------------
--------------------------------------------------------------------------------
--------------------------------------------------------------------------------
--------------------------------------------------------------------------------
--------------------------------------------------------------------------------
--------------------------------------------------------------------------------
--------------------------------------------------------------------------------
--------------------------------------------------------------------------------
--------------------------------------------------------------------------------
--------------------------------------------------------------------------------
--------------------------------------------------------------------------------
--------------------------------------------------------------------------------
--------------------------------------------------------------------------------
--------------------------------------------------------------------------------
------------------------------AACAAGAGATGGTAGTAATAACAACAATAGGTCCAAGATCTTCAGACCTG
GAGGAGGAGATATGAAGGACAATTAGAGAAGTGAATTATATAAATATAAAGTAGTAAAAATTGAACCATTAGGAGTAGCA
CCCACCAAGGCAAAGAGAAGAGTGGTGCANAGAGAAAAAAGAGCAGTAAGAATAGGAGCTTTGTTCCTTAAGTTCTTAAG
AGCAGCAGGAAGCACTATAGGCGCAGCGTCAATGACGCTGACAGTACAGGCCAGACAATTATTGTCTGGTATAGTGCAGC
AGCAGAACAATTTGCTGAGGGCTATTAAGGCGCAACAGCATCTGTTGCAACTCACAGTCTGGGGCATCAAGCAGCTCCAG
GCAAGAATCCTGGCTGTAGAAAGATACCTAAAAGATCAACAGCTCCTGGGGATTTGGGGTTGCTCTAGAAAACTCATTTG
CACCACTGCTGTGCCTTAGAATGCTAGTTAGAGTAATAAATCTCTAGAACAGATTTAGAATCACACGACCTAGATAGAGT
AGGACAGAGAAATTAACAATTACACAAGCTTAATACACTCCTTAATTGAAGAATCGCAAAACCAGCAAGAAAAGAATGAA
CAAGAATTATTAGAATTAGATAAATAGGCAAGTTTGTAGAATTAGTTTAACATAACAAATTGGCTGTGGTATATAAAATT
ATTCATAATGATAGTAAGAAGCTTAGTAAGTTTAAGAATAGTTTTTGCTGTACTTTCTGTAGTGAATAAAGTTAGGCAAA
GATATTCACCATTATCGTTTCAGACCCACCTCCCAATCCCGAAAGGACCCGACAGGCCCGAAGGAATAGAAGAAGAAGGT
AAAGAGAGAGACAAAGACAGATCCATTCAATTAGTGAACGGATCCTTAGCACTTATCTAGGACGATCTGCAGAGCCTGTG
CCTCTTCAGCTACCACCGCTTGAAAGACTTACTCTTGATTGTAACGAAGATTGTAGAACTTCTGGGACGCAGGGGGTAAG
AAGCCCTCAAATATTAGTAGAATCTCCTACAATATTGGAGTCAGGAGCTAAAGAATAGTGCTGTTAGCTTGCTCAATGCC
ACAGCTATAGCAGTAGCTGAGAGGACAGATAAGGTTATAGAAGTAGTACAAGAAGCTTATAGAGCTATTCGCCACATACA
TAGAAGAATAAGACAGGGCTTAGAAAGGATTTTGCTATAAGATAGGTGGCAAGTGGTCAAAAAGTAGTGTGGTTAGATGG
CCTGCTGTAAAGGAAAGAATGAGACGAGCTGAGCCAGCAGCAGATAAGGTAGGAGCAGCATCTCGAGACCTAGAAAAACA
TGGAGCAATCACAAGTAGCAACACAGCAGCTAACAATGCTGCTTGTGCCTAGCTAGAAGCACAAGAGGAGGAGAAGGTGG
GTTTTCCAGTCACACCTCAGGTACCTTTAAGACCAATGACTTACAAGGCAGCTGTAGATCTTAGCCACTTTTTAAAAGAA
AAGGGGGGACTGGAAGGGCTAATTCACTCCCAACGAAGACAAGATATCCTTGATCTGTGGATCTACCACACACAAGGCTA
CTTCCCTGATTGGCAGAACTACACACCAGGACCAGGGATCAGATATCCACTGACCTTTGGATGGCGCTACAAGCTAGTAC
CAGTTGAGCCAGAGAAGTTAGAAGAAGCCAACAAAGGAGAGAACACCAGCTTGTTACACCCTGTGAGCCTGCATGGAATG
GATGACCCGGAGAGAGAAGTGTTAGAGTGGAGGTTTGACAGCCGCCTAGCATTTCATCACGTGGCCCGAGAGCTGCATCC
GGAGTACTTCAAGAACTGCTGATATCGAGCTTGCTACAAGGGACTTTCCGCTAGGGACTTTCCAGGGAAGCGTGGCCTGG
GCGAGACTGGGGAGTGGCGAGCCCTCAGATCCTGCATATAAGCAGCTGCTTTTTGCCTGTACTAGGTCTCTCTGGTTAGA
CCAGATCTGAGCCTCGGAGCTCTCTGGCTAACTAGGGAACCCACTGCTTAAGCCTCAATAAAGCTTGCCTTGAGTGCTTC
AAGTAGTGTGTGCCCATCTGTTGTGTGACTCTAGTAACTAGAGATCCCTCA
>5-17envLTR
--------------------------------------------------------------------------------
--------------------------------------------------------------------------------
--------------------------------------------------------------------------------
--------------------------------------------------------------------------------
--------------------------------------------------------------------------------
--------------------------------------------------------------------------------
--------------------------------------------------------------------------------
--------------------------------------------------------------------------------
--------------------------------------------------------------------------------
--------------------------------------------------------------------------------
--------------------------------------------------------------------------------
--------------------------------------------------------------------------------
--------------------------------------------------------------------------------
--------------------------------------------------------------------------------
--------------------------------------------------------------------------------
--------------------------------------------------------------------------------
--------------------------------------------------------------------------------
--------------------------------------------------------------------------------
```

```
--------------------------------------------------------------------------------
--------------------------------------------------------------------------------
--------------------------------------------------------------------------------
--------------------------------------------------------------------------------
--------------------------------------------------------------------------------
--------------------------------------------------------------------------------
--------------------------------------------------------------------------------
--------------------------------------------------------------------------------
--------------------------------------------------------------------------------
--------------------------------------------------------------------------------
--------------------------------------------------------------------------------
--------------------------------------------------------------------------------
--------------------------------------------------------------------------------
--------------------------------------------------------------------------------
--------------------------------------------------------------------------------
--------------------------------------------------------------------------------
--------------------------------------------------------------------------------
--------------------------------------------------------------------------------
--------------------------------------------------------------------------------
--------------------------------------------------------------------------------
--------------------------------------------------------------------------------
--------------------------------------------------------------------------------
--------------------------------------------------------------------------------
--------------------------------------------------------------------------------
--------------------------------------------------------------------------------
--------------------------------------------------------------------------------
--------------------------------------------------------------------------------
--------------------------------------------------------------------------------
--------------------------------------------------------------------------------
--------------------------------------------------------------------------------
--------------------------------------------------------------------------------
--------------------------------------------------------------------------------
--------------------------------------------------------------------------------
--------------------------------------------------------------------------------
--------------------------------------------------------------------------------
--------------------------------------------------------------------------------
--------------------------------------------------------------------------------
--------------------------------------------------------------------------------
--------------------------------------------------------------------------------
--------------------------------------------------------------------------------
--------------------------------------------------------------------------------
--------------------------------------------------------------------------------
--------------------------------------------------------------------------------
--------------------------------------------------------------------------------
--------------------------------------------------------------------------------
--------------------------------------------------------------------------------
--------------------------------------------------------------------------------
--------------------------------------------------------------------------------
--------------------------------------------------------------------------------
--------------------------------------------------------------------------------
--------------------------------------------------------------------------------
--------------------------------------------------------------------------------
--------------------------------------------------------------------------------
--------------------------------------------------------------------------------
--------------------------------------------------------------------------------
--------------------------------------------------------------------------------
--------------------------------------------------------------------------------
--------------------------------------------------------------------------------
--------------------------------------------------------------------------------
--------------------------------------------------------------------------------
--------------------------------------------------------------------------------
--------------------------------------------------------------------------------
--------------------------------------------------------------------------------
--------------------------------------------------------------------------------
--------------------------------------------------------------------------------
--------------------------------------------------------------------------------
--------------------------------------------------------------------------------
--------------------------------------------------------------------------------
--------------------------------------------------------------------------------
--------------------------------------------------------------------------------
--------------------------------------------------------------------------------
--------------------------------------------------------------------------------
--------------------------------------------------------------------------------
--------------------------------------------------------------------------------
--------------------------------------------------------------------------------
--------------------------------------------------------------------------------
-------------------------------------GATGGTGGTAATAACAACAATAGGTCCGAGATCTTCAGACCTG
GAGGAGGAGATATGAAGGACAATTGGAGAAGTGAATTATATAAATATAAAGTAGTAAAAATTGAACCATTAGGAGTAGCA
CCCACCAAGGCAAAGAGAAGAGTGGTGCAGAGAGAAAAAAGAGCAGTAAGAATAGGAGCTTTGTTCCTTAGGTTCTTAAG
AGCAGCAGGAAGCACTATAGGCGCAGCGTCAATGACGCTGACGGTACAGGTCAGACAATTATTGTCTGGTATAGTGCAGC
AGCAGAACAATTTGCTGAGGGCTATTGAGGCGCAACAGCATCTGTTGCAACTCACAGTCTAGGGCATCAAGCAGCTCCAG
GCAAGAATCCTGGCTGTAGAAAAATACCTAAAGGATCAACAGCTCCTGGGGATTTGGGGTTGCTCTAGAAAACTCATTTG
CACCACTGCTGTGCCTTAGAATGCTAGTTAGAGTAATAAATCTCTAGAACAGATTTAGAATCACACGACCTGGATGGAGT
GGGACAGAGAAATTAACAATTACACAAGCTTAATACACTCCTTAATTGAAGAATCGCAAAACCAGCAAGAAAAGAATGAA
CAAGAATTATTAGAATTAGATAAATAGGCAAGTTTGTGGAATTGGTTTAACATAACAAATTGGCTGTGGTATATAAAATT
ATTCATAATGATAGTAAGAGGCTTAGTAGGTTTAAGAATAGTTTTTGCTGTACTTTCTGTAGTGAATAAAGTTAGGCAAA
GATATTCACCATTATCGTTTCAGACCCACCTCCCAATCCCGAGGGGACCCGACAGGCCCGAAGGAATAGAAGAAGAAGGT
GGAGAAAGAGACAGAGACAGATCCATTCGATTAGTGAACGGATCCTTAGCACTTATCTAGGACGATCCGCGGAGCCTGTG
CCTCTTCAGCTACCACCGCTTGAGAGACTTACTCTTGATTGTAACGAGGATTGTAGAACTTCTAGGACGCAGGGGGTAAG
AAGCCCTCAAATATTGGTGGAATCTCCTACAATATTGGAGTCAGGAGCTAAAGAATAGTGCTGTTAGCTTGCTCAATGCC
ACAGCTATAGCAGTAGCTGAGAGGACAGATAAAGTTATAGAAGTAGTACAAGAAGCTTATAGAGCTATTCGCCACATACC
TAGAAGAATAAGACAGGGCTTAGAAAAGATTTTGCTATAAGATAGGTGGCAAGTGGTCAAAAAGTAGTGTGGTTAGATGG
CCTGCTGTAAAGGAAAGAATGAGACGAGCTAAGCCAGCAGCAGATAAGGTAGGAGCAGCATCTCGAGACCTAGAAAAACA
TGGAGCAATCACAAGTAGCAACACAGCAGCTAACAATGCTGCTTGTGCCTGGCTAGAAGCACAAGAAGAGGAGAAGGTAG
```

GTTTTCCAGTCACACCTCAGGTACCTTTAAGACCAATGACTTACAAGGCAGCTGTAGATCTTAGCCACTTTTTAAAAGAA
AAGGGGGGACTGGAAGGGCTAATTCACTCCCAACGAAGACAAGATATCCTTGATCTGTGGATCTACCACACACAAGGCTA
CTTCCCTGATTGGCAGAACTACACACCAGGACCAAGGATCAGATATCCACTGACCTTTGGATGGCGCTACAAGCTAGTAC
CAGTTGAGCCAGAGAAGTTAGAAGAAGCCAACAAAGGAGAGAACACCAGCTTGTTACACCCTGTGAGCCTGCATGGAATG
GATGACCCGGAGAGAGAAGTGTTAGAGTGGAGGTTTGACAGCCGCCTAGCATTTCATCACGTGGCCCGAGAGCTGCATCC
GGAGTACTTCAAGAACTGCTGATATCGAGCTTGCTACAAGGGACTTTCCGCTGGGGACTTTCCAGGGAGGCGTGGCCTGG
GCGGGACTGGGGAGTGGCGAGCCCTCAGATCCTGCATATAAGCAGCTGCTTTTTGCCTGTACTGGGTCTCTCTGGTTAGA
CCAGATCTGAGCCTAGGAGCTCTCTGGCTAACTAGGGAACCCACTGCTTAAGCCTCAATAAAGCTTGCCTTGAGTGCTTC
AAGTAGTGTGTGCCCATCTGTTGTGTGACTCTGGTAACTAGAGATCCCTCA

>5-22envLTR

------------------------GCTATTAACAAAAAATAGTAGTAATAACAACAATAAGTCCAAGATCTTCAGACCTA
GAAGAAGAGATATAAAGGACAATTAAAGAAGTAAATTATATAAATATAAAGTAGTAAAAATTGAACCATTAAGAGTAGCA
CCCACCAAGGCAAAGAGAAGAGTAGTGCAGAGAGAAAAAAGAGCAGTAAGAATAAGAGCTTTGTTCCTTAAGTTCTTAGG
AGCAGCAAGAAGCACTATAGGCGCAGCGTCAATGACGCTGACAGTACAGGCCAGACAATTATTGTCTAGTATAGTGCAGC
AGCAGAACAATTTGCTGAGGGCTATTAAGGCGCAACAGCATCTGTTGCAACTCACAGTCTAAGGCATCAAGCAGCTCCAG
GCAAGAATCCTGGCTGTAGAAAGATACCTAAAAGATCAACAGCTCCTGAAGATTTAAAGTTGCTCTAGAAAACTCATTTG
CACCACTGCTGTGCCTTAGAATGCTAGTTAGAGTAATAAATCTCTAGAACAGATTTAGAATCACACGACCTAGATAAAGT
AAGACAGAAAAATTAACAATTACACAAGCTTAATACACTCCTTAATTGAAGAATCGCAAAACCAGCAAGAAAAGAATGAA
CAAGAATTATTAGAATTAGATAAATAGGCAAGTTTGTAGAATTAGTTTAACATAACAAATTGGCTGTAGTATATAAAATT
ATTCATAATAATAGTAAGAAGCTTAGTAAGTTTAAGAATAGTTTTTGCTGTACTTTCTGTAGTGAATAAAGTTAGGCAAA
GATATTCACCATTATCGTTTCAGACCCACCTCCCAATCCCGAAAAGACCCGACAGGCCCGAAAGAATAAAAGAAGAAAGT
GAAAAGAAAGACAGAGACAGATCCATTCAATTAGTGAACGGATCCTTAGCACTTATCTAAGACAATCTGCGGAGCCTGTG
CCTCTTCAGCTACCACCGCTTGAAAGACTTACTCTTGATTGTAACGAAGATTGTAGAACTTCTAAGACGCAAGAAATAAG
AAGCCCTCAAATATTAGTAGAATCTCCTACAATATTAAAGTCAGAAGCTAAAGAATAGTGCTGTTAGCTTGCTCAATGCC
ACAGCTATAGCAGTAGCTAAGAGGACAGATAAAGTTATAGAAGTAGTACAAGAAGCTTATAGAGCTATTCGCCACATACC
TAGAAAAATAAGACAAGGCTTAAAAAAGATTTTGCTATAAGATAGGTGGCAAGTGGTCAAAAAGTAGTGTAGTTAGATGG
CCTGCTGTAAAAGAAAGAATGAGACGAGCTAAGCCAGCAGCAGATAAGGTAAGAGCAGCATCTCAAGACCTAGAAAAACA
TAGAGCAATCACAAGTAGCAACACAGCAGCTAACAATGCTGCTTGTGCCTGGCTAGAAGCACAAGAAGAAGAGAAAGTAA
GTTTTCCAGTCACACCTCAGGTACCTTTAAGACCAATGACTTACAAGGCAGCTGTAGATCTTAGCCACTTTTTAAAAGAA
AAGGGGGGACTAGAAAGGCTAATTCACTCCCAACGAAGACAAGATATCCTTGATCTGTAGATCTACCACACACAAAGCTA
CTTCCCTGATTGGCAGAACTACACACCAGGACCAAAGATCAGATATCCACTGACCTTTAGATGGCGCTACAAGCTAGTAC
CAGTTAAGCCAAAGAAGTTAGAAAAAGCCAACAAAAGAAAAAACACCAGCTTGTTACACCCTGTGAGCCTGCATAGAATA
GATGACCCAGAGAGAAAAGTGTTAGAGTAGAAGTTTGACAGCCGCCTAGCATTTCATCACGTGGCCCAAGAGCTGCATCC
AGAGTACTTCAAGAACTGCTGATATCGAGCTTGCTACAAAAGACTTTCCGCTGAAGACTTTCCAAAGAAGCGTGGCCTAG
GCAGGACTGAGGAGTGGCGAGCCCTCAGATCCTGCATATAAGCAGCTGCTTTTTGCCTGTACTAGGTCTCTCTAGTTAGA
CCAGATCTGAGCCTAAGAGCTCTCTGGCTAACTAAGGAACCCACTGCTTAAGCCTCAATAAAGCTTGCCTTAAGTGCTTC
AAGTAGTGTGTGCCCATCTGTTGTGTGACTCTAGTAACTAGAGATCCCTCA

>5-26envLTR

-------------------GGGCTGCTATTAACAAGAGATAGTAGTAATAACAACAATAGGTCCGAGATCTTCAGACCTG
GAGGAGGAGATATGAAGGACAATTAGAGAAGTGAATTATATAAATATAAAGTAGTAAAAATTAAACCATTAAGAGTAGCA
CCCACCAAGGCAAAGAGAAGAGTGGTGCAGAGAGAAAAAAGAGCAGTAAGAATAAAAGCTTTGTTCCTTAGGTTCTTAGG
AGCAGCAGGAAGCACTATAGGCGCAGCGTCAATGACGCTGACAGTACAGGCCAGACAATTATTGTCTGGTATAGTGCAGC
AGCAGAACAATTTGCTGAGGGCTATTGAGGCGCAACAGCATCTGTTGCAACTCACAGTCTAAGGCATCAAGCAGCTCCAG
GCAAGAATCCTGGCTGTAGAAAGATACCTAAAAGATCAACAGCTCCTGAGGATTTGAGGTTGCTCTAAAAAACTCATTTG
CACCACTGCTGTGCCTTAGAATGCTAGTTAGAGTAATAAATCTCTAGAACAGATTTAAAATCACACGACCTAGATAGAGT
AGGACAGAGAAATTAACAATTACACAAGCTTAATACACTCCTTAATTAAAGAATCGCAAAACCAGCAAGAAAAGAATGAA
CAAGAATTATTAGAATTAGATAAATGGGCAAGTTTGTAAAATTAGTTTAACATAACAAATTAGCTGTGGTATATAAAATT
ATTCATAATGATAGTAAGAGGCTTAGTAAGTTTAAGAATAGTTTTTGCTGTACTTTCTGTAGTGAATAGAGTTAGGCAAA
GATATTCACCATTATCGTTTCAGACCCACCTCCCAATCCCGAGGGGACCCGACAGGCCCGAAAGAATAGAAGAAGAAGGT
AAAAAGAAAGACAGAGACAGATCCATTCGATTAGTGAACAGATCCTTAGCACTTATCTAGGACGATCTGCGGAGCCTGTG
CCTCTTCAGCTACCACCGCTTGAGAGACTTACTCTTGATTGTAACGAAGATTGTGGAACTTCTAAGACGCAGGAGGTAGG
AAGCCCTCAAATATTAGTAAAATCTCCTACAATATTAGAGTCAGGAGCTAAAGAATAGTGCTGTTAGCTTGCTCAATGCC
ACAGCTATAGCAGTAGCTGAAAGGACAGATAAAGTTATAGAAGTAGTACAAGAAGCTTATAGAGCTATTCGCCACATACC
TAGAAGAATAAGACAGGGCTTAGAAAAAATTTTGCTATAAGATATGTGGCAAGTGGTCAAAAAGTAGTGTAGTTAGATGG
CCTGCTGTAAAAGAAAGAATGAGACGAGCTAAGCCAGCAGCAGATAGGGTAGGAGCAGCATCTCGAGACCTAGAAAAACA
TAGAGCAATCACAAGTAGCAACACAGCAGCTAACAATGCTGCTTGTGCCTAGCTAAAAGCACAAGAGGAGGAGAAGGTAT
GTTTTCCAGTCACACCTCAGGTACCTTTAAGACCAATGACTTACAAGGCAGCTGTAGATCTTAGCCACTTTTTAAAAGAA
AAGGGGGGACTGGAAGGGCTAATTCACTCCCAACGAAGACAAGATATCCTTGATCTGTGGATCTACCACACACAAGGCTA
CTTCCCTGATTGGCAGAACTACACACCAGGACCAGGGATCAGATATCCACTGACCTTTGGATGGCGCTACAAGCTAGTAC
CAGTTGAGCCAGAGAAGTTAGAAGAAGCCAACAAAGGAGAGAACACCAGCTTGTTACACCCTGTGAGCCTGCATAGAATG
GATGACCCGGAGAGAGAAGTGTTAGAGTGGAGGTTTGACAGCCGCCTAGCATTTCATCACGTGGCCCGAGAGCTGCATCC
AGAGTACTTCAAGAACTGCTGATATCGAGCTTGCTACAAAGGACTTTCCGCTGAAGACTTTCCAGAGAGGCGTGGCCTAG
GCAGGACTAGAGAGTGGCGAGCCCTCAGATCCTGCATATAAGCAGCTGCTTTTTGCCTGTACTAGGTCTCTCTGGTTAGA
CCAGATCTGAGCCTAGGAGCTCTCTGGCTAACTAAGGAACCCACTGCTTAAGCCTCAATAAAGCTTGCCTTGAGTGCTTC
AAGTAGTGTGTGCCCATCTGTTGTGTGACTCTAGTAACTAGAGATCCCTCA

>5-32envLTR

--AAAGCAATGTATGCCCCTCCCATCAGCGGACAAATTAA
ATGTTCATCAAATATTACAGGGCTGCTATTAACAAGAGATGGTGGTAATAACAACAATGGGTCCGAGATCTTCAGACCTG
GAGGAGGAGATATGAGGGACAATTGGAGAAGTGAATTATATAAATATAAAGTAGTAAAAATTGAACCATTAGGAGTAGCA
CCCACCAAGGCAAAGAGAAGAGTGGTGCAGAGAGAAAAAAGAGCAGTAGGAATAGGAGCTTTGTTCCTTAGGTTCTTAGG
AGCAGCAGGAAGCACTATGGGCGCAGCGTCAATGACGCTGACGGTACAGGCCAGACAATTATTGTCTGGTATAGTGCAGC
AACAGAACAATTTGCTGAGGGCTATTGAGGCGCAACAGCATCTGTTGCAACTCACAGTCTGGGGCATCAAGCAGCTCCAG
GCAAGAATCCTGGCTGTGGAAAGATACCTAAAGGATCAACAGCTCCTGGGGATTTGAGGTTGCTCTGGAAAACTCATTTG
CACCACTGCTGTGCCTTGGAATGCTAGTTGGAGTAATAAATCTCTGGAACAGATTTGGAATCACACGACCTGGATGGAGT
AGGACAGAGAAATTAACAATTACACAAGCTTAATACACTCCTTAATTGAAGAATCGCAAAACCAGCAAGAAAAGAATGAA
CAAGAATTATTGGAATTAGATAAATGGGCAAGTTTGTAGAATTGGTTTAACATAACAAATTGGCTGTGGTATATAAAATT
ATTCATAATGATAGTAGGAGGCTTGGTAGGTTTAAGAATAGTTTTTGCTGTACTTTCTGTAGTGAATAGAGTTAGGCAGG
GATATTCACCATTATCGTTTCAGACCCACCTCCCAATCCCGAGGGGACCCGACAGGCCCGAAGGAATAGAAGAAGAAAGT
GGAGAAAGAGACAGAGACAGATCCATTCGATTAGTGAACGGATCCTTAGCACTTATCTAGGACGATCTGCGGAGCCTGTG
CCTCTTCAGCTACCACCGCTTGAGAGACTTACTCTTGATTGTAACGAGGATTGTAGAACTTCTAGGACGCAGGGGGTGGG
AAGCCCTCAAATATTAGTAGAATCTCCTACAATATTGGAGTCAGGAGCTAAAGAATAGTGCTGTTAGCTTGCTCAATGCC
ACAGCTATAGCAGTAGCTGAGAGGACAGATAAGGTTATAGAAGTAGTACAAGAAGCTTATAGAGCTATTCGCCACATACC
TAGAAGAATAAGACAAGGCTTAGAAAGGATTTTGCTATAAGATAGGTGGCAAGTGGTCAAAAAGTAGTGTAGTTGGATGG
CCTGCTGTAAAGGAAAGAATGAGACGAGCTGAGCCAGCAGCAGATGAGGTAGGAGCAGCATCTCGAGACCTAGAAAAACA
TGGAGCAATCACAAGTAGCAACACAGCAGCTAACAATGCTGCTTGTGCCTGGCTAGAAGCACAAGAGGAGGAGAAGGTAG
GTTTTCCAGTCACACCTCAGGTACCTTTAAGACCAATGACTTACAAGGCAGCTGTAGATCTTAGCCACTTTTTAAAAGAA
AAGGGGGGACTGGAAGGGCTAATTCACTCCCAACGAAGACAAGATATCCTTGATCTGTGGATCTACCACACACAAGGCTA
CTTCCCTGATTGGCAGAACTACACACCAGGACCAGGGATCAGATATCCACTGACCTTTAGATGGCGCTACAAGCTAGTAC
CAGTTGAGCCAGAGAAGTTAGAAGAAGCCAACAAAGGAGAGAACACCAGCTTGTTACACCCTGTGAGCCTGCATAGAATA
GATGACCCGGAGAGAGAAGTGTTAGAGTGGAGGTTTGACAGCCGCCTAGCATTTCATCACGTGGCCCGAGAGCTGCATCC
AGAGTACTTCAAGAACTGCTGATATCGAGCTTGCTACAAAGGACTTTCCGCTGAGGACTTTCCAGGGAGGCGTGGCCTAG
GCAGGACTGAGGAGTGGCGAGCCCTCAGATCCTGCATATAAGCAGCTGCTTTTTGCCTGTACTAGGTCTCTCTGGTTAGA
CCAGATCTGAGCCTAGGAGCTCTCTGGCTAACTAAGGAACCCACTGCTTAAGCCTCAATAAAGCTTGCCTTGAGTGCTTC
AAGTAGTGTGTGCCCATCTGTTGTGTGACTCTGGTAACTAGAGATCCCTCA
>5-33envLTR

```
--------------------------------------------------------------------------------
--------------------------------------------------------------------------------
--------------------------------------------------------------------------------
--------------------------------------------------------------------------------
--------------------------------------------------------------------------------
--------------------------------------------------------------------------------
--------------------------------------------------------------------------------
--------------------------------------------------------------------------------
--------------------------------------------------------------------------------
--------------------------------------------------------------------------------
--------------------------------------------------------------------------------
--------------------------------------------------------------------------------
--------------------------------------------------------------------------------
--------------------------------------------------------------------------------
--------------------------------------------------------------------------------
--------------------------------------------------------------------------------
--------------------------------------------------------------------------------
--------------------------------------------------------------------------------
--------------------------------------------------------------------------------
--------------------------------------------------------------------------------
--------------------------------------------------------------------------------
--------------------------------------------------------------------------------
--------------------------------------------------------------------------------
--------------------------------------------------------------------------------
--------------------------------------------------------------------------------
--------------------------------------------------------------------------------
--------------------------------------------------------------------------------
--------------------------------------------------------------------------------
--------------------------------------------------------------------------------
--------------------------------------------------------------------------------
--------------------------------------------------------------------------------
--------------------------------------------------------------------------------
--------------------------------------------------------------------------------
--------------------------------------------------------------------------------
--------------------------------------------------------------------------------
--------------------------------------------------------------------------------
--------------------------------------------------------------------------------
--------------------------------------------------------------------------------
--------------------------------------------------------------------------------
--------------------------------------------------------------------------------
--------------------------------------------------------------------------------
--------------------------------------------------------------------------------
--------------------------------------------------------------------------------
--------------------------------------------------------------------------------
--------------------------------------------------------------------------------
--------------------------------------------------------------------------------
--------------------------------------------------------------------------------
--------------------------------------------------------------------------------
--------------------------------------------------------------------------------
--------------------------------------------------------------------------------
--------------------------------------------------------------------------------
--------------------------------------------------------------------------------
--------------------------------------------------------------------------------
--------------------------------------------------------------------------------
--------------------------------------------------------------------------------
--------------------------------------------------------------------------------
--------------------------------------------------------------------------------
--------------------------------------------------------------------------------
--------------------------------------------------------------------------------
--------------------------------------------------------------------------------
--------------------------------------------------------------------------------
--------------------------------------------------------------------------------
--------------------------------------------------------------------------------
--------------------------------------------------------------------------------
--------------------------------------------------------------------------------
--------------------------------------------------------------------------------
--------------------------------------------------------------------------------
--------------------------------------------------------------------------------
--------------------------------------------------------------------------------
--------------------------------------------------------------------------------
--------------------------------------------------------------------------------
--------------------------------------------------------------------------------
--------------------------------------------------------------------------------
--------------------------------------------------------------------------------
--------------------------------------------------------------------------------
--------------------------------------------------------------------------------
--------------------------------------------------------------------------------
--------------------------------------------------------------------------------
--------------------------------------------------------------------------------
--------------------------------------------------------------------------------
--------------------------------------------------------------------------------
--------------------------------------------------------------------------------
--------------------------------------------------------------------------------
--------------------------------------------------------------------------------
--------------------------------------------------------------------------------
--------------------------------------------------------------------------------
--------------------------------------------------------------------------------
--------------------------------------------------------------------------------
--------------------------------------------------------------------------------
--------------------------------------------------------------------------------
--------------------------------------------------------------------------------
--------------------------------------------------------------------------------
--------------------------------------------------------------------------------
--------------------------------------------------------------------------------
```

------------------------GCTATTAACAAAAGATAGTAGTAATAACAACAATAAGTCCAAGATCTTCAGACCTA
GAGGAAGAGATATGAAAGACAATTAGAGAAGTGAATTATATAAATATNAAGTAGTAAAAATTGAACCATTAAAAGTAGCA
CCCACCAAGGCAAAGAGAAGAGTGGTGCAGAGAGAAAAAAGAGCAGTAAGAATAGGAGCTTTGTTCCTTAAGTTCTTAAG
AGCAGCAGGAAGCACTATAGGCGCAGCGTCAATGACGCTGACAGTACAGGCCAGACAATTATTGTCTAGTATAGTGCAGC
AGCAGAACAATTTGCTGAAGGCTATTGAGGCGCAACAGCATCTGTTGCAACTCACAGTCTGAGGCATCAAGCAGCTCCAG
GCAAGAATCCTGGCTGTAGAAAGATACCTAAAAGATCAACAGCTCCTAAGGATTTAAAGTTGCTCTAAAAAACTCATTTG
CACCACTGCTGTGCCTTAGAATGCTAGTTAAAGTAATAAATCTCTAGAACAGATTTAGAATCACACGACCTGGATAGAGT
AGGACAAAGAAATTAACAATTACACAAGCTTAATACACTCCTTAATTGAAGAATCGCAAAACCAGCAAGAAAAGAATGAA
CAAGAATTATTAGAATTAGATAAATAGGCAAGTTTATAAAATTAGTTTAACATAACAAATTAGCTGTAGTATATAAAATT
ATTCATAATGATAGTAAGAGGCTTAGTAAGTTTAAGAATAGTTTTTGCTGTACTTTCTGTAGTGAATAAAGTTAAGCAAA
GATATTCACCATTATCGTTTCAGACCCACCTCCCAATCCCGAGAAGACCCGACAGGCCCGAAAGAATAGAAGAAGAAGGT
AGAGAAAGAGACAGAGACAGATCCATTCGATTAGTGAACAGATCCTTAGCACTTATCTAAGACGATCTGCAGAGCCTGTG
CCTCTTCAGCTACCACCGCTTAAGAGACTTACTCTTGATTGTAACGAAGATTGTAGAACTTCTAAGACGCAGAAGGTGAG
AAGCCCTCAAATATTAGTAGAATCTCCTACAATATTAGAGTCAGGAGCTAAAGAATAGTGCTGTTAGCTTGCTCAATGCC
ACAGCTATAGCAGTAGCTGAGAAGACAGATAAAGTTATAGAAGTAGTACAAGAAGCTTATAAAGCTATTCGCCACATACC
TAGAAAAATAAGACAAGGCTTAGAAAAGATTTTGCTATAAGATAAGTGGCAAGTGGTCAAAAAGTAGTGTAGTTAGATAG
CCTGCTGTAAAAGAAAGAATGAGACGAGCTAAGCCAGCAGCAGATAAGGTAAGAGCAGCATCTCGAGACCTAGAAAAACA
TAGAGCAATCACAAGTAGCAACACAGCAGCTAACAATGCTGCTTGTGCCTAGCTAGAAGCACAAGAGGAGGAAAAAGTAA
GTTTTCCAGTCACACCTCAGGTACCTTTAAGACCAATGACTTACAAGGCAGCTGTAAATCTTAGCCACTTTTTAAAAGAA
AAGGGGGGACTGGAAGGGCTAATTCACTCCCAACGAAGACAAGATATCCTTGATCTGTAGATCTACCACACACAAGGCTA
CTTCCCTGATTGGCAGAACTACACACCAGGACCAGGGATCAGATATCCACTGACCTTTAGATGGCGCTACAAGCTAGTAC
CAGTTGAGCCAGAGAAGTTAGAAGAAGCCAACAAAGGAGAGAACACCAGCTTGTTACACCCTGTGAGCCTGCATAGAATA
GATGACCCGGAGAGAGAAGTGTTAGAGTGGAAGTTTGACAGCCGCCTAGCATTTCATCACGTGGCCCGAGAGCTGCATCC
GGAGTACTTCAAGAACTGCTGATATCGAGCTTGCTACAAAAGACTTTCCGCTGGGGACTTTCCAAGGAGGCGTGGCCTAG
GCAGGACTGAGAAGTGGCGAGCCCTCAGATCCTGCATATAAGCAGCTGCTTTTTGCCTGTACTAGGTCTCTCTAGTTAGA
CCAGATCTGAGCCTAGGAGCTCTCTGGCTAACTAAGGAACCCACTGCTTAAGCCTCAATAAAGCTTGCCTTAAGTGCTTC
AAGTAGTGTGTGCCCATCTGTTGTGTGACTCTGGTAACTAGAGATCCCTCA

>5-58envLTR

--AAAGCAATGTATGCCCCTCCCATCAGCGGACAAATTAG
ATGTTCATCAAATATTACAGGGCTGCTATTAACAAGAGATGGTGGTAATAACAACAATAGGTCCGAGATCTTCAGACCTG
GAGGAGGAGATATGAAGGACAATTGGAGAAGTGAATTATATAAATATAAAGTAGTAAAAATTGAACCATTAAGAGTAGCA
CCCACCAAGGCAAAGAGAAGAGTGGTGCAGAGAGAAAAAAGAGCAGTAGGAATAGGAGCTTTGTTCCTTAGGTTCTTAAG
AGCAGCAGGAAGCACTATAGGCGCAGCGTCAATGACGCTGACGGTACAGGCCAGACAATTATTGTCTGGTATAGTGCAGC
AGCAGAACAATTTGCTGAGGGCTATTGAGGCGCAACAGCATCTGTTGCAACTCACAGTCTAAGGCATCAAGCAGCTCCAG
GCAAGAATCCTGGCTGTAGAAAGATACCTAAAAGATCAACAGCTCCTGGGGATTTGGGGTTGCTCTAGAAAACTCATTTG
CACCACTGCTGTGCCTTAGAATGCTAGTTAGAGTAATAAATCTCTAGAACAGATTTAAAATCACACGACCTAGATAGAGT
AAGACAGAGAAATTAACAATTACACAAGCTTAATACACTCCTTAATTGAAGAATCGCAAAACCAGCAAGAAAAGAATGAA
CAAGAATTATTAGAATTAGATAAATGGGCAAGTTTGTAGAATTAGTTTAACATAACAAATTGGCTGTGGTATATAAAATT
ATTCATAATGATAGTAAGAGGCTTGGTAGGTTTAAGAATAGTTTTTGCTGTACTTTCTGTAGTGAATAGAGTTAGGCAAG
GATATTCACCATTATCGTTTCAGACCCACCTCCCAATCCCGAGGGGACCCGACAGGCCCGAAGGAATAGAAGAAGAAGGT
GGAGAGAGAGACAGAGACAGATCCATTCGATTAGTGAACGGATCCTTAGCACTTATCTAGGACGATCTGCGGAGCCTGTG
CCTCTTCAGCTACCACCGCTTAAGAGACTTACTCTTGATTGTAACGAGGATTGTAGAACTTCTAGGACGCTGAGGGTAGG
AAGCCCTCAAATATTGGTAGAATCTCCTACAATATTGGAGTCAGGAGCTAAAGAATAGTGCTGTTAGCTTGCTCAATGCC
ACAGCTATAGCAGTAGCTGAGAGGACAGATAAGGTTATAGAAGTAGTACAAGAAGCTTATAAAGCTATTCGCCACATACC
TAGAAGAATAAGACAGGGCTTAGAAAGGATTTTGCTATAAAATAGGTAGCAAGTGGTCAAAAAGTAGTGTGGTTAGATGG
CCTGCTGTAAAGGAAAGAATGAGACGAGCTAAGCCAGCAGCAGATGGGGTAGGAGCAGCATCTCAAGACCTAGAAAAACA
TGGAGCAATCACAAGTAGCAACACAGCAGCTAACAATGCTGCTTGTGCCTGGCTAGAAGCACAAGAGGAGGAGAAAGTAG
GTTTTCCAGTCACACCTCAGGTACCTTTAAGACCAATGACTTACAAGGCAGCTGTAGATCTTAGCCACTTTTTAAAAGAA
AAGGGGGGACTGGAAGGGCTAATTCACTCCCAACGAAGACAAGATATCCTTGATCTGTGGATCTACCACACACAAGGCTA
CTTCCCTGATTGGCAGAACTACACACCAGGACCAGGGATCAGATATCCACTGACCTTTGGATGGCGCTACAAGCTAGTAC
CAGTTGAGCCAGAGAAGTTAGAAGAAGCCAACAAAGGAGAGAACACCAGCTTGTTACACCCTGTGAGCCTGCATAGAATG
GATGACCCGGAGAGAGAAGTGTTAGAGTGGAGGTTTGACAGCCGCCTAGCATTTCATCACGTGGCCCGAGAGCTGCATCC
AGAGTACTTCAAGAACTGCTGATATCAAGCTTGCTACAAAGGACTTTCCGCTGGAGACTTTCCAGGGAGGCGTGGCCTAG
GCAGGACTAAGGAGTGGCGAGCCCTCAGATCCTGCATATAAGCAGCTGCTTTTTGCCTGTACTAGGTCTCTCTAGTTAGA
CCAGATCTGAGCCTAGGAGCTCTCTGGCTAACTAAGGAACCCACTGCTTAAGCCTCAATAAAGCTTGCCTTGAGTGCTTC
AAGTAGTGTGTGCCCATCTGTTGTGTGACTCTGGTAACTAGAGATCCCTCA

>5-69envLTR

------------------------GCTATTAACAAGAGATGGTGGTAATAACAACAATGGGTCCGAGATCTTCAGACCTG
GAGGAGGAGATATGAGGGACAATTGGAGAAGTGAATTATATAAATATAAAGTAGTAAAAATTGAACCATTAGGAGTAGCA
CCCACCAAGGCAAAGAGAAGAGTGGTGCAGAGAGAAAAAAGAGCAGTGGGAATAGGAGCTTTGTTCCTTAGGTTCTTAGG
AGCAGCAGGAAGCACTATGGGCGCAGCGTCAATGACGCTGACGGTACAGGCCAGACAATTATTGTCTGGTATAGTGCAGC
AGCAGAACAATTTGCTGAGGGCTATTGAGGCGCAACAGCATCTGTTGCAACTCACAGTCTGGGGCATCAAGCAGCTCCAG
GCAAGAATCCTGGCTGTGGAAAGATACCTAAAGGATCAACAGCTCCTGGGGATTTGGGGTTGCTCTGGAAAACTCATTTG
CACCACTGCTGTGCCTTGGAATGCTAGTTGGAGTAATAAATCTCTGGAACAGATTTGGAATCACACGACCTGGATGGAGT
GGGACAGAGAAATTAACAATTACACAAGCTTAATACACTCCTTAATTGAAGAATCGCAAAACCAGCAAGAAAAGAATGAA
CAAGAATTATTGGAATTAGATAAATGGGCAAGTTTGTAGAATTGGTTTAACATAACAAATTGGCTGTGGTATATAAAATT
ATTCATAATGATAGTAGGAGGCTTGGTAGGTTTAAGAATAGTTTTTGCTGTACTTTCTGTAGTGAATAGAGTTAGGCAGG
GATATTCACCATTATCGTTTCAGACCCACCTCCCAATCCCGAGGGGACCCGACAGGCCCGAAGGAATAGAAGAAGAAGGT
GGAGAGAGAGACAGAGACAGATCCATTCGATTAGTGAACGGATCCTTAGCACTTATCTGGGACGATCTGCGGAGCCTGTG
CCTCTTCAGCTACCANCGCTTGAGAGACTTACTCTTGATTGTAACGAGGATTGTGGAACTTCTGGGACGCAGGGGGTGGG
AAGCCCTCAAATATTGGTGGAATCTCCTACAATATTGGAGTCAGGAGCTAAAGAATAGTGCTGTTAGCTTGCTCAATGCC
ACAGCTATAGCAGTAGCTGAGGGGACAGATAGGGTTATAGAAGTAGTACAAGAAGCTTATAGAGCTATTCGCCACATACC
TAGAAGAATAAGACAGGGCTTGGAAAGGATTTTGCTATAAGATGGGTGGCAAGTGGTCAAAAAGTAGTGTGGTTGGATGG
CCTGCTGTAAGGGAAAGAATGAGACGAGCTGAGCCAGCAGCAGATGGGGTGGGAGCAGCATCTCGAGACCTAGAAAAACA
TGGAGCAATCACAAGTAGCAACACAGCAGCTAACAATGCTGCTTGTGCCTGGCTAGAAGCACAAGAGGAGGAGAAGGTGG
GTTTTCCAGTCACACCTCAGGTACCTTTAAGACCAATGACTTACAAGGCAGCTGTAGATCTTAGCCACTTTTTAAAAGAA
AAGGGGGGACTGGAAGGGCTAATTCACTCCCAACGAAGACAAGATATCCTTGATCTGTGGATCTACCACACACAAGGCTA
CTTCCCTGATTGGCAGAACTACACACCAGGACCAGGGATCAGATATCCACTGACCTTTGGATGGCGCTACAAGCTAGTAC
CAGTTGAGCCAGAGAAGTTAGAAGAAGCCAACAAAGGAGAGAACACCAGCTTGTTACACCCTGTGAGCCTGCATGGAATG
GATGACCCGGAGAGAGAAGTGTTAGAGTGGAGGTTTGACAGCCGCCTAGCATTTCATCACGTGGCCCGAGAGCTGCATCC
GGAGTACTTCAAGAACTGCTGATATCGAGCTTGCTACAAGGGACTTTCCGCTGAGGACTTTCCAGGGAGGCGTGGCCTGG
GCGGGACTGGGGAGTGGCGAGCCCTCAGATCCTGCATATAAGCAGCTGCTTTTTGCCTGTACTAGGTCTCTCTGGTTAGA
CCAGATCTGAGCCTAGGAGCTCTCTGGCTAACTAAGGAACCCACTGCTTAAGCCTCAATAAAGCTTGCCTTGAGTGCTTC
AAGTAGTGTGTGCCCATCTGTTGTGTGACTCTGGTAACTAGAGATCCCTCA

>5-74envLTR


```
--------------------------------------------------------------------------------
--------------------------------------------------------------------------------
--------------------------------------------------------------------------------
--------------------------------------------------------------------------------
--------------------------------------------------------------------------------
--------------------------------------------------------------------------------
--------------------------------------------------------------------------------
--------------------------------------------------------------------------------
--------------------------------------------------------------------------------
--------------------------------------------------------------------------------
--------------------------------------------------------------------------------
--------------------------------------------------------------------------------
--------------------------------------------------------------------------------
--------------------------------------------------------------------------------
--------------------------------------------------------------------------------
--------------------------------------------------------------------------------
--------------------------------------------------------------------------------
--------------------------------------------------------------------------------
--------------------------------------------------------------------------------
--------------------------------------------------------------------------------
--------------------------------------------------------------------------------
--------------------------------------------------------------------------------
--------------------------------------------------------------------------------
--------------------------------------------------------------------------------
--------------------------------------------------------------------------------
--------------------------------------------------------------------------------
--------------------------------------------------------------------------------
--------------------------------------------------------------------------------
--------------------------------------------------------------------------------
--------------------------------------------------------------------------------
--------------------------------------------------------------------------------
--------------------------------------------------------------------------------
--------------------------------------------------------------------------------
--------------------------------------------------------------------------------
--------------------------------------------------------------------------------
--------------------------------------------------------------------------------
--------------------------------------------------------------------------------
--------------------------------------------------------------------------------
--------------------------------------------------------------------------------
--------------------------------------------------------------------------------
--------------------------------------------------------------------------------
--------------------------------------------------------------------------------
--------------------------------------------------------------------------------
--------------------------------------------------------------------------------
--------------------------------------------------------------------------------
--------------------------------------------------------------------------------
--------------------------------------------------------------------------------
--------------------------------------------------------------------------------
--------------------------------------------------------------------------------
--------------------------------------------------------------------------------
--------------------------------------------------------------------------------
--------------------------------------------------------------------------------
--------------------------------------------------------------------------------
--------------------------------------------------------------------------------
--------------------------------------------------------------------------------
--------------------------------------------------------------------------------
--------------------------------------------------------------------------------
--------------------------------------------------------------------------------
--------------------------------------------------------------------------------
--------------------------------------------------------------------------------
--------------------------------------------------------------------------------
--------------------------------------------------------------------------------
--------------------------------------------------------------------------------
--------------------------------------------------------------------------------
--------------------------------------------------------------------------------
--------------------------------------------------------------------------------
--------------------------------------------------------------------------------
--------------------------------------------------------------------------------
--------------------------------------------------------------------------------
--------------------------------------------------------------------------------
--------------------------------------------------------------------------------
--------------------------------------------------------------------------------
--------------------------------------------------------------------------------
--------------------------------------------------------------------------------
--------------------------------------------------------------------------------
--------------------------------------------------------------------------------
--------------------------------------------------------------------------------
--------------------------------------------------------------------------------
--------------------------------------------------------------------------------
--------------------------------------------------------------------------------
--------------------------------------------------------------------------------
--------------------------------------------------------------------------------
--------------------------------------------------------------------------------
--------------------------------------------------------------------------------
--------------------------------------------------------------------------------
--GGAGGAAATATGAAGGACAATTAGAGAAGTGAATTATATAAATATAAAGTAGTAAAAATTAAACCATTAGGAGTAGCA
CCCACCAAGGCAAAGAGAAGAGTGGTGCAGAGAGAAAAAAGAGCAGTAGGAATAGGAGCTTTGTTCCTTAGGTTCTTAGG
AGCAGCAAGAAGCACTATAGGCGCAGCGTCAATGACGCTGACGGTACAGGCCAGACAATTATTGTCTGGTATAGTGCAGC
AGCAGAACAATTTGCTGAAGGCTATTGAGGCGCAACAGCATCTGTTGCAACTCACAGTCTAAGGCATCAAGCAGCTCCAG
GCAAGAATCCTGGCTGTAGAAAGATACCTAAAGGATCAACAGCTCCTGAGGATTTAGGGTTGCTCTAGAAAACTCATTTG
CACCACTGCTGTGCCTTAGAATGCTAGTTAGAGTAATAAATCTCTAGAACAGATTTAGAATCACACGACCTGGATGGAGT
AGGACAGAGAAATTAACAATTACACAAGCTTAATACACTCCTTAATTGAAGAATCGCAAAACCAGCAAGAAAAGAATGAA
CAAGAATTATTAGAATTAGATAAATAGGCAAGTTTGTAAAATTAGTTTAACATAACAAATTGGCTGTAGTATATAAAATT
ATTCATAATGATAGTAGGAGGCTTAGTAGGTTTAAGAATAGTTTTTGCTGTACTTTCTGTAGTGAATAAAGTTAGGCAAG
```

```
GATATTCACCATTATCGTTTCAGACCCACCTCCCAATCCCGAGGGGACCCGACAGGCCCGAAGGAATAGAAGAAGAAGGT
GGAAAGAAAGACAGAGACAGATCCATTCAATTAGTGAACGGATCCTTAGCACTTATCTAGGACGATCTGCGGAGCCTGTG
CCTCTTCAGCTACCACCGCTTAAGAGACTTACTCTTGATTGTAACGAGGATTGTAGAACTTCTAAGACGCAAGAGGTAGG
AAGCCCTCAAATATTAGTAGAATCTCCTACAATATTGGAGTCAGGAGCTAAAGAATAGTGCTGTTAGCTTGCTCAATGCC
ACAGCTATAGCAGTAGCTGAGGAGACAGATAAAGTTATAGAAGTAGTACAAGAAGCTTATAGAGCTATTCGCCACATACC
TAGAAGAATAAGACAAGGCTTAGAAAAGATTTTGCTATAAGATAAGTAGCAAGTGGTCAAAAAGTAGTGTAGTTAGATAG
CCTGCTGTAAGAGAAAGAATAAGACGAGCTGAGCCAGCAGCAAATAAGGTAAGAGCAGCATCTCAAGACCTAGAAAAACA
TGGAGCAATCACAAGTAGCAACACAGCAGCTAACAATGCTGCTTGTGCCTGGCTAGAAGCACAAGAGGAGGAGAAGGTAG
GTTTTCCAGTCACACCTCAGGTACCTTTAAGACCAATGACTTACAAGGCAGCTGTAGATCTTAGCCACTTTTTAAAAGAA
AAGGGGGGACTAGAAAGGCTAATTCACTCCTAACGAAGACAAGATATCCTTGATCTGTGGATCTACCACACACAAGGCTA
CTTCCCTGATTGGCAGAACTACACACCAAGACCAAAGATCAGATATCCACTGACCTTTAGATGGCGCTACAAGCTAGTAC
CAGTTGAGCCAGAGAAGTTAGAAGAAGCCAACAAAGGAGAGAACACCAGCTTGTTACACCCTGTGAGCCTGCATGGAATG
GATGACCCGGAGAGAGAAGTGTTAGAGTGGAGGTTTGACAGCCGCCTAGCATTTCATCACGTGGCCCGAGAGCTGCATCC
AAAGTACTTCAAGAACTGCTGATATCGAGCTTGCTACAAAAGACTTTCCGCTGAAGACTTTCCAGGGAGGCGTGGCCTAG
GCAGGACTAAGGAGTAGCGAGCCCTCAGATCCTGCATATAAGCAGCTGCTTTTTGCCTGTACTAGGTCTCTCTGGTTAGA
CCAGATCTGAGCCTAGGAGCTCTCTAGCTAACTAAGGAACCCACTGCTTAAGCCTCAATAAAGCTTGCCTTAAGTGCTTC
                AAGTAGTGTGTGCCCATCTGTTGTGTGACTCTAGTAACTAGAGATCCCTCA
                                   >5-78envLTR
--------------------------------------------------------------------------------
--------------------------------------------------------------------------------
--------------------------------------------------------------------------------
--------------------------------------------------------------------------------
--------------------------------------------------------------------------------
--------------------------------------------------------------------------------
--------------------------------------------------------------------------------
--------------------------------------------------------------------------------
--------------------------------------------------------------------------------
--------------------------------------------------------------------------------
--------------------------------------------------------------------------------
--------------------------------------------------------------------------------
--------------------------------------------------------------------------------
--------------------------------------------------------------------------------
--------------------------------------------------------------------------------
--------------------------------------------------------------------------------
--------------------------------------------------------------------------------
--------------------------------------------------------------------------------
--------------------------------------------------------------------------------
--------------------------------------------------------------------------------
--------------------------------------------------------------------------------
--------------------------------------------------------------------------------
--------------------------------------------------------------------------------
--------------------------------------------------------------------------------
--------------------------------------------------------------------------------
--------------------------------------------------------------------------------
--------------------------------------------------------------------------------
--------------------------------------------------------------------------------
--------------------------------------------------------------------------------
--------------------------------------------------------------------------------
--------------------------------------------------------------------------------
--------------------------------------------------------------------------------
--------------------------------------------------------------------------------
--------------------------------------------------------------------------------
--------------------------------------------------------------------------------
--------------------------------------------------------------------------------
--------------------------------------------------------------------------------
--------------------------------------------------------------------------------
--------------------------------------------------------------------------------
--------------------------------------------------------------------------------
--------------------------------------------------------------------------------
--------------------------------------------------------------------------------
--------------------------------------------------------------------------------
--------------------------------------------------------------------------------
--------------------------------------------------------------------------------
--------------------------------------------------------------------------------
--------------------------------------------------------------------------------
--------------------------------------------------------------------------------
--------------------------------------------------------------------------------
--------------------------------------------------------------------------------
--------------------------------------------------------------------------------
--------------------------------------------------------------------------------
--------------------------------------------------------------------------------
--------------------------------------------------------------------------------
--------------------------------------------------------------------------------
--------------------------------------------------------------------------------
--------------------------------------------------------------------------------
--------------------------------------------------------------------------------
--------------------------------------------------------------------------------
--------------------------------------------------------------------------------
--------------------------------------------------------------------------------
--------------------------------------------------------------------------------
--------------------------------------------------------------------------------
--------------------------------------------------------------------------------
--------------------------------------------------------------------------------
--------------------------------------------------------------------------------
--------------------------------------------------------------------------------
--------------------------------------------------------------------------------
--------------------------------------------------------------------------------
--------------------------------------------------------------------------------
--------------------------------------------------------------------------------
--------------------------------------------------------------------------------
--------------------------------------------------------------------------------
--------------------------------------------------------------------------------
--------------------------------------------------------------------------------
--------------------------------------------------------------------------------
```


---AAAAATTGAACCATTAAAAGTAGCA
CCCACCAAGGCAAAGAGAAGAGTAGTGCAGAGAGAAAAAAGAGCAGTANGAATAAAAGCTTTGTTCCTTAAGTTCTTAGG
AGCAGCAAGAAGCACTATAGGCGCAGCGTCAATGACGCTGACAGTACAGGCCAGACAATTATTGTCTAGTATAGTGCAGC
AGCAGAACAATTTGCTGAAGGCTATTGAGGCGCAACAGCATCTGTTGCAACTCACAGTCTAAGGCATCAAGCAGCTCCAG
GCAAGAATCCTGGCTGTAGAAAGATACCTAAAAGATCAACAGCTCCTGAGGATTTGAGGTTGCTCTAGAAAACTCATTTG
CACCACTGCTGTGCCTTAGAATGCTAGTTAAAGTAATAAATCTCTAGAACAGATTTAAAATCACACGACCTAGATAGAGT
AGGACAGAGAAATTAACAATTACACAAGCTTAATACACTCCTTAATTGAAGAATCGCAAAACCAGCAAGAAAAGAATGAA
CAAAAATTATTAGAATTAGATAAATAGGCAAGTTTGTAGAATTAGTTTAACATAACAAATTAGCTGTAGTATATAAAATT
ATTCATAATGATAGTAGGAGGCTTAGTAAGTTTAAAAATAGTTTTTGCTGTACTTTCTGTAATGAATAAAGTTAGGCAAA
GATATTCACCATTATCGTTTCAGACCCACCTCCCAATCCCGAGAGGACCCGACAGGCCCGAAGGAATAGAAGAAGAAGGT
AGAAAGAGAGACAGAGACAGATCCATTCGATTAGTGAACGGATCCTTAGCACTTATCTAAGACGATCTGCAGAGCCTGTG
CCTCTTCAGCTACCACCGCTTGAAAGACTTACTCTTGATTGTAACGAAGATTGTAGAACTTCTAAGACGCAGAAGGTAAG
AAGCCCTCAAATATTAGTAGAATCTCCTACAATATTGGAGTCAGGAGCTAAAGAATAGTGCTGTTAGCTTGCTCAATGCC
ACAGCTATAGCAGTAGCTGAAAAGACAGATAAAGTTATAGAAGTAGTACAAGAAGCTTATAGAGCTATTCGCCACATACC
TAGAAGAATAAGACAAAGCTTAGAAAAGATTTTGCTATAAGATAAGTGGCAAGTAGTCAAAAAGTAGTGTAGTTAGATGG
CCTGCTGTAAAAGAAAGAATAAGACGAGCTGAGCCAGCAGCAGATAAGGTAGGAGCAGCATCTCGAGACCTAGAAAAACA
TGAAGCAATCACAAGTAGCAACACAGCAGCTAACAATGCTGCTTGTGCCTGGCTAAAAGCACAAGAAGAAGAGAAAGTAA
GTTTTCCAGTCACACCTCAGGTACCTTTAAGACCAATGACTTACAAGGCAGCTGTAGATCTTAGCCACTTTTTAAAAGAA
AAGGGGGGACTGGAAGGGCTAATTCACTCCCAACGAAGACAAGATATCCTTGATCTGTAGATCTACCACACACAAGGCTA
CTTCCCTGATTGGCAGAACTACACACCAGGACCAAGGATCAGATATCCACTGACCTTTGGATGGCGCTACAAGCTAGTAC
CAGTTGAGCCAAAGAAGTTAGAAGAAGCCAACAAAGGAGAGAACACCAGCTTGTTACACCCTGTGAGCCTGCATAGAATG
GATGACCCAGAGAGAGAAGTGTTAGAGTGGAGGTTTGACAGCCGCCTAGCATTTCATCACGTGGCCCAAGAGCTGCATCC
GGAGTACTTCAAGAACTGCTGATATCGAGCTTGCTACAAGGGACTTTCCGCTGAGGACTTTCCAAGGAGGCGTGGCCTGG
GCAGGACTGGGGAGTGGCGAGCCCTCAGATCCTGCATATAAGCAGCTGCTTTTTGCCTGTACTAGGTCTCTCTAGTTAGA
CCAGATCTGAGCCTAGGAGCTCTCTGGCTAACTAAGGAACCCACTGCTTAAGCCTCAATAAAGCTTGCCTTGAGTGCTTC
AAGTAGTGTGTGCCCATCTGTTGTGTGACTCTGGTAACTAGAGATCCCTCA

>6-21envLTR


```
--------------------------------------------------------------------------------
--------------------------------------------------------------------------------
--------------------------------------------------------------------------------
--------------------------------------------------------------------------------
--------------------------------------------------------------------------------
--------------------------------------------------------------------------------
--------------------------------------------------------------------------------
--------------------------------------------------------------------------------
--------------------------------------------------------------------------------
--------------------------------------------------------------------------------
--------------------------------------------------------------------------------
--------------------------------------------------------------------------------
--------------------------------------------------------------------------------
--------------------------------------------------------------------------------
--------------------------------------------------------------------------------
--------------------------------------------------------------------------------
--------------------------------------------------------------------------------
--------------------------------------------------------------------------------
--------------------------------------------------------------------------------
--------------------------------------------------------------------------------
--------------------------------------------------------------------------------
--------------------------------------------------------------------------------
--------------------------------------------------------------------------------
--------------------------------------------------------------------------------
--------------------------------------------------------------------------------
--------------------------------------------------------------------------------
--------------------------------------------------------------------------------
--------------------------------------------------------------------------------
--------------------------------------------------------------------------------
--------------------------------------------------------------------------------
--------------------------------------------------------------------------------
--------------------------------------------------------------------------------
--------------------------------------------------------------------------------
--------------------------------------------------------------------------------
--------------------------------------------------------------------------------
--------------------------------------------------------------------------------
--------------------------------------------------------------------------------
--------------------------------------------------------------------------------
--------------------------------------------------------------------------------
--------------------------------------------------------------------------------
--------------------------------------------------------------------------------
--------------------------------------------------------------------------------
--------------------------------------------------------------------------------
--------------------------------------------------------------------------------
--------------------------------------------------------------------------------
--------------------------------------------------------------------------------
--------------------------------------------------------------------------------
--------------------------------------------------------------------------------
--------------------------GCANAGAGAAAAAAGAGCAGTAAGAATAAGAGCTTTGTTCCTTAGGTTCTTAAG
AGCAGCAGGAAGCACTATAGGCGCAGCGTCAATGACGCTGACGGTACAGGCCAGACAATTATTGTCTGGTATAGTGCAGC
AGCAGAACAATTTGCTGAGGGCTATTAAGGCGCAACAGCATCTGTTGCAACTCACAGTCTAAGGCATCAAGCAGCTCCAG
GCAAGAATCCTGGCTGTGGAAAGATACTTAAAAGATCAACAGCTCCTGGGGATTTGGGGTTGCTCTAGAAAACTCATTTG
CACCACTGCTGTGCCTTAGAATGCTAGTTAGAGTAATAAATCTCTAGAACAGATTTAGAATCACACGACCTAGATAGAGT
AGGACAGAGAAATTAACAATTACACAAGCTTAATACACTCCTTAATTGAAGAATCGCAAAACCAGCAAGAAAAGAATGAA
CAAGAATTATTAGAATTAGATAAATAGGCAAGTTTGTAGAATTAGTTTAACATAACAAATTGGCTGTGGTATATAAAATT
ATTCATAATGATAGTAAGAGGCTTAGTAAGTTTAAGAATAGTTTTTGCTGTACTTTCTGTAGTGAATAAAGTTAGGCAAA
AATATTCACCATTATCGTTTCAGACCCACCTCCCAATCCCGAGGGGACCCGACAGGCCCGAAAGAATAGAAGAAGAAGGT
AAAGAGAGAGACAGAGACAGATCCATTCGATTAGTGAACGGATCCTTAGCACTTATCTAGGACGATCTGCGGAGCCTGTG
CCTCTTCAGCTACCACCGCTTGAGAGACTTACTCTTGATTGTAACGAGGATTGTAGAACTTCTAGGACGCAGGAAGTAAG
AAGCCCTCAAATATTAGTAGAATCTCCTACAATATTAGAGTCAGGAGCTAAAGAATAGTGCTGTTAGCTTGCTCAATGCC
ACAGCTATAGCAGTAGCTGAGGGGACAGATAAGGTTATAGAAGTAGTACAAGAAGCTTATAGAGCTATTCGCCACATACC
TAGAAGAATAAGACAAGGCTTAGAAAGGATTTTGCTATAAGATAAGTGGCAAGTGGTCAAAAAGTAGTGTGGTTAGATGG
CCTGCTGTAAAAGAAAGAATGAGACGAGCTGAGCCAGCAGCAGATGGGGTGGGAGCAGCATCTCGAGACCTAGAAAAACA
TGGAGCAATCACAAGTAGCAACACAGCAGCTAACAATGCTGCTTGTGCCTGGCTAGAAGCACAAGAAGAGGAGAAGGTAG
GTTTTCCAGTCACACCTCAAGTACCTTTAAGACCAATGACTTACAAGGCAGCTGTAGATCTTAGCCACTTTTTAAAAGAA
AAGGGGGGACTGGAAGGGCTAATTCACTCCCAACGAAGACAAGATATCCTTGATCTGTGGATCTACCACACACAAGGCTA
CTTCCCTGATTGGCAGAACTACACACCAGGACCAGGGATCAGATATCCACTGACCTTTGGATGGCGCTACAAGCTAGTAC
CAGTTGAGCCAGAGAAGTTAGAAGAAGCCAACAAAGGAGAGAACACCAGCTTGTTACACCCTGTGAGCCTGCATGGAATG
GATGACCCGGAGAAAGAAGTGTTAGAGTGGAAGTTTGACAGCCGCCTAGCATTTCATCNCGTGGCCCGAGAGCTGCATCC
GGAGTACTTCAAGAACTGCTGATATCGAGCTTGCTACAAAGGACTTTCCGCTGAGGACTTTCCAGGGAGGCGTGGCCTAG
GCAGGACTGGGGAGTGGCGAGCCCTCAGATCCTGCATATAAGCAGCTGCTTTTTGCCTGTACTAGGTCTCTCTAGTTAGA
CCAGATCTGAGCCTAGGAGCTCTCTGGCTAACTAGGGAACCCACTGCTTAAGCCTCAATAAAGCTTGCCTTGAGTGCTTC
               AAGTAGTGTGTGCCCATCTGTTGTGTGACTCTAGTAACTAGAGATCCCTCA
                                   >6-24envLTR
--------------------------------------------------------------------------------
--------------------------------------------------------------------------------
--------------------------------------------------------------------------------
--------------------------------------------------------------------------------
--------------------------------------------------------------------------------
--------------------------------------------------------------------------------
--------------------------------------------------------------------------------
--------------------------------------------------------------------------------
--------------------------------------------------------------------------------
--------------------------------------------------------------------------------
--------------------------------------------------------------------------------
--------------------------------------------------------------------------------
--------------------------------------------------------------------------------
--------------------------------------------------------------------------------
--------------------------------------------------------------------------------
--------------------------------------------------------------------------------
--------------------------------------------------------------------------------
--------------------------------------------------------------------------------
--------------------------------------------------------------------------------
--------------------------------------------------------------------------------
```


-------AGGCAAAGAGAAGAGTGGTGCAGAGAGAAAAAAGAGCAGTAAGAATAAGNGCTTTGTTCCTTAAGTTCTTAAG
AGCAGCAAGAAGCACTATAGGCGCAGCGTCAATGACGCTGACGGTACAGGCCAGACAATTATTGTCTAGTATAGTGCAGC
AGCAGAACAATTTGCTGAAGGCTATTGAGGCGCAACAGCATCTGTTGCAACTCACAGTCTAGGGCATCAAGCAGCTCCAG
GCAAGAATCCTGGCTGTAGAAAGATACCTAAAGGATCAACAGCTCCTAAAGATTTAAAGTTGCTCTAGAAAACTCATTTG
CACCACTGCTGTGCCTTAGAATGCTAGTTAGAGTAATAAATCTCTAGAACAGATTTAAAATCACACGACCTGGATGGAGT
GGGACAGAGAAATTAACAATTACACAAGCTTAATACACTCCTTAATTAAAAAATCGCAAAACCAGCAAGAAAAGAATGAA
CAAGAATTATTAGAATTAGATAAATAGGCAAGTTTGTAGAATTAGTTTAACATAACAAATTAGCTGTAGTATATAAAATT
ATTCATAATAATAGTAAGAGGCTTAGTAAGTTTAAGAATAGTTTTTGCTGTACTTTCTGTAGTGAATAAAGTTAGGCAAA
GATATTCACCATTATCGTTTCAAACCCACCTCCCAATCCCAAGAGGACCCGACAGGCCCGAAAGAATAGAAGAAAAAAGT
AGAAAAAAAGACAGAGACAGATCCATTCAATTAGTGAACAGATCCTTAGCACTTATCTAGGACGATCTGCGGAGCCTGTG
CCTCTTCAGCTACCACCGCTTAAGAGACTTACTCTTGATTGTAACGAAGATTGTAGAACTTCTAGGACGCAGGAGGTAAG
AAGCCCTCAAATATTGGTAGAATCTCCTACAATATTAGAGTCAGGAGCTAAAGAATAGTGCTGTTAGCTTGCTCAATGCC
ACAGCTATAGCAGTAGCTGAAAAGACAGATAAAGTTATAGAAGTAGTACAAAAAGCTTATAGAGCTATTCGCCACATACC
TAGAAGAACAAGACAAGGCTTAAAAAGAATTTTGCTATAAGATAAGTGGCAAGTGGTCAAAAAGTAGTGTGGTTAAATAG
CCTGCTGTAAAGGAAAGAATGAGACGAGCTGAGCCAGCAGCAGATAAGGTAGGAGCAGCATCTCAAGACCTAGAAAAACA
TAGAGCAATCACAAGTAGCAACACAGCAGCTAACAATGCTGCTTGTGCCTAGCTAGAAGCACAAGAAGAAAAGAAAGTAA
GTTTTCCAGTCACACCTCAGGTACCTTTAAGACCAATGACTTACAAGGCAGCTGTAGATCTTAGCCACTTTTTAAAAGAA
AAGGGGAGACTAGAAAGGCTAATTCACTCCCAACGAAGACAAGATATCCTTGATCTGTAGATCTACCACACACAAGGCTA

```
CTTCCCTGATTAGCAGAACTACACACCAGGACCAGGGATCAGATATCCACTAACCTTTAGATGGCGCTACAAGCTAGTAC
CAGTTGAGCCAGAGAAGTTAGAAGAAGCCAACAAAGGAGAGAACACCAGCTTGTTACACCCTGTGAGCCTGCATAGAATG
GATGACCCAGAGAGAGAAGTGTTAAAGTGGAAGTTTGACAGCCACCTAGCATTTCATCACGTAGCCCGAGAGCTGCATCC
AGAGTACTTCAAGAACTGCTGATATCGAGCTTGCTACAAAAGACTTTCCGCTAAGGACTTTCCAAAGAGGCGTGGCCTAG
GCAGGACTGGGGAGTGGCGAGCCCTCAGATCCTGCATATAAGCAGCTGCTTTTTGCCTGTACTAAGTCTCTCTGGTTAGA
CCAGATCTGAGCCTGGGAGCTCTCTGGCTAACTAAGGAACCCACTGCTTAAGCCTCAATAAAGCTTGCCTTAAGTGCTTC
AAGTAGTGTGTGCCCATCTGTTGTGTGACTCTGGTAACTAGAGATCCCTCA
>6-31envLTR
--------------------------------------------------------------------------------
--------------------------------------------------------------------------------
--------------------------------------------------------------------------------
--------------------------------------------------------------------------------
--------------------------------------------------------------------------------
--------------------------------------------------------------------------------
--------------------------------------------------------------------------------
--------------------------------------------------------------------------------
--------------------------------------------------------------------------------
--------------------------------------------------------------------------------
--------------------------------------------------------------------------------
--------------------------------------------------------------------------------
--------------------------------------------------------------------------------
--------------------------------------------------------------------------------
--------------------------------------------------------------------------------
--------------------------------------------------------------------------------
--------------------------------------------------------------------------------
--------------------------------------------------------------------------------
--------------------------------------------------------------------------------
--------------------------------------------------------------------------------
--------------------------------------------------------------------------------
--------------------------------------------------------------------------------
--------------------------------------------------------------------------------
--------------------------------------------------------------------------------
--------------------------------------------------------------------------------
--------------------------------------------------------------------------------
--------------------------------------------------------------------------------
--------------------------------------------------------------------------------
--------------------------------------------------------------------------------
--------------------------------------------------------------------------------
--------------------------------------------------------------------------------
--------------------------------------------------------------------------------
--------------------------------------------------------------------------------
--------------------------------------------------------------------------------
--------------------------------------------------------------------------------
--------------------------------------------------------------------------------
--------------------------------------------------------------------------------
--------------------------------------------------------------------------------
--------------------------------------------------------------------------------
--------------------------------------------------------------------------------
--------------------------------------------------------------------------------
--------------------------------------------------------------------------------
--------------------------------------------------------------------------------
--------------------------------------------------------------------------------
--------------------------------------------------------------------------------
--------------------------------------------------------------------------------
--------------------------------------------------------------------------------
--------------------------------------------------------------------------------
--------------------------------------------------------------------------------
--------------------------------------------------------------------------------
--------------------------------------------------------------------------------
--------------------------------------------------------------------------------
--------------------------------------------------------------------------------
--------------------------------------------------------------------------------
--------------------------------------------------------------------------------
--------------------------------------------------------------------------------
--------------------------------------------------------------------------------
--------------------------------------------------------------------------------
--------------------------------------------------------------------------------
--------------------------------------------------------------------------------
--------------------------------------------------------------------------------
--------------------------------------------------------------------------------
--------------------------------------------------------------------------------
--------------------------------------------------------------------------------
--------------------------------------------------------------------------------
--------------------------------------------------------------------------------
--------------------------------------------------------------------------------
--------------------------------------------------------------------------------
--------------------------------------------------------------------------------
--------------------------------------------------------------------------------
--------------------------------------------------------------------------------
--------------------------------------------------------------------------------
--------------------------------------------------------------------------------
--------------------------------------------------------------------------------
--------------------------------------------------------------------------------
--------------------------------------------------------------------------------
--------------------------------------------------------------------------------
--------------------------------------------------------------------------------
--------------------------------------------------------------------------------
--------------------------------------------------------------------------------
--------------------------------------------------------------------------------
--------------------------------------------------------------------------------
--------------------------------------------------------------------------------
--------------------------------------------------------------------------------
--------------------------------------------------------------------------------
--------------------------------------------------------------------------------
```


---ATAACAACAATAGGTCCGAGATCTTCANACCTG
GAGGAGGAGATATGAAGGACAATTGGAGAAGTGAATTATATAAATATAAAGTAGTAAAAATTAAACTATTAGGAGTAGCA
CCCACCAAGGCAAAGAGAAGAGTGGTGCAGAGAGAAAAAAGAGCAGTGGGAATAGGAGCTTTGTTCCTTAGGTTCTTAAG
AGCAGCAGGAAGCACTATAGGCGCAGCGTCAATGACGCTGACGGTACAGGCCAGACAATTATTGTCTGGTATAGTGCAGC
AGCAGAACAATTTGCTGAGGGCTATTGAGGCGCAACAGCATCTGTTGCAACTCACAGTCTAGGGCATCAAGCAGCTCCAG
GCAAGAATCCTAGCTGTAGAAAGATACCTAAAGGATCAACAGCTCCTGAGGATTTAGGGTTGCTCTAGAAAACTCATTTG
CACCACTGCTGTGCCTTAGAATGCTAGTTAGAGTAATAAATCTCTAGAACAGATTTAGAATCACACGACCTGGATGGAGT
GGGACAGAGAAATTAACAATTACACACGCTTAATACACTCCTTAATTGAAGAATCGCAAAACCAGCAAGAAAAGAATGAA
CAAGAATTATTAGAATTAGATAAATAGGCAAGTTTGTAGAATTGGTTTAACATAACAAATTGGCTGTAGTATATAAAATT
ATTCATAATGATAGTAGGAGGCTTAGTAGGTTTAAGAATAGTTTTTGCTGTACTTTCTGTAGTGAATAGAGTTAGGCAAG
GATATTCACCATTATCGTTTCAGACCCACCTCCCAATCCCGAGAGGACCCGACAGGCCCGAAGGAATAGAAGAAGAAGGT
GGAGAGAGAGACAGAGACAGATCCATTCGATTAGTGAACAGATCCTTAGCACTTATCTGGGACGATCTGCGGAGCCTGTG
CCTCTTCAGCTACCACCGCTTGAGAGACTTACTCTTGATTGTAACGAGGATTGTAGAACTTCTAAGACGCAGAAAGTAGG
AAGCCCTCAAATATTGGTGGAATCTCCTACAATATTGGAGTCAGGAGCTAAAGAATAGTGCTGTTAGCTTGCTCAATGCC
ACAGCTATAGCAGTAGCTGAGGGGACAGATAGGGTTATAGAAGTAGTACAAGAAGCTTATAGAGCTATTCGCCACATACC
TAGAAGAATAAGACAGGGCTTGGAAAGGATTTTGCTATAAGATGGGTGGCAAGTGGTCAAAAAGTAGTGTGGTTGGATGG
CCTGCTGTAAGGGAANGAATGAGACGAGCTGAGCCAGCAGCAGATGGGGTGGGAGCAGCATCTCGAGACCTAGAAAAACA
TGGAGCAATCACAAGTAGCAACACAGCAGCTAACAATGCTGCTTGTGCCTGGCTAGAAGCACAAGAGGAGGAGAAGGTGG
GTTTTCCAGTCACACCTCAGGTACCTTTAAGACCAATGACTTACAAGGCAGCTGTAGATCTTAGCCACTTTTTAAAAGAA
AAGGGGGGACTGGAAGGGCTAATTCACTCCCAACGAAGACAAGATATCCTTGATCTGTGGATCTACCACACACAAGGCTA
CTTCCCTGATTGGCAGAACTACACACCAGGACCAAGGATCAGATATCCACTGACCTTTGGATGGCGCTACAAGCTAGTAC
CAGTTGAGCCAGAGAAGTTAGAAGAAGCCAACAAAGGAGAGAACACCAGCTTGTTACACCCTGTGAGCCTGCATAGAATG
GATGACCCGGAGAGAGAAGTGTTAGAGTGGAGGTTTGACAGCCGCCTAGCATTTCATCACGTGGCCCAAGAGCTGCATCC
GGAGTACTTCAAGAACTGCTGATATCGAGCTTGCTACAAGGGACTTTCCGCTGAAGACTTTCCAGGGAGGCGTGGCCTGG
GCGGGACTGGGGAGTGGCGAGCCCTCAGATCCTGCATATAAGCAGCTGCTTTTTGCCTGTACTAGGTCTCTCTGGTTAGA
CCAGATCTGAGCCTAGGAGCTCTCTGGCTAACTAAGGAACCCACTGCTTAAGCCTCAATAAAGCTTGCCTTGAGTGCTTC
AAGTAGTGTGTGCCCATCTGTTGTGTGACTCTGGTAACTAGAGATCCCTCA
>6-32envLTR


```
--------------------------------------------------------------------------------
--------------------------------------------------------------------------------
--------------------------------------------------------------------------------
--------------------------------------------------------------------------------
--------------------------------------------------------------------------------
--------------------------------------------------------------------------------
--------------------------------------------------------------------------------
--------------------------------------------------------------------------------
--------------------------------------------------------------------------------
--------------------------------------------------------------------------------
--------------------------------------------------------------------------------
--------------------------------------------------------------------------------
--------------------------------------------------------------------------------
--------------------------------------------------------------------------------
--------------------------------------------------------------------------------
--------------------------------------------------------------------------------
--------------------------------------------------------------------------------
--------------------------------------------------------------------------------
--------------------------------------------------------------------------------
--------------------------------------------------------------------------------
--------------------------------------------------------------------------------
--------------------------------------------------------------------------------
--------------------------------------------------------------------------------
--------------------------------------------------------------------------------
--------------------------------------------------------------------------------
--------------------------------------------------------------------------------
--------------------------------------------------------------------------------
--------------------------------------------------------------------------------
--------------------------------------------------------------------------------
--------------------------------------------------------------------------------
--------------------------------------------------------------------------------
--------------------------------------------------------------------------------
--------------------------------------------------------------------------------
--------------------------------------------------------------------------------
--------------------------------------------------------------------------------
--------------------------------------------------------------------------------
------------------------GCTATTAACAACAGATGGTAGTAATAACAACAATATGTCCGAGATCTTCAGACCTA
GAGGAGAAGATATGAAGGACAATTAGAGAAGTGAATTATATAAATATAAAGTAATAAAAATTGAACCATTATGAGTAGCA
CCCACCAAGGCAAAGAGAAGAGTAGTGCAAAGAGAAAAAAGAGCAGTAGGAATAA-------------------------
--------------------------------------------------------------------------------
--------------------------------------------------------------------------------
---------------------------------------------------------AGTTGCTCTAGAAAACTCATTTG
CACCACTGCTGTGCCTTAGAATGCTAGTTAGAGTAATAAATCTCTAGAACAGATTTAGAATCACACGACCTAGATAGAGT
AGGACAGAGAAATTAACAATTACACAAGCTTAATACACTCCTTAATTGAAGAATCGCAAAACCAGCAAGAAAAGAATGAA
CAAGAATTATTAGAATTAAATAAATAGGCAAGTTTGTAGAATTAGTTTAACATAACAAATTGGCTGTAGTATATAAAATT
ATTCATAATGATAGTAAGAGGCTTAGTAAGTTTAAGAATAGTTTTTGCTATACTTTCTGTAGTGAATAAAGTTAGGCAAA
GATATTCACCATTATCGTTTCAGACCCACCTCCCAATCCCGAAAAGACCCGACAGGCCCGAAGGAATAGAAGAAGAAGGT
AGAGAGAAAGACAGAGACAGATCCATTCGATTAGTGAACAGATCCTTAGCACTTATCTAGGACGATCTGCGGAGCCTGTG
CCTCTTCAGCTACCACCGCTTGAGAGACTTACTCTTGATTGTAACGAGGATTGTAGAACTTCTAGGACGCAGGGGGTAGG
AAGCCCTCAAATATTAGTAGAATCTCCTACAATATTGAAGTCAAGAGCTAAAGAATAGTGCTGTTAGCTTGCTCAATGCC
ACAGCTATAGCAGTAGCTGAGAGGACAGATAAAGTTATAAAAGTAGTACAAGAAGCTTATAGAGCTATTCGCCACATACC
TAGAAAAATAAGACAAGGCTTAAAAAAGATTTTGCTATAAGATAGGTGGCAAGTAGTCAAAAAGTAGTGTAGTTAGATAG
CCTGCTGTAAGGGAAAGAATGAGACGAGCTAAGCCAGCAGCAGATGGGGTGGGAGCAGCATCTCGAGACCTAGAAAAACA
TGGAGCAATCACAAGTAGCAACACAGCAGCTAACAATGCTGCTTGTGCCTGGCTAGAAGCACAAGAAGAGGAGAAAGTAA
GTTTTCCAGTCACACCTCAGGTACCTTTAAAACCAATGACTTACAANGCAGCTGTAAATCTTAGCCACTTTTTAAAAGAA
AAGGGGGGACTGGAAGGGCTAATTCACTCCCAACGAAGACAAGATATCCTTGATCTGTGGATCTACCACACACAAGGCTA
CTTCCCTGATTGGCAGAACTACACACCAGGACCAAGGATCAGATATCCACTGACCTTTGGATGGCGCTACAAGCTAGTAC
CAGTTGAGCCAGAGAAGTTAGAAGAAGCCAACAAAGGAGAGAACACCAGCTTGTTACACCCTGTGAGCCTGCATGGAATG
GATGACCCGGAGAGAGAAGTGTTAGAGTGGAGGTTTGACAGCCGCCTAGCATTTCATCACGTGGCCCGAGAGCTGCATCC
GGAGTACTTCAAGAACTGCTGATATCGAGCTTGCTACAAGGGACTTTCCGCTGAAGACTTTCCAGGGAGGCGTGGCCTGA
GCAGGACTGAAGAGTGGCGAGCCCTCAGATCCTGCATATAAGCAGCTGCTTTTTGCCTGTACTAGGTCTCTCTGGTTAGA
CCAGATCTGAGCCTAGGAGCTCTCTGGCTAACTAGGGAACCCACTGCTTAAGCCTCAATAAAGCTTGCCTTAAGTGCTTC
AAGTAGTGTGTGCCCATCTGTTGTGTGACTCTAGTAACTAGAGATCCCTCA
>6-51envLTR
--------------------------------------------------------------------------------
--------------------------------------------------------------------------------
--------------------------------------------------------------------------------
--------------------------------------------------------------------------------
--------------------------------------------------------------------------------
--------------------------------------------------------------------------------
--------------------------------------------------------------------------------
--------------------------------------------------------------------------------
--------------------------------------------------------------------------------
--------------------------------------------------------------------------------
--------------------------------------------------------------------------------
--------------------------------------------------------------------------------
--------------------------------------------------------------------------------
--------------------------------------------------------------------------------
--------------------------------------------------------------------------------
--------------------------------------------------------------------------------
--------------------------------------------------------------------------------
--------------------------------------------------------------------------------
--------------------------------------------------------------------------------
--------------------------------------------------------------------------------
--------------------------------------------------------------------------------
--------------------------------------------------------------------------------
--------------------------------------------------------------------------------
--------------------------------------------------------------------------------
--------------------------------------------------------------------------------
--------------------------------------------------------------------------------
--------------------------------------------------------------------------------
--------------------------------------------------------------------------------
--------------------------------------------------------------------------------
--------------------------------------------------------------------------------
```


------------------------GCTATTAACAAGAGATGGTGGTAATAACAACAATAGGTCCGAGATCTTCAGACCTG
GAGGAGGAGATATGAAAGACAATTGGAGAAGTGAATTATATAAATATAAAGTAGTAAAAATTGAACCATTAGGAGTAGCA
CCCACCAAGGCAAAGAGAAGAGTGGTGCAGAGAGAAAAAAGAGCAGTAGGAATAAGAGCTTTGTTCCTTAGGTTCTTAGG
AGCAGCAGGAAGCACTATAGGCGCAGCGTCAATGACGCTGACGGTACAGGCCAGACAATTATTGTCTGGTATAGTGCAGC
AGCAGAACAATTTGCTGAGGGCTATTGAGGCGCAACAGCATCTGTTGCAACTCACAGTCTAAGGCATCAAGCAGCTCCAG
GCAAGAATCCTGGCTGTGGAAAGATACCTAAAGGATCAACAGCTCCTAGAGATTTAAGGTTGCTCTGGAAAACTCATTTG
CACCACTGCTGTGCCTTGGAATGCTAGTTAGAGTAATAAATCTCTGGAACAGATTTGGAATCACACGACCTAGATGGAGT
AGGACAGAGAAATTAACAATTACACAAGCTTAATACACTCCTTAATTGAAGAATCGCAAAACCAGCAAGAAAAGAATGAA
CAAGAATTATTAGAATTAGATAAATGGGCAAGTTTGTGGAATTGGTTTAACATAACAAATTGGCTGTGGTATATAAAATT
ATTCATAATGATAGTAGGAGGCTTAGTAAGTTTAAGAATAGTTTTTGCTGTACTTTCTGTAGTAAATAGAGTTAGGCAAA
GATATTCACCATTATCGTTTCAGACCCACCTCCCAATCCCGAGGGGACCCGACAGGCCCGAAGGAATAGAAGAAGAAGGT
GGAGAGAGAGACAGAGACAGATCCATTCGATTAGTGAACGGATCCTTAGCACTTATCTAGGACGATCTGCGGAGCCTGTG
CCTCTTCAGCTACCACCGCTTGAGAGACTTACTCTTGATTGTAACGAGGATTGTAGAACTTCTAGGACGCAGGAGGTAGG
AAGCCCTCAAATATTGGTAGAATCTCCTACAATATTGGAGTCAGGAGCTAAAGAATAGTGCTGTTAGCTTGCTCAATGCC
ACAGCTATAGCAGTAGCTGAAAGGACAGATAAGGTTATAGAAGTAGTACAAGAAGCTTATAGAGCTATTCGCCACATACC
TAGAAGAATAAGACAGGGCTTGGAAAGGATTTTGCTATAAGATAGGTGGCAAGTGGTCAAAAAGTAGTGTGGTTAGATGG
CCTGCTGTAAGGGAAAGAATGAGACGAGCTGAGCCAGCAGCAGATAAGGTAAGAGCAGCATCTCGAGACCTAGAAAAACA
TGGAGCAATCACAAGTAGCAACACAGCAGCTAACAATGCTGCTTGTGCCTAGCTAGAAGCACAAGAAGAGGAGAAGGTAG
GTTTTCCAGTCACACCTCAGGTACCTTTAAGACCAATGACTTACAAGGCAGCTGTAGATCTTAGCCACTTTTTAAAAGAA
AAGGGGGGACTGGAAGGGCTAATTCACTCCCAACGAAGACAAGATATCCTTGATCTGTGGATCTACCACACACAAGGCTA
CTTCCCTGATTGGCAGAACTACACACCAGGACCAGGGATCAGATATCCACTGACCTTTGGATGGCGCTACAAGCTAGTAC
CAGTTGAGCCAGAGAAGTTAGAAGAAGCCAACAAAGGAGAGAACACCAGCTTGTTACACCCTGTGAGCCTGCATGGAATG
GATGACCCGGAGAGAGAAGTGTTAGAGTGGAGGTTTGACAGCCGCCTAGCATTTCATCACGTGGCCCGAGAGCTGCATCC
GGAGTACTTCAAGAACTGCTGATATCGAGCTTGCTACAAAGGACTTTCCGCTGAGGACTTTCCAGGGAGGCGTGGCCTAG
GCAGGACTGAGGAGTAGCGAGCCCTCAGATCCTGCATATAAGCAGCTGCTTTTTGCCTGTACTAGGTCTCTCTAGTTAGA
CCAGATCTGAGCCTAAGAGCTCTCTGGCTAACTAAGGAACCCACTGCTTAAGCCTCAATAAAGCTTGCCTTAAGTGCTTC
AAGTAGTGTGTGCCCATCTGTTGTGTGACTCTGGTAACTAGAGATCCCTCA

>6-55envLTR


```
--------------------------------------------------------------------------------
--------------------------------------------------------------------------------
--------------------------------------------------------------------------------
--------------------------------------------------------------------------------
--------------------------------------------------------------------------------
--------------------------------------------------------------------------------
--------------------------------------------------------------------------------
--------------------------------------------------------------------------------
--------------------------------------------------------------------------------
--------------------------------------------------------------------------------
--------------------------------------------------------------------------------
--------------------------------------------------------------------------------
--------------------------------------------------------------------------------
--------------------------------------------------------------------------------
--------------------------------------------------------------------------------
--------------------------------------------------------------------------------
--------------------------------------------------------------------------------
--------------------------------------------------------------------------------
--------------------------------------------------------------------------------
--------------------------------------------------------------------------------
--------------------------------------------------------------------------------
--------------------------------------------------------------------------------
--------------------------------------------------------------------------------
--------------------------------------------------------------------------------
--------------------------------------------------------------------------------
--------------------------------------------------------------------------------
--------------------------------------------------------------------------------
--------------------------------------------------------------------------------
--------------------------------------------------------------------------------
--------------------------------------------------------------------------------
--------------------------------------------------------------------------------
--------------------------------------------------------------------------------
--------------------------------------------------------------------------------
--------------------------------------------------------------------------------
--------------------------------------------------------------------------------
--------------------------------------------------------------------------------
--------------------------------------------------------------------------------
--------------------------------------------------------------------------------
--------------------------------------------------------------------------------
--------------------------------------------------------------------------------
--------------------------------------------------------------------------------
--------------------------------------------------------------------------------
--------------------------------------------------------------------------------
--------------------------------------------------------------------------------
--------------------------------------------------------------------------------
--------------------------------------------------------------------------------
--------------------------------------------------------------------------------
--------------------------------------------------------------------------------
--------------------------------------------------------------------------------
--------------------------------------------------------------------------------
--------------------------------------------------------------------------------
--------------------------------------------------------------------------------
--------------------------------------------------------------------------------
--------------------------------------------------------------------------------
--------------------------------------------------------------------------------
--------------------------------------------------------------------------------
--------------------------------------------------------------------------------
--------------------------------------------------------------------------------
--------------------------------------------------------------------------------
--------------------------------------------------------------------------------
--------------------------------------------------------------------------------
--------------------------------------------------------------------------------
--------------------------------------------------------------------------------
--------------------------------------------------------------------------------
--------------------------------------------------------------------------------
--------------------------------------------------------------------------------
--------------------------------------------------------------------------------
--------------------------------------------------------------------------------
--------------------------------------------------------------------------------
--------------------------------------------------------------------------------
--------------------------------------------------------------------------------
--------------------------------------------------------------------------------
--------------------------------------------------------------------------------
--------------------------------------------------------------------------------
--------------------------------------------------------------------------------
--------------------------------------------------------------------------------
--------------------------------------------------------------------------------
--------------------------------------------------------------------------------
--------------------------------------------------------------------------------
--------------------------------------------------------------------------------
--------------------------------------------------------------------------------
--------------------------------------------------------------------------------
--------------------------------------------------------------------------------
--------------------------------------------------------------------------------
--------------------------------------------------------------------------------
--------------------------------------------------------------------------------
--------------------------------------------------------------------------------
--------------------------------------------------------------------------------
--------------------------------------------------------------------------------
--------------------------------------------------------------------------------
--------------------------------------------------------------------------------
--------------------------------------------------------------------------------
----------------------------------------------AATAACAACAATAAGTCCGAGATCTTCAGACCTA
GAGGAGGAGATATGAAGGACAATTGGAGAAGTGAATTATATAAATATAAAGTAGTAAAAATTGAACCATTAGAAGTAGCA
```

CCCACCAAGGCAAAGAGAAGAGTGGTGCAGAGAGAAAAAAGAGCAGTAAGAATAAGAGCTTTGTTCCTTAGGTTCTTAGG
AGCAGCAAGAAGCACTATAGGCGCAGCGTCAATGACGCTGACGGTACAGGCCAGACAATTATTGTCTGGTATAGTGCAGC
AGCAGAACAATTTGCTGAAGGCTATTGAGGCGCAACAGCATCTGTTGCAACTCACAGTCTGGGGCATCAAGCAGCTCCAG
GCAAGAATCCTGGCTATAGAAAGATACCTAAAAGATCAACAGCTCCTAAGGATTTAAAGTTGCTCTAGAAAACTCATTTG
CACCACTGCTGTGCCTTAGAATGCTAGTTAAAGTAATAAATCTCTAGAACAGATTTAGAATCACACGACCTAGATAGAGT
AGGACAGAGAAATTAACAATTACACAAGCTTAATACACTCCTTAATTAAAGAATCGCAAAACCAGCAAGAAAAGAATGAA
CAAGAATTATTAGAATTAGATAAATAGGCAAGTTTATAGAATTAGTTTAACATAACAAATTGGCTGTAGTATATAAAATT
ATTCATAATGATAGTAAGAAGCTTAGTAAGTTTAAGAATAGTTTTTGCTGTACTTTCTGTAGTGAATAAAGTTAGGCAAA
GATATTCACCATTATCGTTTCAGACCCACCTCCCAATCCCGAGAGGACCCGACAGGCCCGAAAGAATAAAAGAAGAAAGT
GAAGAGAAAGACAGAGACAGATCCATTCGATTAGTGAACAGATCCTTAGCACTTATCTAAGACGATCTGCAGAGCCTGTG
CCTCTTCAGCTACCACCGCTTAAAAGACTTACTCTTAATTGTAACGAAAATTGTAGAACTTCTAGGACGCAGAGGGTAGG
AAGCCCTCAAATATTAGTAAAATCTCCTACAATATTAGAGTCAGGAGCTAAAGAATAGTGCTGTTAGCTTGCTCAATGCC
ACAGCTATAGCAGTAGCTGAGAGGACAGATAAAGTTATAGAAGTAGTACAAGAAGCTTATAAAGCTATTCGCCACATACC
TAGAAGAATAAGACAAAGCTTAGAAAAGATTTTGCTATAAGATAGGTGGCAAGTGGTCAAAAAATAGTGTAGTTAGATGG
CCTGCTGTAAAAGAAAGAATAAGACGAGCTGAGCCAGCAGCAGATGAAGTAGGAGCAGCATCTCGAGACCTAGAAAAACA
TAGAGCAATCACAAGTAGCAACACAGCAGCTAACAATGCTGCTTGTGCCTGGCTAGAAGCACAAGAAAAAGAGAAAGTAA
GTTTTCCAGTCACACCTCAGGTACCTTTAAGACCAATGACTTACAAGGCAGCTGTAGATCTTAGCCACTTTTTAAAAGAA
AAGGGGGGACTGGAAGGGCTAATTCACTCCCAACGAAGACAAGATATCCTTGATCTGTGGATCTACCACACACAAGGCTA
CTTCCCTGATTGGCAGAACTACACACCAGGACCAGGGATCAGATATCCACTGACCTTTGGATGGCGCTACAAGCTAGTAC
CAGTTGAGCCAGAGAAGTTAGAAGAAGCCAACAAAGGAGAGAACACCAGCTTGTTACACCCTGTGAGCCTGCATGGAATG
GATGACCCGGAGAGAGAAGTGTTAGAGTGGAGGTTTGACAGCCGCCTAGCATTTCATCACGTGGCCCGAGAGCTGCATCC
GGAGTACTTCAAGAACTGCTGATATCGAGCTTGCTACAAAGGACTTTCCGCTGGGGACTTTCCAGGGAGGCGTGGCCTGG
GCGGGACTAAGGAGTGGCGAGCCCTCAGATCCTGCATATAAGCAGCTGCTTTTTGCCTGTACTAGGTCTCTCTGGTTAGA
CCAGATCTGAGCCTAGGAGCTCTCTGGCTAACTAAGGAACCCACTGCTTAAGCCTCAATAAAGCTTGCCTTGAGTGCTTC
AAGTAGTGTGTGCCCATCTGTTGTGTGACTCTGGTAACTAGAGATCCCTCA

>6-94envLTR

------------------------GCTATTAACAAGAGATGGTAGTAATAACAACAATAGGTCCGAGATCTTCAGACCTG
GAGGAGGAGATATGAAGGACAATTAGAGAAGTGAATTATATAAATATAAAGTAGTAAAAATTGAACCATTAAGAGTAGCA
CCCACCAAGGCAAAGAGAAGAGTGGTGCAGAGAGAAAAAAGAGCAGTAAGAATAGGAGCTTTGTTCCTTAGGTTCTTAAG
AGCAGCAGGAAGCACTATAGGCGCAGCGTCAATGACGCTGACGGTACAGGCCAGACAATTATTGTCTGGTATAGTGCAGC
AGCAGAACAATTTGCTGAGGGCTATTGAGGCGCAACAGCATCTGTTGCAACTCACAGTCTGGGGCATCAAGCAGCTCCAG
GCAAGAATCCTGGCTGTAGAAAGATACCTAAAGGATCAACAGCTCCTAAGGATTTGAGGTTGCTCTAGAAAACTCATTTG
CACCACTGCTGTGCCTTAGAATGCTAGTTAGAGTAATAAATCTCTGGAACAGATTTAGAATCACACGACCTAGATAGAGT
AGGACAGAAAAATTAACAATTACACAAGCTTAATACACTCCTTAATTGAAGAATCGCAAAACCAGCAAGAAAAGAATGAA
CAAGAATTATTAGAATTAGATAAATAGGCAAGTTTGTAGAATTAGTTTAACATAACAAATTAGCTGTAGTATATAAAATT
ATTCATAATGATAGTAGGAAGCTTAGTAGGTTTAAGAATAGTTTTTGCTGTACTTTCTGTAGTGAATAAAGTTAGGCAAA
GATATTCACCATTATCGTTTCAGACCCACCTCCCAATCCCGAGGGGACCCGACAGGCCCGAAAGAATAGAAGAAGAAGGT
GGAGAGAGAGACAGAGACAGATCCATTCGATTAATGAACGGATCCTTAGCACTTATCTAGGACGATCTGCAGAGCCTGTG
CCTCTTCAGCTACCACCGCTTGAGAGACTTACTCTTGATTGTAACGAAGATTGTAGAACTTCTAAGACGCAGGAAGTAAG
AAGCCCTCAAATATTAGTAGAATCTCCTACAATATTGGAGTCAGGAGCTAAAGAATAGTGCTGTTAGCTTGCTCAATGCC
ACAGCTATAGCAGTAGCTGAGAGGACAGATAAGGTTATAGAAGTAGTACAAGAAGCTTATAAAGCTATTCGCCACATACC
TAGAAGAATAAGACAGGGCTTAGAAAAGATTTTGCTATAAGATAGGTGGCAAGTGGTCAAAAAGTAGTGTGGTTAGATGG
CCTGCTGTAAAAGAAAGAATGAGACGAGCTGAGCCAGCAGCAGATAGAGTAGGAGCAGCATCTCGAGACCTAGAAAAACA
TAGAGCAATCACAAGTAGCAACACAGCAGCTAACAATGCTGCTTGTGCCTGGCTAGAAGCACAAGAGGAGGAGAAGGTAG
GTTTTCCAGTCACACCTCAAGTACCTTTAAGACCAATGACTTACAAGGCAGCTGTAGATCTTAGCCACTTTTTAAAAGAA
AAGGGGGGACTGGAAGGGCTAATTCACTCCCAACGAAGACAAGATATCCTTGATCTGTGGATCTACCACACACAAGGCTA
CTTCCCTGATTGGCAGAACTACACACCAGGACCAGGGATCAGATATCCACTGACCTTTGGATGGCGCTACAAGCTAGTAC
CAGTTGAGCCAGAGAAGTTAGAAGAAGCCAACAAAGGAGAGAACACCAGCTTGTTACACCCTGTGAGCCTGCATGGAATG
GATGACCCGGAGAGAGAAGTGTTAGAGTGGAGGTTTGACAGCCGCCTAGCATTTCATCACGTGGCCCGAGAGCTGCATCC
GGAGTACTTCAAGAACTGCTGATATCGAGCTTGCTACAAAGGACTTTCCGCTGAGGACTTTCCAGGGAGGCGTAGCCTAG
GCGGGACTGAGGAGTGGCAAGCCCTCAGATCCTGCATATAAGCAGCTGCTTTTTGCCTATACTAAGTCTCTCTAGTTAGA
CCAGATCTGAGCCTAGGAGCTCTCTGGCTAACTAAGGAACCCACTGCTTAAGCCTCAATAAAGCTTGCCTTGAGTGCTTC
AAGTAGTGTGTGCCCATCTGTTGTGTGACTCTAGTAACTAGAGATCCCTCA
>6-97envLTR


```
--------------------------------------------------------------------------------
--------------------------------------------------------------------------------
--------------------------------------------------------------------------------
--------------------------------------------------------------------------------
--------------------------------------------------------------------------------
--------------------------------------------------------------------------------
--------------------------------------------------------------------------------
--------------------------------------------------------------------------------
--------------------------------------------------------------------------------
--------------------------------------------------------------------------------
--------------------------------------------------------------------------------
--------------------------------------------------------------------------------
--------------------------------------------------------------------------------
--------------------------------------------------------------------------------
--------------------------------------------------------------------------------
--------------------------------------------------------------------------------
--------------------------------------------------------------------------------
--------------------------------------------------------------------------------
--------------------------------------------------------------------------------
--------------------------------------------------------------------------------
--------------------------------------------------------------------------------
--------------------------------------------------------------------------------
--------------------------------------------------------------------------------
--------------------------------------------------------------------------------
--------------------------------------------------------------------------------
--------------------------------------------------------------------------------
--------------------------------------------------------------------------------
--------------------------------------------------------------------------------
--------------------------------------------------------------------------------
--------------------------------------------------------------------------------
--------------------------------------------------------------------------------
--------------------------------------------------------------------------------
--------------------------------------------------------------------------------
--------------------------------------------------------------------------------
--------------------------------------------------------------------------------
--------------------------------------------------------------------------------
--------------------------------------------------------------------------------
--------------------------------------------------------------------------------
--------------------------------------------------------------------------------
--------------------------------------------------------------------------------
--------------------------------------------------------------------------------
--------------------------------------------------------------------------------
--------------------------------------------------------------------------------
--------------------------------------------------------------------------------
--------------------------------------------------------------------------------
--------------------------------------------------------------------------------
--------------------------------------------------------------------------------
--------------------------------------------------------------------------------
--------------------------------------------------------------------------------
--------------------------------------------------------------------------------
--------------------------------------------------------------------------------
--------------------------------------------------------------------------------
--------------------------------------------------------------------------------
--------------------------------------------------------------------------------
-----------------------TGCTATTAACAAGAGATGGTAGTAATAACAACAATAGGTCCAAGATCTTCAGACCTA
GAGGAAGAGATATGAAGGACAATTAGAGAAGTGAATTATATAAATATAAAGTAGTAAAATTGAACCATTAAGAGTAGCA
CCCACCAAGGCAAAGAGAAGAGTAGTGCAGAGAGAAAAAAGAGCAGTAAGAATAAGAGCTTTGTTCCTTAGGTTCTTAAG
AGCAGCAAGAAGCACTATAGGCGCAGCGTCAATGACGCTGACGGTACAGGCCAGACAATTATTGTCTAGTATAGTGCAGC
AGCAGAACAATTTGCTGAGGGCTATTAAGGCGCAACAGCATCTGTTGCAACTCACAGTCTGAGGCATCAAGCAGCTCCAG
GCAAGAATCCTGGCTGTGGAAAGATACCTAAAGGATCAACAGCTCCTAAAGATTTAAAGTTGCTCTAGAAAACTCATTTG
CACCACTGCTGTGCCTTAGAATGCTAGTTGAAGTAATAAATCTCTAGAACAGATTTAGAATCACACGACCTGGATAGAGT
AGGACAGAGAAATTAACAATTACACAAGCTTAATACACTCCTTAATTGAAGAATCGCAAAACCAGCAAGAAAAGAATGAA
CAAGAATTATTAGAATTAGATAAATAGGCAAGTTTGTGGAATTGGTTTAACATAACAAATTGGCTGTAGTATATAAAATT
ATTCATAATGATAGTAGGAGGCTTGGTAGGTTTAAGAATAGTTTTTGCTGTACTTTCTGTAGTGAATAGAGTTAGGCAGG
GATATTCACCATTATCGTTTCAGACCCACCTCCCAATCCCGAGGGGACCCGACAGGCCCGAAGGAATAGAAGAAGAAGGT
GGAGAGAGAGACAGAGACAGATCCATTCGATTAGTGAACGGATCCTTAGCACTTATCTGGGACGATCTGCGGAGCCTGTG
CCTCTTCAGCTACCACCGCTTGAGAGACTTACTCTTGATTGTAACGAGGATTGTAGAACTTCTAGGACGCAGGGGGTAGG
AAGCCCTCAAATATTGGTGGAATCTCCTACAATATTGGAGTCAGGAGCTAAAGAATAGTGCTGTTAGCTTGCTCAATGCC
ACAGCTATAGCAGTAGCTGAGAGGACAGATAGGGTTATAGAAGTAGTACAAGAAGCTTATAGAGCTATTCGCCACATACC
TAGAAGAATAAGACAGGGCTTGGAAAGGATTTTGCTATAAGATAGGTGGCAAGTGGTCAAAAAGTAGTGTGGTTAGATGG
CCTGCTGTAAGGGAAAGAATGAGACGAGCTGAGCCAGCAGCAGATGAGGTAGGAGCAGCATCTCGAGACCTAGAAAAACA
TGGAGCAATCACAAGTAGCAACACAGCAGCTAACAATGCTGCTTGTGCCTGGCTAGAAGCACAAGAAGAAGAGAAGGTAG
GTTTTCCAGTCACACCTCAGGTACCTTTAAGACCAATGACTTACAAGGCAGCTGTAGATCTTAGCCACTTTTTAAAAGAA
AAGGGGGGACTAGAAGGGCTAATTCACTCCCAACGAAGACAAGATATCCTTGATCTGTAGATCTACCACACACAAGGCTA
CTTCCCTGATTGGCAGAACTACACACCAGGACCAGGGATCAGATATCCACTGACCTTTGGATGGCGCTACAAGCTAGTAC
CAGTTGAGCCAGAGAAGTTAGAAGAAGCCAACAAAAGAGAGAACACCAGCTTGTTACACCCTGTGAGCCTGCATAGAATA
GATGACCCAGAGAGAGAAGTGTTAGAGTGGAGGTTTGACAGCCGCCTAGCATTTCATCACGTGGCCCGAGAGCTGCATCC
GGAGTACTTCAAGAACTGCTGATATCGAGCTTGCTACAAAGGACTTTCCGCTGAGGACTTTCCAGGGAGGCGTGGCCTAG
GCAAGACTAAGGAGTGGCGAGCCCTCAGATCCTGCATATAAGCAGCTGCTTTTTGCCTGTACTAGGTCTCTCTGGTTAGA
CCAGATCTGAGCCTAGGAGCTCTCTGGCTAACTAAGGAACCCACTGCTTAAGCCTCAATAAAGCTTGCCTTGAGTGCTTC
AAGTAGTGTGTGCCCATCTGTTGTGTGACTCTAGTAACTAGAGATCCCTCA
```

2.Sequence alignments from the in vitro titration experiment: near-full length proviruses

```
>consensus
--------------------------------------------------------------------------------
--------------------------------------------------------------------------------
--------------------------------------------------------------------------------
--------------------------------------------------------------------------------
--------------------------------------------------------------------------------
--------------------------------------------------------------------------------
--------------------------------------------------------------------------------
```

```
--------------------------------------------------------------------------------
--------------------------------------------------------------------------------
--------------------------------------------------------------------------------
--------------------------------------------------------------------------------
--------------------------------------------------------------------------------
--------------------------------------------------------------------------------
--------------------------------------------------------------------------------
---------------------------------------------------------------ACCCTATAGTGCAGAAC
ATCCAGGGGCAA-ATGGTACATCAGGCCATATCACCTAGAACTTTAAATGCATGGGTAAAAGTAGTAGAAGAGAAGGCTT
TCAGCCCAGAAGTGATACCCATGTTTTCAGCATTATCAGAAGGAGCCACCCCACAAGATTTAAACACCATGCTAAACACA
GTGGGGGGACATCAAGCAGCCATGCAAATGTTAAAAGAGACCATCAATGAGGAAGCTGCAGAATGGGATAGAGTGCATCC
AGTGCATGCAGGGCCTATTGCACCAGGCCAGATGAGAGAACCAAGGGGAAGTGACATAGCAGGAACTACTAGTACCCTTC
AGGAACAAATAGGATGGATGACACATAATCCACCTATCCCAGTAGGAGAAATCTATAAAAGATGGATAATCCTGGGATTA
AATAAAATAGTAAGAATGTATAGCCCTACCAGCATTCTGGACATAAGACAAGGACCAAAGGAACCCTTTAGAGACTATGT
AGACCGATTCTATAAAACTCTAAGAGCCGAGCAAGCTTCACAAGAGGTAAAAAATTGGATGACAGAAACCTTGTTGGTCC
AAAATGCGAACCCAGATTGTAAGACTATTTTAAAAGCATTGGGACCAGGAGCGACACTAGAAGAAATGATGACAGCATGT
CAGGGAGTGGGGGGACCCGGCCATAAAGCAAGAGTTTTGGCTGAAGCAATGAGCCAAGTAACAAATCCAGCTACCATAAT
GATACAGAAAGGCAATTTTAGGAACCAAAGAAAGACTGTTAAGTGTTTCAATTGTGGCAAAGAAGGGCACATAGCCAAAA
ATTGCAGGGCCCCTAGGAAAAAGGGCTGTTGGAAATGTGGAAAGGAAGGACACCAAATGAAAGATTGTACTGAGAGACAG
GCTAATTTTTTAGGGAAGATCTGGCCTTCCCACAAGGGAAGGCCAGGGAATTTTCTTCAGAGCAGACCAGAGCCAACAGC
CCCACCAGAAGAGAGCTTCAGGTTTGGGGAAGAGACAACAACTCCCTCTCAGAAGCAGGAGCCGATAGACAAGGAACTGT
ATCCTTTAGCTTCCCTCAGATCACTCTTTGGCAGCGACCCCTCGTCACAATAAAGATAGGGGGGCAATTAAAGGAAGCTC
TATTAGATACAGGAGCAGATGATACAGTATTAGAAGAAATGAATTTGCCAGGAAGATGGAAACCAAAAATGATAGGGGGA
ATTGGAGGTTTTATCAAAGTAAGACAGTATGATCAGATACTCATAGAAATCTGCGGACATAAAGCTATAGGTACAGTATT
AGTAGGACCTACACCTGTCAACATAATTGGAAGAAATCTGTTGACTCAGATTGGCTGCACTTTAAATTTTCCCATTAGTC
CTATTGAGACTGTACCAGTAAAATTAAAGCCAGGAATGGATGGCCCAAAAGTTAAACAATGGCCATTGACAGAAGAAAAA
ATAAAAGCATTAGTAGAAATTTGTACAGAAATGGAAAAGGAAGGAAAAATTTCAAAAATTGGGCCTGAAAATCCATACAA
TACTCCAGTATTTGCCATAAAGAAAAAAGACAGTACTAAATGGAGAAAATTAGTAGATTTCAGAGAACTTAATAAGAGAA
CTCAAGATTTCTGGGAAGTTCAATTAGGAATACCACATCCTGCAGGGTTAAAACAGAAAAAATCAGTAACAGTACTGGAT
GTGGGCGATGCATATTTTTCAGTTCCCTTAGATAAAGACTTCAGGAAGTATACTGCATTTACCATACCTAGTATAAACAA
TGAGACACCAGGGATTAGATATCAGTACAATGTGCTTCCACAGGGATGGAAAGGATCACCAGCAATATTCCAGTGTAGCA
TGACAAAAATCTTAGAGCCTTTTAGAAAACAAAATCCAGACATAGTCATCTATCAATACATGGATGATTTGTATGTAGGA
TCTGACTTAGAAATAGGGCAGCATAGAACAAAAATAGAGGAACTGAGACAACATCTGTTGAGGTGGGGATTTACCACACC
AGACAAAAAACATCAGAAAGAACCTCCATTCCTTTGGATGGGTTATGAACTCCATCCTGATAAATGGACAGTACAGCCTA
TAGTGCTGCCAGAAAAGGACAGCTGGACTGTCAATGACATACAGAAATTAGTGGGAAAATTGAATTGGGCAAGTCAGATT
TATGCAGGGATTAAAGTAAGGCAATTATGTAAACTTCTTAGGGGAACCAAAGCACTAACAGAAGTAGTACCACTAACAGA
AGAAGCAGAGCTAGAACTGGCAGAAAACAGGGAGATTCTAAAAGAACCGGTACATGGAGTGTATTATGACCCATCAAAAG
ACTTAATAGCAGAAATACAGAAGCAGGGGCAAGGCCAATGGACATATCAAATTTATCAAGAGCCATTTAAAAATCTGAAA
ACAGGAAAGTATGCAAGAATGAAGGGTGCCCACACTAATGATGTGAAACAATTAACAGAGGCAGTACAAAAAATAGCCAC
AGAAAGCATAGTAATATGGGGAAAGACTCCTAAATTT-AAATTACCCATACAAAAGGAAACATGGGAAGCATGGTGGACA
GAGTATTGGCAAGCCACCTGGATTCCTGAGTGGGAGTTTGTCAATACCCCTCCCTTAGTGAAGTTATGGTACCAGTTAGA
GAAAGAACCCATAATAGGAGCAGAAACTTTCTATGTAGATGGGGCAGCCAATAGGGAAACTAAATTAGGAAAAGCAGGAT
ATGTAACTGACAGAGGAAGACAAAAAGTTGTCCCCCTAACGGACACAACAAATCAGAAGACTGAGTTACAAGCAATTCAT
CTAGCTTTGCAGGATTCGGGATTAGAAGTAAACATAGTGACAGACTCACAATATGCATTGGGAATCATTCAAGCACAACC
AGATAAGAGTGAATCAGAGTTAGTCAGTCAAATAATAGAGCAGTTAATAAAAAAGGAAAAAGTCTACCTGGCATGGGTAC
CAGCACACAAAGGAATTGGAGGAAATGAACAAGTAGATAAATTGGTCAGTGCTGGAATCAGGAAAGTACTATTTTTAGAT
GGAATAGATAAGGCCCAAGAAGAACATGAGAAATATCACAGTAATTGGAGAGCAATGGCTAGTGATTTTAACCTACCACC
TGTAGTAGCAAAAGAAATAGTAGCCAGCTGTGATAAATGTCAGCTAAAAGGGGAAGCCATGCATGGACAAGTAGACTGTA
GCCCAGGAATATGGCAGCTAGATTGTACACATTTAGAAGGAAAAGTTATCTTGGTAGCAGTTCATGTAGCCAGTGGATAT
ATAGAAGCAGAAGTAATTCCAGCAGAGACAGGGCAAGAAACAGCATACTTCCTCTTAAAATTAGCAGGAAGATGGCCAGT
AAAAACAGTACATACAGACAATGGCAGCAATTTCACCAGTACTACAGTTAAGGCCGCCTGTTGGTGGGCGGGGATCAAGC
AGGAATTTGGCATTCCCTACAATCCCCAAAGTCAAGGAGTAATAGAATCTATGAATAAAGAATTAAAGAAAATTATAGGA
CAGGTAAGAGATCAGGCTGAACATCTTAAGACAGCAGTACAAATGGCAGTATTCATCCACAATTTTAAAAGAAAAGGGGG
GATTGGGGGGTACAGTGCAGGGGAAAGAATAGTAGACATAATAGCAACAGACATACAAACTAAAGAATTACAAAAACAAA
TTACAAAAATTCAAAATTTTCGGGTTTATTACAGGGACAGCAGAGATCCAGTTTGGAAAGGACCAGCAAAGCTCCTCTGG
AAAGGTGAAGGGGCAGTAGTAATACAAGATAATAGTGACATAAAAGTAGTGCCAAGAAGAAAAGCAAAGATCATCAGGGA
TTATGGAAAACAGATGGCAGGTGATGATTGTGTGGCAAGTAGACAGGATGAGGATTAACACATGGAAAAGATTAGTATAA
TAGCATATGTATATTTCAAGGAAAGCTAAGGACTGGTTTTATAGACATCACTATGAAAGTACTAATCCAAAAATAAGTTC
AGAAGTACACATCCCACTAGGGGATGCTAAATTAGTAATAACAACATATTGGGGTCTGCATACAGGAGAAAGAGACTGGC
ATTTGGGTCAGGGAGTCTCCATAGAATGGAGGAAAAAGAGATATAGCACACAAGTAGACCCTGACCTAGCAGACCAACTA
ATTCATCTGCACTATTTTGATTGTTTTTCAGAATCTGCTATAAGAAATACCATATTAGGACGTATAGTTAGTCCTAGGTG
TGAATATCAAGCAGGACATAACAAGGTAGGATCTCTACAGTACTTGGCACTAGCAGCATTAATAAAACCAAAACAGATAA
AGCCACCTTTGCCTAGTGTTAGGAAACTGACAGAGGACAGATGGAACAAGCCCCAGAAGACCAAGGGCCACAGAGGGAGC
CATACAATGAATGGACACTAGAGCTTTTAGAGGAACTTAAGAGTGAAGCTGTTAGACATTTTCCTAGGATATGGCTCCAT
AACTTAGGACAACATATCTATGAAACTTACGGGGATACTTGGGCAGGAGTGGAAGCCATAATAAGAATTCTGCAACAACT
GCTGTTTATCCATTTCAGAATTGGGTGTCGACATAGCAGAATAGGCGTTACTCGACAGAGGAGAGCAAGAAATGGAGCCA
GTAGATCCTAGACTAGAGCCCTGGAAGCATCCAGGAAGTCAGCCTAAAACTGCTTGTACCAATTGCTATTGTAAAAAGTG
TTGCTTTCATTGCCAAGTTTGTTTCATAACAAAAGCCTTAGGCATCTCCTATGGCAGGAAGAAGCGGAGACAGCGACGAA
GACCTCCTCAAGGCAGTCAGACTCATCAAGTTTCTCTATCAAAGCAGTAAGTAATACATGTAATGCAACCTATACAAATA
GCAATAGTAGCATTAGTAGTAGCAATAATAATAGCAATAGTTGTGTGGTCCATAGTAATCATAGAATATAGGAAAATATT
AAGACAAAGAAAAATAGACAGGTTAATTGATAGACTAATAGAAAGAGCAGAAGACAGTGGCAATGAGAGTGAAGGAGAAA
TATCAGCACTTGTGGAGATGGGGGTGGAGATGGGGCACCATGCTCCTTGGGATGTTGATGATCTGTAGTGCTACAGAAAA
ATTGTGGGTCACAGTCTATTATGGGGTACCTGTGTGGAAGGAAGCAACCACCACTCTATTTTGTGCATCAGATGCTAAAG
CATATGATACAGAGGTACATAATGTTTGGGCCACACATGCCTGTGTACCCACAGACCCCAACCCACAAGAAGTAGTATTG
GTAAATGTGACAGAAAATTTTAACATGTGGAAAAATGACATGGTAGAACAGATGCATGAGGATATAATCAGTTTATGGGA
TCAAAGCCTAAAGCCATGTGTAAAATTAACCCCACTCTGTGTTAGTTTAAAGTGCACTGATTTGAAGAATGATACTAATA
CCAATAGTAGTAGCGGGAGAATGATAATGGAGAAAGGAGAGATAAAAAACTGCTCTTTCAATATCAGCACAAGCATAAGA
GGTAAGGTGCAGAAAGAATATGCATTTTTTTATAAACTTGATATAATACCAATAGATAATGATACTACCAGCTATACGTT
GACAAGTTGTAACACCTCAGTCATTACACAGGCCTGTCCAAAGGTATCCTTTGAGCCAATTCCCATACATTATTGTGCCC
CGGCTGGTTTTGCGATTCTAAAATGTAATAATAAGACGTTCAATGGAACAGGACCATGTACAAATGTCAGCACAGTACAA
TGTACACATGGAATTAGGCCAGTAGTATCAACTCAACTGCTGTTAAATGGCAGTCTAGCAGAAGAAGAGGTAGTAATTAG
ATCTGTCAATTTCACGGACAATGCTAAAACCATAATAGTACAGCTGAACACATCTGTAGAAATTAATTGTACAAGACCCA
ACAACAATACAAGAAAAAAAATCCGTATCCAGAGGGGACCAGGGAGAGCATTTGTTACAATAGGAAAAATAGGAAATATG
AGACAAGCACATTGTAACATTAGTAGAGCAAAATGGAATGCCACTTTAAAACAGATAGCTAGCAAATTAAGAGAACAATT
TGGAAATAATAAAACAATAATCTTTAAGCAATCCTCAGGAGGGGACCCAGAAATTGTAACGCACAGTTTTAATTGTGGAG
GGGAATTTTTCTACTGTAATTCAACACAACTGTTTAATAGTACTTGGTTTAATAGTACTTGGAGTACTGAAGGGTCAAAT
AACACTGAAGGAAGTGACACAATCACACTCCCATGCAGAATAAAACAATTTATAAACATGTGGCAGGAAGTAGGAAAAGC
AATGTATGCCCCTCCCATCAGCGGACAAATTAGATGTTCATCAAATATTACAGGGCTGCTATTAACAAGAGATGGTGGTA
ATAACAACAATGGGTCCGAGATCTTCAGACCTGGAGGAGGAGATATGAGGGACAATTGGAGAAGTGAATTATATAAATAT
AAAGTAGTAAAAATTGAACCATTAGGAGTAGCACCCACCAAGGCAAAGAGAAGAGTGGTGCAGAGAGAAAAAAGAGCAGT
GGGAATAGGAGCTTTGTTCCTTGGGTTCTTGGGAGCAGCAGGAAGCACTATGGGCGCAGCGTCAATGACGCTGACGGTAC
AGGCCAGACAATTATTGTCTGGTATAGTGCAGCAGCAGAACAATTTGCTGAGGGCTATTGAGGCGCAACAGCATCTGTTG
CAACTCACAGTCTGGGGCATCAAGCAGCTCCAGGCAAGAATCCTGGCTGTGGAAAGATACCTAAAGGATCAACAGCTCCT
GGGGATTTGGGGTTGCTCTGGAAAACTCATTTGCACCACTGCTGTGCCTTGGAATGCTAGTTGGAGTAATAAATCTCTGG
```

```
AACAGATTTGGAATCACACGACCTGGATGGAGTGGGACAGAGAAATTAACAATTACACAAGCTTAATACACTCCTTAATT
GAAGAATCGCAAAACCAGCAAGAAAAGAATGAACAAGAATTATTGGAATTAGATAAATGGGCAAGTTTGTGGAATTGGTT
TAACATAACAAATTGGCTGTGGTATATAAAATTATTCATAATGATAGTAGGAGGCTTGGTAGGTTTAAGAATAGTTTTTG
CTGTACTTTCTGTAGTGAATAGAGTTAGGCAGGGATATTCACCATTATCGTTTCAGACCCACCTCCCAATCCCGAGGGGA
CCCGACAGGCCCGAAGGAATAGAAGAAGAAGGTGGAGAGAGAGACAGAGACAGATCCATTCGATTAGTGAACGGATCCTT
AGCACTTATCTGGGACGATCTGCGGAGCCTGTGCCTCTTCAGCTACCACCGCTTGAGAGACTTACTCTTGATTGTAACGA
GGATTGTGGAACTTCTGGGACGCAGGGGGTGGGAAGCCCTCAAATATTGGTGGAATCTCCTACAATATTGGAGTCAGGAG
CTAAAGAATAGTGCTGTTAGCTTGCTCAATGCCACAGCTATAGCAGTAGCTGAGGGGACAGATAGGGTTATAGAAGTAGT
ACAAGAAGCTTATAGAGCTATTCGCCACATACCTAGAAGAATAAGACAGGGCTTGGAAAGGATTTTGCTATAAGATGGGT
GGCAAGTGGTCAAAAAGTAGTGTGGTTGGATGGCCTGCTGTAAGGGAAAGAATGAGACGAGCTGAGCCAGCAGCAGATGG
GGTGGGAGCAGCATCTCGAGACCTAGAAAAACATGGAGCAATCACAAGTAGCAACACAGCAGCTAACAATGCTGCTTGTG
CCTGGCTAGAAGCACAAGAGGAGGAGAAGGTGGGTTTTCCAGTCACACCTCAGGTACCTTTAAGACCAATGACTTACAAG
GCAGCTGTAGATCTTAGCCACTTTTTAAAAGAAAAGGGGGGACTGGAAGGGCTAATTCACTCCCAACGAAGACAAGATAT
CCTTGATCTGTGGATCTACCACACACAAGGCTACTTCCCTGATTGGCAGAACTACACACCAGGACCAGGGATCAGATATC
CACTGACCTTTGGATGGCGCTACAAGCTAGTACCAGTTGAGCCAGAGAAGTTAGAAGAAGCCAACAAAGGAGAGAACACC
AGCTTGTTACACCCTGTGAGCCTGCATGGAATGGATGACCCGGAGAGAGAAGTGTTAGAGTGGAGGTTTGACAGCCGCCT
AGCATTTCATCACGTGGCCCGAGAGCTGCATCCGGAGTACTTCAAGAACTGCTGATATCGAGCTTGCTACAAGGGACTTT
CCGCTGGGGACTTTCCAGGGAGGCGTGGCCTGGGCGGGACTGGGGAGTGGCGAGCCCTCAGATCCTGCATATAAGCAGCT
GCTTTTTGCCTGTACTGGGTCTCTCTGGTTAGACCAGATCTGAGCCTGGGAGCTCTCTGGCTAACTAGGGAACCCACTGC
TTAAGCCTCAATAAAGCTTGCCTTGAGTGCTTCAAGTAGTGTGTGCCCATCTGTTGTGTGACTCTGGTAACTAGAGATCC
CTCA
>2-29LONG
--------------------------------------------------------------------------------
--------------------------------------------------------------------------------
--------------------------------------------------------------------------------
--------------------------------------------------------------------------------
--------------------------------------------------------------------------------
--------------------------------------------------------------------------------
--------------------------------------------------------------------------------
--------------------------------------------------------------------------------
--------------------------------------------------------------------------------
--------------------------------------------------------------------------------
--------------------------------------------------------------------------------
--------------------------------------------------------------------------------
--------------------------------------------------------------------------------
--------------------------------------------------------------------------------
---------------------------------------------------------------ACCCTATAGTGCAGAAC
ATCCAGGGGCAA-ATGGTACATCAGGCCATATCACCTAGAACTTTAAATGCATGGGTAAAAGTAGTAGAAGAGAAGGCTT
TCAGCCCAGAAGTGATACCCATGTTTTCAGCATTATCAGAAGGAGCCACCCCACAAGATTTAAACACCATGCTAAACACA
GTGGGGGGACATCAAGCAGCCATGCAAATGTTAAAAGAGACCATCAATGAGGAAGCTGCAGAATAGGATAGAGTGCATCC
AGTGCATGCAGGGCCTATTGCACCAGGCCAGATGAGAGAACCAAGGGGAAGTGACATAGCAGGAACTACTAGTACCCTTC
AGGAACAAATAGGATGGATGACACATAATCCACCTATCCCAGTAGGAGAAATCTATAAAAGATGGATAATCCTGGGATTA
AATAAAATAGTAAGAATGTATAGCCCTACCAGCATTCTGGACATAAGACAAGGACCAAAGGAACCCTTTAGAGACTATGT
AGACCGATTCTATAAAACTCTAAGAGCCGAGCAAGCTTCACAAGAGGTAAAAAATTGGATGACAGAAACCTTGTTGGTCC
AAAATGCGAACCCAGATTGTAAGACTATTTTAAAAGCATTGGGACCAGGAGCGACACTAGAAGAAATGATGACAGCATGT
CAGGGAGTGGGGGGACCCGGCCATAAAGCAAGAGTTTTGGCTGAAGCAATGAGCCAAGTAACAAATCCAGCTACCATAAT
GATACAGAAAGGCAATTTTAAGAACCAAAGAAAGACTGTTAAGTGTTTCAATTGTGGCAAAGAAGGGCACATAGCCAAAA
ATTGCAGGGCCCCTAGGAAAAAGGGCTGTTGGAAATGTAGAAAAGAAGGACACCAAATGAAAGATTGTACTGAGAGACAG
GCTAATTTTTTAGGAAGATCTGGCCTTCCCACAAGGGAAGGCCAAGGAATTTTCTTCAGAGCAGACCAGAGCCAACAGC
CCCACCAGAAGAGAGCTTCAGGTTTGGGGAAGAGACAACAACTCCCTCTCAGAAGCAGGAGCCGATAGACAAGGAACTGT
ATCCTTTAGCTTCCCTCAGATCACTCTTTGGCAGCGACCCCTCGTCACAATAAAGATAGGGGGGCAATTAAAGGAAGCTC
TATTAGATACAGGAGCAGATGATACAGTATTAGAAGAAATGAATTTGCCAGGAAGATGGAAACCAAAAATGATAGGGGGA
ATTGGAGGTTTTATCAAAGTAAGACAGTATGATCAGATACTCATAGAAATCTGCGGACATAAAGCTATAGGTACAGTATT
AGTAGGACCTACACCTGTCAACATAATTGGAAGAAATCTGTTGACTCAGATTGGCTGCACTTTAAATTTTCCCATTAGTC
CTATTGAGACTGTACCAGTAAAATTAAAGCCAGGAATGGATGGCCCAAAAGTTAAACAATGGCCATTGACAGAAGAAAAA
ATAAAAGCATTAGTAGAAATTTGTACAGAAATGGAAAAGGAAGGAAAAATTTCAAAAATTGGGCCTGAAAATCCATACAA
TACTCCAGTATTTGCCATAAAGAAAAAAGACAGTACTAAATGGAGAAAATTAGTAGATTTCAGAGAACTTAATAAGAGAA
CTCAAGATTTCTGGGAAGTTCAATTAGGAATACCACATCCTGCAGGGTTAAAACAGAAAAAATCAGTAACAGTACTAGAT
GTAGGCGATGCATATTTTTCAGTTCCCTTAGATAAAGACTTCAGGAAGTATACTGCATTTACCATACCTAGTATAAACAA
TGAGACACCAGGGATTAGATATCAGTACAATGTGCTTCCACAGGGATAGAAAGGATCACCAGCAATATTCCAGTGTAGCA
TGACAAAAATCTTAGAGCCTTTTAGAAAACAAAATCCAGACATAGTCATCTATCAATACATGGATGATTTGTATGTAGGA
TCTGACTTAGAAATAGGGCAGCATAGAACAAAAATAGAGGAACTGAGACAACATCTGTTGAGGTGAGGATTTACCACACC
AGACAAAAAACATCAGAAAGAACCTCCATTCCTTTGGATAGGTTATGAACTCCATCCTGATAAATGGACAGTACAGCCTA
TAGTGCTGCCAGAAAAGGACAGCTGGACTGTCAATGACATACAGAAATTAGTAGGAAAATTGAATTGGGCAAGTCAGATT
TATGCAGGGATTAAAGTAAGGCAATTATGTAAACTTCTTAGGGGAACCAAAGCACTAACAGAAGTAGTACCACTAACAGA
AGAAGCAGAGCTAGAACTGGCAGAAAACAGGGAGATTCTAAAAGAACCGGTACATGGAGTGTATTATGACCCATCAAAAG
ACTTAATAGCAGAAATACAGAAGCAGGGGCAAGGCCAATGGACATATCAAATTTATCAAGAGCCATTTAAAAATCTGAAA
ACAGGAAAGTATGCAAGAATGAAAGGTGCCCACACTAATGATGTGAAACAATTAACAGAGGCAGTACAAAAAATAGCCAC
AGAAAGCATAGTAATATGGGGAAAGACTCCTAAATTT-AAATTACCCATACAAAAGGAAACATAGGAAGCATGGTGGACA
GAGTATTGGCAAGCCACCTGGATTCCTGAGTGGGAGTTTGTCAATACCCCTCCCTTAGTGAAGTTATAGTACCAGTTAGA
GAAAGAACCCATAATAGGAGCAGAAACTTTCTATGTAGATGGGGCAGCCAATAAGGAAACTAAATTAGGAAAAGCAGGAT
ATGTAACTGACAGAGGAAGACAAAAAGTTGTCCCCCTAACGGACACAACAAATCAGAAGACTGAGTTACAAGCAATTCAT
CTAGCTTTGCAGGATTCAGGATTAGAAGTAAACATAGTGACAGACTCACAATATGCATTAGGAATCATTCAAGCACAACC
AGATAAGAGTGAATCAGAGTTAGTCAGTCAAATAATAGAGCAGTTAATAAAAAAAGAAAAAGTCTACCTGGCATAGGTAC
CAGCACACAAAGGAATTGGAGGAAATGAACAAGTAGATAAATTGGTCAGTGCTGGAATCAGGAAAGNACTATTTTTAGAT
GGAATAGATAAGGCCCAAGAAGAACATGAGAAATATCACAGTAATTGGAGAGCAATGGCTAGTGATTTTAACCTACCACC
TGTAGTAGCAAAAGAAATAGTAGCCAGCTGTGATAAATGTCAGCTAAAAGGGGAAGCCATGCATGGACAAGTAGACTGTA
GCCCAGGAATATGGCAGCTAGATTGTACACATTTAGAAAGAAAAGTTATCTTAGTAGCAGTTCATGTAGCCAGTGGATAT
ATAGAAGCAGAAGTAATTCCAGCAGAGACAGGGCAAGAAACAGCATACTTCCTCTTAAAATTAGCAGGAAGATGGCCAGT
AAAAACAGTACATACAGACAATGGCAGCAATTTCACCAGTACTACAGTTAAGGCCGCCTGTTGGTAGGCGAGGATCAAGC
AGGAATTTGGCATTCCCTACAATCCCCAAAGTCAAGGAGTAATAGAATCTATGAATAAAGAATTAAAGAAAATTATAGGA
CAGGTAAGAGATCAGGCTGAACATCTTAAGACAGCAGTACAAATGGCAGTATTCATCCACAATTTTAAAAGAAAAGGGGG
GATTGGGGGGTACAGTGCAGGGGAAAGAATAGTAGACATAATAGCAACAGACATACAAACTAAAGAATTACAAAAACAAA
TTACAAAAATTCAAAATTTTCGGGTTTATTACAGGGACAGCAGAGATCCAGTTTGGAAAGGACCAGCAAAGCTCCTCTGG
AAAGGTGAAGGGGCAGTAGTAATACAAGATAATAGTGACATAAAAGTAGTGCCAAGAAGAAAAGCAAAGATCATCAGGGA
TTATGGAAAACAGATGGCAGGTGATGATTGTGTGGCAAGTAGACAGGATGAGGATTAACACATGGAAAAGATTAGTATAA
TAGCATATGTATATTTCAAGGAAAGCTAAGGACTGGTTTTATAGACATCACTATGAAAGTACTAATCCAAAAATAAGTTC
AGAAGTACACATCCCACTAGGGGATGCTAAATTAGTAATAACAACATATTGAGGTCTGCATACAGGAGAAAGAGACTGGC
ATTTGGGTCAGGGAGTCTCCATAGAATGGAGGAAAAAGAGATATAGCACACAAGTAGACCCTGACCTAGCAGACCAACTA
ATTCATCTGCACTATTTTGATTGTTTTTCAGAATCTGCTATAAGAAATACCATATTAGGACGTATAGTTAGTCCTAGGTG
TGAATATCAAGCAGGACATAACAAGGTAGGATCTCTACAGTACTTGGCACTAGCAGCATTAATAAAACCAAAACAGATAA
AGCCACCTTTGCCTAGTGTTAGGAAACTGACAGAGGACAGATGGAACAAGCCCCAGAAGACCAAGGGCCACAGAAGGAGC
CATACAATGAATGGACACTGGAGCTTTTAGAGGAACTTAAGAGTGAAGCTGTTAGACATTTTCCTAGGATATGGCTCCAT
AACTTAGGACAACATATCTATGAAACTTACGGGGATACTTGGGCGGGAGTGGAAGCCATAATAAGAATTCTGCAACAACT
```

GCTGTTTATCCATTTCAGAATTAGGTGTCGACATAGCAGAATAGGCGTTACTCGACAGAGGAGAGCAAGAAATGGAGCCA
GTAGATCCTAGACTAGAGCCCTAGAAGCATCCAGGAAGTCAGCCTAAAACTGCTTGTACCAATTGCTATTGTAAAAAGTG
TTGCTTTCATTGCCAAGTTTGTTTCATAACAAAAGCCTTAGGCATCTCCTATGGCAGGAAGAAGCGGAGACAGCGACGAA
GACCTCCTCAAGGCAGTCAGACTCATCAAGTTTCTCTATCAAAGCAGTAAGTAATACATGTAATGCAACCTATACAAATA
GCAATAGTAGCATTAGTAGTAGCAATAATAATAGCAATAGTTGTGTGGTCCATAGTAATCATAGAATATAGGAAAATATT
AAGACAAAGAAAAATAGACAGGTTAATTGATAGACTAATAGAAAGAGCAGAAGACAGTGGCAATGAGAGTGAAGGAGAAA
TATCAGCACTTGTGGAGATGGGGGTGGAGATGGGGCACCATGCTCCTTGGGATGTTGATGATCTGTAGTGCTACAGAAAA
ATTGTGGGTCACAGTCTATTATGGGGTACCTGTGTGGAAGGAAGCAACCACCACTCTATTTTGTGCATCAGATGCTAAAG
CATATGATACAGAGGTACATAATGTTTGGGCCACACATGCCTGTGTACCCACAGACCCCAACCCACAAGAAGTAGTATTG
GTAAATGTGACAGAAAATTTTAACATGTGGAAAAATGACATGGTAGAACAGATGCATGAGGATATAATCAGTTTATAGGA
TCAAAGCCTAAAGCCATGTGTAAAATTAACCCCACTCTGTGTTAGTTTAAAGTGCACTGATTTGAAGAATGATACTAATA
CCAATAGTAGTAGCGGGAGAATGATAATGGAGAAAGGAGAGATAAAAAACTGCTCTTTCAATATCAACACAAGCATAAGA
GGTAAGGTGCAGAAAGAATATGCATTTTTTTATAAACTTGATATAATACCAATAGATAATGATACTACCAGCTATACGTT
GACAAGTTGTAACACCTCAGTCATTACACAGGCCTGTCCAAAGGTATCCTTTGAGCCAATTCCCATACATTATTGTGCCC
CGGCTGGTTTCGCGATTCTAAAATGTAATAATAAGACGTTCAATGGAACAGGACCATGTACAAATGTCAGCACAGTACAA
TGTACACATGGAATTAGGCCAGTAGTATCAACTCAACTGCTGTTAAATGGCAGTCTAGCAGAAGAAGAGGTAGTAATTAG
ATCTGTCAATTTCACGGACAATGCTAAAACCATAATAGTACAGCTGAACACATCTGTAGAAATTAATTGTACAAGACCCA
ACAACAATACAAGAAAAAAAATCCGTATCCAGAAGGGACCAGGGAGAGCATTTGTTACAATAGGAAAAATAGGAAATATG
AGACAAGCACATTGTAACATTAGTAGAGCAAAATGGAATGCCACTTTAAAACAGATAGCTAGCAAATTAAGAGAACAATT
TGGAAATAATAAAACAATAATCTTTAAGCAATCCTCAGGAGGGGACCCAGAAATTGTAACGCACAGTTTTAATTGTGGAG
AGGAATTTTTCTACTGTAATTCAACACAACTGTTTAATAGTACTTGGTTTAATAGTACTTGGAGTACTGAAAGGTCAAAT
AACACTGAAGGAAGTGACACAATCACACTCCCATGCAGAATAAAACAATTTATAAACATGTGGCAGGAAGTAGGAAAAGC
AATGTATGCCCCTCCCATCAGCGGACAAATTAGATGTTCATCAAATATTACAGGGCTGCTATTAACAAGAGATGGTGGTA
ATAACAACAATGGGTCCGAGATCTTCAGACCTGGAGGAGGAGATATGAAGGACAATTGGAGAAGTGAATTATATAAATAT
AAAGTAGTAAAAATTGAACCATTAGGAGTAGCACCCACCAAGGCAAAGAGAAGAGTAGTGCAGAGAGAAAAAAGAGCAGT
AGGAATAGGAGCTTTGTTCCTTAGGTTCTTAGGAGCAGCAGGAAGCACTATAGGCGCAGCGTCAATGACGCTGACGGTAC
AGGCCAGACAATTATTGTCTGGTATAGTGCAGCAGCAGAACAATTTGCTGAGGGCTATTGAGGCGCAACAGCATCTGTTG
CAACTCACAGTCTGAGGCATCAAGCAGCTCCAGGCAAGAATCCTGGCTGTGGAAAGATACCTAAAGGATCAACAGCTCCT
GGGGATTTGAGGTTGCTCTGGAAAACTCATTTGCACCACTGCTGTGCCTTGGAATGCTAGTTGGAGTAATAAATCTCTGG
AACAGATTTGGAATCACACGACCTGGATGGAGTAGGACAGAGAAATTAACAATTACACAAGCTTAATACACTCCTTAATT
GAAGAATCGCAAAACCAGCAAGAAAAGAATGAACAAGAATTATTAGAATTAGATAAATGGGCAAGTTTGTGGAATTGGTT
TAACATAACAAATTGGCTGTGGTATATAAAATTATTCATAATGATAGTAGGAGGCTTGGTAGGTTTAAGAATAGTTTTTG
CTGTACTTTCTGTAGTGAATAGAGTTAGGCAAGGATATTCACCATTATCGTTTCAGACCCACCTCCCAATCCCGAGGGGA
CCCGACAGGCCCGAAGGAATAGAAGAAGAAGGTGGAGAGAGAGACAGAGACAGATCCATTCGATTAGTGAACGGATCCTT
AGCACTTATCTAGGACGATCTGCGGAGCCTGTGCCTCTTCAGCTACCACCGCTTGAGAGACTTACTCCTGATTGTAACGA
GGATTGTGGAACTTCTAAGACGCAGGGGGTGAGAAGCCCTCAAATATTGGTAAAATCTCCTACAATATTAGAGTCAGGAG
CTAAAGAATAGTGCTGTTAGCTTGCTCAATGCCACAGCTATAGCAGTAGCTGAGAGGACAGATAAGGTTATAGAAGTAGT
ACAAGAAGCTTATAGAGCTATTCGCCACATACCTAGAAGAATAAGACAGGGCTTAGAAAGGATTTTGCTATAAGATAAGT
GGCAAGTGGTCAAAAAGTAGTGTGGTTAGATGGCCTGCTGTAAGGGAAAGAATGAGACGAGCTGAGCCAGCAGCAGATGG
GGTGGGAGCAGCATCTCAAGACCTAGAAAAACATAGAGCAATCACAAGTAGCAACACAGCAGCTAACAATGCTGCTTGTG
CCTGGCTAGAAGCACAAGAGGAGGAGAAGGTAGGTTTTCCAGTCACACCTCAGGTACCTTTAAGACCAATGACTTACAAG
GCAGCTGTAGATCTTAGCCACTTTTTAAAAGAAAAGGGGGGACTGGAAGGGCTAATTCACTCCCAACGAAGACAAGATAT
CCTTGATCTGTGGATCTACCACACACAAGGCTACTTCCCTGATTGGCAGAACTACACACCAGGACCAGGGATCAGATATC
CACTGACCTTTGGATGGCGCTACAAGCTAGTACCAGTTGAGCCAGAGAAGTTAGAAGAAGCCAACAAAGGAGAGAACACC
AGCTTGTTACACCCTGTGAGCCTGCATGGAATGGATGACCCGGAGAGAGAAGTGTTAGAGTGGAGGTTTGACAGCCGCCT
AGCATTTCATCACGTGGCCCGAGAGCTGCATCCGGAGTACTTCAAGAACTGCTGATATCGAGCTTGCTACAAGGGACTTT
CCGCTGAGGACTTTCCAGGGAGGCGTGGCCTGGGCAGGACTGGGGAGTGGCGAGCCCTCAGATCCTGCATATAAGCAGCT
GCTTTTTGCCTGTACTAGGTCTCTCTGGTTAGACCAGATCTGAGCCTAGGAGCTCTCTGGCTAACTAAGGAACCCACTGC
TTAAGCCTCAATAAAGCTTGCCTTGAGTGCTTCAAGTAGTGTGTGCCCATCTGTTGTGTGACTCTAGTAACTAGAGATCC
CTCA

>3-78LONG

---AAAAGTAGTAGAAGAGAAGGCTT
TCGGCCCAGAAGTGATACCCATGTTTTCAGCATTATCAGAAGGAGCCACCCCACAAGATTTAAACACCATGCTAAACACA
GTGGGGGGACATCAAGCAGCCATGCAAATGTTAAAAGAGACCATCAATGAGGAAGCTGCAGAATGGGATAGAGTGCATCC
AGTGCATGCAGGGCCTATTGCACCAGGCCAGATGAGAGAACCAAGGGGAAGTGACATAGCAGGAACTACTAGTACCCTTC
AGGAACAAATAGGATGGATGACACATAATCCACCTATCCCAGTAGGAGAAATCTATAAAAGATGGATAATCCTGGGATTA
AATAAAATAGTAAGAATGTATAGCCCTACCAGCATTCTGGACATAAGACAAGGACCAAAGGAACCCTTTAGAGACTATGT
AGACCGATTCTATAAAACTCTAAGAGCCGAGCAAGCTTCACAAGAGGTAAAAAATTGGATGACAGAAACCTTGTTGGTCC
AAAATGCGAACCCAGATTGTAAGACTATTTTAAAAGCATTAGGACCAGAAGCGACACTAGAAGAAATGATGACAGCATGT
CAGGGAGTGGGGGGACCCGGCCATAAAGCAAGAGTTTTGGCTGAAGCAATGAGCCAAGTAACAAATCCAGCTACCATAAT
GATACAGAAAGGCAATTTTAGGAACCAAAGAAAGACTGTTAAGTGTTTCAATTGTGGCAAAGAAGGGCACATAGCCAAAA
ATTGCAGGGCCCCTAGGAAAAAGGGCTGTTGGAAATGTGGAAAGGAAGGACACCAAATGAAAGATTGTACTGAGAGACAG
GCTAATTTTTTAGGGAAGATCTGGCCTTCCCACAAGGGAAGGCCAGGGAATTTTCTTCAGAGCAGACCAGAGCCAACAGC
CCCACCAGAAGAGAGCTTCAGGTTTGGGGAAGAGACAACAACTCCCTCTCAGAAGCAGGAGCCGATAGACAAGGAACTGT
ATCCTTTAGCTTCCCTCAGATCACTCTTTGGCAGCGACCCCTCGTCACAATAAAGATAGGGGGGCAATTAAAGGAAGCTC
TATTAGATACAGGAGCAGATGATACAGTATTAGAAGAAATGAATTTGCCAGGAAGATAGAAACCAAAAATGATAGGGGGA
ATTAGAGGTTTTATCAAAGTAAGACAGTATGATCAGATACTCATAGAAATCTGCGGACATAAAGCTATAAGTACAGTATT
AGTAGGACCTACACCTGTCAACATAATTGGAAGAAATCTGTTGACTCAGATTGGCTGCACTTTAAATTTTCCCATTAGTC
CTATTGAGACTGTACCAGTAAAATTAAAGCCAGGAATGGATGGCCCAAAAGTTAAACAATGGCCATTGACAGAAGAAAAA
ATAAAAGCATTAGTAGAAATTTGTACAGAAATGGAAAAGGAAGGAAAAATTTCAAAAATTAGGCCTGAAAATCCATACAA
TACTCCAGTATTTGCCATAAAGAAAAAAGACAGTACTAAATGGAGAAAATTAGTAGATTTCAGAGAACTTAATAAGAGAA
CTCAAGATTTCTAGGAAGTTCAATTAAGAATACCACATCCTGCAGGGTTAAAACAGAAAAAATCAGTAACAGTACTAGAT
GTGGGCGATGCATATTTTTCAGTTCCCTTAGATAAAGACTTCAGGAAGTATACTGCATTTACCATACCTAGTATAAACAA
TGAGACACCAGGGATTAGATATCAGTACAATGTGCTTCCACAGGGATGGAAAGGATCACCAGCAATATTCCAGTGTAGCA
TGACAAAAATCTTAGAGCCTTTTAGAAAACAAAATCCAGACATAGTCATCTATCAATACATAGATGATTTGTATGTAGGA
TCTGACTTAGAAATAGGGCAGCATAGAACAAAAATAGAGGAACTGAGACAACATCTGTTAAGGTGAGGATTTACCACACC
AGACAAAAAACATCAGAAAGAACCTCCATTCCTTTGGATAGGTTATGAACTCCATCCTG---------------------
----

--------------------GAAAGACTCCTAAATTT-AAATTACCCATACAAAAGGAAACATAGGAAGCATGGTGGACA
GAGTATTGGCAAGCCACCTAGATTCCTGAGTAGGAGTTTGTCAATACCCCTCCCTTAGTGAAGTTATGGTACCAGTTAGA
GAAAGAACCCATAATAGGAGCAGAAACTTTCTATGTAGATAGGGCAGCCAATAAGGAAACTAAATTAGGAAAAGCAGGAT
ATGTAACTGACAGAGGAAGACAAAAAGTTGTCCCCCTAACGGACACAACAAATCAGAAGACTGAGTTACAAGCAATTCAT
CTAGCTTTGCAAGATTCAGGATTAGAAGTAAACATAGTGACAGACTCACAATATGCATTAAGAATCATTCAAGCACAACC
AGATAAGAGTGAATCAGAGTTAGTCAGTCAAATAATAGAGCAGTTAATAAAAAAGGAAAAAGTCTACCTGGCATAAGTAC
CAGCACACAAAGGAATTGGAGGAAATGAACAAGTAGATAAATTAGTCAGTGCTAGAATCAGAAAAGTACTATTTTTAGAT
GGAATAGATAAGGCCCAAGAAGAACATGAGAAATATCACAGTAATTAGAGAGCAATGGCTAGTGATTTTAACCTACCACC
TGTAGTAGCAAAAGAAATAGTAGCCAGCTGTGATAAATGTCAGCTAAAAGGGGAAGCCATGCATGGACAAGTAGACTGTA
GCCCAGGAATATGGCAGCTAGATTGTACACATTTAGAAGGAAAAGTTATCTTAGTAGCAGTTCATGTAGCCAGTGGATAT
ATAGAAGCAGAAGTAATTCCAGCAGAGACAGGGCAAGAAACAGCATACTTCCTCTTAAAATTAGCAGGAAGATGGCCAGT
AAAAACAGTACATACAGACAATGGCAGCAATTTCACCAGTACTACAGTTAAGGCCGCCTGTTAGTAGGCGAGGATCAAGC
AGGAATTTGGCATTCCCTACAATCCCCAAAGTCAAGGAGTAATAGAATCTATGAATAAAGAATTAAAGAAAATTATAGGA
CAGGTAAGAGATCAGGCTGAACATCTTAAGACAGCAGTACAAATGGCAGTATTCATCCACAATTTTAAAAGAAAAGGGGG
GATTGGGGGGTACAGTGCAGGGGAAAGAATAGTAGACATAATAGCAACAGACATACAAACTAAAGAATTACAAAAACAAA
TTACAAAAATTCAAAATTTTCGAGTTTATTACAGGGACAGCAGAGATCCAGTTTAGAAAGGACCAGCAAAGCTCCTCTGG
AAAGGTGAAGGGGCAGTAGTAATACAAGATAATAGTGACATAAAAGTAGTGCCAAGAAGAAAAGCAAAGATCATCAGGGA
TTATAGAAAACAGATGGCAGGTGATGATTGTGTGGCAAGTAGACAGGATGAGGATTAACACATAGAAAAGATTAGTATAA
TAGCATATGTATATTTCAAGGAAAGCTAAGGACTGGTTTTATAGACATCACTATGAAAGTACTAATCCAAAAATAAGTTC
AGAAGTACACATCCCACTAGGGGATGCTAAATTAGTAATAACAACATATTAGGGTCTGCATACAGGAGAAAGAGACTGGC
ATTTAGGTCAGGGAGTCTCCATAGAATGGAGGAAAAAGAGATATAGCACACAAGTAGACCCTGACCTAGCAGACCAACTA
ATTCATCTGCACTATTTTGATTGTTTTTCAGAATCTGCTATAAGAA---------------------------------G
TGAATATCAAGCAGGACATAACAAGGTAGGATCTCTACAGTACTTGGCACTAGCAGCATTAATAAAACCAAAACAGATAA
AGCCACCTTTGCCTAGTGTTAGGAAACTGACAGAGGACAGATGGAACAAGCCCCAGAAGACCAAGGGCCACAGAGGGAGC
CATACAATGAATGGACACTAGAGCTTTTAGAGGAACTTAAGAGTGAAGCTGTTAGACATTTTCCTAAGATATGGCTCCAT
AACTTAGGACAACATATCTATGAAACTTACAAAGATACTTAGGCAGGAGTGGAAGCCATAATAAGAATTCTGCAACAACT
GCTGTTTATCCATTTCAGAATTAGGTGTCGACATAGCAGAATAGGCGTTACTCGACAGAGGAGAGCAAGAAATAGAGCCA
GTAGATCCTAGACTAGAGCCCTAGAAGCATCCAGGAAGTCAGCCTAAAACTGCTTGTACCAATTGCTATTGTAAAAAGTG
TTGCTTTCATTGCCAAGTTTGTTTCATAACAAAAGCCTTAGGCATCTCCTATGGCAGGAAGAAGCGGAGACAGCGACGAA
GACCTCCTCAAGGCAGTCAGACTCATCAAGTTTCTCTATCAAAGCAGTAAGTAATACATGTAATGCAACCTATACAAATA
GCAATAGTAGCATTAGTAGTAGCAATAATAATAGCAATAGTTGTGTGGTCCATAGTAATCATAGAATATAAGAAAATATT
AAGACAAAGAAAAATAGACAGGTTAATTGATAGACTAATAGAAAGAGCAGAAGACAGTGGCAATGAGAGTGAAGGAGAAA
TATCAGCACTTGTGGAGATGGGGGTGGAGATAGGGCACCATGCTCCTTAGGATGTTGATGATCTGTAGTGCTACAGAAAA
ATTGTAGGTCACAGTCTATTATGAGGTACCTGTGTAGAAGGAAGCAACCACCACTCTATTTTGTGCATCAGATGCTAAAG
CATATGATACAGAGGTACATAATGTTTAGGCCACACATGCCTGTGTACCCACAGACCCCAACCCACNAGAAGTAGTATTA
GTAAATGTGACAGAAAATTTTAACATGTGGAAAAATGACATAGTAGAACAGATGCATGAGGATATAATCAGTTTATAAGA
TCAAAGCCTAAAGCCATGTGTAAAATTAACCCCACTCTGTGTTAGTTTAAAGTGCACTGATTTGAAGAATGATACTAATA
CCAATAGTANTAGCGGGAGAATGATAATGGAGAAAGGAGAGATAAAAAACTGCTCTTTCAATATCAGCACAAGCATAAGA
GGTAAGGTGCAGAAAGAATATGCATTTTTTTATAAACTTGATATAATACCAATAGATAATGATACTACCAGCTATACGTT
GACAAGTTGTAACACCTCAGTCATTACACAGGCCTGTCCAAAGGTATCCTTTGAGCCAATTCCCATACATTATTGTGCCC
CGGCTGGTTTTGCGATTCTAAAATGTAATAATAAGACGTTCAATAGAACAGGACCATGTACAAATGTCAGCACAGTACAA
TGTACACATAGAATTAGGCCAGTAGTATCAACTCAACTGCTGTTAAATGGCAGTCTAGCAGAAGAAGAGGTAGTAATTAG
ATCTGTCAATTTCACGGACAATGCTAAAACCATAATAGTACAGCTGAACACATCTGTAGAAATTAATTGTACAAGACCCA
ACAACAATACAAGAAAAAAAATCCGTATCCAGAGGGGACCAGGGAGAGCATTTGTTACAATAGGAAAAATAGGAAATATG
AGACAAGCACATTGTAACATTAGTAGAGCAAAATGGAATGCCACTTTAAAACAGATAGCTAGCAAATTAAGAGAACAATT
TGGAAATAATAAAACAATAATCTTTAAGCAATCCTCAGGAGGGGACCCAGAAATTGTAACGCACAGTTTTAATTGTGGAG
GGGAATTTTTCTACTGTAATTCAACACAACTGTTTAATAGTACTTAGTTTAATAGTACTTGGAGTACTGAAAGGTCAAAT
AACACTGAAGGAAGTGACACAATCACACTCCCATGCAGAATAAAACAATTTATAAACATGTGGCAAGAAGTAGGAAAAGC
AATGTATGCCCCTCCCATCAGCGGACAAATTAGATGTTCATCAAATATTACAGGGCTGCTATTAACAAGAGATGGTGGTA
ATAACAACAATAAGTCCGAGATCTTCAGAC?TGGAGGAGGAGATATGAAGGACAATTGGAGAAGTGAATTATATAAATAT
AAAGTAGTAAAAATTGAACCATTAGGAGTAGCACCCACCAAGGCAAAGAGAAGAGTGGTGCAGAGAGAAAAAAGAGCAGT
AAGAATAAGAGCTTTGTTCCTTAGGTTCTTAGGAGCAGCAAGAAGCACTATAGGCGCAGCGTCAATGACGCTGACGGTAC
AGGCCAGACAATTATTGTCTGGTATAGTGCAGCAGCAGAACAATTTGCTGAGGGCTATTGAGGCGCAACAGCATCTGTTG
CAACTCACAGTCTGGGGCATCAAGCAGCTCCAGGCAAGAATCCTGGCTGTAGAAAGATACCTAAAGGATCAACAGCTCCT
GGGGATTTGGGGTTGCTCTGGAAAACTCATTTGCACCACTGCTGTGCCTTGGAATGCTAGTTGGAGTAATAAATCTCTGG
AACAGATTTAGAATCACACGACCTGGATGGAGTAGGACAGAGAAATTAACAATTACACAAGCTTAATACACTCCTTAATT
AAAGAATCGCAAAACCAGCAAGAAAAGAATGAACAAGAATTATTAGAATTAGATAAATAGGCAAGTTTGTGGAATTGGTT
TAACATAACAAATTGGCTGTGGTATATAAAATTATTCATAATGATAGTAGGAGGCTTGGTAGGTTTAAGAATAGTTTTTG
CTGTACTTTCTGTAGTGAATAGAGTTAGGCAGGGATATTCACCATTATCGTTTCAGACCCACCTCCCAATCCCGAGGGGA
CCCGACAGGCCCGAAGGAATAGAAGAAGAAGGTGGAGAGAGAGACAGAGACAGATCCATTCGATTAGTGAACGGATCCTT
AGCACTTATCTGGGACGATCTGCGGAGCCTGTGCCTCTTCAGCTACCACCGCTTGAGAGACTTACTCTTGATTGTAACGA
GGATTGTGGAACTTCTAGGACGCAGGGGGTGGGAAGCCCTCAAATATTGGTGGAATCTCCTACAATATTAGAGTCAGGAG
CTAAAGAATAGTGCTGTTAGCTTGCTCAATGCCACAGCTATAGCAGTAGCTGAGGGGACAGATAAGGTTATAGAAGTAGT
ACAAGAAGCTTATAGAGCTATTCGCCACATACCTAGAAGAATAAGACAGGGCTTGGAAAGGATTTTGCTATAAGATAGGT
GGCAAGTGGTCAAAAAGTAGTGTAGTTGGATGGCCTGCTGTAAGGGAAAGAATGAGACGAGCTGAGCCAGCAGCAGATGG
GGTGGGAGCAGCATCTCGAGACCTAGAAAAACATGGAGCAATCACAAGTAGCAACACAGCAGCTAACAATGCTGCTTGTG
CCTGGCTAGAAGCACAAGAGGAGGAGAAGGTAAGTTTTCCAGTCACACCTCAGGTACCTTTAAGACCAATGACTTACAAG
GCAGCTGTAGATCTTAGCCACTTTTTAAAAGAAAAGGGGGGACTGGAAAGGCTAATTCACTCCCAACGAAGACAAGATAT
CCTTGATCTGTGGATCTACCACACACAAGGCTACTTCCCTGATTGGCAGAACTACACACCAGGACCAGGGATCAGATATC
CACTGACCTTTGGATGGCGCTACAAGCTAGTACCAGTTGAGCCAGAGAAGTTAGAAGAAGCCAACAAAGGAGAGAACACC
AGCTTGTTACACCCTGTGAGCCTGCATAGAATAGATGACCCGGAGAGAGAAGTGTTAGAGTGGAGGTTTGACAGCCGCCT
AGCATTTCATCACGTGGCCCGAGAGCTGCATCCAGAGTACTTCAAGAACTGCTGATATCGAGCTTGCTACAAAGGACTTT
CCGCTGAGGACTTTCCAGGGAGGCGTGGCCTAGGCGGGACTAAGGAGTGGCGAGCCCTCAGATCCTGCATATAAGCAGCT
GCTTTTTGCCTGTACTAGGTCTCTCTGGTTAGACCAGATCTGAGCCTAGGAGCTCTCTGGCTAACTAAGGAACCCACTGC
TTAAGCCTCAATAAAGCTTGCCTTAAGTGCTTCAAGTAGTGTGTGCCCATCTGTTGTGTGACTCTGGTAACTAGAGATCC
CTCA
>3-38LONG


```
-----------------------------------------------------ACCCTATAGTGCAGAAC
ATCCAGGGGCAA-ATGGTACATCANGCCATATCACCTAGAACTTTAAATGCATAGGTAAAAGTAGTAGAAGAGAAGGCTT
TCAGCCCAGAAGTGATACCCATGTTTTCAGCATTATCAGAAGGAGCCACCCCACAAGATTTAAACACCATGCTAAACACA
GTGAGGGGACATCAAGCAGCCATGCAAATGTTAAAAGAGACCATCAATGAGGAAGCTGCAGAATAGGATAAAGTGCATCC
AGTGCATGCAGGGCCTATTGCACCAGGCCAGATGAGAGAACCAAGGGGAAGTGACATAGCAGGAACTACTAGTACCCTTC
AGGAACAAATAGGATAGATGACACATAATCCACCTATCCCAGTAGGAGAAATCTATAAAAGATGGATAATCCTAGGATTA
AATAAAATAGTAAGAATGTATAGCCCTACCAGCATTCTGGACATAAGACAAGGACCAAAGGAACCCTTTAGAGACTATGT
AGACCGATTCTATAAAACTCTAAGAGCCGAGCAAGCTTCACAAGAGGTAAAAAATTAGATGACAGAAACCTTGTTGGTCC
AAAATGCGAACCCAGATTGTAAGACTATTTTAAAAGCATTAGGACCAGGAGCGACACTAGAAGAAATGATAACAGCATGT
CAGGGAGTGGGGGGACCCGGCCATAAAGCAAGAGTTTTGGCTGAAGCAATGAGCCAAGTAACAAATCCAGCTACCATAAT
GATACAGAAAGGCAATTTTAGGAACCAAAGAAAGACTGTTAAGTGTTTCAATTGTGGCAAAGAAGGGCACATAGCCAAAA
ATTGCAGGGCCCCTAAGAAAAAGGGCTGTTAGAAATGTAGAAAGGAAGGACACCAAATGAAAGATTGTACTGAGAGACAG
GCTAATTTTTTAGGGAAGATCTGGCCTTCCCACAAAGGAAGGCCAAGGAATTTTCTTCAGAGCAGACCAGAGCCAACAGC
CCCACCAGAAGAGAGCTTCAGGTTTGGGGAAGAGACAACAACTCCCTCTCAGAAGCAGGAGCCGATAGACAAGGAACTGT
ATCCTTTAGCTTCCCTCAGATCACTCTTTGGCAGCGACCCCTCGTCACAATAAAGATAGGGGGGCAATTAAAGGAAGCTC
TATTAGATACAGGAGCAGATGATACAGTATTAGAAGAAATGAATTTGCCAGGAAGATAGAAACCAAAAATGATAGGAGGA
ATTGGAGGTTTTATCAAAGTAAGACAGTATGATCAGATACTCATAGAAATCTGCGGACATAAAGCTATAAGTACAGTATT
AGTAAGACCTACACCTGTCAACATAATTGGAAGAAATCTGTTGACTCAGATTGGCTGCACTTTAAATTTTCCCATTAGTC
CTATTGAGACTGTACCAGTAAAATTAAAGCCAAGAATAGATGGCCCAAAAGTTAAACAATGGCCATTGACAGAAGAAAAA
ATAAAAGCATTAGTAAAAATTTGTACAGAAATAGAAAAAGAAAGAAAAATTTCAAAAATTAGGCCTGAAAATCCATACAA
TACTCCAGTATTTGCCATAAAGAAAAAAGACAGTACTAAATGGAGAAAATTAGTAGATTTCAGAGAACTTAATAAGAGAA
CTCAAAATTTCTAGGAAGTTCAATTAGGAATACCACATCCTGCAAGGTTAAAACAGAAAAAATCAGTAACAGTACTAGAT
GTAGGCGATGCATATTTTTCAGTTCCCTTAGATAAAGACTTCAGGAAGTATACTGCATTTACCATACCTAGTATAAACAA
TGAGACACCAGGGATTAGATATCAGTACAATGTGCTTCCACAAGGATAGAAAAGATCACCAGCAATATTCCAGTGTAGCA
TGACAAAAATCTTAGAGCCTTTTAGAAAACAAAATCCAGACATAGTCATCTATCAATACATAGATGATTTGTATGTAAGA
TCTGACTTAGAAATAGGGCAGCATAGAACAAAAATAGAGGAACTGAGAC---------------------
----------------------------------------------------------------------
----------------------------------------------------------------------
----------------------------------------------------------------------
----------------------------------------------------------------------
------------------------------------TCAAATTTATCAAGAGCCATTTAAAAATNTGAAA
ACAGGAAAGTATGCAAGAATGAAAGGTGCCCACACTAATGATGTGAAACAATTAACAGAGGCAGTACAAAAAATAGCCAC
AGAAAGCATAGTAATATGGGGAAAGACTCCTAAATTTTAAATTACCCATACAAAAGGAAACATGGGAAGCATGGTGGACA
GAGTATTGGCAAGCCACCTGGATTCCTGAGTAGGAGTTTGTCAATACCCCTCCCTTAGTGAAGTTATGGTACCAGTTAGA
GAAAGAACCCATAATAGGAGCAGAAACTTTCTATGTAGATGGGGCAGCCAATAGGGAAACTAAATTAGGAAAAGCAGGAT
ATGTAACTGACAGAGGAAGACAAAAAGTTGTCCCCCTAACGGACACAACAAATCAGAAGACTGAGTTACAAGCAATTCAT
CTAGCTTTGCAGGATTCAGGATTAGAAGTAAACATAGTGACAGACTCACAATATGCATTAGGAATCATTCAAGCACAACC
AGATAAGAGTGAATCAGAGTTAGTCAGTCAAATAATAGAGCAGTTAATAAAAAAGGAAAAAGTCTACCTGGCATAGGTAC
CAGCACACAAAGGAATTGGAGGAAATGAACAAGTAGATAAATTGGTCAGTGCTAGAATCAGGAAAGTACTATTTTTAGAT
AGAATAGATAAGGCCCAAGAAGAACATGAGAAATATCACAGTAATTGGAGAGCAATGGCTAGTGATTTTAACCTACCACC
TGTAGTAGCAAAAGAAATAGTAGCCAGCTGTGATAAATGTCAGCTAAAAGGGGAAGCCATGCATGGACAAGTAGACTGTA
GCCCAGGAATATGGCAGCTAGATTGTACACATTTAGAAGGAAAAGTTATCTTAGTAGCAGTTCATGTAGCCAGTGGATAT
ATAGAAGCAGAAGTAATTCCAGCAGAGACAGGGCAAGAAACAGCATACTTCCTCTTAAAATTAGCAGGAAGATGGCCAGT
AAAAACAGTACATACAGACAATGGCAGCAATTTCACCAGTACTACAGTTAAGGCCGCCTGTTGGTGGGCGGGGATCAAGC
AGGAATTTGGCATTCCCTACAATCCCCAAAGTCAAGGAGTAATAGAATCTATGAATAAAGAATTAAAGAAAATTATAGGA
CAGGTAAGAGATCAGGCTGAACATCTTAAGACAGCAGTACAAATGGCAGTATTCATCCACAATTTTAAAAGAAAAGGGGG
GATTGGGGGGTACAGTGCAGGGGAAAGAA-----------------------------------------
----------------------------------------------------------------------
----------------------------------------------------------------------
----------------------------------------------------------------------
----------------------------------------------------------------------
----------------------------------------------------------------------
----------------------------------------------------------------------
----------------------------------------------------------------------
----------------------------------------------------------------------
----------------------GAGGACAGATAGAACAAGCCCCAGAAGACCAAGGGCCACAGAGGGAGC
CATACAATGAATGGACACTAGAGCTTTTAGAGGAACTTAAGAGTGAAGCTGTTAGACATTTTCCTAGGATATGGCTCCAT
AACTTAGGACAACATATCTATGAAACTTACGAGGATACTTGGGCAGGAGTAGAAGCCATAATAAGAATTCTGCAACAACT
GCTGTTTATCCATTTCAGAATTAGGTGTCGACATAGCAGAATAGGCGTTACTCGACAGAGGAGAGCAAGAAATGGAGCCA
GTAGATCCTAGACTAGAGCCCTGGAAGCATCCAGGAAGTCAGCCTAAAACTGCTTGTACCAATTGCTATTGTAAAAAGTG
TTGCTTTCATTGCCAAGTTTGTTTCATAACAAAAGCCTTAGGCATCTCCTATGGCAGGAAGAAGCGGAGACAGCGACGAA
GACCTCCTCAAGGCAGTCAGACTCATCAAGTTTCTCTATCAAAGCAGTAAGTAATACATGTAATGCAACCTATACAAATA
GCAATAGTAGCATTAGTAGTAGCAATAATAATAGCAATAGTTGTGTAGTCCATAGTAATCATAGAATATAGGAAAATATT
AAGACAAAGAAAAATAGACAGGTTAATTGATAGACTAATAGAAAGAGCAGAAGACAGTGGCAATGAGAGTGAAGGAGAAA
TATCAGCACTTGTGGAGATGGGGGTGGAGATGGGGCACCATGCTCCTTAGGATGTTGATGATCTGTAGTGCTACAGAAAA
ATTGTGGGTCACAGTCTATTATGAGGTACCTGTGTGGAAGGAAGCAACCACCACTCTATTTTGTGCATCAGATGCTAAAG
CATATGATACAGAGGTACATAATGTTTAGGCCACACATGCCTGTGTACCCACAGACCCCAACCCACAAGAAGTAGTATTA
GTAAATGTGACAGAAAATTTTAACATGTGGAAAAATGACATGGTAGAACAGATGCATGAGGATATAATCAGTTTATAGGA
TCAAAGCCTAAAGCCATGTGTAAAATTAACCCCACTCTGTGTTAGTTTAAAGTGCACTGATTTGAAGAATGATACTAATA
CCAATAGTAGTAGCGGGAGAATGATAATAGAGAAAGGAGAGATAAAAAACTGCTCTTTCAATATCAGCACAAGCATAAGA
GGTAAGGTGCAGAAAGAATATGCATTTTTTTATAAACTTGATATAATACCAATAGATAATGATACTACCAGCTATACGTT
GACAAGTTGTAACACCTCAGTCATTACACAGGCCTGTCCAAAAGTATCCTTTGAGCCAATTCCCATACATTATTGTGCCC
CGGCTGGTTTTGCGATTCTAAAATGTAATAATAANACGTNCAATAGAACAGGACCATGTACAAGTGTCAGCACAGTACAA
TGTACACATGGAATTAGGCCAGTAGTATCAACTCAACTGCTGTTAAATGGCAGTCTAGCAGAAGAAGAGGTAGTAATTAG
ATCTGTCAATTTCACGGACAATGCTAAAACCATAATAGTACAGCTGAACACATCTGTAGAAATTAATTGTACAAGACCCA
ACAACAATACAAGAAAAAAAATCCGTATCCAGAGGGGACCAGGGAGAGCATTTGTTACAATAGGAAAAATAGGAAATATG
AGACAAGCACATTGTAACATTAGTAGAGCAAAATGGAATGCCACTTTAAAACAGATAGCTAGCAAATTAAGAGAACAATT
TGGAAATAATAAAACAATAATCTTTAAGCAATCCTCAGGAGGGGACCCAGAAATTGTAACGCACAGTTTTAATTGTGGAG
GGGAATTTTTCTACTGTAATTCAACACAACTGTTTAATAGTACTTGGTTTAATAGTACTTAGAGTACTGAAGGGTCAAAT
AACACTGAAAGAAGTGACACAATCACACTCCCATGCAGAATAAAACAATTTATAAACATGTGGCAGGAAGTAGGAAAAGC
AATGTATGCCCCTCCCATCAGCGGACAAATTAGATGTTCATCAAATATTACAGGGNNGCTATTAACAAGAGATGGTGGTA
ATAACAACAATGGGTCCGAGATCTTCAGACCTGGAGGAGGAGATATGAAGGACAATTGGAGAAGTGAATTATATAAATAT
AAAGTAGTAAAAATTGAACCATTAGGAGTAGCACCCACCAAGGCAAAGAGAAGAGTGGTGCAGAGAGAAAAAAGAGCAGT
AGGAATAGGAGCTTTGTTCCTTAGGTTCTTAGGAGCAGCAGGAAGCACTATAGGCGCAGCGTCAATGACGCTGACGGTAC
AGGCCAGACAATTATTGTCTGGTATAGTGCAGCAGCAGAACAATTTGCTGAGGGCTATTGAGGCGCAACAGCATCTGTTG
CAACTCACAGTCTGGGGCATCAAGCAGCTCCAGGCAAGAATCCTGGCTGTGGAAAGATACCTAAAGGATCAACAGCTCCT
AGGGATTTGGGGTTGCTCTAGAAAACTCATTTGCACCACTGCTGTGCCTTGGAATGCTAGTTGGAGTAATAAATCTCTGG
AACAGATTTGGAATCACACGACCTGGATGGAGTGGGACAGAGAAATTAACAATTACACAAGCTTAATACACTCCTTAATT
GAAGAATCGCAAAACCAGCAAGAAAAGAATGAACAAGAATTATTAGAATTAGATAAATGGGCAAGTTTGTAGAATTGGTT
TAACATAACAAATTGGCTGTGGTATATAAAATTATTCATAATGATAGTAGGAGGCTTGGTAAGTTTAAGAATAGTTTTTG
CTGTACTTTCTGTAGTGAATAGAGTTAGGCAAGGATATTCACCATTATCGTTTCAGACCCACCTCCCAATCCCGAGGGGA
CCCGACAGGCCCGAAGGAATAGAAGAAGAAGGTGGAGAGAGAGACAGAGACAGATCCATTCGATTAGTGAACGGATCCTT
AGCACTTATCTAGGACGATCTGCGGAGCCTGTGCCTCTTCAGCTACCACCGCTTGAGAGACTTACTCTTGATTGTAACGA
GGATTGTAGAACTTCTAGGACGCAGGGGGTGGGAAGCCCTCAAATATTGGTAGAATCTCCTACAATATTGGAGTCAGGAG
```

CTAAAGAATAGTGCTGTTAGCTTGCTCAATGCCACAGCTATAGCAGTAGCTGAAGGGACAGATAAGGTTATAGAAGTAGT
ACAAGAAGCTTATAGAGCTATTCGCCACATACCTAGAAGAATAAGACAGGGCTTGGAAAGGATTTTGCTATAAGATAGGT
GGCAAGTGGTCAAAAAGTAGTGTGGTTAGATGGCCTGCTGTAAAAGAAAGAATGAGACGAGCTGAGCCAGCAGCAGATGG
GGTGGGAGCAGCATCTCGAGACCTAGAAAAACATGGAGCAATCACAAGTAGCAACACAGCAGCTAACAATGCTGCTTGTG
CCTAGCTAGAAGCACAAGAGGAGGAGAAGGTAGGTTTTCCAGTCACACCTCAGGTACCTTTAAGACCAATGACTTACAAG
GCAGCTGTAGATCTTAGCCACTTTTTAAAAGAAAAGGGGGGACTGGAAGGGCTAATTCACTCCCAACGAAGACAAGATAT
CCTTGATCTGTGGATCTACCACACACAAGGCTACTTCCCTGATTGGCAGAACTACACACCAGGACCAGGGATCAGATATC
CACTGACCTTTGGATGGCGCTACAAGCTAGTACCAGTTGAGCCAGAGAAGTTAGAAGAAGCCAACAAAGGAGAGAACACC
AGCTTGTTACACCCTGTGAGCCTGCATGGAATGGATGACCCGGAGAGAGAAGTGTTAGAGTGGAGGTTTGACAGCCGCCT
AGCATTTCATCACGTGGCCCGAGAGCTGCATCCGGAGTACTTCAAGAACTGCTGATATCGAGCTTGCTACAAAGGACTTT
CCGCTAAGGACTTTCCAGGGAGGCGTGGCCTAGGCAAGACTGAAGAGTGGCGAGCCCTCAGATCCTGCATATAAGCAGCT
GCTTTTTGCCTGTACTAAGTCTCTCTGGTTAGACCAGATCTGAGCCTAGGAGCTCTCTGGCTAACTAGGGAACCCACTGC
TTAAGCCTCAATAAAGCTTGCCTTGAGTGCTTCAAGTAGTGTGTGCCCATCTGTTGTGTGACTCTGGTAACTAGAGATCC
CTCA

>3-14LONG

---AAAAGTAGTAGAAGAGAAGGCTT
TCAGCCCAGAAGTGATACCCATGTTTTCAGCATTATCAGAAGGAGCCACCCCACAAGATTTAAACACCATGCTAAACACA
GTGGGGGGACATCAAGCAGCCATGCAAATGTTAAAAGAGACCATCAATGAGGAAGCTGCAGAATGGGATAGAGTGCATCC
AGTGCATGCAGGGCCTATTGCACCAGGCCAGATGAGAGAACCAAGGGGAAGTGACATAGCAGGAACTACTAGTACCCTTC
AGGAACAAATAGGATGGATGACACATAATCCACCTATCCCAGTAAGAGAAATCTATAAAAGATGGATAATCCTGGGATTA
AATAAAATAGTAAGAATGTATAGCCCTACCAGCATTCTGGACATAAGACAAGGACCAAAGGAACCCTTTAGAGACTATGT
AGACCGATTCTATAAAACTCTAAGAGCCGAGCAAGCTTCACAAGAGGTAAAAAATTGGATGACAGAAACCTTGTTGGTCC
AAAATGCGAACCCAGATTGTAAGACTATTTTAAAAGCATTGGGACCAGGAGCGACACTAGAAGAAATGATGACAGCATGT
CAGGGAGTGGGGGGACCCGGCCATAAAGCAAGAGTTTTGGCTGAAGCAATGAGCCAAGTAACAAATCCAGCTACCATAAT
GATACAGAAAGGCAATTTTAGGAACCAAAGAAAGACTGTTAAGTGTTTCAATTGTGGCAAAGAAAGGCACATAGCCAAAA
ATTGCAGGGCCCCTATGAAAAAGGGCTGTTGGAAATGTGGAAAGGAAGGACACCAAATGAAAGATTGTACTAAGAGACAG
GCTAATTTTTTAGGGAAGATCTGGCCTTCCCACAAGGGAAGGCCAAGGAATTTTCTTCAGAGCAGACCAGAGCCAACAGC
CCCACCAGAAGAGAGCTTCAGGTTTGGGGAAGAGACAACAACTCCCTCTCAGAAGCAGGAGCCGATAGACAAGGAACTGT
ATCCTTTAGCTTCCCTCAGATCACTCTTTGGCAGCGACCCCTCGTCACAATAAAGATAGGGGGGCAATTAAAGGAAGCTC
TATTAGATACAGGAGCAGATGATACAGTATTAGAAGAAATGAATTTGCCAGGAAGATGGAAACCAAAAATGATAGGGGGA
ATTGGAGGTTTTATCAAAGTAAGACAGTATGATCAGATACTCATAGAAATCTGCGGACATAAAGCTATAGGTACAGTATT
AGTAGGACCTACACCTGTCAACATAATTGGAAGAAATCTGTTGACTCAGATTGGCTGCACTTTAAATTTTCCCATTAGTC
CTATTGAGACTGTACCAGTAAAATTAAAGCCAGGAATGGATGGCCCAAAAGTTAAACAATGGCCATTGACAGAAGAAAAA
ATAAAAGCATTAGTAGAAATTTGTACAGAAATGGAAAAGGAAGGAAAAATTTCAAAAATTGGGCCTGAAAATCCATACAA
TACTCCAGTATTTGCCATAAAGAAAAAAGACAGTACTAAATGGAGAAAATTAGTAGATTTCAGAGAACTTAATAAGAGAA
CTCAAGATTTCTAGGAAGTTCAATTAGGAATACCACATCCTGCAGGGTTAAAACAGAAAAAATCAGTAACAGTACTGGAT
GTGGGCGATGCATATTTTTCAGTTCCCTTAGATAAAGACTTCAGGAAGTATACTGCATTTACCATACCTAGTATAAACAA
TGAGACACCAGGGATTAGATATCAGTACAATGTGCTTCCACAGGGATGGAAAGGATCACCAGCAATATTCCAGTGTAGCA
TGACAAAAATCTTAGAGCCTTTTAGAAAACAAAATCCAGACATAGTCATCTATCAATACATGGATGATTTGTATGTACGA
TCTGACTTAGAAATAGGGCAGCATAGAACAAAAATAGAGGAACTGAGACAACATCTGTTGAGGTAAGGATTTACCACACC
AGACAAAAAACATCAGAAAGAACCTCCATTCCTTTGGATAGGTTATGAACTCCATCCTGATAAATGGACAGTACAGCCTA
TAGTGCTGCCAGAAAAGGACAGCTGGACTGTCAATGACATACAGAAATTAGTAGGAAAATTGAATTAGGCAAGTCAGATT
TATGCAGGGATTAAAGTAAGGCAATTATGTAAACTTCTTAGAGGAACCAAAGCACTAACAGAAGTAGTACCACTAACAGA
AGAAGCAGAGCTAGAACTGGCAGAAAACAGGGAGATTCTAAAAGAACCGGTACATGGAGTGTATTATGACCCATCAAAAG
ACTTAATAGCAGAAATACAGAAGCAGGGGCAAGGCCAATGGACATATCAAATTTATCAAGAGCCATTTAAAAATCTGAAA
ACAGGAAAGTATGCAAGAATGAAGGGTGCCCACACTAATGATGTGAAACAATTAACAGAGGCAGTACAAAAAATAGCCAC
AGAAAGCATAGTAATATGGGGAAAGACTCCTAAATTT-AAATTACCCATACAAAAGGAAACATGGGAAGCATGGTGGACA
GAGTATTGGCAAGCCACCTGGATTCCTGAGTAGGAGTTTGTCAATACCCCTCCCTTAGTGAAGTTATGGTACCAGTTAGA
GAAAGAACCCATAATAGGAGCAGAAACTTTCTATGTAGATGGGGCAGCCAATAAGGAAACTAAATTAGGAAAAGCAGGAT
ATGTAACTGACAGAGGAAGACAAAAAGTTGTCCCCCTAACGGACACAACAAATCAGAAGACTGAGTTACAAGCAATTCAT
CTAGCTTTGCAGGATTCGGGATTAGAAGTAAACATAGTGACAGACTCACAATATGCATTAGGAATCATTCAAGCACAACC
AGATAAGAGTGAATCAGAGTTAGTCAGTCAAATAATAGAGCAGTTAATAAAAAAGGAAAAAGTCTACCTGGCATGGGTAC
CAGCACACAAAGGAATTAGAGGAAATGAACAAGTAGATAAATTGGTCAGTGCTGGAATCAGGAAAGTACTATTTTTAGAT
GGAATAGATAAGGCCCAAGAAGAACATGAGAAATATCACAGTAATTGGAGAGCAATGGCTAGTGATTTTAACCTACCACC
TGTAGTAGCAAAAGAAATAGTAGCCAGCTGTGATAAATGTCAGCTAAAAGGGGAAGCCATGCATGGACAAGTAGACTGTA
GCCCAGGAATATGGCAGCTAGATTGTACACATTTAGAAGGAAAAGTTATCTTGGTAGCAGTTCATGTAGCCAGTGGATAT
ATAGAAGCAGAAGTAATTCCAGCAGAGACAGGGCAAGAAACAGCATACTTCCTCTTAAAATTAGCAGGAAGATGGCCAGT
AAAAACAGTACATACAGACAATGGCAGCAATTTCACCAGTACTACAGTTAAGGCCGCCTGTTGGTGGGCGGGGATCAAGC
AGGAATTTGGCATTCCCTACAATCCCCAAAGTCAAGGAGTAATAGAATCTATGAATAAAGAATTAAAGAAAATTATAGGA
CAGGTAAGAGATCAGGCTGAACATCTTAAGACAGCAGTACAAATGGCAGTATTCATCCACAATTTTAAAAGAAAAGGGGG
GATTGGGGGGTACAGTGCAGGGGAAAGAATAGTAGACATAATAGCAACAGACATACAAACTAAAGAATTACAAAAACAAA
TTACAAAAATTCAAAATTTTCAGGTTTATTACAGGGACAGCAGAGATCCAGTTTGGAAAGGACCAGCAAAGCTCCTCTGG
AAAGGTGAAGGGGCAGTAGTAATACAAGATAATAGTGACATAAAAGTAGTGCCAAGAAGAAAAGCAAAGATCATCAGGGA
TTATAGAAAACAGATGGCAGGTGATGATTGTGTGGCAAGTAGACAGGATGAGGATTAACACATGGAAAAGATTAGTATAA
TAGCATATGTATATTTCAAGGAAAGCTAAGGACTAGTTTTATAGACATCACTATGAAAGTACTAATCCAAAAATAAGTTC
AGAAGTACACATCCCACTAGGGGATGCTAAATTAGTAATAACAACATATTAGGGTCTGCATACAGGAGAAAGAGACTGGC
ATTTGGGTCAGGGAGTCTCCATAGAATGGAGGAAAAAGAGATATAGCACACAAGTAGACCCTGACCTAGCAGACCAACTA
ATTCATCTGCACTATTTTGATTGTTTTTCAGAATCTGCTATAAGAAATACCATATTAGG---------------------
--------------------------------CTCTACAGTACTTGGCACTAGCAGCATTAATAAAACCAAAACAGATAA
AGCCACCTTTGCCTAGTGTTAGGAAACTGACAAAGGACAGATAGAACAAGCCCCAGAAGACCAAAGGCCACAGAGGGAGC
CATACAATGAATGGACACTAGAGCTTTTAGAGGAACTTAAGAGTGAAGCTGTTAGACATTTTCCTAGGATATGGCTCCAT
AACTTAGGACAACATATCTATGAAACTTACGAGGATACTTGGGCAGGAGTGGAAGCCATAATAAGAATTCTGCAACAACT
GCTGTTTATCCATTTCAGAATTAGGTGTCGACATAGCAGAATAGGCATTACTCGACAGAGGAGAGCAAGAAATGGAGCCA
GTAGATCCTAGACTAGAGCCCTGGAAGCATCCAGGAAGTCAGCCTAAAACTGCTTGTACCAATTGCTATTGTAAAAAGTG
TTGCTTTCATTGCCAAGTTTGTTTCATAACAAAAGCCTTAGGCATCTCCTATGGCAGGAAGAAGCGGAGACAGCGACGAA
GACCTCCTCAAGGCAGTCAGACTCATCAAGTTTCTCTATCAAAGCAGTAAGTAATACATGTAATGCAACCTATACAAATA
GCAATAGTAGCATTAGTAGTAGCAATAATAATAGCAATAGTTGTGTGGTCCATAGTAATCATAGAATATAGGAAAATATT
AAGACAAAGAAAAATAGACAGGTTAATTGATAGACTAATAGAAAGAGCAGAAGACAGTGGCAATGAGAGTGAAGGAGAAA
TATCAGCACTTGTGGAGATAGAGGTGGAGATGGGGCACCATGCTCCTTAGAATGTTAATAATCTGTAGTGCTACAGAAAA

ATTGTGGGTCACAGTCTATTATAAAGTACCTGTGTGGAAGGAAGCAACCACCACTCTATTTTGTGCATCAGATGCTAAAG
CATATAATACAGAGGTACATAATGTTTGGGCCACACATGCCTGTGTACCCACAAACCCCAACCCACAAAAAGTAGTATTA
GTAAATGTGACAGAAAATTTTAACATGTGGAAAAATGACATGGTAGAACAGATGCATGAGGATATAATCAGTTTATAGGA
TCAAAGCCTAAAGCCATGTGTAAAATTAACCCCACTCTGTGTTAGTTTAAAGTGCACTGATTTGAAGAATGATACTAATA
CCAATAGTAGTAGCAGGAGAATGATAATGGAGAAAGGAGAGATAAAAAACTGCTCTTTCAATATCAGCACAAGCATAAGA
GGTAAGGTGCAGAAAGAATATGCATTTTTTTATAAACTTGATATAATACCAATAGATAATGATACTACCAGCTATACGTT
GACAAGTTGTAACACCTCAGTCATTACACAGGCCTGTCCAAAGGTATCCTTTAAGCCAATTCCCATACATTATTGTGCCC
CGGCTGGTTTTGCGATTCTAAAATGTAATAATAAGACGTTCAATGGAACAGGACCATGTACAAATGTCAGCACAGTACAA
TGTACACATGGAATTAGGCCAGTAGTATCAACTCAACTGCTGTTAAATGGCAGTCTAGCAGAAGAAGAGGTAGTAATTAG
ATCTGTCAATTTCACGGACAATGCTAAAACCATAATAGTACAGCTGAACACATCTGTAGAAATTAATTGTACAAGACCCA
ACAACAATACAAGAAAAAAAATCCGTATCCAGAGGGGACCAAGGAGAGCATTTGTTACAATAGGAAAAATAGGAAATATG
AGACAAGCACATTGTAACATTAGTAGAGCAAAATGGAATGCCACTTTAAAACAGATAGCTAGCAAATTAAGAGAACAATT
TGGAAATAATAAAACAATAATCTTTAAGCAATCCTCAGGAGGGGACCCAGAAATTGTAACGCACAGTTTTAATTGTGGAG
GGGAATTTTTCTACTGTAATTCAACACAACTGTTTAATAGTACTTGGTTTAATAGTACTTGGAGTACTGAAGGGTCAAAT
AACACTGAAGGAAGTGACACAATCACACTCCCATGCAGAATAAAACAATTTATAAACATGTGGCAGGAAGTAGGAAAAGC
AATGTATGCCCCTCCCATCAGCGGACAAATTAGATGTTCATCAAATATTACAGGGCTGCTATTAACAAGAGATGGTGGTA
ATAACAACAATGGGTCCGAGATCTTCAGACCTGGAGGAGGAGATATGAAGGACAATTGGAGAAGTGAATTATATAAATAT
AAAGTAGTAAAAATTGAACCATTAGGAGTAGCACCCACCAAGGCAAAGAGAAGAGTGGTGCAGAGAGAAAAAAGAGCAGT
AGGAATAGGAGCTTTGTTCCTTAGGTTCTTAGGAGCAGCAGGAAGCACTATGGGCGCAGCGTCAATGACGCTGACGGTAC
AGGCCAGACAATTATTGTCTGGTATAGTGCAGCAGCAGAACAATTTGCTGAGGGCTATTGAGGCGCAACAGCATCTGTTG
CAACTCACAGTCTGGGGCATCAAGCAGCTCCAGGCAAGAATCCTGGCTGTAGAAAGATACCTAAAGGATCAACAGCTCCT
GGGGATTTGGGGTTGCTCTAGAAAACTCATTTGCACCACTGCTGTGCCTTGCAATGCTAGTTGGAGTAATAAATCTCTGG
AACAGATTTGGAATCACACGACCTGGATGGAGTAGGACAGAGAAATTAACAATTACACAAGCTTAATACACTCCTTAATT
GAAGAATCGCAAAACCAGCAAGAAAAGAATGAACAAGAATTATTAGAATTAGATAAATGGGCAAGTTTGTGGAATTAGTT
TAACATAACAAATTGGCTGTGGTATATAAAATTATTCATAATGATAGTAGGAGGCTTGGTAGGTTTAAGAATAGTTTTTG
CTGTACTTTCTGTAGTGAATAGAGTTAGGCAGGGATATTCACCATTATCGTTTCAGACCCACCTCCCAATCCCGAGGGGA
CCCGACAGGCCCGAAGGAATAGAAGAAGAAGGTGGAGAGAGAGACAGAGACAGATCCATTCGATTAGTGAACGGATCCTT
AGCACTTATCTAGGACGATCTGCGGAGCCTGTGCCTCTTCAGCTACCACCGCTTGAGAGACTTACTCTTGATTGTAACGA
GGATTGTAGAACTTCTGGGACGCAGAGGGTGGGAAGCCCTCAAATATTGGTAGAATCTCCTACAATATTGGAGTCAGGAG
CTAAAGAATAGTGCTGTTAGCTTGCTCAATGCCACAGCTATAGCAGTAGCTGAGAGGACAGATAAGGTTATAGAAGTAGT
ACAAGAAGCTTATAGAGCTATTCGCCACATACCTAGAAGAATAAGACAGGGCTTGGAAAGGATTTTGCTATAAGATAGGT
GGCAAGTGGTCAAAAAGTAGTGTGGTTGGATGGCCTGCTGTAAGGGAAAGAATGAGACGAGCTGAGCCAGCAGCAGATGG
GGTGGGAGCAGCATCTCGAGACCTAGAAAAACATGGAGCAATCACAAGTAGCAACACAGCAGCTAACAATGCTGCTTGTG
CCTGGCTAGAAGCACAAGAGGAGGAGAAGGTAGGTTTTCCAGTCACACCTCAGGTACCTTTAAGACCAATGACTTACAAG
GCAGCTGTAGATCTTAGCCACTTTTTAAAAGAAAAGGGGGGACTGGAAGGGCTAATTCACTCCCAACGAAGACAAGATAT
CCTTGATCTGTGGATCTACCACACACAAGGCTACTTCCCTGATTGGCAGAACTACACACCAGGACCAGGGATCAGATATC
CACTGACCTTTGGATGGCGCTACAAGCTAGTACCAGTTGAGCCAGAGAAGTTAGAAGAAGCCAACAAAGGAGAGAACACC
AGCTTGTTACACCCTGTGAGCCTGCATGGAATGGATGACCCGGAGAGAGAAGTGTTAGAGTGGAGGTTTGACAGCCGCCT
AGCATTTCATCACGTGGCCCGAGAGCTGCATCCGGAGTACTTCAAGAACTGCTGATATCGAGCTTGCTACAAAGGACTTT
CCGCTGGGGACTTTCCAAGGAGGCGTGGCCTGGGCGGGACTGAGGAGTGGCGAGCCCTCAGATCCTGCATATAAGCAGCT
GCTTTTTGCCTGTACTGGGTCTCTCTGGTTAGACCAGATCTGAGCCTGGGAGCTCTCTGGCTAACTAGGGAACCCACTGC
TTAAGCCTCAATAAAGCTTGCCTTGAGTGCTTCAAGTAGTGTGTGCCCATCTGTTGTGTGACTCTGGTAACTAGAGATCC
CTCA
>3-16LONG

------------------------TTTCAGCATTATCAGAAGGAGCCACCCCACAAGATTTAAACACCATGCTAAACACA
GTGGGGGGACATCAAGCAGCCATGCAAATGTTAAAAGAGACCATCAATGAGGAAGCTGCAGAATAGGATAGAGTGCATCC
AGTGCATGCAGGGCCTATTGCACCAGGCCAGATGAGAGAACCAAAAGGAAGTGACATAGCAGGAACTACTAGTACCCTTC
AGGAACAAATAGGATGGATGACACATAATCCACCTATCCCAGTAGGAGAAATCTATAAAAGATGGATAATCCTAGGATTA
AATAAAATAGTAAGAATGTATAGCCCTACCAGCATTCTGGACATAAGACAAGGACCAAAAGAACCCTTTAGAGACTATGT
AGACCGATTCTATAAAACTCTAAGAGCCGAGCAAGCTTCACAAGAGGTAAAAAATTGGATGACAGAAACCTTGTTGGTCC
AAAATGCGAACCCAGATTGTAAGACTATTTTAAAAGCATTAGGACCAGGAGCGACACTAGAAGAAATGATGACAGCATGT
CAGGGAGTAGGAGGACCCGGCCATAAAGCAAGAGTTTTGGCTGAAGCAATGAGCCAAGTAACAAATCCAGCTACCATAAT
GATACAGAAAGGCAATTTTAGGAACCAAAGAAAGACTGTTAAGTGTTTCAATTGTGGCAAAGAAAGGCACATAGCCAAAA
ATTGCAGGGCCCCTAGGAAAAAGGGCTGTTAGAAATGTGGAAAGGAAGGACACCAAATGAAAGATTGTACTGAGAGACAG
GCTAATTTTTTAGGGAAGATCTGGCCTTCCCACAAGGGAAGGCCAAGGAATTTTCTTCAGAGCAGACCAGAGCCAACAGC
CCCACCAGAAGAGAGCTTCAGGTTTGAAGAAGAGACAACAACTCCCTCTCAGAAGCAGGAGCCGATAGACAAAGAACTGT
ATCCTTTAGCTTCCCTCAGATCACTCTTTGGCAGCGACCCCTCGTCACAATAAAGATAGGGGGGCAATTAAAGGAAGCTC
TATTAGATACAGGAGCAGATGATACAGTATTAGAAGAAATGAATTTGCCAGGAAGATGGAAACCAAAAATGATAGGGGGA
ATTGGAGGTTTTATCAAAGTAAGACAGTATGATCAGATACTCATAGAAATCTGCGGACATAAAGCTATAGGTACAGTATT
AGTAGGACCTACACCTGTCAACATAATTGGAAGAAATCTGTTGACTCAGATTGGCTGCACTTTAAATTTTCCCATTAGTC
CTATTGAGACTGTACCAGTAAAATTAAAGCCAGGAATAGATGGCCCAAAAGTTAAACAATGGCCATTGACAGAAGAAAAA
ATAAAAGCATTAGTAGAAATTTGTACAGAAATGGAAAAGGAAGGAAAAATTTCAAAAATTAGGCCTGAAAATCCATACAA
TACTCCAGTATTTGCCATAAAGAAAAAAGACAGTACTAAATGGAGAAAATTAGTAGATTTCAGAGAACTTAATAAGAGAA
CTCAAGATTTCTAAGAAGTTCAATTAGGAATACCACATCCTGCAGGGTTAAAACAGAAAAAATCAGTAACAGTACTGGAT
GTGGGCGATGCATATTTTTCAGTTCCCTTAGATAAAGACTTCAGGAAGTATACTGCATTTACCATACCTAGTATAAACAA
TGAGACACCAGGGATTAGATATCAGTACAATGTGCTTCCACAGAGATGGAAAGGATCACCAGCAATATTCCAGTGTAGCA
TGACAAAAATCTTAGAGCCTTTTAGAAAACAAAATCCAGACATAGTCATCTATCAATACATGGATGATTTGTATGTAGGA
TCTGACTTAGAAATAGGGCAGCATAGAACAAAAATAGAGGAACTGAGACAACATCTGTTGAGGTAGAGATTTACCAC---
----
--TTAGGCAAGTCAGATT
TATGCAGGGATTAAAGTAAGGCAATTATGTAAACTTCTTAGGGGAACCAAAGCACTAACAGAAGTAGTACCACTAACAGA
AGAAGCAGAGCTAGAACTGGCAGAAAACAGGGAGATTCTAAAAGAACCGGTACATGGAGTGTATTATGACCCATCAAAAG
ACTTAATAGCAGAAATACAGAAGCAGAGGCAAGGCCAATGGACATATCAAATTTATCAAAAGCCATTTAAAAATCTGAAA
ACAGGAAAGTATGCAAGAATGAAGGGTGCCCACACTAATGATGTGAAACAATTAACAGAGGCAGTACAAAAAATAGCCAC
AGAAAGCATAGTAATATGGGGAAAGACTCCTAAATTT-AAATTACCCATACAAAAGGAAACATAGGAAGCATGGTGGACA
GAGTATTGGCAAGCCACCTAGATTCCTGAGTGGGAGTTTGTCAATACCCCTCCCTTAGTGAAGTTATGGTACCAGTTAGA
GAAAGAACCCATAATAGGAGCAGAAACTTTCTATGTAGATAGGGCAGCCAATAGGGAAACTAAATTAGGAAAAGCAGGAT
ATGTAACTGACAGAGGAAGACAAAAAGTTGTCCCCCTAACGGACACAACAAATCAGAAGACTGAGTTACAAGCAATTCAT

CTAGCTTTGCAGGATTCAGGATTAGAAGTAAACATAGTGACAGACTCACAATATGCATTAGGAATCATTCAAGCACAACC
AGATAAGAGTGAATCAGAGTTAGTCAGTCAAATAATAGAGCAGTTAATAAAAAAGGAAAAAGTCTACCTGGCATAGGTAC
CAGCACACAAAGGAATTGGAGGAAATGAACAAGTAGATAAATTGGTCAGTGCTAGAATCAGGAAAGTACTATTTTTAGAT
AGAATAGATAAGGCCCAAGAAGAACATAAGAAATATCACAGTAATTAGAGAGCAATGGCTAGTGATTTTAACCTACCACC
TGTAGTAGCAAAAGAAATAGTAGCCAGCTGTGATAAATGTCAGCTAAAAGGGGAAGCCATGCATGGACAAGTAGACTGTA
GCCCAGGAATATGGCAGCTAGATTGTACACATTTAGAAGGAAAAGTTATCTTGGTAGCAGTTCATGTAGCCAGTGGATAT
ATAGAAGCAGAAGTAATTCCAGCAGAGACAGGGCAAGAAACAGCATACTTCCTCTTAAAATTAGCAGGAAGATGGCCAGT
AAAAACAGTACATACAGACAATGGCAGCAATTTCACCAGTACTACAGTTAAGGCCGCCTGTTGGTGGGCGAGGATCAAGC
AGGAATTTGGCATTCCCTACAATCCCCAAAGTCAAGAAGTAATAGAATCTATGAATAAAGAATTAAAGAAAATTATAGGA
CAGGTAAGAGATCAGGCTGAACATCTTAAGACAGCAGTACAAATGGCAGTATTCATCCACAATTTTAAAAGAAAAGGGGG
GATTGGGGGGTACNGTGCAGGGGAAAGAATAGTAGACATAATAGCAACAGACATACAAACTAAAGAATTACAAAAACAAA
TTACAAAAATTCAAAATTTTCAGGTTTATTACAGGGACAGCAGAGATCCAGTTTGGAAAGGACCAGCAAAGCTCCTCTGG
AAAGGTGAAGGGGCAGTAGTAATACAAGATAATAGTGACATAAAAGTAGTGCCAAGAAGAAAAGCAAAGATCATCAGGGA
TTATGGAAAACAGATGGCAGGTGATGATTGTGTGGCAAGTAGACAGGATGAGGATTAACACATGGAAAAGATTAGTATAA
TAGCATATGTATATTTCAAGGAAAGCTAAGGACTGGTTTTATAGACATCACTATGAAAGTACTAATCCAAAAATAAGTTC
AGAAGTACACATCCCACTAGGGGATGCTAAATTAGTAATAACAACATATTGGGGTCTGCATACAGGAGAAAGAGACTGGC
ATTTAGGTCAGGGAGTCTCCATAGAATGGAGGAAAAAGAGATATAGCACACAAGTAGACCCTGACCTAGCAGACCAACTA
ATTCATC---
----------------------------GGATCTCTACAGTACTTGGCACTAGCAGCATTAATAAAACCAAAACAGATAA
AGCCACCTTTGCCTAGTGTTAGGAAACTGACAGAGGACAGATGGAACAAGCCCCAGAAGACCAAGGGCCACAGAGGGAGC
CATACAATGAATGGACACTAGAGCTTTTAGAGGAACTTAAGAGTGAAGCTGTTAGACATTTTCCTAGGATATGGCTCCAT
AACTTAGGACAACATATCTATGAAACTTACGGGGATACTTGGGCAGGAGTGGAAGCCATAATAAGAATTCTGCAACAACT
GCTGTTTATCCATTTCAGAATTAGGTGTCGACATAGCAGAATAGGCGTTACTCGACAGAGGAGAGCAAGAAATGGAGCCA
GTAGATCCTAGACTAGAGCCCTGGAAGCATCCAGGAAGTCAGCCTAAAACTGCTTGTACCAATTGCTATTGTAAAAAGTG
TTGCTTTCATTGCCAAGTTTGTTTCATAACAAAAGCCTTAGGCATCTCCTATGGCAGGAAGAAGCGGAGACAGCGACGAA
GACCTCCTCAAGGCAGTCAGACTCATCAAGTTTCTCTATCAAAGCAGTAAGTAATACATGTAATGCAACCTATACAAATA
GCAATAGTAGCATTAGTAGTAGCAATAATAATAGCAATAGTTGTGTGGTCCATAGTAATCATAGAATATAGGAAAATATT
AAGACAAAGAAAAATAGACAGGTTAATTGATAGACTAATAGAAAGAGCAGAAGACAGTGGCAATGAGAGTGAAGGAGAAA
TATCAGCACTTGTGGAGATAGGGGTGGAGATAGGGCACCATGCTCCTTGGGATGTTGATGATCTGTAGTGCTACAGAAAA
ATTGTAGGTCACAGTCTATTATAGAGTACCTGTGTAGAAGGAAGCAACCACCACTCTATTTTGTGCATCAGATGCTAAAG
CATATGATACAGAGGTACATAATGTTTAGGCCACACATGCCTGTGTACCCACAGACCCCAACCCACAAGAAGTAGTATTG
GTAAATGTGACAGAAAATTTTAACATGTGGAAAAATGACATGGTAGAACAGATGCATGAGGATATAATCAGTTTATAGGA
TCAAAGCCTAAAGCCATGTGTAAAATTAACCCCACTCTGTGTTAGTTTAAAGTGCACTGATTTGAAGAATGATACTAATA
CCAATAGTAGTAGCAGAAGAATGATAATGGAGAAAGGAGAGATAAAAAACTGCTCTTTCAATATCAGCACAAGCATAAGA
GGTAAGGTGCAGAAAGAATATGCATTTTTTTATAAACTTGATATAATACCAATAGATAATGATACTACCAGCTATACGTT
GACAAGTTGTAACACCTCAGTCATTACACAGGCCTGTCCAAAGGTATCCTTTGAGCCAATTCCCATACATTATTGTGCCC
CGGCTAGTTTTGCGATTCTAAAATGTAATAATAAGACGTTCAATGGAACAGGACCATGTACAAATGTCAGCACAGTACAA
TGTACACATGGAATTAGGCCAGTAGTATCAACTCAACTGCTGTTAAATGGCAGTCTAGCAGAAGAAGAGGTAGTAATTAG
ATCTGTCAATTTCACGGACAATGCTAAAACCATAATAGTACAGCTGAACACATCTGTAGAAATTAATTGTACAAGACCCA
ACAACAATACAAGAAAAAAAATCCGTATCCAGAAGGGACCAGGGAGAGCATTTGTTACAATAGGAAAAATAGGAAATATG
AGACAAGCACATTGTAACATTAGTAGAGCAAAATGGAATGCCACTTTAAAACAGATAGCTAGCAAATTAAGAGAACAATT
TGGAAATAATAAAACAATAATCTTTAAGCAATCCTCAGGAGAGGACCCAGAAATTGTAACGCACAGTTTTAATTGTGGAG
AGGAATTTTTCTACTGTAATTCAACACAACTGTTTAATAGTACTTGGTTTAATAGTACTTAGAGTACTGAAAGGTCAAAT
AACACTGAAGGAAGTGACACAATCACACTCCCATGCAGAATAAAACAATTTATAAACATGTGGCAGGAAGTAGGAAAAGC
AATGTATGCCCCTCCCATCAGCGGACAAATTAGATGTTCATCAAATATTACAGGGCTGCTATTAACAAGAGATGGTGGTA
ATAACAACAATGGGTCCGAGATCTTCAGACCTGGAGGAGGAGATATGAGGGACAATTGGAGAAGTGAATTATATAAATAT
AAAGTAGTAAAAATTGAACCATTAGGAGTAGCACCCACCAAGGCAAAGAGAAGAGTGGTGCAGAGAGAAAAAAGAGCAGT
AGGAATAGGAGCTTTGTTCCTTAGGTTCTTAGGAGCAGCAGGAAGCACTATGGGCGCAGCGTCAATGACGCTGACGGTAC
AGGCCAGACAATTATTGTCTGGTATAGTGCAGCAGCAGAACAATTTGCTGAGGGCTATTGAGGCGCAACAGCATCTGTTG
CAACTCACAGTCTGGGGCATCAAGCAGCTCCAGGCAAGAATCCTGGCTGTGGAAAGATACCTAAAGGATCAACAGCTCCT
GGGGATTTGGGGTTGCTCTGGAAAACTCATTTGCACCACTGCTGTGCCTTGGAATGCTAGTTGGAGTAATAAATCTCTAG
AACAGATTTGGAATCACACGACCTGGATGGAGTGGGACAGAGAAATTAACAATTACACAAGCTTAATACACTCCTTAATT
GAAGAATCGCAAAACCAGCAAGAAAAGAATGAACAAGAATTATTGGAATTAGATAAATGGGCAAGTTTGTGGAATTGGTT
TAACATAACAAATTGGCTGTGGTATATAAAATTATTCATAATGATAGTAGGAGGCTTGGTAGGTTTAAGAATAGTTTTTG
CTGTACTTTCTGTAGTGAATAGAGTTAGGCAGGGATATTCACCATTATCGTTTCAGACCCACCTCCCAATCCCGAGGGGA
CCCGACAGGCCCGAAGGAATAGAAGAAGAAGGTGGAGAGAGAGACAGAGACAGATCCATTCGATTAGTGAACGGATCCTT
AGCACTTATCTGGGACGATCTGCGGAGCCTGTGCCTCTTCAGCTACCACCGCTTGAGAGACTTACTCTTGATTGTAACGA
GGATTGTGGAACTTCTAGGACGCAGGAGGTAGGAAGCCCTCAAATATTGGTGGAATCTCCTACAATATTGGAGTCAGGAG
CTAAAGAATAGTGCTGTTAGCTTGCTCAATGCCACAGCTATAGCAGTAGCTGAGGGGACAGATAGGGTTATAGAAGTAGT
ACAAGAAGCTTATAGAGCTATTCGCCACATACCTAGAAGAATAAGACAGGGCTTAGAAAGGATTTTGCTATAAGATGGGT
GGCAAGTGGTCAAAAAGTAGTGTGGTTGGATGGCCTGCTGTAAGGGAAAGAATGAGACGAGCTGAGCCAGCAGCAGATGG
GGTGGGAGCAGCATCTCGAGACCTAGAAAAACATGGAGCAATCACAAGTAGCAACACAGCAGCTAACAATGCTGCTTGTG
CCTGGCTAGAAGCACAAGAGGAGGAGAAGGTAGGTTTTCCAGTCACACCTCAGGTACCTTTAAGACCAATGACTTACAAG
GCAGCTGTAGATCTTAGCCACTTTTTAAAAGAAAAGGGGGGACTGGAAGGGCTAATTCACTCCCAACGAAGACAAGATAT
CCTTGATCTGTGGATCTACCACACACAAGGCTACTTCCCTGATTGGCAGAACTACACACCAGGACCAGGGATCAGATATC
CACTGACCTTTGGATGGCGCTACAAGCTAGTACCAGTTGAGCCAGAGAAGTTAGAAGAAGCCAACAAAGGAGAGAACACC
AGCTTGTTACACCCTGTGAGCCTGCATGGAATGGATGACCCGGAGAGAGAAGTGTTAGAGTGGAGGTTTGACAGCCGCCT
AGCATTTCATCACGTGGCCCGAGAGCTGCATCCGGAGTACTTCAAGAACTGCTGATATCGAGCTTGCTACAAGGGACTTT
CCGCTGGGGACTTTCCAGGGAGGCGTGGCCTGGGCGGGACTGGGGAGTGGCGAGCCCTCAGATCCTGCATATAAGCAGCT
GCTTTTTGCCTGTACTAGGTCTCTCTGGTTAGACCAGATCTGAGCCTGGGAGCTCTCTGGCTAACTAGGGAACCCACTGC
TTAAGCCTCAATAAAGCTTGCCTTGAGTGCTTCAAGTAGTGTGTGCCCATCTGTTGTGTGACTCTGGTAACTAGAGATCC
CTCA
