# Supplementary material for: APOBEC3G-Induced Hypermutation of Human Immunodeficiency Virus Type-1 Is Typically a Discrete “All or Nothing” Phenomenon
Source: PLoS Genet. 2012 Mar 22;8(3):e1002550. doi: 10.1371/journal.pgen.1002550 (PMC3310730; doi:10.1371/journal.pgen.1002550)
Supplement: Table S1 — Mutation summary. (PDF) [file pgen.1002550.s003.pdf]

Supplementary table 1.
Mutation summary

Titration	All sequences						Hypermutated sequences only						Non-hypermutated sequences only					
**1**	From/To	A	C	G	T	Total	From/To	A	C	G	T	Total	From/To	A	C	G	T	Total
**0% wt-hA3G**	A		0	0	0	4970	A		0	0	0	0	A		0	0	0	4970
**100% E259Q-hA3G**	C	0		0	1	3272	C	0		0	0	0	C	0		0	1	3272
**(8 sequences)**	G	1	0		0	4601	G	0	0		0	0	G	1	0		0	4601
**(0 hypermutated)**	T	0	0	0		3800	T	0	0	0		0	T	0	0	0		3800
**2**	From/To	A	C	G	T	Total	From/To	A	C	G	T	Total	From/To	A	C	G	T	Total
**1% wt-hA3G**	A		1	0	0	12557	A		1	0	0	1284	A		0	0	0	11273
**99% E259Q-hA3G**	C	1		0	1	8242	C	0		0	0	838	C	1		0	1	7404
**(19 sequences)**	G	61	0		0	11575	G	58	0		0	1174	G	3	0		0	10401
**(2 hypermutated)**	T	0	6	0		9587	T	0	1	0		974	T	0	5	0		8613
**3**	From/To	A	C	G	T	Total	From/To	A	C	G	T	Total	From/To	A	C	G	T	Total
**3.3% wt-hA3G**	A		0	3	0	11715	A		0	0	0	5618	A		0	3	0	6097
**96.7% E259Q-hA3G**	C	0		0	0	7734	C	0		0	0	3682	C	0		0	0	4052
**(19 sequences)**	G	218	1		0	10840	G	218	1		0	5177	G	0	0		0	5663
**(9 hypermutated)**	T	0	1	1		8978	T	0	1	0		4287	T	0	0	1		4691
**4**	From/To	A	C	G	T	Total	From/To	A	C	G	T	Total	From/To	A	C	G	T	Total
**10% wt-hA3G**	A		1	3	0	11950	A		0	3	0	9456	A		1	0	0	2494
**90% E259Q-hA3G**	C	1		0	0	7843	C	1		0	0	6208	C	0		0	0	1635
**(19 sequences)**	G	489	3		5	11002	G	488	3		5	8694	G	1	0		0	2308
**(15 hypermutated)**	T	0	1	0		9106	T	0	1	0		7205	T	0	0	0		1901
**5**	From/To	A	C	G	T	Total	From/To	A	C	G	T	Total	From/To	A	C	G	T	Total
**33% wt-hA3G**	A		0	0	1	9434	A		0	0	1	8802	A		0	0	0	632
**67% E259Q-hA3G**	C	1		0	2	6181	C	1		0	2	5768	C	0		0	0	413
**(15 sequences)**	G	1039	1		4	8689	G	1038	1		3	8107	G	1	0		1	582
**(14 hypermutated)**	T	0	2	0		7189	T	0	2	0		6707	T	0	0	0		482
**6**	From/To	A	C	G	T	Total	From/To	A	C	G	T	Total	From/To	A	C	G	T	Total
**100% wt-hA3G**	A		1	0	0	4854	A		1	0	0	4854	A		0	0	0	0
**0% E259Q-hA3G**	C	0		0	2	3200	C	0		0	2	3200	C	0		0	0	0
**(8 sequences)**	G	590	1		2	4486	G	590	1		2	4486	G	0	0		0	0
**(8 hypermutated)**	T	0	1	0		3719	T	0	1	0		3719	T	0	0	0		0
	From/To	A	C	G	T	Total	From/To	A	C	G	T	Total	From/To	A	C	G	T	Total
	A		3	6	1	55480	A		2	3	1	30014	A		1	3	0	25466
**TOTAL**	C	3		0	6	36472	C	2		0	4	19696	C	1		0	2	16776
	G	2398	6		11	51193	G	2392	6		10	27638	G	6	0		1	23555
	T	0	11	1		42379	T	0	6	0		22892	T	0	5	1		19487
	**TOTAL**	**2401**	**20**	**7**	**18**	**185524**	**TOTAL**	**2394**	**14**	**3**	**15**	**100240**	**TOTAL**	**7**	**6**	**4**	**3**	**85284**
