# Supplementary material for: APOBEC3G-Induced Hypermutation of Human Immunodeficiency Virus Type-1 Is Typically a Discrete “All or Nothing” Phenomenon
Source: PLoS Genet. 2012 Mar 22;8(3):e1002550. doi: 10.1371/journal.pgen.1002550 (PMC3310730; doi:10.1371/journal.pgen.1002550)
Supplement: Table S2 — PCR primers. (PDF) [file pgen.1002550.s004.pdf]

Supplementary Table 2.

PCR primers:

PROJECT	Primer Name	Nested PCR Round	F /R	Fragment	HXB2# (3' base)	Fragment Length (kb)	Sequence (5'-to-3')
**Near-full length APOBEC3G/3F - PCR**	Vifhyp R1F Gag	1	Forward	Gag - 3'LTR	1060	8.63	TTATATAATACAGTAGCAACCCTCTATTGTGTGCATC
	Vifhyp R1R U5 3'LTR	1	Reverse	Gag - 3'LTR	9688	8.63	TGCTAGAGATTTT**YY**ACACTGACTAAAAGGG
	1F Gag	2a	Forward	Gag-Pol	1151	2.13	GTAAGAAAAAAGCACAGCAAGCAGCAGC
	2R GagPol	2a	Reverse	Gag-Pol	3277	2.13	CCTTTTCTGGCAGCACTATAGGCTGTACTG
	3F Pol	2b	Forward	Pol-Vif	3271	2.22	GAAAGGATCACCAGCAATATTCCAGTGTAGC
	4R PolVif	2b	Reverse	Pol-Vif	5488	2.22	CTTTATCTGTTTTGGTTTTATTAATGCTGCTAGTGC
	5F VifEnv	2c	Forward	Vif-Env	5368	2.46	GACCCTGACCTAGCAGACCAACTAATTCATC
	6REnv	2c	Reverse	Vif-Env	7824	2.46	CTGGCCTGTACCGTCAGCGTCATTG
	7F EnvNef1	2d	Forward	Env-3'LTR	7542	2.14	AAAGCAATGTATGCCCCTCCCATCAG
	9R U5 3'LTR	2d	Reverse	Env-3'LTR	9681	2.14	TTCCACACTGACCTAAAAGGGT**Y**TGAGG
**Near-full length APOBEC3G/3F - Sequencing**	All of the above and:						
	1R Gag	seq	Reverse	Gag	2239	-	AAAGAGTGATCTGAGGGAAGCTAAAGGATACAG
	2F GagPol	seq	Forward	GagPol	1916	-	AAGCAATGAGCCAAGTAACAAATCCAGCTAC
	3R Pol	seq	Reverse	Pol	4458	-	TGCTTCTATATATCCACTGGCTACATGAACTGC
	4F PolVif	seq	Forward	Pol	4081	-	GCATTGGGAATCATTCAAGCACAACC
	5R VifEnv	seq	Reverse	Env-Vif	6569	-	ACAGAGTGGGGTTAATTTTACACATGGCTTTAG
	6F Env	seq	Forward	Env	6390	-	GGAAGCAACCACCACTCTATTTTGTGCATC
	7R EnvNef1	seq	Reverse	Env	8920	-	TTGTTAGCTGCTGTGTTGCTACTTGTGATTG
	8F EnvNef2	seq	Forward	Env-Nef	8392	-	ATTATCGTTTCAGACCCACCTCCCAATC
	9F Nef3'LTR	seq	Forward	Nef-3'LTR	9181	-	CTACTTCCCTGATTGGCAGAACTACACACC
**PATIENT-DERIVED VIF - PCR**	Vif 5 Outer	1	Forward	Vif	4843	1.00	GCAGGGGAAAGAATARTAGACATAATAGC
	Vif 3 Outer	1	Reverse	Vif	5840	1.00	GGATGCTTCCAGGGCTCTAGKYTAGGATCTAC
	Vif 5 B Inner	2	Forward	Vif	4966	0.80	AAGGACCAGCAAAGCTYCTCTGGAAAGGTGA
	Vif 3 Inner	2	Reverse	Vif	5773	0.80	GCCTGTTCTGCTATGTYGACACCCAATTCTGAA

PCR cycling conditions:

Near-full length Vif-APOBEC3G/3F - R1-PCR	Vifhyp R1L ann60	95ºC (1')	95ºC (30") -> 60ºC (30") -> 68ºC (10') - (15 cycles)	95ºC (30") -> 68ºC (10') - (20 cycles)	68ºC (10') -> 4ºC end
Near-full length Vif-APOBEC3G/3F - R2-PCR	Vifhyp R2 ann60	95ºC (1')	95ºC (30") -> 60ºC (30") -> 68ºC (3') - (15 cycles)	95ºC (30") -> 68ºC (3') - (20 cycles)	68ºC (10') -> 4ºC end
Patient-derived vif - R1-PCR	Vif R1	95ºC (1')	95ºC (30") -> 68ºC (3') - (35 cycles)	-	68ºC (3') -> 4ºC end
Patient-derived vif - R2-PCR	Vif R2	95ºC (1')	95ºC (30") -> 68ºC (3') - (35 cycles)	-	68ºC (10') -> 4ºC end
