# Supplementary material for: APOBEC3G-Induced Hypermutation of Human Immunodeficiency Virus Type-1 Is Typically a Discrete “All or Nothing” Phenomenon
Source: PLoS Genet. 2012 Mar 22;8(3):e1002550. doi: 10.1371/journal.pgen.1002550 (PMC3310730; doi:10.1371/journal.pgen.1002550)
Supplement: Table S3 — GenBank accession numbers. (PDF) [file pgen.1002550.s005.pdf]

Supplementary Table 3; accession numbers

Hypermutant Reference	Sequence Name	Accession Number	Subtype	Accession Numbers of Closely Related Non-Hypermutated Sequences for Generating Reference Sequence Estimate (identified from tree based on alignment of 'repaired' sequences)
01AE_a	OUR700I	AY358058	01AE	AY358061, AY358049, AY358051, AY358052, DQ789392
01AE_b	00TH_C1705HYP	AY945715	01AE	AY358061, AY358049, AY358051, AY358052, DQ789392
01AE_c	OUR658I	AY358055	01AE	AY358067, AY358062, AY358037, AY945716, AY358059, AY945712
01AE_d	OUR736I	AY358054	01AE	AY358067, AY358062, AY358037, AY945716, AY358059, AY945712
01AE_e	OUR598I	AY358053	01AE	AY358067, AY358062, AY358037, AY945716, AY358059, AY945713
01AE_g	00TH_C4141HYP	AY945723	01AE	AY945723
01AE_h	00TH_C1468HYP	AY945714	01AE	AY945720, AY945730, AY713421, AY945722
02AG_a	01CM1188NG	AY371135	02AG	AY371132, AY271690, AY371138, AY371125, AY371129, AY371137, AY371136
02AG_b	98US_MSC5007	AY444808	02AG	AF063223, AF063224, DQ168578, AY371133
02AG_c	98US_MSC4041	AY444810	02AG	DQ168577, AY444809, AY829204, AY829207, AY829214, DD409979
12BF	ARMA185	AY037279	12BF	AF408630, AF385934, AY536238, AF408629, AF385935, AF037272, AF385936
A1_a	NKU3007	AF457091	A1	AF457066, AF457077, AF004885
A1_b	KSM4023	AF457076	A1	AF361872, AF457067, AF457070, AF457080, AF457053, AF457075
A1_c	KNH1214	AF457071	A1	AF361872, AF457067, AF457070, AF457080, AF457053, AF457075
A1_d	KER2018	AF457057	A1	AF457089, AF484493, U51190
A1_e	UGC27305	AF484484	A1	AF457083, AF069673, AF484478, AF484509, AF484508
A1D_a	TZBFL0169	AF442568	A1D	AY253316, AF442566, AF442570
A1D_b	KSM4017	AF457074	A1D	AF457078, AF071473, AF457082
A1D_c	170_3_2	AF237166	A1D	AF484510, AF484517, AF484521, AF484522
B_a	ARMA173	AY037274	B	DQ383748
B_b	COL105	AY561241	B	AY037268, DQ207942, AY037282, DQ383746
B_c	02UZ0673	AY829213	B	AY037268, DQ207942, AY037282, DQ383746
B_d	01UYTRA1101	AY781125	B	AY037268, DQ207942, AY037282, DQ383746
B_e	LTG0218	AY531116	B	DQ007903, DQ007902, DQ007901, AY180905, U71182
B_j	CANB6FULL	AY779559	B	AY779553, AY779554, AY779555
BF_a	ARMA062	AY037273	BF	AF408628, AY037267, AY771593, AY771592
BF_b	ARMA070	AY037276	BF	AF408628, AY037267, AY771593, AY771592
C_a	03ZASK071B1	DQ056407	C	DQ396374, DQ396375, DQ351216, DQ396378, AY878063, DQ164109, DQ093588, AY878067, DQ164114, DQ396382, DQ056406
C_b	03ZASK008B2	DQ164123	C	DQ275647, AY901966, AY901972
C_c	03ZASK086B1	DQ164124	C	DQ275647, AY901966, AY901972
C_d	04ZASK048B2	DQ164128	C	DQ369989, DQ093599
C_e	04ZAPS187B1	DQ164125	C	DQ164122, DQ093602
C_f	03ZASK012B2	DQ275665	C	DQ093594, DQ396377, DQ164107, DQ396373, AY463217
C_g	99ET8	AY255828	C	AY255823, AY713417, U46016, AY255827, AY255825
C_h	CO883	AY734557	C	AY253308, AY253310, AY043176
O	VAUAF407419	AF407419	O	AF407418 (From same patient)
01C	C2267HYP	AY945735	01C	AY262830
16_A2D	KISII5009	AF457060	16_A2D	AY945736
01AE-fromScreen	Fj061	EF036536	01AE	EF03652, EF03652, EF036530
